# Lipid-lowering and glucose-lowering drug targets differentially modulate antipsychotic treatment efficacy in schizophrenia

**DOI:** 10.1016/j.xcrm.2026.102653

**Published:** 2026-03-17

**Authors:** Yunqing Zhu, Rui Yuan, Zhe Lu, Yuyanan Zhang, Zhewei Kang, Xiaoyang Feng, Guorui Zhao, Junyuan Sun, Jing Guo, Tong Yu, Yang Yang, Yaoyao Sun, Weihua Yue

**Affiliations:** 1 Peking University Sixth Hospital, Peking University Institute of Mental Health, NHC Key Laboratory of Mental Health (Peking University), National Clinical Research Center for Mental Disorders (Peking University Sixth Hospital), Beijing 100191, China; 2 PKU-IDG/McGovern Institute for Brain Research, Peking University, Beijing 100871, China

**Keywords:** apolipoprotein C3, glucokinase, schizophrenia, antipsychotic efficacy

## Abstract

Schizophrenia is frequently comorbid with dyslipidemia and hyperglycemia. However, whether metabolic-modifying agents aggravate schizophrenia progression remains unclear. We perform a drug-target genetic association study in two independent Han Chinese schizophrenia cohorts (*N* = 2,111/292 for discovery/validation). Leveraging metabolic genome-wide association studies, we generate genetic risk scores (GRSs) for lipid-modifying and hypoglycemic targets. Those with higher *APOC3* (inhibited by volanesorsen/olezarsen) GRS exhibit attenuated triglycerides and improvement in negative symptoms assessed by Positive and Negative Syndrome Scale (PANSS) (*β* = 1.23, 95% confidence interval [CI]: 0.30–2.16). Higher *GCK* (activated by dorzagliatin) GRS is associated with decreased glucose and less improvement across PANSS total (*β* = −1.70, 95% CI: −2.91–0.50), positive, negative, general subscales. Causal associations of *GCK* are replicated in independent validation. The effects of *APOC3* and *GCK* on negative symptom recovery are robust in hyperlipidemic/diabetic subgroups. Genetically proxied proteomics analysis provides further functional validation for the identified target-outcome associations. Our findings suggest volanesorsen/olezarsen as potential adjunctive candidates; dorzagliatin warrants prudence in schizophrenia with metabolic disturbance.

## Highlights

• *APOC3*-proxied lipid lowering elevates antipsychotic efficacy of negative symptoms
• *GCK*-proxied glucose lowering reduces positive, negative, general-symptom recovery
• *APOC3/GCK* negative-symptom effects persist in hyperlipidemic/diabetic subgroups
• Genetic proteomics validate blood-APOC3, brain-GCK effects on negative symptoms

## Graphical abstract

**Figure:**
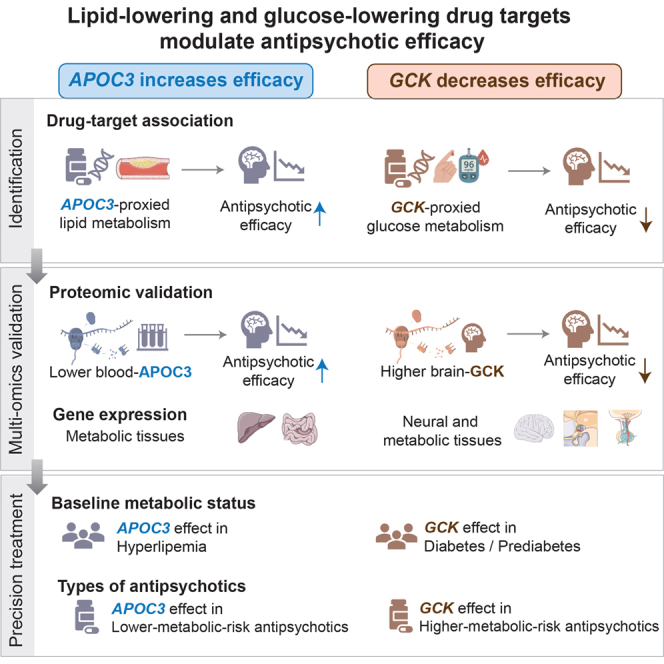

## Teaser

Whether metabolic-modifying agents aggravate schizophrenia progression remains unclear. Zhu et al. identify that *APOC3*-proxied lipid lowering improves schizophrenia negative-symptom recovery, and *GCK*-related glucose lowering worsens antipsychotic efficacy. Causal effects of *APOC3/GCK* on negative symptoms are robust in hyperlipidemic/diabetic subgroups. This study suggests volanesorsen/olezarsen as potential adjunctive candidates, and dorzagliatin warrants prudence in schizophrenia-metabolic comorbidity.

## Introduction

Schizophrenia (SCZ) carries a heavy psychiatric burden worldwide, leading to considerable disability and substantial financial costs. Compared to the general population, individuals with SCZ have a markedly reduced life expectancy by approximately 16–20 years, mainly attributed to comorbid cardiometabolic diseases.^1^ A global meta-analysis of 198 studies reported a pooled prevalence of metabolic disturbance of 33.4% in SCZ, representing a 1.87-fold higher risk than in the general population.^2^ Hypertriglyceridemia and diabetes were associated with 2.73- and 1.99-fold elevated risks, respectively.^3^ Several key contributors to this increasing comorbidity included the antipsychotic-induced metabolic side effects, shared genetic susceptibility between SCZ and metabolic indicators,^4^ unhealthy diets, and few physical activities.^5^ Previous clinical studies reported that lipid-modifying drugs may induce neuropsychiatric adverse effects, including paranoia, irritability, depression, and cognitive impairments.^6^^,^^7^ Adjuvant metformin might also worsen psychotic symptoms in SCZ.^8^ Given concerns that lipid- and glucose-modifying agents may exacerbate psychiatric symptoms, clinicians are less likely to prescribe metabolic medications in those with SCZ (37%) than in non-SCZ populations (85%).^9^^,^^10^ Thus, it is critical to explore the potential impact of commonly prescribed metabolic-regulating drugs on the progress of psychotic symptoms, which could promote coordinated treatments for individuals co-occurring with SCZ and metabolic disorders.

To date, an increasing number of randomized clinical trials (RCTs) have examined whether metabolic medicines can modify antipsychotic efficacy in SCZ. Most trials of lipid-modifying drugs have centered on statins. The latest meta-analysis involving nine RCTs (*N* = 533) found that adjunctive statin therapy was associated with improvements in negative symptoms in SCZ.^11^ However, the potential influence of other classes of agents on the antipsychotic efficacy remains largely unknown. Moreover, previous clinical trials demonstrated inconsistent evidence for different types of glucose-lowering agents on the efficacy of antipsychotics. A recent multicenter RCT reported that adding semaglutide, a glucagon-like peptide-1 receptor agonist, to quetiapine therapy did not affect psychotic symptom recovery (*N* = 31).^12^ Similarly, a meta-analysis (10 RCTs, *N* = 453) indicated that current evidence did not support an impact of topiramate on the clinical improvement of SCZ.^13^ In contrast, another RCT conducted among 40 patients with SCZ suggested a potential benefit of pioglitazone adjuvant therapy on the reduction rate of Positive and Negative Syndrome Scale (PANSS) total score and negative scale.^14^

The heterogeneity of these clinical findings suggests that specific glucose-lowering drug targets might differently modulate the antipsychotic efficacy. Similar target-specific dynamics extend to distinct lipid-lowering targets. Comprehensive profiling of the known lipid-modifying and antidiabetic targets will help detect the targets affecting the SCZ symptoms, thereby informing the co-prescribing in psychiatric practice.

Our recent work pinpointed several pleiotropic loci shared between SCZ and cardiometabolic traits among East Asians, motivating the identification of common therapeutic targets through a genetics-based approach. The drug-target Mendelian randomization (MR) analysis applies genetic instruments in each target gene region. This strategy can mimic a randomly assigned drug-intervention study for causal inference, thereby reducing the reverse and confounding bias in observational studies.^15^^,^^16^ By concentrating on well-defined pharmacologically active targets, this approach offers deeper mechanistic insight into drug action and is more practical and cost-effective than traditional RCTs.^17^ None of the previous studies have evaluated the drug-target effects on SCZ symptoms from a genomic aspect. Given the complex metabolic disturbances in patients with SCZ,^18^ examining the joint impact of glucose-modulating and lipid-modulating drug targets on antipsychotic efficacy is necessary.

**Figure 1:**
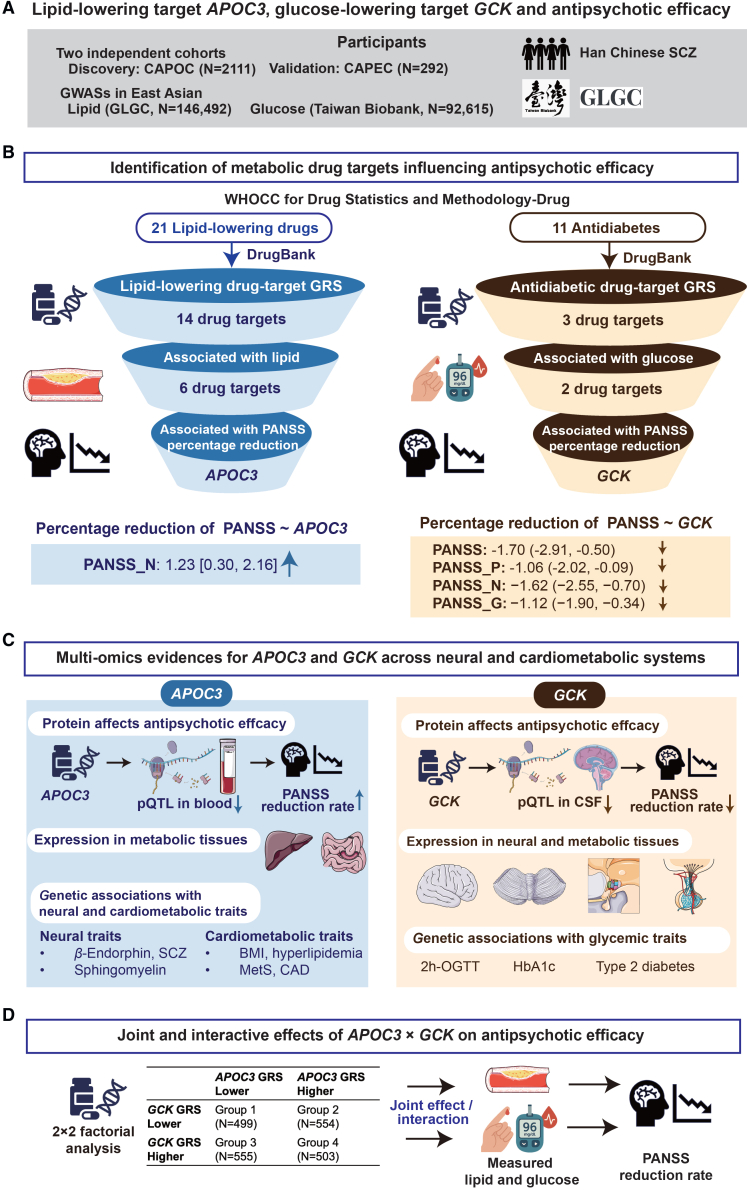
Study flowchart (A) Information of study participants. (B) Identification of lipid-lowering target *APOC3* and antidiabetic target *GCK* influencing antipsychotic efficacy. The effect size and 95% CI, expressed as beta (95% CI), represent the average percentage reduction in the PANSS for per standard deviation increase in the drug-target GRS. (C) Proteomic, gene-expression, and genetic-association evidences for *APOC3* and *GCK* across neural and cardiometabolic systems. (D) A framework of 2 × 2 factorial analysis estimating joint and interactive effects of *APOC3* and *GCK* on antipsychotic efficacy. Abbreviations: CAPOC, Chinese Antipsychotics Pharmacogenomics Consortium; CAPEC, Chinese Antipsychotics Pharmacogenetics Consortium; GLGC, Global Lipids Genetics Consortium; GWAS, genome-wide association study; *APOC3*, apolipoprotein C3; *GCK*, glucokinase; GRS, genetic risk score; PANSS, Positive and Negative Syndrome Scale; P, positive scale; N, negative scale; G, general psychopathology scale; pQTL, protein quantitative trait loci; CSF, cerebrospinal fluid; BMI, body mass index; 2h-OGTT, 2-hour oral glucose tolerance test; HbA1c, hemoglobin A1c; T2D, type 2 diabetes; SCZ, schizophrenia.

Based on two independent cohorts of participants with SCZ, we conducted a drug-target genetic association study, aimed to (1) estimate the causal effects of lipid lowering and antidiabetic targets on lipid and glucose levels, respectively, as well as their influences on the efficacy of antipsychotic treatments; (2) interpret the identified drug targets affecting antipsychotic efficacy from a multi-omics perspective across brain and cardiometabolic tissues; and (3) evaluate the potential joint and interactive effects of the identified lipid-lowering and antidiabetic targets on lipid and glucose, as well as an antipsychotic efficacy through a 2 × 2 factorial drug-target design (Figure 1).

## Results

### Participant characteristics

Our study followed the Strengthening the Reporting of Observational Studies in Epidemiology MR reporting guidelines (Table S1). The present discovery cohort (*N* = 2,111) was based on the Chinese Antipsychotics Pharmacogenomics Consortium (CAPOC) study, with antipsychotic efficacy assessed at week six. In the CAPOC study, the six-week total PANSS percentage reduction had a median (interquartile range [IQR], %) of 52.4 (29.2, 72.1) (Figure S1). Total cholesterol (TC), triglycerides (TG), and glucose levels (median [IQR], mg/dL) were 155.5 (135.3, 179.8), 88.6 (64.7, 126.7), and 85.8 (77.5, 93.9), respectively. All of the 14 genetic risk scores (GRSs) for lipid-lowering targets and three antidiabetic drug-target GRSs had *F* statistics larger than 10 (Tables S2, S3, S4, S5, S6, and S7). Participants with higher GRS of lipid-lowering target apolipoprotein C3 (*APOC3*, inhibited by volanesorsen/olezarsen) exhibited reduced concentrations of TC (*p* for trend = 0.005) and TG (*p* for trend <0.001). The *APOC3*-GRS accounted for 0.3% and 5.5% of the variances in TC and TG, respectively (Tables 1; Tables S2 and S8). Similarly, the group with higher GRS of glucose-lowering target glucokinase (*GCK*, activated by dorzagliatin) showed decreased glucose (*p* for trend = 0.017), with the *GCK*-GRS explaining 1.1% of the glucose variance (Tables 1, S2, and S9). No significant associations were observed between drug-target GRSs and other baseline characteristics (*p* for trend >0.05) (Tables 1 and S10).

**Table 1:** Baseline characteristics of participants in the CAPOC study

Baseline characteristics	All	*APOC3* GRS		*GCK* GRS	
		Lower	Higher	Lower	Higher
N (%)	2111	1054 (49.9)	1057 (50.1)	1053 (49.9)	1058 (51.1)

**Clinical Characteristics**					

Male (%)	1048 (49.6)	512 (48.6)	536 (50.7)	529 (50.2)	519 (49.1)
Female (%)	1063 (50.4)	542 (51.4)	521 (49.3)	524 (49.8)	539 (50.9)
Age (years)	30.0 (24.0, 38.0)	30.0 (24.0, 38.0)	30.0 (23.0, 38.0)	30.0 (24.0, 38.0)	30.0 (23.0, 38.0)
Center in the south (%)	858 (40.6)	417 (39.6)	441 (41.7)	405 (38.5)	453 (42.8)
Middle school or lower (%)	1194 (56.6)	591 (56.1)	603 (57.0)	581 (55.2)	613 (57.9)
Married (%)	692 (32.8)	349 (33.1)	343 (32.5)	333 (31.6)	359 (33.9)
First episode (%)	604 (28.6)	305 (28.9)	299 (28.3)	300 (28.5)	304 (28.7)
Course (years)	4.5 (2.0, 10.0)	4.7 (2.0, 10.0)	4.5 (1.8, 10.0)	5.0 (2.0, 10.0)	4.1 (2.0, 9.4)
Previous medication (%)	1107 (52.4)	538 (51.0)	569 (53.8)	566 (53.8)	541 (51.1)
Assigned medication with stronger metabolic side effects (%)	1056 (50.0)	511 (48.5)	545 (51.6)	517 (49.1)	539 (50.9)

**Physical Measurements**					

BMI (kg/m^2^)	21.7 (19.6, 24.2)	21.8 (19.8, 24.2)	21.6 (19.5, 24.2)	21.6 (19.6, 24.2)	21.8 (19.7, 24.2)
SBP (mmHg)	118 (110, 120)	118 (110, 120)	117 (110, 120)	118 (110, 120)	118 (110, 120)
DBP (mmHg)	76 (70, 80)	76 (70, 80)	76 (70, 80)	76 (70, 80)	75 (70, 80)

**Laboratory Measurements**					

TC (mg/dL)^a^	155.5 (135.3, 179.8)	158.0 (135.3, 181.0)	154.3 (135.0, 178.3)	154.7 (134.2, 178.7)	156.6 (135.7, 181.0)
TG (mg/dL)^a^	88.6 (64.7, 126.7)	93.9 (68.2, 133.7)	85.9 (62.0, 118.7)	88.6 (64.7, 126.7)	89.5 (64.7, 124.9)
HDLC (mg/dL)	49.9 (42.2, 59.2)	49.5 (42.2, 59.2)	50.3 (42.2, 59.2)	49.5 (42.2, 58.8)	50.3 (42.2, 60.3)
LDLC (mg/dL)	84.7 (67.3, 106.3)	85.8 (68.2, 107.5)	83.5 (66.5, 105.2)	85.1 (66.9, 106.0)	84.7 (68.1, 106.3)
Glucose (mg/dL)^a^	85.8 (77.5, 93.9)	85.2 (77.5, 93.7)	86.5 (77.5, 94.6)	86.5 (78.2, 94.8)	85.2 (77.3, 93.7)

Notes.

a Participants with increased *APOC3* GRS, predicting reduced concentrations of TC (*p* for trend = 0.005) and TG (*p* for trend <0.001). Similarly, the group with higher GRS of *GCK*, reflecting decreased glucose (*p* for trend = 0.017). The median and interquartile range (IQR) values were displayed for continuous variables. Abbreviations: BMI, body mass index; SBP, systolic blood pressure; DBP, diastolic blood pressure; LDLC, low-density lipoprotein cholesterol; HDLC, high-density lipoprotein cholesterol; TG, triglyceride; TC, total cholesterol. GRS, genetic risk score; CAPOC, Chinese Antipsychotics Pharmacogenomics Consortium study.

Our independent validation was derived from the Chinese Antipsychotics Pharmacogenetics Consortium (CAPEC) study (*N* = 292). By week eight, the CAPEC participants achieved a median 62.8% decrease in the total PANSS score, with an IQR of 47.0%–75.7% (Figure S1). The detailed characteristics of individuals were displayed in Table S11. The regression diagnostics did not reveal major violations of model assumptions or evidence of model instability for the main analyses (Tables S12 and S13).

### Associations between drug-target GRSs with lipids and glucose

**Figure 2:**
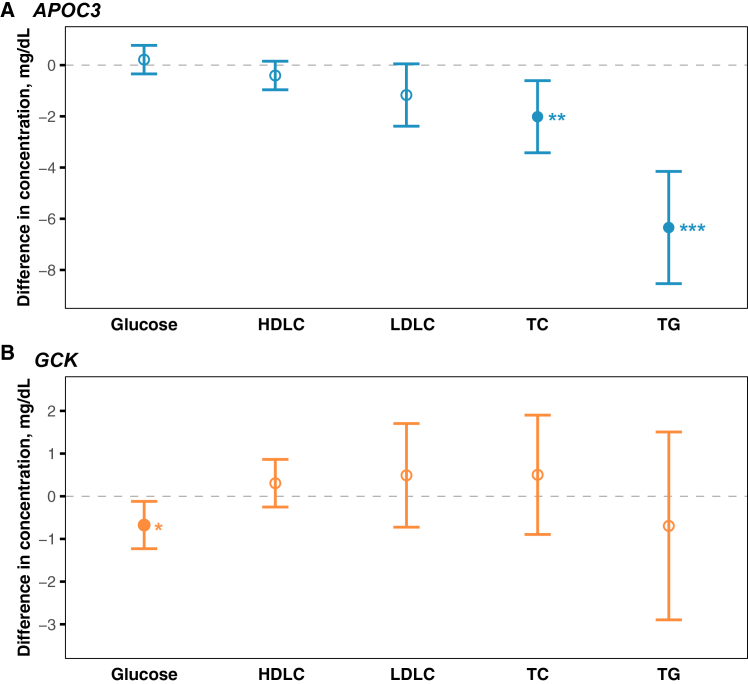
Associations of *APOC3*, *GCK* GRSs with the lipids and glucose (A) Associations of *APOC3* GRS with the lipids and glucose. (B) Associations of *GCK* GRS with the lipids and glucose. The beta (95% CI) represents the difference in the plasma lipids and glucose for per standard deviation increase in the drug-target GRS. Error bars indicate the 95% CIs. Nominal *p* values are provided. ^∗^*p* < 0.05, ^∗∗^*p* < 0.01, ^∗∗∗^*p* < 0.001. The corresponding result can be found in Tables S8 and S9. Abbreviations: *APOC3*, apolipoprotein C3; *GCK*, glucokinase; HDLC, high-density lipoprotein cholesterol; LDLC, low-density lipoprotein cholesterol; TC, total cholesterol; TG, triglyceride; GRS, genetic risk score.

Among the 14 lipid-modifying drug-target GRSs, one, four, one, and three GRSs were negatively associated with the measured low-density lipoprotein cholesterol (LDLC), high-density lipoprotein cholesterol (HDLC), TG, and TC, respectively (Benjamini-Hochberg false-discovery-rate-corrected *p* [P_FDR_] < 0.05). Two antidiabetic drug-target GRSs were associated with lower glucose levels (P_FDR_ < 0.05). Full results could be found in Tables S8 and S9. Individuals carrying higher GRS of *APOC3*, mimicking exposure to the lipid-modifying drug volanesorsen/olezarsen, exhibited attenuated levels of TG and TC, in turn. The corresponding differences were (*β*, 95% confidence intervals [95% CI], per standard deviations [SD] increase in GRS) −6.34 (−8.53, −4.15) mg/dL (P_FDR_ <0.001) and −2.02 (−3.42, −0.61) mg/dL (P_FDR_ = 0.025). Those with higher GRS of *GCK* (target of glucose-lowering drug dorzagliatin) demonstrated a reduction in the level of glucose (*β* [95% CI]: −0.68 [−1.23, −0.12], P_FDR_ = 0.034). Details were shown in Figure 2. Associations between drug-target GRSs and metabolic traits in the CAPEC study were in Table S14.

### Associations between drug-target GRSs and the PANSS percentage reduction

**Figure 3:**
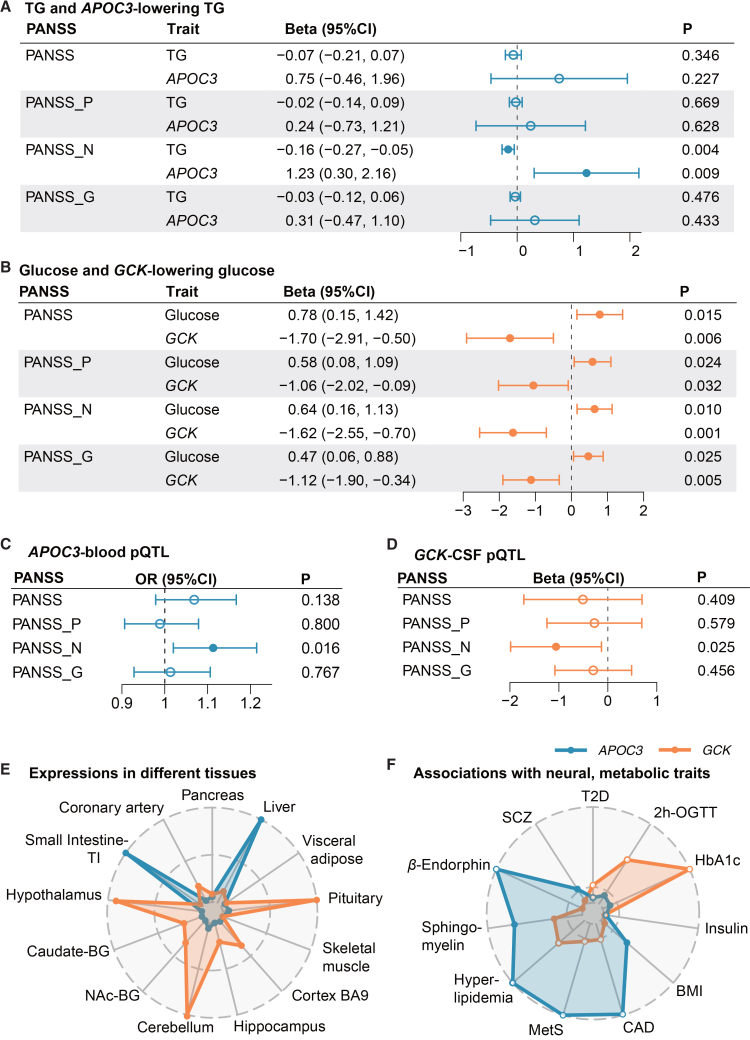
Associations between *APOC3*-lowering TG, *GCK*-lowering glucose, and PANSS percentage reduction and multi-omics evidence (A) Associations between the GRS of higher TG, *APOC3-*proxied lowering TG, and the percentage reduction of PANSS. (B) Associations between the GRS of higher glucose, *GCK*-proxied lowering glucose, and the percentage reduction of PANSS. (C and D) Associations between genetically predicted APOC3 protein in the blood and GCK protein in CSF on the reduction rate of PANSS. The beta (95% CI) represents the average percentage reduction in the PANSS for per standard deviation increase in the drug-target GRS. OR (95% CI) represents the probability of better antipsychotic efficacy (PANSS reduction rate > median value) for a standard deviation increase in the drug-target GRS. Error bars indicate the 95% CIs. Nominal *p* values are presented. The corresponding result can be found in Tables S35–S38. (E) Expression levels (transcripts per million) of *APOC3* and *GCK* in different tissues from the GTEx v.10. Full results are in Table S40. (F) Genetic associations between *APOC3*, *GCK*, and multiple neural, metabolic-related traits. For variants where the effect allele is associated with lower glucose or lipid levels, solid circles indicate positive associations with the traits, and open circles indicate negative associations. Full results are in Table S41. Abbreviations: *APOC3*, apolipoprotein C3; *GCK*, glucokinase; TG, triglyceride; PANSS, Positive and Negative Syndrome Scale; N, negative scale; G, general psychopathology scale; P, positive scale; CSF, cerebrospinal fluid; pQTL, protein quantitative trait loci; VAT, visceral adipose tissue; BA9, Brodmann area 9; NAc, nucleus accumbens; BG, basal ganglia; T2D, type 2 diabetes; BMI, body mass index; 2h-OGTT, 2-hour oral glucose tolerance test; HbA1c, hemoglobin A1c; CAD, coronary artery disease; MetS, metabolic syndrome; SCZ, schizophrenia; GRS, genetic risk score.

The single drug-target genetic association analysis demonstrated that the *APOC3*-proxied lowering TG and TC elevated the antipsychotic efficacy of the negative symptom. In the CAPOC study, genetically predicted higher levels of TG were significantly associated with 0.16% (0.05%, 0.27%) lower reduction rate of the negative scale of PANSS, as well as a lower probability of better antipsychotic efficacy (odds ratio [OR] [95% CI]: 0.98 [0.97, 0.99], P_FDR_ < 0.05) (Tables S15, S16, S17, and S18). An SD increase in the *APOC3* GRS—reflecting lower TG and TC—was associated with 1.23% (0.30%, 2.16%) and 0.96% (0.02%, 1.89%) greater PANSS negative subscale percentage reduction, respectively (nominal *p* < 0.05) (to convert to per 10 mg/dL increase in TG and TC, multiply by 1.58 and 4.95 correspondingly). *APOC3*-predicted TG lowering was significantly associated with better antipsychotic efficacy (OR [95% CI]: 1.16 [1.06, 1.26], P_FDR_ = 0.036). We did not observe associations between the *APOC3*-proxied lowering of TG and TC and the percentage reduction of total PANSS, as well as the positive and general psychopathology subscales. (Figures 3A, S19, and S20). Colocalization analysis at the *APOC3* locus supported a shared causal single-nucleotide polymorphism (SNP) between TG/TC and PANSS-negative percentage reduction. The top candidate shared variant was rs651821, with an H4 posterior probability (SNP.PP.H4) exceeding 0.6 (Table S21; Figure S2).

In contrast, the glucose reduction predicted by *GCK* diminished the therapeutic efficacy. Genetically predicted increased glucose levels were associated with greater PANSS reduction rates across total (*β* [95% CI]: 0.78 [0.15, 1.42]), positive (*β* [95% CI]: 0.58 [0.08, 1.09]), negative (*β* [95% CI]: 0.64 [0.16, 1.13]), and general psychopathology (*β* [95% CI]: 0.47 [0.06, 0.88]) subscales (P_FDR_ < 0.05) (Tables S22, S23, S24, and S25). The *GCK*-proxied attenuated level of glucose had a negative effect on the PANSS percentage reduction. This was reflected in the significantly diminished PANSS total and negative, general psychopathology reduction rates (P_FDR_ < 0.05), and a suggestively significant decrease in PANSS-positive percentage reduction (*p* = 0.032). The corresponding *β* [95% CI] were −1.70 (−2.91, −0.50), −1.62 (−2.55, −0.70), −1.12 (−1.90, −0.34), and −1.06 (−2.02, −0.09) (to convert to per 10 mg/dL increase in glucose, multiply by 14.71) (Figure 3B; Table S26). The associations were robust in the sensitivity analysis applying the dichotomized outcome of the antipsychotic treatment efficacy (Table S27). Furthermore, rs3757840 was revealed as the top shared SNP in the colocalization analysis at the *GCK* locus between measured glucose and the reduction rates across PANSS total and three subscales (SNP.PP.H4 > 0.6) (Table S21; Figure S2).

The causal effect of *GCK*-predicted glucose was validated in the independent CAPEC cohort. Among the CAPEC participants, *GCK* GRS showed negative effects on the reduction rates of total PANSS and negative subscale (P_FDR_ < 0.05). It was also inversely associated with the percentage reduction of the PANSS positive subscale at a nominal significance level (*p* = 0.046). See the Tables S28, S29, S30, and S31 for details. Two-sample Mendelian randomization analyses further supported these causal associations (Tables S32 and S33).

### Multi-omics evidence across neural and cardiometabolic systems supports the identified causal effects of *APOC3* and *GCK*

The causal effects of *APOC3* and *GCK* on the therapeutic outcomes were validated by analyses leveraging downstream molecular data across the circulatory and brain domains. Genetically predicted lowering of APOC3 protein concentrations in the blood was associated with a reduction in the TG (*β* [95% CI]: −6.01 [−8.20, −3.83]) and TC (*β* [95% CI]: −1.82 [−3.22, −0.43]) (P_FDR_ < 0.05). It also enhanced the probability of greater antipsychotic efficacy (OR [95% CI]: 1.11 [1.02, 1.22]) with suggestive significance (*p* = 0.016). The entire result was presented in Figure 3C and Tables S34–S36. In contrast, genetically predicted higher cerebrospinal fluid (CSF) GCK levels were associated with lowering glucose (*β* [95% CI]: −0.75 [−1.30, −0.19], P_FDR_ = 0.011). It was also associated with decreased negative symptom percentage reduction at nominal significance (*p* = 0.025). The corresponding *β* [95% CI] was −1.06 [−1.99, −0.13] (Figures 3D and S3; Tables S34, S37, and S38).

The mediation analysis showed that the effect of lowering APOC3 protein on better antipsychotic efficacy for negative symptoms was attributed to attenuated TG (OR [95% CI] of lowering TG conditioning on APOC3-protein quantitative trait loci (pQTL)-GRS: 1.30 [1.06, 1.60]). Little evidence showed the mediation effect of lowering TC (OR [95% CI]: 1.10 [0.96, 1.25]). Besides, the association between higher GCK in CSF and worse antipsychotic efficacy was mediated by decreased glucose (*β* [95% CI] of lowering glucose conditioning on GCK-pQTL-GRS: −1.43 [–2.43, −0.42]) (Table S39).

Notably, colocalization analysis again pinpointed rs651821 within *APOC3*—the SNP used to construct APOC3-pQTL-GRS—as the top shared variant between circulating APOC3 protein and negative-symptom efficacy (SNP.PP.H4 = 0.715). Likewise, rs3757840 in *GCK*, which was employed in the GCK-pQTL-GRS, emerged as the leading shared signal between the CSF GCK protein and percentage reduction of PANSS negative subscale (SNP.PP.H4 = 0.689) (Table S21; Figure S2).

Leveraging the gene expression profiles in different tissues, we captured that *APOC3* exhibited higher levels in the lipid-metabolic systems, involving the liver and small intestine. *GCK*, on the other hand, was predominantly expressed in the brain regions responsible for homeostatic and cognitive functions, including the hypothalamus, pituitary, and cortex. Given GCK’s established roles in pancreatic and hepatic glucose metabolism, we compared its expression between these two tissues. We observed that *GCK* exhibited higher expression in the liver (transcripts per million [TPM] = 0.857) than its expression levels in the pancreas (TPM = 0.511) (Figure 3E; Table S40).

Interrogation of GWAS datasets further indicated that *APOC3* was associated with neuropeptide and membrane lipid composition, including *β*-endorphin and sphingomyelin. It was also associated with the susceptibility to metabolic syndrome (MetS), coronary artery disease, and SCZ (all *p* < 0.05). The *GCK* displayed stronger associations with the glycemic metabolic indicators, involving the 2-hour oral glucose tolerance test, hemoglobin A1c, and type 2 diabetes (all *p* < 0.05). Detailed results could be found in Figures 3F and S41. Based on individuals from the CAPOC study, we observed causal associations between drug-target *APOC3* and the level of aspartate aminotransferase, a typical enzyme reflecting liver function (*β* [95% CI]: 0.82 [0.07, 1.56]) (Table S42).

### Subgroup analysis

**Figure 4:**
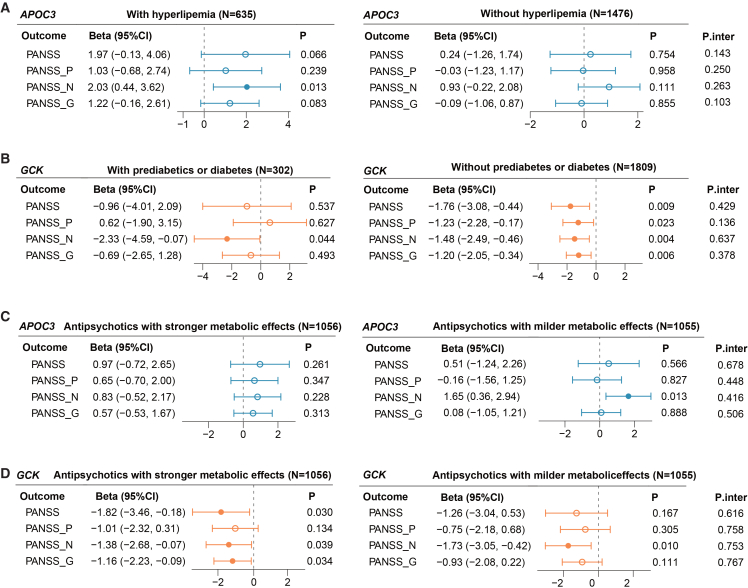
Stratified analysis by the baseline metabolic statuses and the assigned types of antipsychotics (A and B) Stratified analysis by the baseline metabolic status. Patients with baseline TG ≥ 151 mg/dL (1.70 mmol/L) or baseline HDL ≤ 40 mg/dL (1.04 mmol/L) were categorized in the hyperlipemia group (*N* = 635); others were in the non-hyperlipemia group (*N* = 1,476). Patients with baseline glucose ≥ 100.8 mg/dL (5.6 mmol/L) were classified into the prediabetes or diabetes group (*N* = 302); the remaining patients were in the non-diabetes or prediabetes group (*N* = 1,809). (C and D) Stratified analysis by the assigned types of antipsychotics. Patients prescribed risperidone, olanzapine, or quetiapine were grouped as receiving medication with stronger metabolic side effects (*N* = 1056), while others (prescribed aripiprazole, ziprasidone, perphenazine, or haloperidol) comprised the milder-metabolic-effect group (*N* = 1055). Full results are in Tables S43 and S44. The beta (95% CI) represents the average percentage reduction in the PANSS for per standard deviation increase in the drug-target GRS. Error bars indicate the 95% CIs. Nominal *p* values are presented. P.inter represented the interaction test between the stratified factor and drug-target GRS. Abbreviations: *APOC3*, apolipoprotein C3; *GCK*, glucokinase; TG, triglyceride; PANSS, Positive and Negative Syndrome Scale; N, negative scale; G, general psychopathology scale; P, positive scale; GRS, genetic risk score.

The beneficial effect of *APOC3*-predicted TG lowering on negative symptom recovery was robust in participants with hyperlipemia (*n* = 635, *β* [95% CI]: 2.03 [0.44, 3.62], nominal *p* = 0.013). The detrimental effect of *GCK*-predicted glucose reduction on negative symptom recovery was consistent among those with prediabetes or diabetes (*n* = 302, *β* [95% CI]: −2.33 [−4.59, −0.07], nominal *p* = 0.044). Significant causal associations of *GCK*-proxied glucose with percentage reductions of PANSS total, positive, negative, and general psychopathology subscales were observed among individuals free of glucose dysfunction (*n* = 1,809, all P_FDR_ < 0.05) (Figures 4A and 4B; Tables S43 and S44).

When stratified by antipsychotic class, suggestive associations of *GCK*-predicted glucose were observed in patients receiving antipsychotics with stronger metabolic effect (*n* = 1,056). These associations spanned PANSS total (*β* [95% CI]: −1.82 [−3.46, −0.18]), negative (*β* [95% CI]: −1.38 [−2.68, −0.07]), and general psychopathology scales (*β* [95% CI]: −1.16 [−2.23, −0.09]). The causal effect of *APOC3*-predicted TG on the negative symptom relief was found in those using drugs with milder effects (*n* = 1,055, *β* [95% CI]: 1.65 [0.36, 2.94]) (all nominal *p* < 0.05, Figures 4C and 4D; Tables S43 and S44).

Among the participants with relapsed SCZ (*n* = 1713), *APOC3*-mediated TG reduction was positively associated with the negative-symptom improvement (*β* [95% CI]: 1.40 [0.38, 2.41], nominal *p* = 0.007). Glycemic lowering index by the *GCK* aligned with a weaker therapeutic efficacy, including lower reduction rates in total, negative, and general psychopathology PANSS scores (P_FDR_ < 0.05). *GCK*-related glucose lowering attenuated the percentage reduction of PANSS negative scales among males (*n* = 1048, P_FDR_ = 0.008). In females, it was associated with reduced percentage reduction in the total and general psychopathology scales (*n* = 1,063, nominal *p* < 0.05) (Figure S4; Tables S43 and S44). No significant interactive effects between the drug-target GRSs and the subgroup factors were observed (P for interaction >0.05) (Figures 4 and S4; Tables S43 and S44). Subgroup analysis in the CAPEC study was shown in Tables S45 and S46.

### Joint and interaction associations between *APOC3*, *GCK*, and antipsychotic efficacy

Compared to the group with relatively lower GRSs of both targets (*n* = 498), participants with both elevated GRSs (*n* = 502) showed a greater reduction in measured TG. The respective *β* [95% CI] was −11.23 [−17.63, −4.82]. Evaluating the product of the continuous GRSs of *APOC3* and *GCK*, we did not observe the nonadditive interaction effect on the lipids (*p* > 0.05) (Figure S5; Tables S47 and S49).

Relative to individuals with low GRSs for both targets, those with an elevated GRS of *GCK* but an attenuated *APOC3* GRS (*n* = 556)—suggesting lower glycemia and higher lipid levels—experienced poorer antipsychotic efficacy. This was notable for total PANSS and the negative scale of PANSS, with *β* [95% CI] estimates of −3.46 (−6.89, −0.04) and −3.08 (−5.71, −0.44), showing suggestive significance (*p* < 0.05) (Tables S50 and S51). However, we observed no significant combined effect of the two targets on PANSS percentage reduction and no evidence of interaction (*p* > 0.05) (Figure S6; Tables S52 and S53).

## Discussion

We performed a drug-target genetic association study to assess the independent and combined causal effects of lipid- and glucose-lowering drug targets on antipsychotic treatment efficacy among individuals with SCZ. We identified that *APOC3*-proxied lowering of TG and TC could enhance the improvement of negative symptoms, whereas *GCK* was associated with poor treatment outcomes, through the attenuated glucose concentration. By leveraging multi-omics data in the brain and cardiometabolic systems, we validated the influences of *APOC3* and *GCK* on the efficacy of antipsychotics. The further colocalization analysis strengthened their on-target effects. The *APOC3* and *GCK* jointly reduced TG, without a combined impact on antipsychotic efficacy. Our study provided genetic evidence for the potential benefits and pharmacovigilance considerations of cardiometabolic agents on antipsychotic efficacy.

APOC3 plays a key role in TG metabolism. In the present study, it showed higher expression in digestive tissues, particularly the liver and small intestine. Our findings were similar to previous mechanism studies, which reported that APOC3 could not only inhibit the lipoprotein lipase and block the hepatic uptake of TG-rich lipoprotein remnants^19^ but also modulate chylomicron formation and intestinal secretion of dietary TG.^20^ The APOC3 is the primary inhibitory target of volanesorsen and olezarsen, two distinct antisense oligonucleotides targeting elevated TG in chylomicronemia.^21^^,^^22^^,^^23^ Compared with the healthy controls, *APOC3* expression is downregulated in patients with SCZ, suggesting its diagnostic relevance for the disorder.^24^ Notably, among those affected by SCZ, our findings were aligned with previous genetic association studies. Two of the studies reported that the genetic variations of *APOC3* were associated with attenuated serum TG,^25^ as well as the risk of MetS.^26^

Focusing on the impact of APOC3 on the progression of SCZ, a recent meta-analysis involving nine RCTs (533 patients with SCZ) indicated that adjunctive statin therapy might benefit the reduction rates of PANSS negative and total scores.^11^ This is consistent with our findings demonstrating a protective effect of *APOC3*-proxied lipid lowering. From a peripheral perspective, a previous study (*N* = 80) showed that the polymorphisms in *APOC3* were related to the olanzapine pharmacokinetic variability.^27^ Given the lipophilic nature of olanzapine, *APOC3*-mediated TG transport may influence its pharmacokinetic distribution. Moreover, inhibition of APOC3 in the liver and small intestine, with consequent reductions in remnant lipid particles and microvascular inflammatory injury,^28^^,^^29^ could improve blood-brain barrier integrity and the central availability of antipsychotic drugs and nutrients.^30^^,^^31^ In addition, lowering TG in blood helps normalize abnormal inflammatory biomarkers—such as C-reactive protein^32^ and circulating cytokines^33^—and reduce oxidative stress.^34^ It may also modulate the neurochemical mediators (e.g., leptin),^34^ thereby facilitating the central nervous system recovery and functional improvement in SCZ. At the central level, we observed a significant association between *APOC3* and *β*-endorphin, and higher expressions of *APOC3* in the hypothalamus and cortex. Our findings imply that APOC3 might contribute to neuroregulatory “lipid-opioid-dopamine” networks with potential impact on negative symptom recovery.^35^^,^^36^ Besides, a previous study indicated that APOC3 might function as a hypothalamic lipid-sensing modulator.^37^ The APOC3-inhibition-driven relief of peripheral metabolic stress could lessen the metabolic strain on central energy-reward circuits.^38^ This, in turn, could support the remodeling of dopamine- and emotion-related networks relevant to antipsychotic response.^39^

The glucokinase is a hexokinase-family enzyme that facilitates the conversion of glucose to glucose-6-phosphate, the initiating step in most glucose-metabolic pathways. In the present study, *GCK* was expressed more abundantly in hepatic tissue than in the pancreas, paralleling prior *in vivo* evidence. A liver-targeted *GCK* agonist reduced glycemia without raising the circulating insulin,^40^ whereas overexpressed hepatic *GCK* accelerated glucose clearance in insulin-null type 1 diabetic mice.^41^ These findings underscored an insulin-independent role for glucokinase in liver glucose homeostasis. The glucokinase activator—dorzagliatin—boosts glucose sensitivity and helps maintain glucose homeostasis in individuals with type 2 diabetes.^42^

Our findings indicated that the *GCK*-induced glucose lowering attenuated the antipsychotic efficacy. Consistent with our results, a previous clinical study (64 patients with SCZ, 33 healthy controls) reported that the dysfunction of the glucokinase/glucose-6-phosphate metabolism pathway could lead to cytotoxicity in neurons and cognitive impairment in SCZ.^43^ We detected dominant expression of *GCK* within hypothalamic and cortical regions, consistent with its role as a neuronal glucose sensor in the hypothalamus.^44^^,^^45^ *GCK* gain-of-function variants may both chronically lower peripheral glucose levels and lower the glycemic threshold at which central glucose-sensing neurons initiate counterregulatory responses.^44^ This may increase the risk of severe hypoglycaemia^46^ and predispose the brain to recurrent neuroglycopenic episodes (brain energy insufficiency).^46^^,^^47^ These episodes can lead to oxidative and inflammatory stress^46^ and neurotoxic injury^48^ in hippocampal,^49^ prefrontal,^50^ and striatal circuits,^51^ potentially undermining antipsychotic efficacy.^46^^,^^47^ In parallel, hypothalamic GCK-positive neurons interface with autonomic, HPA,^52^^,^^53^ and reward-related networks that regulate mesolimbic dopamine.^54^^,^^55^ Recurrent low-glucose states may perturb reward dopamine signaling and hinder antipsychotic efficacy.^56^ In the peripheral system, GCK functions as a glucose-sensing enzyme in the liver.^40^^,^^57^ Accordingly, genetically predicted increases in GCK activity enhance hepatic glucose uptake and disposal and predispose individuals to recurrent hypoglycemic episodes. This could contribute to neuroglycopenia and other central disturbances, fostering unfavorable neurobiological conditions for SCZ recovery.^46^^,^^47^

From a translational perspective, our findings provide genetic support for APOC3 inhibition (volanesorsen and olezarsen) as a candidate pathway that may beneficially modulate antipsychotic efficacy. The clinical reliability of this finding warrants further consideration. Volanesorsen and olezarsen are administered subcutaneously to lower APOC3 and TG. Both drugs have undergone phase 3 evaluation in familial chylomicronemia syndrome (FCS),^21^^,^^23^ and olezarsen is approved in America and Europe as a diet adjunct in adults with FCS.^58^ Our genetic analyses indicated that *APOC3*-proxied TG lowering did not materially affect common safety indicators, including blood pressure, QTc interval, renal and hepatic indices, and prolactin levels. Such lines of evidence collectively support the clinical safety of these two drugs. However, the potential adverse effects of these drugs (e.g., thrombocytopenia)^21^^,^^23^ and the pleiotropic effects of APOC3 in the inflammatory and endothelial pathways^28^ have not been systematically evaluated in people with SCZ. On the other hand, although these two drugs lower TG predominantly in the liver and have limited brain penetration, APOC3-inhibition-related TG lowering may still influence antipsychotic response through downstream metabolic and neurovascular pathways. It may modify the peripheral distribution of lipophilic antipsychotics^27^ and attenuate systemic and cerebrovascular inflammation.^33^ Such changes could help preserve the blood-brain barrier integrity,^30^ increase central antipsychotic availability, and ultimately facilitate recovery in SCZ. Our observation of *APOC3* expression in the brain and its association with *β*-endorphin provides supportive evidence that APOC3 may interface with opioid-related neuroregulatory circuits^35^^,^^36^ and central lipid-sensing pathways,^37^ contributing to central nervous system improvement. Future proof-of-concept RCTs in SCZ populations with comorbid hypertriglyceridemia are required to assess the feasibility, safety, and potential clinical benefit of APOC3-targeted interventions, incorporating detailed monitoring of psychiatric outcomes (particularly negative symptoms) to improve the current prescription guidelines. By contrast, our *GCK*-related glucose-lowering findings call for clinical vigilance, and any future use of dorzagliatin in SCZ populations should pay particular attention to potential adverse effects on antipsychotic response.

The drug-target genetic association strategy, which applies genetic variants within the ±100 kb region of each target, could simulate the on-target effect of the corresponding drugs. This approach can also minimize the risk of inducing horizontal pleiotropy due to the mixed variants from genes nearby.^59^ Our colocalization analysis showed that both *APOC3*- and *GCK*-linked antipsychotic efficacy signals shared causal variants with their respective pQTL and with lipids or glucose. The mediation analysis indicated that such effects of these target proteins on SCZ recovery were attributed to the lowering of TG or glucose. Together, these investigations further support predominantly on-target causal effects of *APOC3*- and *GCK*-related lipid and glucose lowering on antipsychotic efficacy. The 2 × 2 factorial drug-target framework further mimics simultaneous interventions of lipid- and glucose-modifying drugs, allowing us to evaluate their combined effect.^18^

Our stratified analysis implied that, among patients complicated with lipid dysfunction, *APOC3* could be considered as a lipid-lowering target that may enhance negative-symptom improvement. In contrast, *GCK* emerged as a cautionary glucose-modifying target, as it was associated with worse improvement in negative symptoms, among patients with prediabetes or diabetes. Nevertheless, several antipsychotics, such as olanzapine, are known for their common metabolic side effects.^60^ Whether glucose-lowering agents should be co-administered to prevent antipsychotic-induced metabolic disturbances remains an active area of investigation.^61^^,^^62^ Our results raise concerns for using GCK-targeting glucose-lowering drugs as adjunctive treatments, since they may exacerbate psychotic symptoms when co-prescribed with high-metabolic-risk antipsychotics. Besides, the lipid-lowering effect of *APOC3* appeared especially advantageous in patients taking agents with minimal metabolic liabilities.

In summary, based on large-scale longitudinal data of Han Chinese patients with SCZ, we assessed both the individual and combined associations of the metabolism-modifying agents on 6-week antipsychotic treatment efficacy. Validation across two separate cohorts underscored the robustness of our major findings. We revealed that the *APOC3*-proxied lipid-lowering levels might enhance the efficacy of antipsychotic treatments in SCZ, whereas the attenuated glucose related to the *GCK* variants could elevate the risk of worsening therapeutic outcomes. Our findings suggested volanesorsen and olezarsen as potential lipid-modifying adjunctive candidates, while the glucose-lowering drug—dorzagliatin—warrants clinical prudence in managing patients with concurrent psychiatric and metabolic disturbance. The genetic insights from this study pave the way for tailored interventions and therapeutic targets in clinical psychiatry.

### Limitations of the study

We acknowledged limitations for further discussion. First, genetic instruments represent lifelong exposure to agents, which differs from short-term randomized pharmacologic trials. In addition, real-world compounds might have off-target effects and yield outcomes that diverge from present findings, which need to be clarified by future clinical studies. And residual confounding cannot be fully excluded. Second, due to limited genome-wide significant pQTLs in East Asians, we selected lipid- and glucose-associated SNPs within target loci; results remained consistent in sensitivity analysis using pQTLs meeting *p* < 0.005. Currently available brain-based pQTL datasets are derived exclusively from Europeans, despite similar allele frequencies across ancestries,^63^ East Asian resources are warranted to validate these findings. Because our study was conducted in East Asians, its generalizability to other ancestries remains uncertain. Extensions in diverse ancestries are required to replicate our findings and explore potential ancestry-specific effects. Third, drug-target MR aims to prioritize targets that might influence SCZ progression and inform further clinical investigations.^64^^,^^65^ Focusing solely on associations surviving multiple testing might overlook some biologically meaningful targets. Given our limited sample size—particularly in the prediabetes/diabetes subgroup—relative to prior drug-target MR studies,^66^^,^^67^ we also considered nominal results and interpreted results in the context of effect sizes and 95% CI.^68^^,^^69^

## Resource availability

### Lead contact

Further information and requests for resources should be directed to and will be fulfilled by the lead contact, Weihua Yue (dryue@bjmu.edu.cn).

### Materials availability

This study did not generate unique reagents.

### Data and code availability

• The GWAS and expression quantitative trait loci (eQTL) summary statistics were publicly available. GWAS summary statistics from the Biobank of Japan could be obtained from the corresponding websites (https://pheweb.jp/). GWAS summary statistics for lipids were obtained from the Global Lipids Genetics Consortium (https://csg.sph.umich.edu/willer/public/glgc-lipids2021/results/ancestry_specific/). GWAS summary statistics for type 2 diabetes were from the Type 2 Diabetes Global Genetics Initiative (https://diagram-consortium.org/downloads.html). Other GWAS summary statistics were downloaded from the GWAS Catalog (https://www.ebi.ac.uk/gwas/). The eQTL data from the Genotype Tissue Expression (GTEx) were obtained from the corresponding portals (https://gtexportal.org/home/downloads/). The blood-pQTL summary statistics from the Guangzhou Nutrition and Health study were obtained from the website https://omics.lab.westlake.edu.cn/data/proteins/phenotypes. The CSF-pQTL was downloaded from the https://www.researchsquare.com/article/rs-2814616/v1.
• Constrained by the local law on the management of human genetic resources and the requirements of the research project, the sharing of individual-level data of the Chinese Antipsychotics Pharmacogenomics Consortium study and the Chinese Antipsychotics Pharmacogenetics Consortium was restricted from public access. The data that support the findings of this study are available from the lead contact, upon reasonable request with a proposal. All requests must be approved by the relevant ethics boards and data custodians.
• No custom code is reported in this study. Any additional information required to reanalyze the data reported in this work paper is available from the lead contact upon request.

## Acknowledgments

We want to acknowledge the participants and investigators of the Chinese Antipsychotics Pharmacogenomics Consortium study and Chinese Antipsychotics Pharmacogenetics Consortium, Global Lipids Genetics Consortium, Taiwan Biobank, Biobank of Japan, Type 2 Diabetes Global Genetics Initiative, Guangzhou Nutrition and Health study, Million Veteran Program, UK Biobank, Meta-Analysis of Glucose and Insulin-related Traits Consortium, The Qatar Genome Program Research Consortium, the Adult Genotype Tissue Expression Project, as well as the genome-wide association study conducted by Chen et al., Zhen et al., Park et al., and Cruchaga et al.

This study was supported by the National Key R&D Program of China (2025YFC2511200), 10.13039/501100001809National Natural Science Foundation of China (82330042, 82441005, 82301687, 82501828, 82501802), 10.13039/501100012166National Key R&D Program of China (2023YFE0119400, 2021YFF1201100), Capital's Funds for Health Improvement and Research (2024-1-4111), Fundamental Research Funds for the Central Universities (10.13039/501100007937Peking University Medicine Fund for world’s leading discipline and discipline cluster development, BMU2022DJXK007), 10.13039/501100005088Beijing Municipal Health Commission Research Ward Programme (3rd batch), Non-profit 10.13039/501100022599Central Research Institute Fund of 10.13039/501100005150Chinese Academy of Medical Sciences (2023-PT320-08), 10.13039/501100005090Beijing Nova Program (20230484425), Youth Talent Support Program of the 10.13039/100010097China Association for Science and Technology, Beijing Natural Science Foundation (7254462), and 10.13039/501100017003Peking University Health Science Center Outstanding Doctoral Student Innovation Fund (BMU2025BSS0011).

## Author contributions

W.Y. is the primary investigator. W.Y. and Y.S. designed the study, acquired funding, and contributed to interpretation of data. Y. Zhu and R.Y. analyzed the data. Y. Zhu wrote the first draft of the article. Z.L., Y. Zhang, Z.K., X.F., G.Z., J.S., J.G., T.Y., and Y.Y. were responsible for phenotype cleaning, preparation of the tables and figures, and providing further data interpretation. W.Y. and Y.S. gave interpretation for the study results and the development of the study conclusion. All authors contributed to drafting the work or critically revising it for important intellectual content and made substantial contributions to the concept and design of the study and data acquisition, analysis, and interpretation.

## Declaration of interests

The authors declare no competing interests.

## Declaration of generative AI and AI-assisted technologies in the writing process

During the preparation of this work, the authors used ChatGPT in order to improve language and readability. After using this tool/service, the authors reviewed and edited the content as needed and take full responsibility for the content of the publication.

## STAR★Methods

### Key resources table

**Table:** 

REAGENT or RESOURCE	SOURCE	IDENTIFIER
**Deposited data**		

GWAS summary statistics of lipids	Global Lipids Genetics Consortium^70^	https://csg.sph.umich.edu/willer/public/glgc-lipids2021/results/ancestry_specific/
GWAS summary statistics of glucose	Taiwan Biobank^71^	http://ftp.ebi.ac.uk/pub/databases/gwas/summary_statistics/GCST90278001-GCST90279000/GCST90278628
GWAS summary statistics of Hemoglobin A1c levels	Taiwan Biobank^71^	http://ftp.ebi.ac.uk/pub/databases/gwas/summary_statistics/GCST90278001-GCST90279000/GCST90278632
GWAS summary statistics of systolic blood pressure	Biobank of Japan^72^	https://pheweb.jp/pheno/SBP
GWAS summary statistics of schizophrenia	Biobank of Japan^72^	https://pheweb.jp/pheno/Schizophrenia
GWAS summary statistics of coronary artery disease	Biobank of Japan^73^	https://pheweb.jp/pheno/CAD
GWAS summary statistics of body mass index	Biobank of Japan^74^	https://pheweb.jp/pheno/BMI
GWAS summary statistics of sphingomyelin levels	UK Biobank^75^	http://ftp.ebi.ac.uk/pub/databases/gwas/summary_statistics/GCST90092001-GCST90093000/GCST90092982
GWAS summary statistics of type 2 diabetes	Type 2 Diabetes Global Genetics Initative^76^	https://diagram-consortium.org/downloads.html
pQTL summary statistics in the blood	Guangzhou Nutrition and Health study^77^	https://omics.lab.westlake.edu.cn/data/proteins/phenotypes
GWAS summary statistics of hyperlipidemia	Million Veteran Program^78^	http://ftp.ebi.ac.uk/pub/databases/gwas/summary_statistics/GCST90475001-GCST90476000/GCST90475718
GWAS summary statistics of beta-endorphin levels	The Qatar Genome Program Research Consortium^35^	http://ftp.ebi.ac.uk/pub/databases/gwas/summary_statistics/GCST90161001-GCST90162000/GCST90161374
eQTL summary statistics	The Adult Genotype Tissue Expression Project v10^79^	https://gtexportal.org/home/aboutAdultGtex
GWAS summary statistics of fasting insulin	Chen et al.^80^	http://ftp.ebi.ac.uk/pub/databases/gwas/summary_statistics/GCST90002001-GCST90003000/GCST90002237
GWAS summary statistics of oral glucose tolerance test-2h	Zhen et al.^81^	http://ftp.ebi.ac.uk/pub/databases/gwas/summary_statistics/GCST90297001-GCST90298000/GCST90297790
GWAS summary statistics of metabolic syndrome	Park et al.^82^	http://ftp.ebi.ac.uk/pub/databases/gwas/summary_statistics/GCST90444001-GCST90445000/GCST90444489
pQTL summary statistics in the cerebrospinal fluid	Cruchaga et al.^83^	https://neurogenomics.wustl.edu/open-science/raw-data/
Chinese Antipsychotics Pharmacogenomics Consortium - Individual-level phenotype and genotype data	Yu et al.^84^	N/A
Chinese Antipsychotics Pharmacogenetics Consortium - Individual-level phenotype and genotype data	Yu et al.^84^	N/A

**Software and algorithms**		

R 4.1.3	Open-source	https://cran.r-project.org/
PLINK v1.9	Purcell et al.^85^	https://www.cog-genomics.org/plink2/
IMPUTE v2	Bryan et al.^86^	https://mathgen.stats.ox.ac.uk/impute/impute_v2.html
SHAPEIT v2	Delaneau et al.^87^	https://mathgen.stats.ox.ac.uk/genetics_software/shapeit/shapeit.html

### Experimental model and study participant details

We applied two non-overlapping, clinically ascertained cohorts of individuals with SCZ. The discovery cohort was based on the CAPOC study, conducted in 2010, across five research centers in China, involving a total of 3030 participants.^84^ The eligible patients provided their sociodemographic and clinical questionnaire information, physical measurements, and blood samples upon the baseline assessment. Individuals were assigned to six groups of antipsychotics (aripiprazole, olanzapine, quetiapine, risperidone, ziprasidone, or one of the first-generation antipsychotics [haloperidol or perphenazine]) for six weeks. An independent validation cohort was from the CAPEC study. Patients were enrolled from two study centers in China (*N* = 568) in 2009–2010 and received 8 weeks of treatment with olanzapine, aripiprazole, risperidone, quetiapine, clozapine, ziprasidone, or perphenazine.^84^^,^^88^

The CAPOC and CAPEC studies included Han Chinese participants, aged 18 to 45 years, diagnosed with SCZ based on the Diagnostic and Statistical Manual of Mental Disorders - Fourth Edition (DSM-IV) in the acute phase. Those who had severe, unstable physical diseases, such as hypertension, myocardial infarction, or other cardiac diseases; those who had QTc prolongation or a history of congenital QTc prolongation were excluded.

We further excluded participants without qualified genotype data (CAPOC: *n* = 474, CAPEC: *n* = 265), those with missing values or outliers (>3 SD, CAPOC: *n* = 442, CAPEC: *n* = 11), and those who reported using anti-cardiovascular disease drugs (CAPOC: *n* = 3, CAPEC: *n* = 3), leaving 2,111 and 292 participants for the discovery and validation cohorts, respectively (Figure S7). Among the participants from the CAPOC study, the median (IQR) age was 30.0 (24.0–38.0) years, and 1,048 (49.6%) were male. In the CAPEC cohort, the median (IQR) age was 32.0 (23.0–40.0) years, with males accounting for 45.2% of participants. A summary of the multi-omics dataset applied for the present study was shown in Table S54.

The CAPOC study was approved by the ethics committee of the Peking University Sixth Hospital and each participating site. The reference number was 2009-LUNSHEN-23, and the approval date was April 21st, 2009. This study was registered at the Chinese Clinical Trial Registry (ChiCTR-TRC-10000934). The CAPEC study was approved by the ethics committee of each participating site, registered at the Chinese Clinical Trial Registry (ChiCTR-RNC-09000522). For both studies, informed written consent was obtained from all included participants in accordance with the Declaration of Helsinki. All participants were asked to appoint a family member or close friend involved in the informed consent discussion and help the patients with decision-making.

For the publicly available GWAS, expression, and protein quantitative trait loci summary statistics, all contributing studies were conducted in accordance with the ethical standards of the relevant national and institutional committees on human experimentation and the Helsinki Declaration. All study protocols were approved by the institutional ethics review boards at each site, and written or verbal informed consent was obtained from all participants.

### Method details

#### Genotyping and quality control

A total of 900,015 SNPs in the CAPOC study and 487,774 SNPs in the CAPEC study were genotyped using the Illumina Human Omni ZhongHua8 Beadchips (Illumina, San Diego, CA, USA) designed for the Chinese. Genotype quality control was conducted using the following exclusion criteria: (1) duplicated SNPs, (2) call rate <0.05, (3) minor allele frequency (MAF) < 0.05, (4) Hardy-Weinberg P (P_HW_) < 10^−6^. The imputation was performed using IMPUTE (Version 2), taking the 1000 Genomes Project-East Asian population (1000G-EAS)^63^ as the reference. A total of 11,986,772 SNPs from the CAPOC study and 10,732,149 SNPs from the CAPEC study passed the imputation quality threshold (info score >0.6).^84^^,^^89^

Among the 3030 participants of CAPOC study, 474 participants without qualified genotype data were excluded, and 11 out of 303 participants in the CAPEC study were also excluded following the exclusive criteria: (1) call rate <0.05, (2) sex discrepancy, (3) relatedness (proportion identical-by-descent <0.2); (4) heterozygosity (> three standard deviations); (5) principal components outliers. Qualified samples were phased using SHAPEIT (Version 2).

The present study additionally performed the genomic quality control for the imputed SNPs. The SNPs that met any of the criteria were excluded: (1) call rate <0.02, (2) MAF <0.01, (3) P_HW_ < 10^−6^. For the CAPOC study, a total of 4,995,111 SNPs and 2,111 participants were included in the analysis; whereas 2,508,713 SNPs and 292 participants from the CAPEC study were included for the present analysis (Figure S7).

#### Drug-target GRS

Referring to the World Health Organization Collaborating Center for Drug Statistics Methodology and DrugBank platform,^90^ we selected 14 genes encoding pharmacological targets of lipid-lowering drugs and three targets of antidiabetic drugs (Table S55). Drug-target GRSs were constructed by extracting significant genetic associations (*p* < 5 × 10^−8^) with lipids and glucose concentrations from the Global Lipids Genetics Consortium (GLGC, *N* = 146,492, East Asian)^70^ and Taiwan Biobank (*N* = 92,615, East Asian).^71^ We restricted the independent (r^2^ < 0.1) common variants (MAF >0.005) within the 100 kb region around the target genes (Table S2). The weighted drug-target GRSs were constructed by summing the number of effect alleles weighted by the effect size at each SNP. The effect sizes were obtained from corresponding GWASs (GLGC for lipid and Taiwan Biobank for glucose) and harmonized to ensure that all SNP effects reflected decreases in the relevant lipids or glucose.^91^^,^^92^

In addition, we calculated traditional (genome-wide) GRSs for lipids and glucose. The significant, independent, common genetic associations (*p* < 5 × 10^−8^, r^2^ < 0.001, MAF >0.005) with lipids and glucose were extracted from GLGC and Taiwan Biobank (Tables S3, S4, and S5). The GRSs were calculated by combining all variants for each target, weighed by effect sizes corresponding to higher lipid or glucose levels.^91^^,^^92^

To evaluate the strength of these GRSs, we calculated the proportion of variance explained (*R*^*2*^) by the selected SNPs using the formula: *R*^*2*^ = Σ*β*^2^ × 2 × MAF × (1 − MAF), where *β* denotes the effect size. We further assessed instrument strength using the *F*-statistic, computed as *F* = [(*N* − *K* − 1)/*K*] × *R*^*2*^/(1 − *R*^*2*^), where *N* was the sample size and *K* was the number of SNPs included in the GRS.^67^^,^^91^^,^^92^ When allele-frequency information was unavailable, we calculated the *F* value using the formula: *F* = (*β/*standard error*).*^92^^,^^93^^,^^94^

#### Outcomes

Fasting blood biochemistry was measured upon the baseline survey, including glucose and lipid (LDLC, HDLC, TG, and TC, mg/dL). Patients were assessed by trained psychiatrists both at the baseline visit and during a subsequent follow-up (CAPOC at week six; CAPEC at week eight). The severity of psychiatric symptoms was quantified using the PANSS, capturing a comprehensive total score and detailed subscores reflecting positive, negative, and general psychopathology domains. The percentage reduction in the total PANSS was applied as the primary outcome to assess the efficacy of antipsychotic treatments, and was calculated according to the formula below.^95^ Given that treatment efficacy varies across different symptom domains, the percentage reduction in the three subscales in PANSS served as the secondary outcomes. We conducted a sensitivity analysis using a binary classification of better antipsychotic efficacy, defined as achieving a PANSS percentage reduction greater than the median value.

PANSSpercentagereduction=(PANSSendpointscore−PANSSbaselinescore)/(PANSSbaselinescore−30)×100

∗ When analyzing subscale scores, baseline adjustment of 30 was not applied.

#### Covariates

The present drug-target genetic association analysis was conditioned on the following covariates: gender, age, age,^2^ study centers, the top five genetic principal components (PCs), course of SCZ, and previous medication. The assigned type of antipsychotic drug during follow-up was further adjusted when antipsychotic efficacy served as the outcome.

### Quantification and statistical analysis

#### Single drug-target analysis

A one-sample drug-target genetic association analysis was applied to evaluate the causal effects of drug targets on the outcomes. The characteristics were described by the median-dichotomized drug-target GRSs, which predicted relatively lower and higher concentrations of lipid and glucose. Associations between drug-target GRSs and baseline characteristics were tested, in which the linear trends were assessed by treating GRSs as continuous variables.

We investigated the associations between drug-target GRSs and measured lipid or glucose levels using linear regression. The drug-target GRSs significantly associated with lipid or glucose traits - representing genetically proxied lipid-lowering or glucose-lowering effects - were subsequently regressed on the PANSS percentage reduction.

In addition, we assessed the associations between levels of glucose, lipid, and the antipsychotic efficacy using both conventional prospective analysis and one-sample MR analysis. The latter applied a two-stage linear regression for causal estimation: glucose or lipid was regressed on the corresponding traditional GRS. The PANSS percentage reduction was regressed with the traditional GRS-predicted glucose or lipids.

#### Stratified analysis and independent validation

##### Stratified analysis

To further examine the effects of identified drug targets on antipsychotic efficacy among participants under specific baseline metabolic conditions, classes of antipsychotics, and specific clinical characteristics, we performed stratified analyses. These analyses were conducted according to: (1) hyperlipemia (baseline TG ≥ 151 mg/dL [1.70 mmol/L] or baseline HDL ≤ 40mg/dL [1.04 mmol/L]),^96^ prediabetes or diabetes status (baseline glucose ≥ 100.8 mg/dL [5.6 mmol/L])^97^; (2) antipsychotics with stronger (risperidone, olanzapine, quetiapine, or clozapine) or milder (aripiprazole, ziprasidone, perphenazine, or haloperidol) metabolic side effects; (3) clinical stage of first-episode, drug naive (individuals with <2 years of first-episode SCZ course, and <14 days of antipsychotic exposure) or recurrent patients; (4) gender. We additionally tested for non-additive interaction by including an interaction term between the drug-target GRS and the stratifying variable.

##### Independent validation

To validate the identified associations between drug targets and antipsychotic efficacy, we applied the CAPEC study, a cohort of Han Chinese participants diagnosed with SCZ disorder independent from the CAPOC, as the replication dataset.

##### Two-sample Mendelian randomization analysis

We integrated the non-overlapping GWASs of the metabolic indicators (GWAS of lipid from the GLGC, GWAS of glucose from the Taiwan Biobank) and PANSS percentage change (CAPOC study) among East Asians to replicate the causal effects of each drug target using two-sample MR analysis.

Genetic instruments for each drug target were selected using the same criteria applied in our one-sample MR analysis. Mendelian randomization pleiotropy residual sum and outlier (MR-PRESSO)^98^ was applied. Outlier SNPs (global test *p* < 0.05) were excluded to minimize pleiotropy bias. For the causal estimation, inverse-variance-weighted (IVW)^99^ was applied as the primary analysis, along with the weighted median (WM)^100^ method as a sensitivity analysis. The IVW method provided an unbiased causal estimate if there was no horizontal pleiotropy. WM gave valid tests even if the prevalence of invalid SNPs was up to 50%.^100^ We assessed directional horizontal pleiotropy using the intercept test from MR-Egger (ME) regression.^101^ Cochrane’s *Q* test was performed to assess the heterogeneity among SNPs, and a random-effect model in IVW was applied if heterogeneity existed (*p* < 0.05). Two-sample MR Analyses were carried out using R packages “TwoSampleMR” (version 0.5.6),^102^ “MR-PRESSO” (version 1.0)^98^ in the R environment (version 4.1.3).

##### Multi-omics expressions

To deepen our insight into the identified associations between lipid- and glucose-modulating targets and antipsychotic efficacy, we performed a broad downstream investigation combining multi-omics datasets across the cardiometabolic and cerebral systems.

##### Protein-level drug-target GRS analysis

We constructed the drug-target GRSs by applying pQTL data in the blood^77^ and CSF.^83^ We extracted the significant, genetic associations (*p* < 0.005) with protein concentrations in the blood from the Guangzhou Nutrition and Health study (GNHS, 2410 East Asians),^77^ and in the CSF based on 3,107 Europeans,^83^ separately. The SNPs were further restricted to independent (r^2^ < 0.1) common variants (MAF >0.005) within the 100 kb region around the target genes (Tables S6 and S7). To mimic the pharmacological effects of glucose- and lipid-lowering drugs on their target proteins, we adjusted the pQTL-GRS according to the drug–target interaction type. For targets inhibited by their corresponding drugs, the initial pQTL-GRS was multiplied by (−1) to simulate the suppressed effect; for targets stimulated by agonists, we retained the original pQTL-GRS coefficients^66^^,^^67^ (Figure S3).

Associations between the pQTL-GRSs and measured lipid, glucose, and antipsychotic efficacy were examined using linear or logistic regression models. These pQTL-GRSs enabled validation of whether these targets influence the SCZ treatment efficacy through the protein level changes in the peripheral and central compartments.

##### Mediation analysis

A mediation analysis was performed to estimate whether the effects of drug-target pQTL-GRSs were mediated by the lowering of lipids or glucose. We conditioned the pQTL-GRS on the GRS of lipids (for lipid-lowering targets) and the GRS of glucose (for glucose-lowering targets). The same genetic variants as those in pQTL-GRS were used, but weighted by their effects on the measured lipid and glucose derived from independent GWAS summary statistics.^70^^,^^71^

##### Colocalization analysis

To strengthen the on-target causal effect, we performed a Bayesian colocalization analysis for each identified target, incorporating the pQTL dataset, GWASs of lipids and glucose, and GWAS of PANSS percentage reduction.^103^ The colocalization analysis tested the posterior probability of five hypotheses: H_0_: SNP was not associated with either trait. H_1_: SNP was only associated with the first trait. H_2_: SNP was only associated with the second trait; H_3_: Two independent SNPs were separately associated with the two traits; H_4_: A shared SNP was associated with both traits. The LD-pruning was performed before the Bayesian test using PLINK 1.9 (window size = 500kb, r^2^ = 0.8). The posterior probability of H4 (PP.H4) larger than 0.6 was applied to identify causal SNPs, and the SNP with the highest PP.H4 was reported as the lead shared variant within each locus.^103^^,^^104^ The colocalization analysis was performed using the R package “coloc” (version 5.2.3).^103^

##### Tissue expression and metabolic/brain associations

By leveraging the expression levels of the identified drug-target genes from the Adult Genotype Tissue Expression Project (GTEx v10),^79^ we illustrated the expression profiles for each target across different tissues. We further explored drug-target genetic associations with the laboratory and physiological indicators related to the glucose-lipid pathway and brain domain, along with the related disorders, based on GWAS summary statistics and the CAPOC study. Using individual-level data from CAPOC, we explored the associations between the identified lipid-lowering, antidiabetic drug-target GRSs with other laboratory and physical measurements, including blood pressure, pulse, QTc interval, liver enzymes, urea nitrogen, creatinine, and prolactin. Linear regression was applied.

##### Factorial Mendelian randomization

The GRSs that showed significant associations with antipsychotic efficacy in the single drug-target genetic association analysis were carried forward for the factorial MR analysis. Participants were allocated into the 2 × 2 factorial groups based on the median-dichotomized GRSs of each lipid-glucose drug-target pair: (1) genetically predicted higher levels of both lipid and glucose (reference group); (2) genetically predicted lower lipid but higher glucose; (3) genetically predicted higher lipid but lower glucose; (4) genetically predicted lower levels of both lipid and glucose, analogous to a factorial RCT involving two drug classes. Joint associations between the two types of targets on the reduction rates of PANSS were evaluated using linear regression. Non-additive interaction was assessed by including the product term of continuous GRSs of these two targets.

##### Model diagnostics

For all linear regression models, we assessed for heteroskedasticity using the Breusch–Pagan test^105^ and the White test.^106^ We additionally calculated heteroskedasticity-consistent (HC3)-robust standard errors via a sandwich variance–covariance estimator, reporting both conventional and HC3-based 95% CIs and *p* values. For logistic regression models, we focused on HC3-robust standard errors for the log-odds. In both linear and logistic models, we examined influential points using Cook’s distance (>0.5) and studentized deleted residuals (>4),^107^^,^^108^^,^^109^ refitted models after excluding flagged observations to assess the sensitivity of the estimates. We classified each effect as “stable” if (i) the direction of association was unchanged between the primary and sensitivity models, (ii) the 95% CIs overlapped, and (iii) statistical significance at α = 0.05 was concordant across models; otherwise, the effect was labeled “sensitive”. Variance inflation factors (VIFs) were calculated for all covariates to evaluate multicollinearity, and VIF values >10 were considered indicative of potentially problematic multicollinearity.

All analyses were performed in the R environment (version 4.1.3).^102^ Considering the multiple tests of drug targets and outcomes, significance was defined as P_FDR_ < 0.05, and nominal *p* < 0.05 was considered suggestive. In the factorial MR analysis, we applied the Bonferroni correction for the total PANSS and three subscales, setting the significance threshold at *p* < 0.05/4 = 0.0125. Data were analyzed from August 2024 to December 2025.

## Supplemental information

Document S1. Figures S1–S7 and Tables S3–S11, S14–S42, and S45–S55

Table S1. STROBE-MR checklist of recommended items to address in reports of Mendelian randomization studies, related to Figures 1–4

Table S2. Instrument variables applied for lipid-lowering and antidiabetic drug-target GRS, related to Figure 1

Table S12. Tests of the linear regressions, related to Figures 2–4

Table S13. Heteroskedasticity test of logistic regressions, related to Figures 2–4

Table S43. Stratified analysis between APOC3 and GCK GRSs and PANSS reduction rate, related to Figure 4

Table S44. Stratified analysis between APOC3 and GCK drug-target GRSs and better antipsychotic efficacy, related to Figure 4

Document S2. Article plus supplemental information
