# Supplementary material for: Lipid-lowering and glucose-lowering drug targets differentially modulate antipsychotic treatment efficacy in schizophrenia
Source: Cell Rep Med. 2026 Mar 17;7(3):102653. doi: 10.1016/j.xcrm.2026.102653 (PMC13006399; doi:10.1016/j.xcrm.2026.102653)
Supplement: Document S1. Figures S1–S7 and Tables S3–S11, S14–S42, and S45–S55 [file mmc1.pdf]

Cell Reports Medicine, Volume *7*

Supplemental information

Lipid-lowering and glucose-lowering drug targets differentially modulate antipsychotic treatment efficacy in schizophrenia

Yunqing Zhu, Rui Yuan, Zhe Lu, Yuyanan Zhang, Zhewei Kang, Xiaoyang Feng, Guorui Zhao, Junyuan Sun, Jing Guo, Tong Yu, Yang Yang, Yaoyao Sun, and Weihua Yue

Supplemental Figures

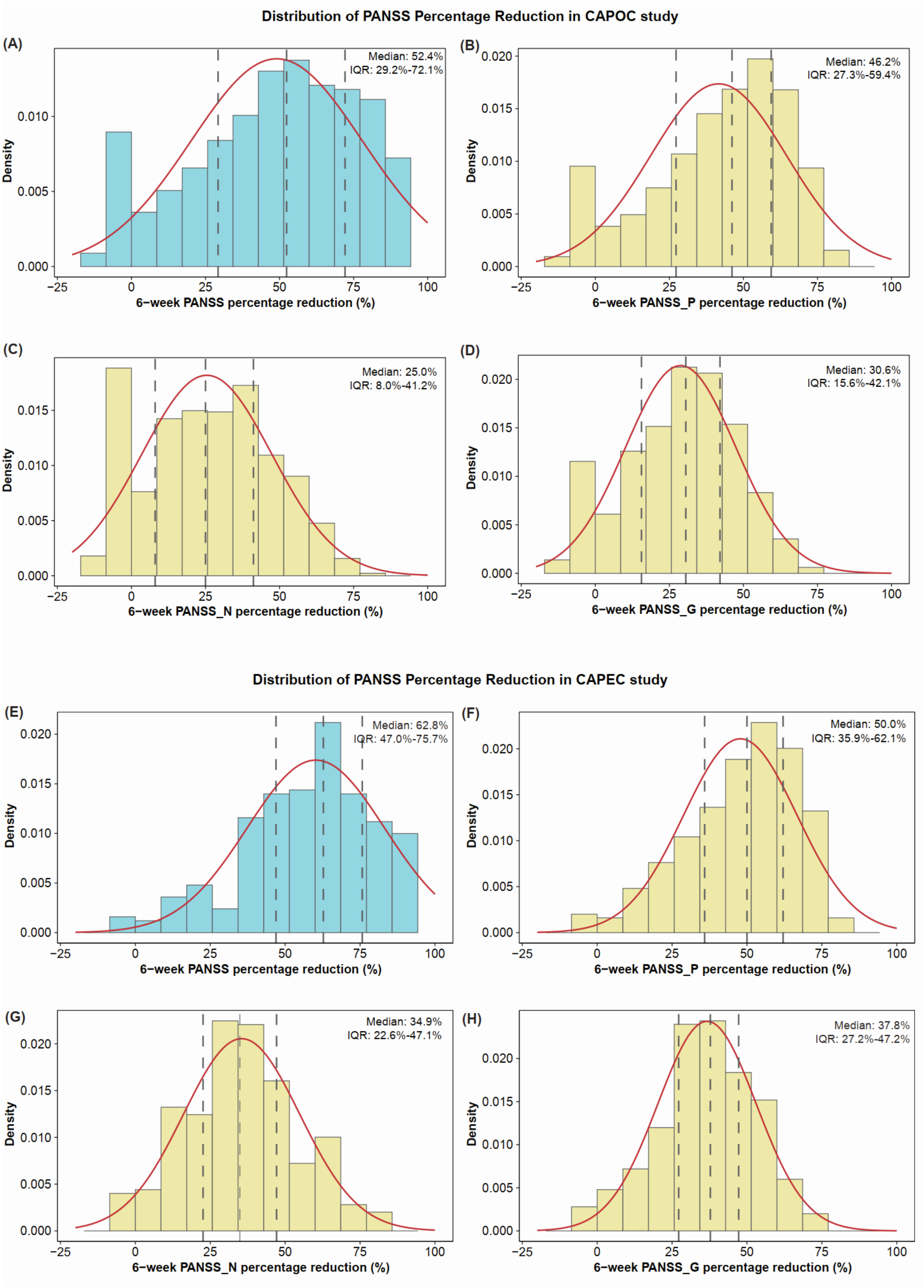

Figure S1. Distribution of PANSS percentage reduction. Related to Table 1.

Panel A-D exhibited the six-week percentage reduction of Positive and Negative Syndrome Scale (PANSS) total score, positive (P), negative (N), and general psychopathology (G) subscales in the Chinese Antipsychotics Pharmacogenomics Consortium (CAPOC) study. **Panel E-H** exhibited the eight-week percentage reduction of PANSS total score, PANSS_P, PANSS_N, and PANSS_G

subscales in the Chinese Antipsychotics Pharmacogenetics Consortium (CAPEC) study. The median and interquartile range (IQR) values were displayed.

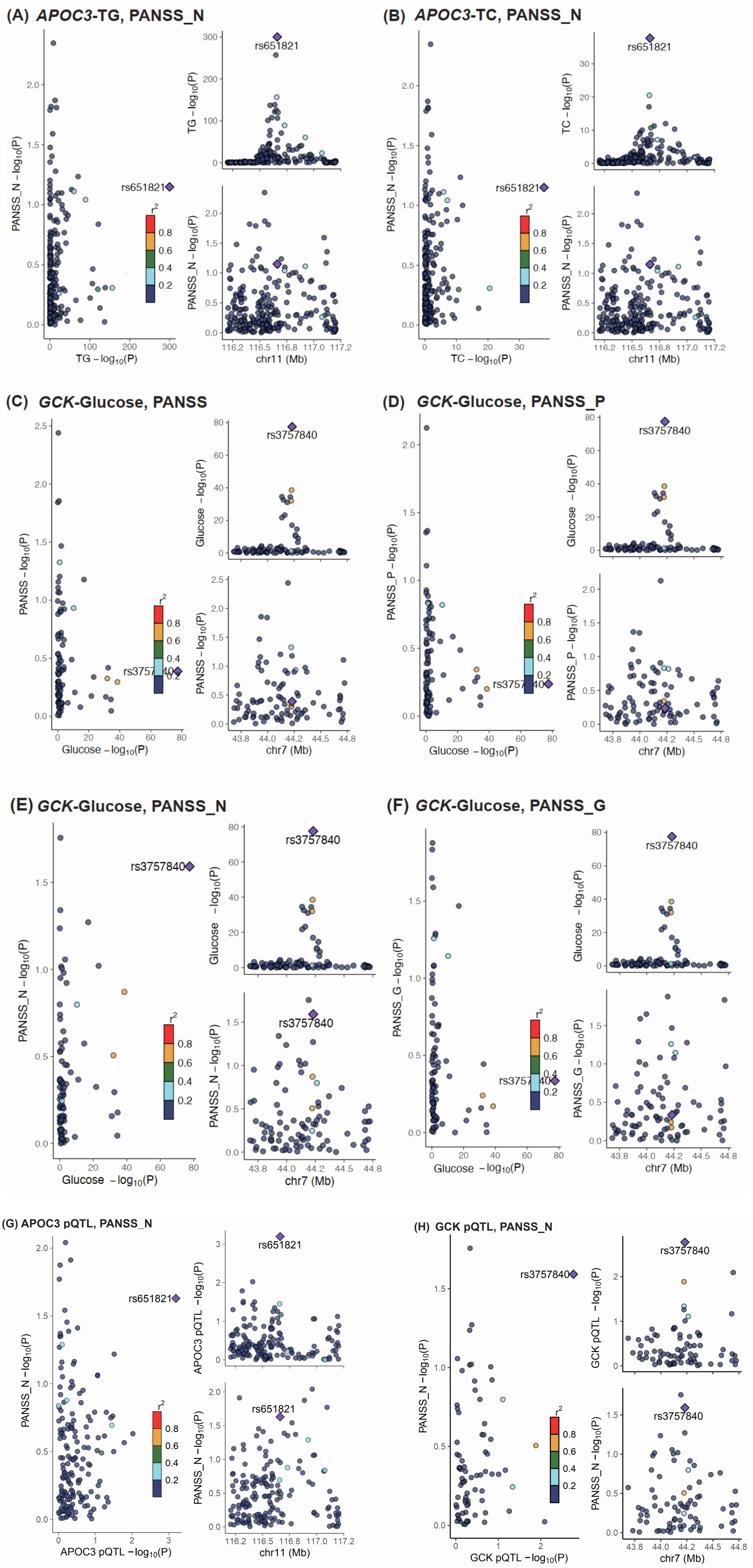

(A) APOC3-TG, PANSS_N
(B) APOC3-TC, PANSS_N
(C) GCK-Glucose, PANSS
(D) GCK-Glucose, PANSS_P
(E) GCK-Glucose, PANSS_N
(F) GCK-Glucose, PANSS_G
(G) APOC3 pQTL, PANSS_N
(H) GCK pQTL, PANSS_N
rs651821
rs3757840
PANSS_N −log10(P)
TG −log10(P)
TC −log10(P)
PANSS −log10(P)
PANSS_P −log10(P)
PANSS_G −log10(P)
Glucose −log10(P)
APOC3 pQTL −log10(P)
GCK pQTL −log10(P)
chr11 (Mb)
chr7 (Mb)
r2
0.8
0.6
0.4
0.2

Figure S2. Colocalization analysis between GWAS of glucose and lipids, *APOC3* and *GCK* pQTL data, GWAS of PANSS percentage reduction. Related to Figure 3.

Each panel showed a colocalized locus (PP.H4>0.6) identified for the corresponding trait pair. The associations for each trait were shown on the right, and the combined association for pairwise traits was shown on the left. The candidate-shared causal variant identified by pairwise colocalization analysis was shown in the purple diamond. The color of each variant represented its linkage distribution (LD) relationship with the candidate-shared causal variant. All genomic location is based on the reference genome hg19, and LD calculation was based on 1000 Genomes in Phase III of the East Asian population.

(A) *APOC3* locus for TG-PANSS_N percentage reduction; (B) *APOC3* locus for TC-PANSS_N percentage reduction; (C) *GCK* locus for glucose-PANSS percentage reduction; (D) *GCK* locus for glucose-PANSS_P percentage reduction; (E) *GCK* locus for glucose-PANSS_N percentage reduction; (F) *GCK* locus for glucose-PANSS_G percentage reduction; (G) *APOC3* locus for APOC3 protein in blood – better antipsychotic efficacy of PANSS_N; (H) *GCK* locus for GCK protein in CSF- PANSS_N percentage reduction.

Abbreviations: *APOC3*, apolipoprotein C3; *GCK*, glucokinase; TG, triglyceride; TC, total cholesterol; PANSS, Positive and Negative Syndrome Scale; N, Negative scale; G, General psychopathology scale; P, Positive scale; pQTL, protein quantitative trait loci - genetic risk score; CSF, cerebrospinal fluid.

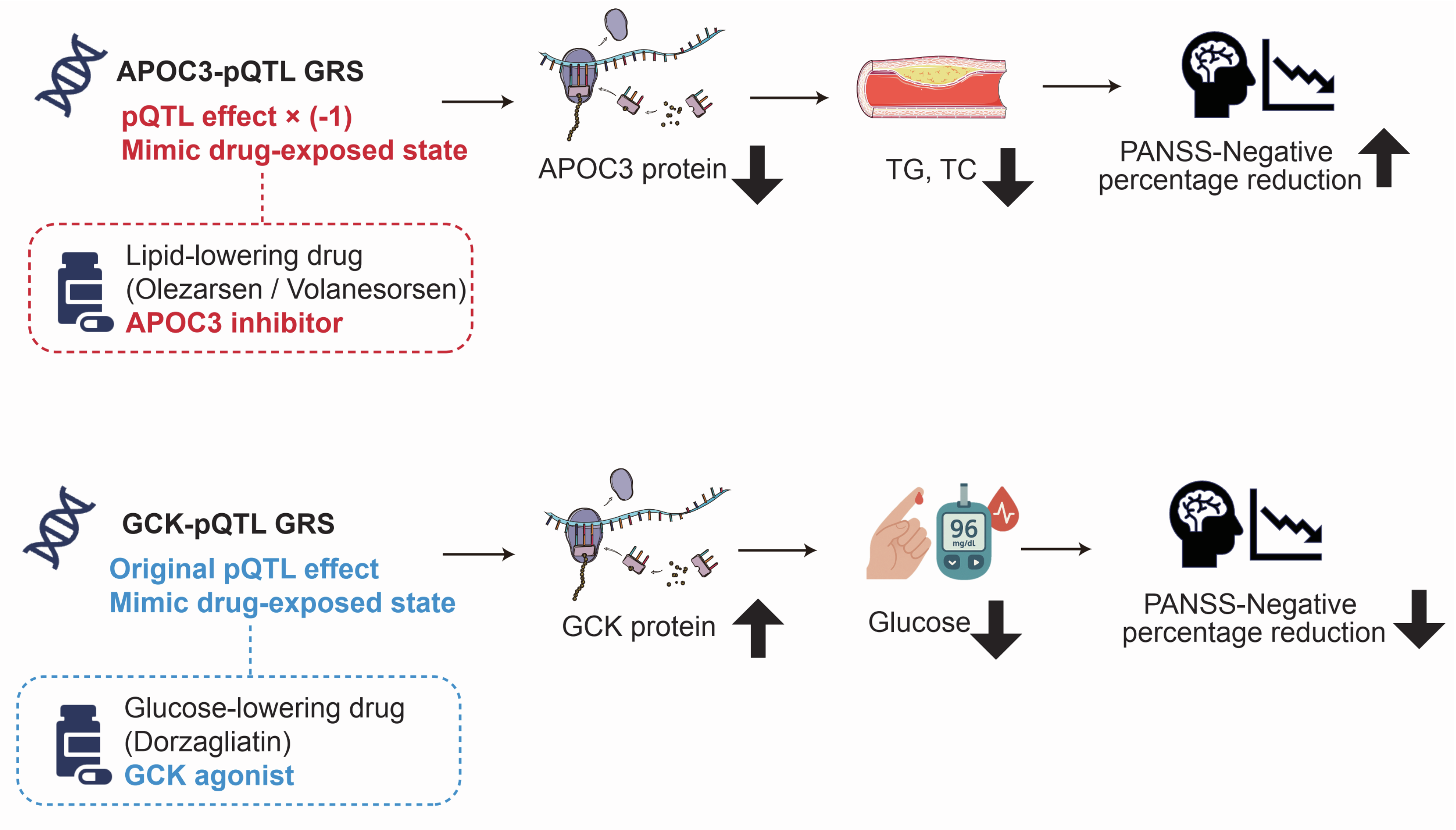

Figure S3. Construction of *APOC3, GCK* pQTL GRSs and their associations with lipid, glucose, and PANSS-negative percentage reduction. Related to Figure 3.

Notes: We calculated the drug-target pQTL-GRSs following published drug-target genetic association studies (PMID: 40105833, 35921096)

Abbreviations: *APOC3*, apolipoprotein C3; *GCK*, glucokinase; GRS, genetic risk score; TG, triglyceride; TC, total cholesterol; PANSS, Positive and Negative Syndrome Scale; N, Negative scale; protein quantitative trait loci (pQTL).

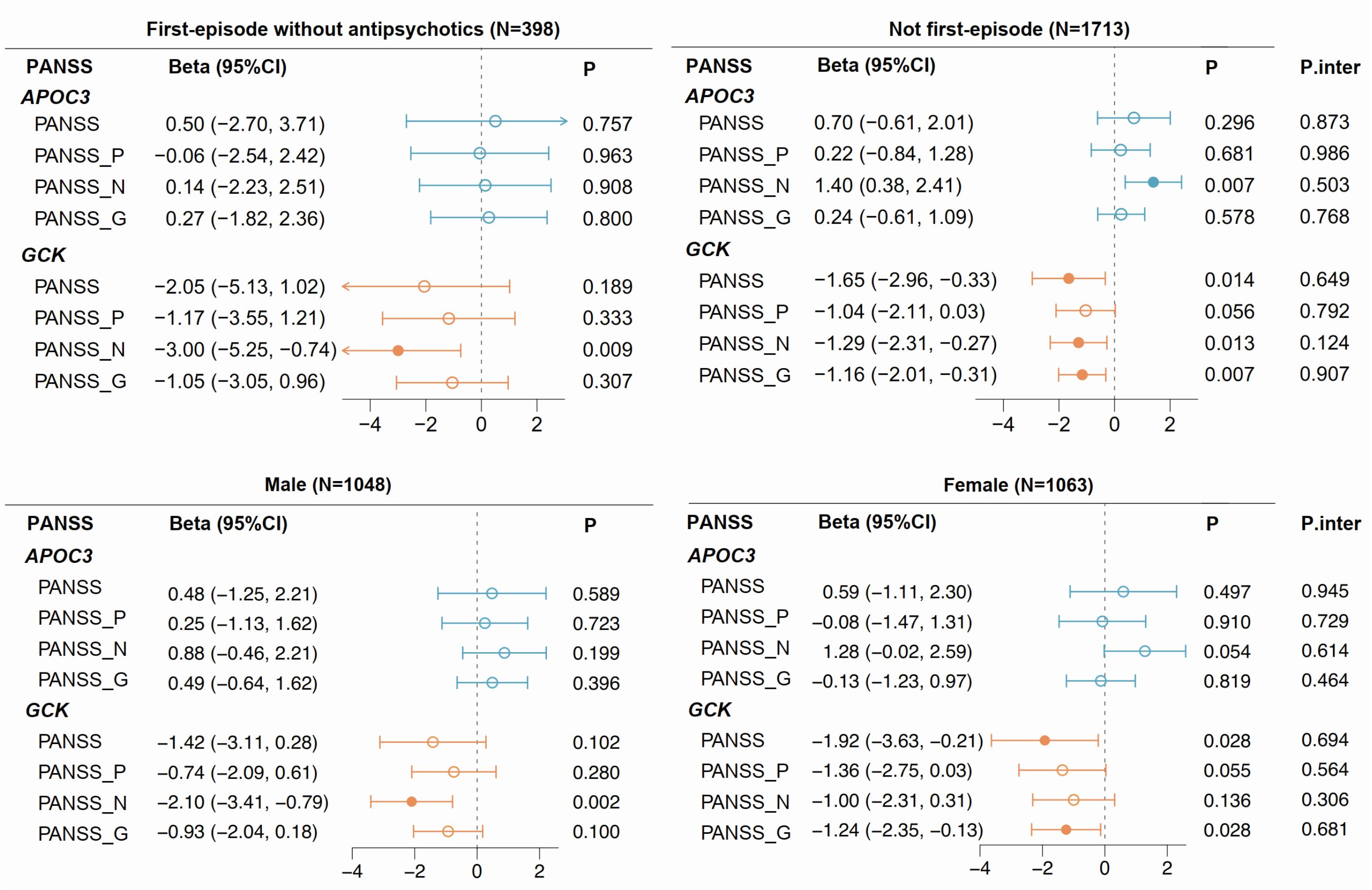

Figure S4. Stratified analysis by the schizophrenia course and gender. Related to Figure 4.

Notes: The beta (95%CIs) represented the average percentage reduction in the PANSS for per standard deviation increase in the drug-target GRS.

The first episode without antipsychotics was defined as individuals with < 2 years of first-episode schizophrenia course, and < 14 days of antipsychotic exposure (N=398), others were classified as the non-first-episode patients (N=1713).

Abbreviations: *APOC3*, apolipoprotein C3; *GCK*, glucokinase; TG, triglyceride; PANSS, Positive and Negative Syndrome Scale; N, Negative scale; G, General psychopathology scale; P, Positive scale; GRS, genetic risk score.

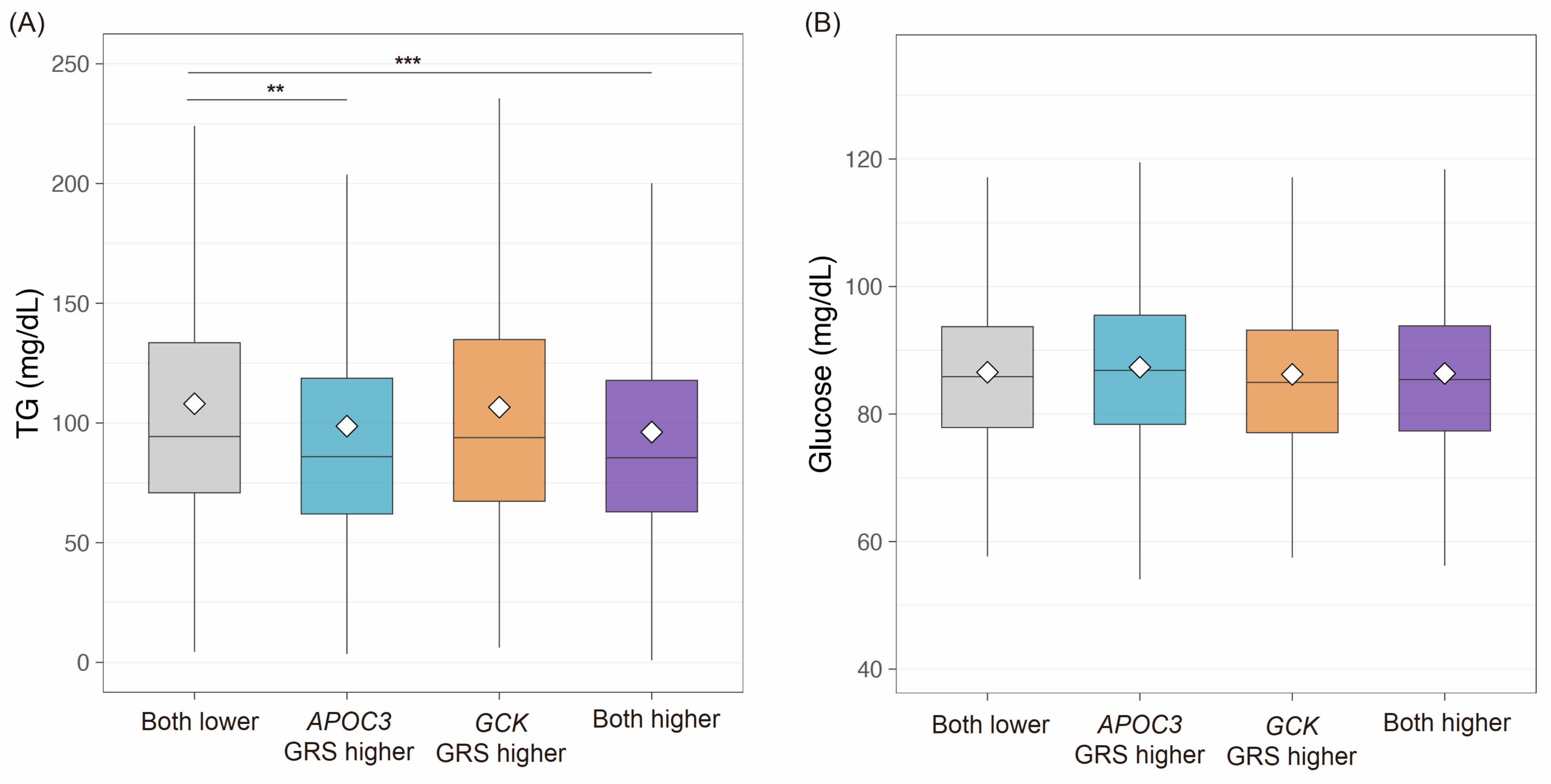

Figure S5. Triglyceride and glucose by 2*2 factorial MR study of *APOC3* and *GCK* GRSs in the CAPOC study. Related to Figure 1.

Notes: Full results could be found in Tables S48-49.* P<0.05, ** P<0.01, *** P<0.001.

Abbreviations: *APOC3*, apolipoprotein C3; *GCK*, glucokinase; TG, triglyceride; GRS, genetic risk score; CAPOC, Chinese Antipsychotics Pharmacogenomics Consortium; MR, mendelian randomization.

PANSS	Group	PANSS percentage reduction	Beta (95%CI)	P.Interaction
PANSS	Both lower	50.0 (31.2, 71.7)	Reference	0.704
	APOC3 GRS higher	51.0 (33.0, 75.0)	0.57 (−2.85, 3.99)	
	***GCK* GRS higher**	**47.4 (25.0, 72.0)**	**−3.46 (−6.89, −0.04)**	
	Both higher	47.7 (27.8, 68.4)	−2.88 (−6.39, 0.63)	
PANSS_P	Both lower	42.3 (27.8, 60.0)	Reference	0.916
	APOC3 GRS higher	43.1 (30.0, 60.9)	0.61 (−2.14, 3.35)	
	GCK GRS higher	40.8 (24.8, 59.3)	−1.95 (−4.70, 0.80)	
	Both higher	40.4 (26.9, 57.7)	−2.17 (−4.98, 0.65)	
PANSS_N	Both lower	26.3 (10.0, 42.7)	Reference	0.525
	APOC3 GRS higher	27.3 (9.1, 42.3)	0.87 (−1.76, 3.50)	
	***GCK* GRS higher**	**23.7 (7.1, 39.5)**	**−3.08 (−5.71, −0.44)**	
	Both higher	24.6 (5.8, 40.7)	−2.04 (−4.74, 0.65)	
PANSS_G	Both lower	29.6 (18.4, 42.0)	Reference	0.962
	APOC3 GRS higher	29.9 (16.4, 44.2)	0.00 (−2.22, 2.22)	
	GCK GRS higher	28.0 (14.2, 41.2)	−2.20 (−4.43, 0.02)	
	Both higher	27.9 (14.8, 41.1)	−2.15 (−4.42, 0.13)	

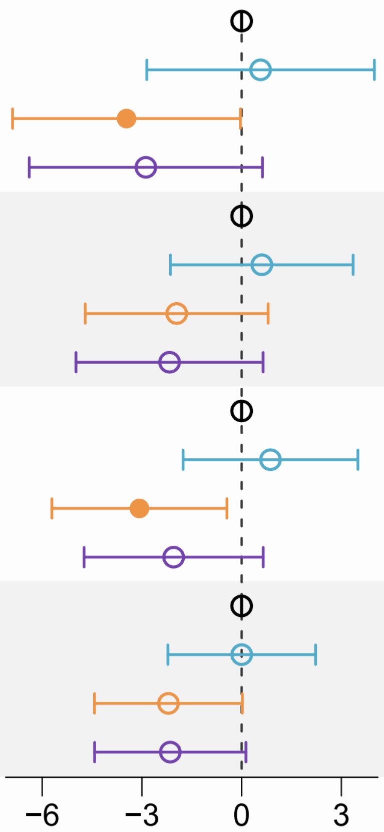

Figure S6. Combined and interactive effects of *APOC3* and *GCK* on the percentage reduction of PANSS. Related to Figure 1.

Notes: The beta (95%CIs) represented the average percentage reduction in the PANSS of the group with higher *APOC3* GRS, higher *GCK* GRS, and both higher GRSs (joint association), versus the reference group – those with both lower GRSs. The statistical significance of interactions was tested through a product term of the continuous *APOC3, GCK* GRSs. Full results could be found in Tables S50-53.

Abbreviations: *APOC3*, apolipoprotein C3; *GCK*, glucokinase; TG, triglyceride; PANSS, Positive and Negative Syndrome Scale; N, Negative scale; G, General psychopathology scale; P, Positive scale; GRS, genetic risk score.

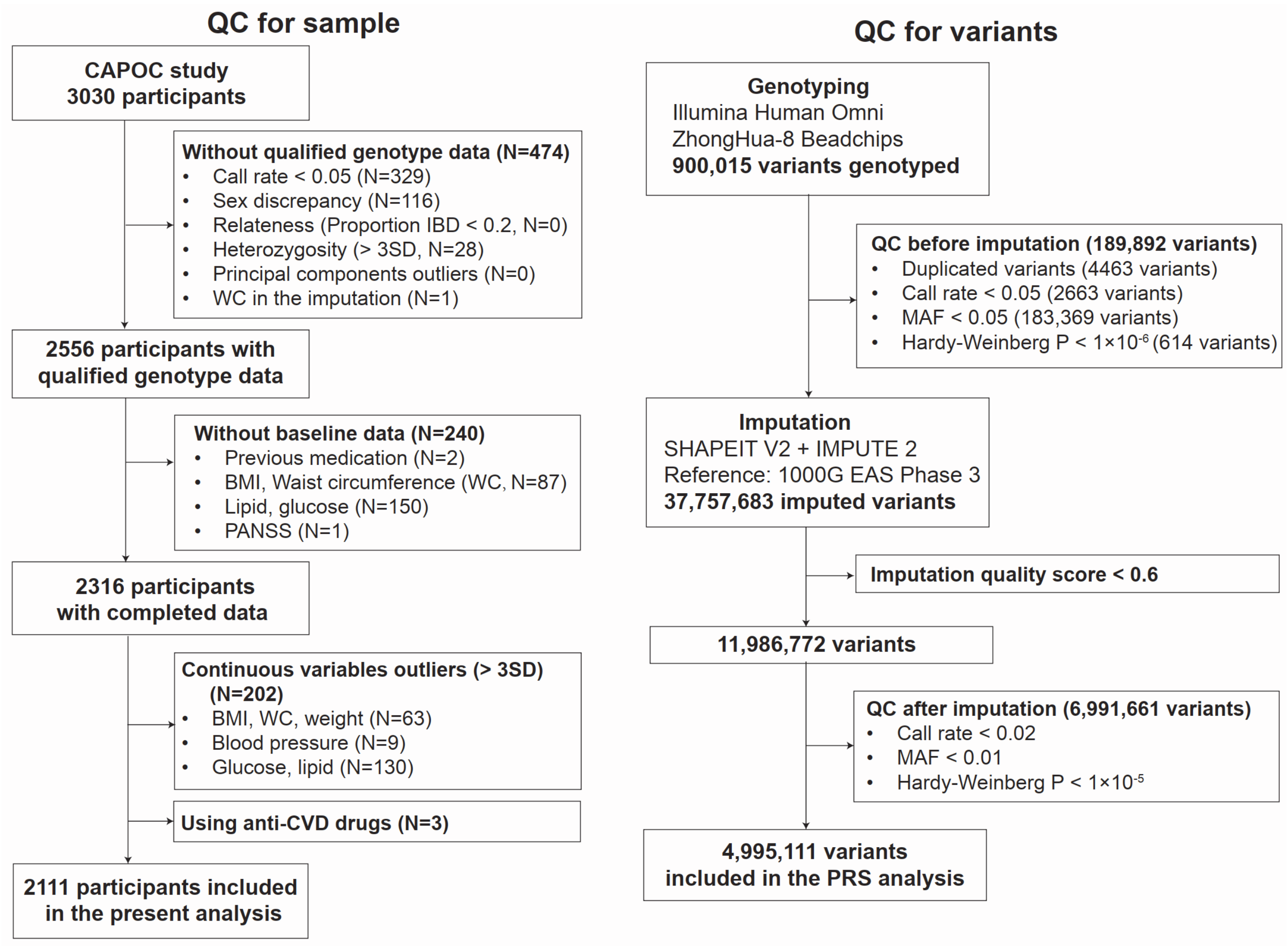

Figure S7. Quality control for participants and variants in the CAPOC study. Related to Table 1.

Notes: Abbreviations: QC, Quality control; CAPOC, Chinese Antipsychotics Pharmacogenomics Consortium; IBD, Identical by Descent; SD, standard deviation; WC, waist circumference; BMI, body mass index; CVD, cardiovascular disease; PANSS, Positive and Negative Syndrome Scale; MAF, minor allele frequency; EAS, East Asian; 1000G EAS Phase 3, 1000 Genomes Project Phase 3 East Asian (EAS) population; PRS, Polygenic Risk Score.

Supplemental Tables

Table S3. Instrument variables applied for TG GRS. Related to Figure 3.

SNP	CHR	BP	P	EA	OA	Beta	SE	EAF
rs1077835	15	58723426	5.01E-36	G	A	0.070	0.005	0.413
rs116111528	6	32313512	2.52E-11	T	C	0.087	0.012	0.0457
rs1260326	2	27730940	1.80E-75	C	T	-0.098	0.005	0.48
rs13246993	7	73022746	3.62E-54	A	G	-0.135	0.008	0.105
rs139154032	11	116678000	1.83E-11	A	G	-0.137	0.019	0.0164
rs1475537	9	92212750	8.93E-10	T	C	0.037	0.006	0.263
rs16990971	20	44601293	3.12E-13	G	A	0.079	0.010	0.0609
rs174559	11	61581656	1.32E-12	A	G	0.041	0.006	0.409
rs2954021	8	126482077	7.97E-42	A	G	0.071	0.005	0.435
rs34828061	8	59382169	1.80E-08	G	A	-0.033	0.006	0.262
rs35570672	8	18272635	8.75E-15	C	T	-0.041	0.005	0.434
rs3752442	4	3446883	1.09E-11	G	A	-0.038	0.005	0.453
rs3755980	4	88042251	4.85E-09	T	C	0.032	0.005	0.364
rs4418728	10	94839724	3.28E-09	G	T	0.039	0.006	0.214
rs4495740	1	63124465	1.53E-34	G	T	-0.082	0.006	0.186
rs4704834	5	156443066	3.51E-18	A	G	-0.053	0.006	0.248
rs483082	19	45416178	4.37E-49	T	G	0.113	0.007	0.166
rs58542926	19	19379549	3.00E-17	T	C	-0.090	0.010	0.067
rs651821	11	116662579	4.95E-525	C	T	0.291	0.006	0.283
rs6706783	2	21191409	1.44E-13	A	T	0.062	0.008	0.105
rs7078456	10	65269479	1.53E-09	C	T	0.034	0.005	0.33
rs7165077	15	58686809	2.89E-18	T	C	-0.050	0.005	0.359
rs74855321	8	19836984	2.40E-92	T	C	-0.177	0.008	0.0986
rs7928320	11	116942753	2.98E-42	G	C	-0.117	0.008	0.103
rs7928320	11	116942753	2.98E-42	G	C	-0.117	0.008	0.103
rs821840	16	56993886	4.51E-10	G	A	-0.044	0.007	0.163
rs9687832	5	55861595	4.44E-08	A	G	0.045	0.008	0.111

Notes: R^2 for SNP: $R^2 = \Sigma\beta^2 \times 2 \times EAF \times (1 - EAF)$, where β denoted the effect size.

F for each drug-target GRS: $F = [(N - K - 1) / K] \times R^2 / (1 - R^2)$, where N was the sample size and K was the number of SNPs included in the GRS.

R^2 of TG GRS = 0.075; F of TG GRS = 439.83.

Abbreviations: CHR, chromosome; BP, base pair; EA, effect allele; OA, other allele; SE, standard error; EAF, effect allele frequency; GRS, genetic risk score; TG, triglyceride.

Table S4. Instrument variables applied for TC GRS. Related to Figure 3.

SNP	CHR	BP	P	EA	OA	Beta	SE	EAF
rs10438978	18	47158186	2.72E-10	T	C	-0.028137	0.00420706	0.277
rs1065853	19	45413233	5.80E-314	T	G	-0.370244	0.00922769	0.0503
rs1077835	15	58723426	4.21E-53	G	A	0.0633784	0.00390075	0.455
rs10846744	12	125312425	1.12E-09	G	C	-0.0291246	0.00451383	0.376
rs10860591	12	100822511	3.83E-14	C	T	0.0341989	0.0042668	0.281
rs10889348	1	63078222	9.17E-20	T	A	-0.0497546	0.00516258	0.163
rs11102967	1	109817245	4.29E-68	C	T	-0.137375	0.00743748	0.0707
rs112784971	8	59406490	8.27E-25	T	C	-0.0462477	0.00424522	0.27
rs11320208	1	234850422	2.84E-20	A	C	-0.0412222	0.00421865	0.27
rs11601507	11	5701074	1.04E-09	A	C	0.0590618	0.00913568	0.084
rs12162136	17	67082266	4.63E-17	G	A	0.0340664	0.00383063	0.408
rs12173764	6	31314012	5.05E-14	T	C	-0.0322456	0.00404242	0.405
rs12233358	22	30432631	3.62E-09	G	C	0.0399809	0.00639682	0.0956
rs12314392	12	110010866	2.49E-09	G	A	0.0270593	0.00428454	0.275
rs12484801	22	44325565	1.50E-11	T	C	-0.0275028	0.00384796	0.417
rs12995972	2	62956520	7.58E-14	A	C	-0.034681	0.00437859	0.261
rs151021730	19	50031075	1.80E-08	A	G	-0.0619466	0.0103872	0.0372
rs17129638	10	113981435	2.05E-11	C	T	-0.0291265	0.00410254	0.295
rs17231506	16	56994528	1.12E-43	T	C	0.0716347	0.00487972	0.181
rs174565	11	61591636	2.05E-20	G	C	-0.0397076	0.0040483	0.4
rs17660635	8	9197250	2.44E-09	G	A	-0.10843	0.0171596	0.0122
rs184643955	16	71452979	1.88E-08	A	G	0.0986131	0.0165573	0.0134
rs2206689	20	39209518	8.44E-13	A	G	0.028864	0.0038091	0.435
rs2263608	10	113944940	4.64E-17	T	A	0.038167	0.00429186	0.265
rs2306986	4	100504575	6.37E-10	C	G	0.0402142	0.00614219	0.203
rs2714875	7	17925975	1.28E-08	A	G	0.0250345	0.00415409	0.294
rs2737265	8	116667634	1.03E-13	G	A	-0.032534	0.00412984	0.286
rs2738464	19	11242307	7.78E-80	G	C	-0.0850829	0.00424537	0.298
rs2740488	9	107661742	1.14E-63	C	A	-0.0765346	0.00428923	0.252
rs2807834	1	220970593	4.39E-14	T	G	-0.0422582	0.00528475	0.153
rs2954021	8	126482077	8.01E-43	A	G	0.0547932	0.00377102	0.44
rs3809868	17	45750596	3.29E-12	G	A	0.0296915	0.00402474	0.35
rs3810308	19	11333596	3.84E-59	C	T	-0.0750637	0.00436982	0.25
rs4344974	20	39818861	6.73E-14	T	C	-0.0401339	0.00505656	0.168
rs4454000	4	69950571	7.30E-10	G	A	-0.0272272	0.00417316	0.313
rs4622073	1	93255073	5.96E-11	A	G	-0.0554232	0.00799476	0.062
rs4690014	4	3436062	1.31E-15	A	G	-0.0331682	0.00391727	0.419
rs532436	9	136149830	4.56E-50	A	G	0.0685843	0.00435182	0.243
rs55686478	8	18250375	1.51E-09	G	A	-0.024068	0.00375991	0.491
rs56130071	7	21598753	1.56E-10	C	G	0.0299187	0.00441349	0.268
rs57176252	10	74662593	2.77E-13	A	C	-0.0356643	0.0046089	0.218
rs5758361	22	41852732	3.93E-10	C	A	0.0276585	0.00417331	0.355
rs57825321	2	21247065	8.47E-78	A	T	-0.114637	0.00579626	0.119
rs61946969	13	32946805	6.48E-15	A	G	-0.0315782	0.00382483	0.415
rs6437108	2	158455135	1.17E-17	T	C	-0.0393568	0.00434274	0.276
rs6453131	5	74644706	7.43E-65	T	G	-0.0677423	0.00376062	0.496

rs651821	11	116662579	1.85E-38	C	T	0.0571283	0.00415872	0.31
rs6663252	1	55630151	2.46E-34	C	T	-0.0743687	0.00574581	0.124
rs6874202	5	156391628	2.23E-38	T	C	-0.062447	0.00455101	0.219
rs6959252	7	25984566	1.18E-09	A	G	0.0259676	0.00403025	0.401
rs7165077	15	58686809	2.34E-19	T	C	-0.0513008	0.00538352	0.359
rs72607108	9	107592641	1.47E-11	G	T	-0.0494518	0.00691596	0.0793
rs7312955	12	123747783	1.68E-08	A	C	0.0254173	0.00425314	0.29
rs7400614	13	114551980	3.36E-11	A	C	0.0304648	0.0043381	0.301
rs75352129	2	20848192	7.85E-28	T	C	0.136357	0.0117723	0.0263
rs77303550	16	72079657	9.24E-67	T	C	-0.0881541	0.0048214	0.228
rs7770628	6	161018174	2.91E-15	C	T	0.0635399	0.00759832	0.118
rs78753419	1	55811671	3.41E-10	T	A	0.0345461	0.00519426	0.155
rs7954039	12	121398654	1.67E-15	A	C	0.0318793	0.00377914	0.492
rs814295	2	27743215	1.40E-33	G	A	-0.0510712	0.00399223	0.321
rs9376090	6	135411228	4.95E-14	C	T	-0.0448819	0.00562452	0.281
rs9399668	6	101161950	7.42E-09	T	C	-0.0257582	0.00420637	0.292
rs9953437	18	47120600	3.98E-49	A	G	0.0594315	0.00380832	0.428

Notes: R^2 for SNP: $R^2 = \Sigma\beta^2 \times 2 \times EAF \times (1 - EAF)$, where β denoted the effect size.

F for each drug-target GRS: $F = [(N - K - 1) / K] \times R^2 / (1 - R^2)$, where N was the sample size and K was the number of SNPs included in the GRS

R^2 of TC GRS = 0.066; F of TC GRS = 164.32

Abbreviations: CHR, chromosome; BP, base pair; EA, effect allele; OA, other allele; SE, standard error; EAF, effect allele frequency; GRS, genetic risk score; TG, triglyceride.

Table S5. Instrument variables applied for glucose GRS. Related to Figure 3.

SNP	chr	bp19	EA	OA	Beta	SE	EAF	P
rs10010131	4	6292915	A	G	-0.045	0.008	0.069	3.55E-08
rs10158845	1	43455283	A	G	-0.026	0.004	0.513	4.68E-09
rs10267664	7	156770498	C	G	-0.031	0.005	0.294	1.51E-10
rs10441113	7	44288665	A	G	-0.041	0.007	0.127	9.12E-10
rs10811658	9	22128600	A	G	-0.042	0.005	0.478	9.55E-21
rs10830963	11	92708710	C	G	-0.084	0.005	0.590	2.63E-77
rs10882083	10	94321973	C	G	0.026	0.005	0.372	1.45E-08
rs12429454	13	28481210	A	G	0.047	0.004	0.471	4.57E-28
rs12443160	15	40656141	T	C	0.024	0.004	0.436	1.66E-08
rs1260326	2	27730940	T	C	-0.059	0.004	0.492	9.12E-46
rs13023591	2	173595699	T	C	0.050	0.004	0.514	7.24E-31
rs1402837	2	169757354	T	C	0.106	0.004	0.404	1.23E-133
rs1406981	6	117269641	T	C	0.027	0.004	0.620	1.95E-10
rs1409333	10	95390919	T	C	-0.028	0.004	0.444	2.40E-11
rs1420566	16	56065760	A	G	0.024	0.004	0.406	3.80E-08
rs16922302	9	672920	C	G	0.059	0.005	0.265	5.25E-35
rs17085675	5	95727664	A	T	0.043	0.004	0.682	1.62E-21
rs17168486	7	14898282	T	C	0.055	0.004	0.479	7.76E-40
rs174554	11	61579463	A	G	0.047	0.004	0.422	1.15E-27
rs1974619	7	15065300	T	C	0.069	0.005	0.675	1.74E-49
rs204926	11	8255106	A	G	-0.038	0.005	0.333	5.75E-16
rs2237896	11	2858440	A	G	-0.045	0.005	0.345	7.08E-22
rs2273800	14	100847707	A	G	0.027	0.004	0.554	6.17E-10
rs231840	11	2724130	T	C	0.032	0.006	0.813	1.32E-08
rs243020	2	60585028	A	G	-0.031	0.005	0.351	1.15E-11
rs2908289	7	44223942	A	G	0.129	0.005	0.196	3.63E-131
rs340515	2	45188370	T	G	-0.097	0.004	0.530	3.16E-104
rs340878	1	214157049	C	G	0.036	0.006	0.814	5.25E-10
rs35674932	10	12261810	A	T	-0.037	0.004	0.449	2.04E-18
rs363404	10	119009631	T	C	-0.025	0.005	0.536	3.24E-08
rs3755934	4	1236083	T	C	-0.039	0.004	0.334	4.90E-18
rs3787186	20	50153344	T	C	0.030	0.005	0.375	7.76E-11
rs3802177	8	118185025	A	G	-0.072	0.004	0.462	2.19E-67
rs419145	1	33179156	A	G	0.030	0.005	0.215	4.90E-09
rs4339696	9	4295880	T	G	-0.037	0.004	0.427	1.10E-18
rs459193	5	55806751	A	G	-0.029	0.004	0.469	5.75E-12
rs519084	13	26821164	A	T	0.026	0.005	0.433	4.79E-09
rs55804588	7	75852586	C	G	-0.041	0.007	0.861	2.63E-10
rs56237081	2	222036237	T	C	0.037	0.006	0.198	4.57E-11
rs56252704	13	33556228	A	G	0.047	0.005	0.209	1.58E-19
rs59299413	7	127843398	C	G	0.040	0.007	0.135	3.47E-09

rs6048206	20	22561382	T	C	0.096	0.006	0.857	1.95E-56
rs61839365	10	26515666	A	G	-0.036	0.005	0.265	4.68E-14
rs6495240	15	77766556	T	C	-0.029	0.004	0.655	3.16E-11
rs67131976	6	20686878	T	C	0.061	0.004	0.342	7.59E-43
rs6780171	3	185503456	A	T	0.029	0.005	0.233	5.01E-09
rs6932473	6	153438573	A	T	-0.025	0.004	0.326	1.20E-08
rs6972160	7	89815373	C	G	-0.036	0.006	0.822	6.31E-11
rs7109575	11	72463435	A	G	-0.066	0.008	0.067	1.00E-14
rs7161785	15	62395224	C	G	-0.054	0.004	0.340	1.38E-34
rs7165887	15	51750846	A	G	-0.027	0.005	0.405	4.07E-09
rs7209518	17	67580175	A	C	0.026	0.005	0.639	2.63E-08
rs883541	17	66449122	A	G	-0.026	0.004	0.525	7.59E-10
rs9263964	6	31186039	T	C	0.025	0.004	0.422	1.74E-09
rs9296285	6	39040469	A	G	0.048	0.005	0.766	5.13E-22
rs9788833	16	292772	T	C	0.027	0.004	0.413	9.33E-10
rs9873341	3	152387723	T	C	0.032	0.005	0.276	1.82E-10

Notes: R^2 for SNP: $R^2 = \Sigma\beta^2 \times 2 \times EAF \times (1 - EAF)$, where β denoted the effect size.

F for each drug-target GRS: $F = [(N - K - 1) / K] \times R^2 / (1 - R^2)$, where N was the sample size and K was the number of SNPs included in the GRS

R^2 of glucose GRS = 0.056; F of glucose GRS = 95.56

Abbreviations: CHR, chromosome; BP, base pair; EA, effect allele; OA, other allele; SE, standard error; EAF, effect allele frequency; GRS, genetic risk score; TG, triglyceride.

Table S6. Instrument variables applied for GRS of APOC3-pQTL in the blood. Related to Figure 3.

SNP	CHR	BP	EA	OA	N	MAF	Beta	SE	P	R^2	F
rs651821	11	116662579	C	T	2410	0.282197	0.106423	0.03115	0.000638	0.00458837	11.0997303

Notes: $R2 = \Sigma\beta^2 \times 2 \times EAF \times (1 - EAF)$, where β denoted the effect size.

$F = [(N - K - 1) / K] \times R^2 / (1 - R^2)$, where N=2410, K = 1

As the TG-lowering drugs (olezarsen and volanesorsen) are inhibitors of APOC3, we multiplied the initial APOC3-pQTL-GRS by (-1) to simulate the drug-induced APOC3 inhibition and the corresponding lipid-lowering effect after treatment.

Abbreviations: SNP, single nucleotide polymorphism; CHR, chromosome; BP, base pair; EA, effect allele; OA, other allele; GRS, genetic risk score; MAF, minor allele frequency.

Table S7. Instrumental variables applied for CSF-pQTL GRSs of GCK. Related to Figure 3.

Gene	SNP	CHR	BP	OA	EA	EAF_1000G EUR	EAF_1000G EAS	EAF_CAPOC	Beta	SE	P	F
GCK	rs3757840	7	44191617	T	G	0.467	0.479	0.464	0.073	0.023	0.0017	10.073724

Notes: As the allele frequency was not available in the CSF-pQTL dataset, F value was calculated by: $F= (beta/SE)^2$

As the antidiabetic drug - dorzagliatin is an agonist of GCK, we retained the original coefficients when calculating GCK-pQTL GRS to mimic the activation of GCK and the glucose-lowering effect of the treatment.

Abbreviations: SNP, single nucleotide polymorphism; CHR, chromosome; BP, base pair; EA, effect allele; OA, other allele; GRS, genetic risk score; CSF, cerebrospinal fluid.

EAF information was based on the 1000 Genomes Project Phase 3; CAPOC, Chinese Antipsychotics Pharmacogenomics Consortium.

Table S8. Associations between lipid-lowering drug-target GRSs, *GCK* GRS, with lipids in the CAPOC study. Related to Figure 2.

Trait	Gene	Beta	SE	CI	P	P.FDR
LDLC	*ABCA1*	0.437	0.618	-0.774 ~ 1.649	0.479	0.561
	PCSK9	-0.607	0.611	-1.805 ~ 0.590	0.320	0.451
	APOB	-1.667	0.620	-2.882 ~ -0.451	0.007	0.031
	LDLR	-1.068	0.623	-2.290 ~ 0.155	0.087	0.190
	LPA	-0.612	0.618	-1.825 ~ 0.600	0.322	0.451
	HMGCR	-1.409	0.620	-2.625 ~ -0.194	0.023	0.081
	APOC3	-1.168	0.621	-2.385 ~ 0.050	0.060	0.150
	GCK	0.490	0.619	-0.724 ~ 1.704	0.429	0.536
HDLC	*PPARG*	-0.373	0.287	-0.935 ~ 0.189	0.193	0.375
	HCAR3	-0.245	0.288	-0.809 ~ 0.320	0.395	0.512
	HCAR2	-0.245	0.288	-0.809 ~ 0.320	0.395	0.512
	ABCA1	-0.853	0.284	-1.410 ~ -0.296	0.003	0.021
	MTTP	-0.319	0.285	-0.878 ~ 0.240	0.264	0.431
	APOC3	-0.406	0.284	-0.963 ~ 0.152	0.153	0.315
	CETP	-1.164	0.285	-1.724 ~ -0.604	4.72E-05	0.001
	APOB	-0.116	0.290	-0.684 ~ 0.453	0.690	0.710
	LDLR	-0.708	0.282	-1.261 ~ -0.155	0.012	0.047
	LPL	-1.095	0.280	-1.644 ~ -0.545	9.72E-05	0.001
	LPA	0.172	0.284	-0.385 ~ 0.729	0.544	0.595
	GCK	0.305	0.285	-0.253 ~ 0.863	0.283	0.431
TG	*ANGPTL3*	-1.925	1.124	-4.129 ~ 0.280	0.087	0.190
	APOC3	-6.343	1.118	-8.534 ~ -4.151	1.58E-08	5.53E-07
	CETP	-2.350	1.133	-4.572 ~ -0.129	0.038	0.121
	APOB	-2.287	1.125	-4.494 ~ -0.081	0.042	0.123
	LPL	-1.220	1.106	-3.390 ~ 0.950	0.270	0.431
	GCK	-0.695	1.123	-2.896 ~ 1.506	0.536	0.595
TC	*ANGPTL3*	-0.002	0.715	-1.404 ~ 1.400	0.998	0.998
	APOB	-2.010	0.715	-3.412 ~ -0.609	0.005	0.025
	APOC3	-2.016	0.718	-3.424 ~ -0.607	0.005	0.025
	CETP	-0.308	0.719	-1.718 ~ 1.102	0.668	0.708
	HMGCR	-1.394	0.715	-2.796 ~ 0.008	0.051	0.137
	LDLR	-2.108	0.716	-3.513 ~ -0.703	0.003	0.021
	LPA	-0.873	0.712	-2.270 ~ 0.523	0.220	0.405
	PCSK9	-0.766	0.704	-2.146 ~ 0.614	0.276	0.431
	GCK	0.503	0.713	-0.895 ~ 1.901	0.481	0.561

Notes: Adjusted for age, age^2, gender, center, first five principal components, course of disease, and previous drug usage.

False discovery rate (FDR)-corrected P-value < 0.05 was applied as the significant threshold accounting for multiple tests across drug targets and outcomes. The conventional P<0.05 was applied as the threshold of suggestive significance.

Abbreviations: LDLC, low-density lipoprotein cholesterol; HDLC, high-density lipoprotein cholesterol; TG, triglyceride; TC, total cholesterol; GRS, genetic risk score; SE, standard error; CAPOC, Chinese Antipsychotics Pharmacogenomics Consortium.

Table S9. Associations between antidiabetic target GRSs, *APOC3* GRS with glucose in the CAPOC study. Related to Figure 2.

Trait	Gene	Beta	SE	CI	P	P.FDR
Glucose	*ABCB11*	-1.296	0.283	-1.851 ~ -0.742	4.80E-06	1.92E-05
	GCK	-0.675	0.283	-1.229 ~ -0.120	0.017	0.034
	GLP1R	-0.282	0.286	-0.842 ~ 0.279	0.325	0.433
	APOC3	0.214	0.284	-0.344 ~ 0.771	0.452	0.452

Notes: Adjusted for age, age^2, gender, center, first five principal components, course of disease, and previous drug usage.

False discovery rate (FDR)-corrected P-value < 0.05 was applied as the significant threshold accounting for multiple tests across drug targets and outcomes. The conventional P<0.05 was applied as the threshold of suggestive significance.

Abbreviations: GRS, genetic risk score; CAPOC, Chinese Antipsychotics Pharmacogenomics Consortium study; SE, standard error.

Table S10. Associations between drug-target GRSs and baseline characteristics. Related to Table 1.

CAPOC study								
**GRS**	**Outcome**	**Model**	**Coff***	**SE**	**CI_low**	**CI_high**	**P**	**P.FDR**
*APOC3*_TC	Gender	logistic	1.021	0.045	0.936	1.114	0.644	0.910
	Age (years)	linear	-0.022	0.021	-0.064	0.020	0.302	0.910
	Center is south	logistic	1.056	0.101	0.867	1.286	0.588	0.910
	First episode	logistic	0.979	0.057	0.875	1.095	0.707	0.910
	Course of SCZ	linear	0.580	1.297	-1.962	3.121	0.655	0.910
	With previous medication	logistic	1.025	0.048	0.932	1.127	0.614	0.910
	Education_lower than middle school	logistic	0.989	0.045	0.905	1.081	0.809	0.910
	Married	logistic	0.967	0.054	0.869	1.075	0.533	0.910
	SBP (mmHg)	linear	-0.154	0.238	-0.621	0.312	0.517	0.910
	DBP (mmHg)	linear	-0.144	0.174	-0.485	0.197	0.407	0.910
	Pulse	linear	-0.219	0.223	-0.657	0.218	0.325	0.910
	BMI (kg/m^2)	linear	-0.022	0.072	-0.164	0.120	0.757	0.910
*APOC3*_TG	Gender	logistic	0.988	0.044	0.906	1.078	0.792	0.910
	Age (years)	linear	-0.018	0.021	-0.060	0.024	0.403	0.910
	Center is south	logistic	1.041	0.097	0.862	1.259	0.675	0.910
	First episode	logistic	0.982	0.056	0.881	1.096	0.749	0.910
	Course of SCZ	linear	-0.564	1.290	-3.092	1.964	0.662	0.910
	With previous medication	logistic	1.081	0.048	0.984	1.188	0.105	0.910
	Education_lower than middle school	logistic	1.009	0.045	0.924	1.103	0.839	0.916
	Married	logistic	0.985	0.055	0.885	1.097	0.786	0.910
	SBP (mmHg)	linear	-0.405	0.237	-0.869	0.059	0.087	0.910
	DBP (mmHg)	linear	-0.280	0.173	-0.619	0.059	0.106	0.910
	Pulse	linear	-0.065	0.222	-0.500	0.370	0.770	0.910
	BMI (kg/m^2)	linear	-0.071	0.072	-0.212	0.070	0.326	0.910
*GCK*_glucose	Gender	logistic	1.045	0.044	0.959	1.139	0.318	0.910
	Age (years)	linear	-0.042	0.021	-0.083	0.000	0.050	0.910
	Center is south	logistic	1.008	0.100	0.828	1.226	0.939	0.939
	First episode	logistic	0.936	0.056	0.838	1.045	0.237	0.910
	Course of SCZ	linear	-1.222	1.285	-3.740	1.296	0.342	0.910

	With previous medication	logistic	0.948	0.048	0.863	1.042	0.270	0.910
	Education_lower than middle school	logistic	1.048	0.045	0.959	1.145	0.301	0.910
	Married	logistic	1.022	0.054	0.919	1.137	0.688	0.910
	SBP (mmHg)	linear	-0.114	0.236	-0.576	0.349	0.630	0.910
	DBP (mmHg)	linear	0.015	0.173	-0.323	0.353	0.930	0.939
	Pulse	linear	0.265	0.221	-0.168	0.698	0.231	0.910
	BMI (kg/m^2)	linear	0.011	0.072	-0.130	0.151	0.884	0.936
**CAPEC study**								
**GRS**	**Outcome**	**Model**	**Coff***	**SE**	**CI_low**	**CI_high**	**P**	**P.FDR**
*APOC3*_TC	Gender	logistic	0.912	0.136	0.699	1.191	0.500	0.852
	Age (years)	linear	0.047	0.105	-0.159	0.253	0.658	0.852
	Center	logistic	0.833	0.237	0.523	1.327	0.442	0.852
	First episode	logistic	0.818	0.149	0.611	1.094	0.175	0.852
	SBP (mmHg)	linear	-0.371	0.723	-1.787	1.045	0.608	0.852
	DBP (mmHg)	linear	-0.300	0.540	-1.358	0.758	0.579	0.852
	BMI (kg/m^2)	linear	0.396	0.244	-0.083	0.874	0.106	0.852
*APOC3*_TG	Gender	logistic	0.854	0.133	0.659	1.108	0.235	0.852
	Age (years)	linear	0.040	0.102	-0.160	0.240	0.697	0.852
	Center	logistic	0.894	0.234	0.565	1.415	0.633	0.852
	First episode	logistic	0.878	0.143	0.664	1.163	0.365	0.852
	SBP (mmHg)	linear	-0.204	0.701	-1.579	1.170	0.771	0.852
	DBP (mmHg)	linear	-0.220	0.524	-1.247	0.807	0.675	0.852
	BMI (kg/m^2)	linear	0.345	0.237	-0.120	0.810	0.147	0.852
*GCK*_glucose	Gender	logistic	0.858	0.127	0.669	1.099	0.225	0.852
	Age (years)	linear	-0.071	0.098	-0.263	0.120	0.466	0.852
	Center	logistic	0.932	0.232	0.592	1.468	0.760	0.852
	First episode	logistic	0.906	0.136	0.695	1.182	0.466	0.852
	SBP (mmHg)	linear	0.106	0.674	-1.215	1.427	0.875	0.879
	DBP (mmHg)	linear	0.077	0.504	-0.910	1.064	0.879	0.879
	BMI (kg/m^2)	linear	0.222	0.228	-0.226	0.669	0.333	0.852

Note: For logistic regression models, Coff represents the odds ratio (OR) and SE represents the standard error of log(OR); for linear regression models, "Coff" represents the regression coefficient (β) and SE represents the standard error of β.

The models were adjusted for gender, age, study centers, SCZ course, previous drug usage, PC1~5 (excluding any variable from the covariates when it served as the outcome)

Abbreviations: BMI, body mass index; SBP, systolic blood pressure; DBP, diastolic blood pressure; GRS, genetic risk score; CAPOC, Chinese Antipsychotics Pharmacogenomics Consortium study; CAPEC, Chinese Antipsychotics Pharmacogenetics Consortium.

Table S11. Baseline characteristics of participants in the CAPEC study. Related to Table 1

Baseline characteristics	All	*APOC3*_GRS		*GCK*_GRS	
		Lower	Higher	Lower	Higher
N	292	146 (50.0)	146 (50.0)	130 (44.5)	162 (55.5)
Male (%)	132 (45.2)	64 (43.8)	68 (46.6)	58 (44.6)	74 (45.7)
Female (%)	160 (54.8)	82 (56.2)	78 (53.4)	72 (55.4)	88 (54.3)
Age (years)	32.0 (23.0, 40.0)	31.5 (23.2, 39.0)	32.0 (23.0, 41.0)	31.5 (24.0, 39.0)	32.0 (23.0, 41.0)
First episode (%)	94 (32.2)	48 (32.9)	46 (31.5)	39 (30.0)	55 (34.0)
TC (mg/dL)	154.7 (135.3, 182.2)	154.7 (136.1, 185.4)	154.7 (135.3, 177.3)	157.2 (136.3, 184.5)	151.6 (135.3, 181.7)
TG (mg/dL)	93.0 (70.6, 132.9)	97.4 (75.5, 137.7)	89.5 (64.9, 128.9)	98.8 (74.6, 130.9)	89.0 (64.9, 134.6)
HDLC (mg/dL)	46.4 (36.7, 55.7)	44.5 (35.6, 55.2)	47.2 (38.5, 56.7)	45.6 (37.5, 54.9)	46.8 (36.7, 57.1)
LDLC (mg/dL)	90.9 (74.5, 119.5)	94.7 (76.4, 121.3)	88.0 (74.3, 114.3)	94.5 (75.2, 121.4)	90.5 (74.0, 116.7)
Glucose (mg/dL)	83.4 (75.5, 93.6)	82.6 (73.6, 92.4)	84.5 (77.4, 93.7)	83.9 (75.9, 93.7)	83.3 (75.3, 93.3)

Notes: The median and interquartile range (IQR) values were displayed for continuous variables.

Abbreviations: LDLC, low-density lipoprotein cholesterol; HDLC, high-density lipoprotein cholesterol; TG, triglyceride; TC, total cholesterol; GRS, genetic risk score; CAPEC, Chinese Antipsychotics Pharmacogenetics Consortium study.

Table S14. Associations between *GCK, APOC3* GRSs with glucose, lipids in the CAPEC study. Related to Figure 2.

Gene	Trait	Beta	SE	CI	P	P.FDR
GCK	Glucose	0.131	0.956	-1.751 ~ 2.014	0.891	0.891
*APOC3*_TG	TG	-4.131	3.819	-11.651 ~ 3.388	0.280	0.420
*APOC3*_TC	TC	-2.945	2.307	-7.488 ~ 1.598	0.203	0.420

Note: Adjusted for age, age^2, gender, center, first five principal components, course of disease, and previous drug usage.

False discovery rate (FDR)-corrected P-value < 0.05 was applied as the significant threshold accounting for multiple tests across drug targets and outcomes. The conventional P<0.05 was applied as the threshold of suggestive significance.

Abbreviations: GRS, genetic risk score; CAPEC, Chinese Antipsychotics Pharmacogenetics Consortium study; SE, standard error. PANSS, Positive and Negative Syndrome Scale; N, Negative scale; G, General psychopathology scale; P, Positive scale; SE, standard error. LDLC, low-density lipoprotein cholesterol; HDLC, high-density lipoprotein cholesterol; TG, triglyceride; TC, total cholesterol.

Table S15. Conventional observational associations between TG, TC, and PANSS percentage reduction in the CAPOC study. Related to Figure 3.

Exposure	PANSS	Beta	SE	CI	P	P.FDR
TG	PANSS	0.014	0.012	-0.010 ~ 0.037	0.251	0.568
	PANSS_P	0.014	0.010	-0.005 ~ 0.033	0.148	0.568
	PANSS_N	0.012	0.009	-0.006 ~ 0.030	0.187	0.568
	PANSS_G	0.003	0.008	-0.012 ~ 0.018	0.703	0.803
TC	PANSS	-0.007	0.019	-0.044 ~ 0.029	0.692	0.803
	PANSS_P	0.003	0.015	-0.026 ~ 0.033	0.825	0.825
	PANSS_N	0.006	0.014	-0.022 ~ 0.034	0.668	0.803
	PANSS_G	-0.013	0.012	-0.037 ~ 0.011	0.284	0.568

Note: Adjusted for age, age^2, gender, center, course of disease, previous drug usage, and assigned medication.

False discovery rate (FDR)-corrected P-value < 0.05 was applied as the significant threshold accounting for multiple tests across drug targets and outcomes. The conventional P<0.05 was applied as the threshold of suggestive significance.

Abbreviations: PANSS, Positive and Negative Syndrome Scale; N, Negative scale; G, General psychopathology scale; P, Positive scale; SE, standard error. TG, triglyceride; TC, total cholesterol; CAPOC, Chinese Antipsychotics Pharmacogenomics Consortium.

Table S16. Conventional observational associations between lipids and better antipsychotic efficacy in the CAPOC study. Related to Figure 3.

Exposure	PANSS	OR	SE_logOR	CI	P	P.FDR
TG	PANSS	1.001	0.001	1.000 ~ 1.003	0.135	0.419
	PANSS_P	1.001	0.001	0.999 ~ 1.003	0.257	0.514
	PANSS_N	1.000	0.001	0.998 ~ 1.002	0.843	0.994
	PANSS_G	1.000	0.001	0.998 ~ 1.002	0.931	0.994
TC	PANSS	0.998	0.001	0.995 ~ 1.000	0.081	0.419
	PANSS_P	0.998	0.001	0.995 ~ 1.001	0.157	0.419
	PANSS_N	1.001	0.001	0.998 ~ 1.004	0.525	0.840
	PANSS_G	1.000	0.001	0.997 ~ 1.003	0.994	0.994

Note: Adjusted for age, age^2, gender, center, course of disease, previous drug usage, and assigned medication.

Better antipsychotic efficacy: PANSS percentage reduction > median value of PANSS percentage reduction; The standard error (SE) corresponds to the beta coefficient derived from the natural logarithm of the odds ratio (log OR);

False discovery rate (FDR)-corrected P-value < 0.05 was applied as the significant threshold accounting for multiple tests across drug targets and outcomes. The conventional P<0.05 was applied as the threshold of suggestive significance.

Abbreviations: PANSS, Positive and Negative Syndrome Scale; N, Negative scale; G, General psychopathology scale; P, Positive scale; SE, standard error. TG, triglyceride; TC, total cholesterol; CAPOC, Chinese Antipsychotics Pharmacogenomics Consortium.

Table S17. One-sample Mendelian randomization between lipid and PANSS percentage reduction in the CAPOC study. Related to Figure 3.

Exposure	PANSS	Beta	SE	CI	P	P.FDR
TG	PANSS	-0.068	0.072	-0.209 ~ 0.073	0.346	0.635
	PANSS_P	-0.025	0.058	-0.138 ~ 0.089	0.669	0.766
	PANSS_N	-0.160	0.055	-0.268 ~ -0.052	0.004	0.032
	PANSS_G	-0.033	0.047	-0.125 ~ 0.058	0.476	0.635
TC	PANSS	-0.061	0.077	-0.211 ~ 0.090	0.430	0.635
	PANSS_P	-0.009	0.061	-0.129 ~ 0.112	0.890	0.890
	PANSS_N	-0.048	0.059	-0.163 ~ 0.068	0.416	0.635
	PANSS_G	-0.048	0.050	-0.145 ~ 0.050	0.337	0.635

Note: Adjusted for age, age^2, gender, center, course of disease, previous drug usage, assigned medication, first five principal components.

False discovery rate (FDR)-corrected P-value < 0.05 was applied as the significant threshold accounting for multiple tests across drug targets and outcomes. The conventional P<0.05 was applied as the threshold of suggestive significance.

Abbreviations: PANSS, Positive and Negative Syndrome Scale; N, Negative scale; G, General psychopathology scale; P, Positive scale; SE, standard error. TG, triglyceride; TC, total cholesterol; CAPOC, Chinese Antipsychotics Pharmacogenomics Consortium.

Table S18. One-sample Mendelian randomization between lipids and better antipsychotic efficacy in the CAPOC study. Related to Figure 3.

Exposure	PANSS	OR	SE_logOR	CI	P	P.FDR
TG	PANSS	0.993	0.005	0.983 ~ 1.003	0.187	0.499
	PANSS_P	1.001	0.005	0.990 ~ 1.011	0.923	0.923
	PANSS_N	0.979	0.005	0.968 ~ 0.989	3.97E-05	3.18E-04
	PANSS_G	0.999	0.005	0.988 ~ 1.009	0.793	0.906
TC	PANSS	0.990	0.006	0.980 ~ 1.001	0.085	0.340
	PANSS_P	0.995	0.006	0.984 ~ 1.006	0.395	0.632
	PANSS_N	0.996	0.006	0.986 ~ 1.007	0.511	0.681
	PANSS_G	0.995	0.006	0.984 ~ 1.006	0.347	0.632

Note: Adjusted for age, age^2, gender, center, course of disease, previous drug usage, assigned medication, first five principal components.

Better antipsychotic efficacy: PANSS percentage reduction > median value of PANSS percentage reduction; The standard error (SE) corresponds to the beta coefficient derived from the natural logarithm of the odds ratio (log OR).

False discovery rate (FDR)-corrected P-value < 0.05 was applied as the significant threshold accounting for multiple tests across drug targets and outcomes. The conventional P<0.05 was applied as the threshold of suggestive significance.

Abbreviations: PANSS, Positive and Negative Syndrome Scale; N, Negative scale; G, General psychopathology scale; P, Positive scale; TG, triglyceride; TC, total cholesterol; CAPOC, Chinese Antipsychotics Pharmacogenomics Consortium.

Table S19. Associations between lipid-lowering drug-target GRSs and PANSS percentage reduction in the CAPOC study. Related to Figure 3.

Trait	Gene	PANSS	Beta	SE	CI_lower	CI_upper	P	P.FDR
TG	*APOC3*	PANSS	0.745	0.617	-0.464	1.955	0.227	0.908
	APOC3	PANSS_P	0.240	0.495	-0.730	1.211	0.628	0.946
	APOC3	PANSS_N	1.230	0.474	0.301	2.159	0.009	0.324
	APOC3	PANSS_G	0.314	0.400	-0.471	1.099	0.433	0.946
LDLC	*APOB*	PANSS	0.389	0.616	-0.819	1.596	0.528	0.946
	APOB	PANSS_P	0.403	0.494	-0.565	1.371	0.415	0.946
	APOB	PANSS_N	-0.443	0.473	-1.371	0.486	0.350	0.946
	APOB	PANSS_G	0.137	0.399	-0.647	0.920	0.732	0.946
HDLC	*ABCA1*	PANSS	0.242	0.615	-0.963	1.447	0.694	0.946
	ABCA1	PANSS_P	0.202	0.493	-0.765	1.169	0.682	0.946
	ABCA1	PANSS_N	-0.171	0.473	-1.098	0.756	0.718	0.946
	ABCA1	PANSS_G	0.320	0.399	-0.461	1.102	0.422	0.946
	CETP	PANSS	-1.076	0.618	-2.289	0.137	0.082	0.691
	CETP	PANSS_P	-0.825	0.496	-1.798	0.148	0.096	0.691
	CETP	PANSS_N	-0.691	0.476	-1.624	0.242	0.146	0.751
	CETP	PANSS_G	-0.727	0.401	-1.514	0.059	0.070	0.691
	LDLR	PANSS	-0.033	0.610	-1.230	1.164	0.956	0.981
	LDLR	PANSS_P	0.035	0.490	-0.925	0.995	0.942	0.981
	LDLR	PANSS_N	-0.011	0.469	-0.931	0.909	0.981	0.981
	LDLR	PANSS_G	-0.207	0.396	-0.984	0.569	0.601	0.946
	LPL	PANSS	-0.604	0.607	-1.794	0.587	0.320	0.946
	LPL	PANSS_P	-0.762	0.487	-1.717	0.192	0.117	0.702
	LPL	PANSS_N	-0.148	0.467	-1.063	0.767	0.751	0.946
	LPL	PANSS_G	-0.493	0.394	-1.264	0.279	0.211	0.908
TC	*APOB*	PANSS	0.269	0.617	-0.941	1.478	0.663	0.946
	APOB	PANSS_P	0.214	0.495	-0.756	1.184	0.666	0.946
	APOB	PANSS_N	-0.539	0.474	-1.469	0.390	0.256	0.922
	APOB	PANSS_G	0.249	0.400	-0.535	1.033	0.534	0.946
	APOC3	PANSS	0.451	0.622	-0.768	1.671	0.468	0.946
	APOC3	PANSS_P	0.117	0.499	-0.861	1.095	0.815	0.946
	APOC3	PANSS_N	0.955	0.478	0.018	1.891	0.046	0.691
	APOC3	PANSS_G	0.040	0.403	-0.750	0.831	0.920	0.981
	LDLR	PANSS	0.083	0.618	-1.130	1.296	0.893	0.981
	LDLR	PANSS_P	0.137	0.496	-0.836	1.109	0.783	0.946
	LDLR	PANSS_N	0.192	0.475	-0.740	1.125	0.686	0.946
	LDLR	PANSS_G	0.107	0.401	-0.680	0.894	0.790	0.946

Note: Adjusted for age, age^2, gender, center, course of disease, previous drug usage, assigned medication, first five principal components;

False discovery rate (FDR)-corrected P-value < 0.05 was applied as the significant threshold accounting for multiple tests across drug targets and outcomes. The conventional P<0.05 was applied as the threshold of suggestive significance;

Abbreviations: PANSS, Positive and Negative Syndrome Scale; N, Negative scale; G, General psychopathology scale; P, Positive scale; LDLC, low density lipoprotein cholesterol; HDLC, high-density lipoprotein cholesterol; TG, triglyceride; TC, total cholesterol; GRS, genetic risk score; CAPOC, Chinese Antipsychotics Pharmacogenomics Consortium.

Table S20. Associations between lipid-lowering drug-target GRSs and better antipsychotic efficacy in the CAPOC study. Related to Figure 3.

Trait	Gene	PANSS	OR	SE_logOR	CI_lower	CI_upper	P	P.FDR
TG	*APOC3*	PANSS	1.084	0.045	0.992	1.185	0.074	0.459
	APOC3	PANSS_P	0.997	0.045	0.913	1.089	0.952	0.952
	APOC3	PANSS_N	1.156	0.045	1.059	1.263	0.001	0.036
	APOC3	PANSS_G	1.044	0.045	0.955	1.141	0.342	0.675
LDLC	*APOB*	PANSS	1.077	0.045	0.986	1.177	0.102	0.459
	APOB	PANSS_P	1.048	0.045	0.959	1.144	0.300	0.675
	APOB	PANSS_N	1.009	0.045	0.924	1.102	0.840	0.889
	APOB	PANSS_G	1.022	0.045	0.936	1.117	0.627	0.837
HDLC	*ABCA1*	PANSS	1.044	0.045	0.956	1.141	0.338	0.675
	ABCA1	PANSS_P	1.016	0.045	0.931	1.109	0.724	0.869
	ABCA1	PANSS_N	1.009	0.045	0.925	1.102	0.836	0.889
	ABCA1	PANSS_G	1.044	0.045	0.956	1.141	0.334	0.675
	CETP	PANSS	0.947	0.045	0.866	1.035	0.226	0.675
	CETP	PANSS_P	0.920	0.045	0.842	1.005	0.064	0.459
	CETP	PANSS_N	0.926	0.045	0.848	1.012	0.089	0.459
	CETP	PANSS_G	0.927	0.045	0.848	1.013	0.096	0.459
	LDLR	PANSS	0.981	0.045	0.899	1.071	0.674	0.867
	LDLR	PANSS_P	1.022	0.044	0.937	1.115	0.628	0.837
	LDLR	PANSS_N	0.945	0.044	0.866	1.030	0.199	0.675
	LDLR	PANSS_G	0.965	0.045	0.884	1.054	0.430	0.744
	LPL	PANSS	0.969	0.044	0.888	1.057	0.478	0.782
	LPL	PANSS_P	0.912	0.044	0.836	0.995	0.038	0.456
	LPL	PANSS_N	0.958	0.044	0.879	1.045	0.334	0.675
	LPL	PANSS_G	0.960	0.044	0.880	1.047	0.356	0.675
TC	*APOB*	PANSS	1.044	0.045	0.955	1.141	0.345	0.675
	APOB	PANSS_P	1.004	0.045	0.919	1.096	0.932	0.952
	APOB	PANSS_N	0.986	0.045	0.903	1.077	0.755	0.877
	APOB	PANSS_G	1.016	0.045	0.930	1.110	0.721	0.869
	APOC3	PANSS	1.053	0.046	0.963	1.152	0.255	0.675
	APOC3	PANSS_P	1.022	0.045	0.935	1.117	0.627	0.837
	APOC3	PANSS_N	1.134	0.045	1.038	1.240	0.005	0.090
	APOC3	PANSS_G	1.012	0.045	0.926	1.107	0.786	0.884
	LDLR	PANSS	0.978	0.045	0.895	1.069	0.624	0.837
	LDLR	PANSS_P	1.036	0.045	0.948	1.131	0.434	0.744
	LDLR	PANSS_N	0.950	0.045	0.870	1.037	0.254	0.675
	LDLR	PANSS_G	0.975	0.045	0.892	1.065	0.568	0.837

Note: Adjusted for age, age^2, gender, center, course of disease, previous drug usage, assigned medication, first five principal components; Better antipsychotic efficacy: PANSS percentage reduction > median value of PANSS percentage reduction; False discovery rate (FDR)-corrected P-value < 0.05 was applied as the significant threshold accounting for multiple tests across drug targets and outcomes. The conventional P<0.05 was applied as the threshold of suggestive significance. The standard error (SE) corresponds to the beta coefficient derived from the natural logarithm of the odds ratio (log OR).

Abbreviations: PANSS, Positive and Negative Syndrome Scale; N, Negative scale; G, General psychopathology scale; P, Positive scale; LDLC, low density lipoprotein cholesterol; HDLC, high-density lipoprotein cholesterol; TG, triglyceride; TC, total cholesterol; GRS, genetic risk score; CAPOC, Chinese Antipsychotics Pharmacogenomics Consortium;

Table S21. Colocalization analysis between GWASs of glucose and PANSS percentage reduction, and pQTL and PANSS percentage reduction of negative symptoms. Related to Figure 3.

Gene locus	Trait1	Trait2	PP.H3	PP.H4	Causal SNP	SNP.PP.H4
APOC3	TG	PANSS_N	0.013	0.094	rs651821	1.000
APOC3	TC	PANSS_N	0.013	0.094	rs651821	1.000
GCK	Glucose	PANSS	0.007	0.027	rs3757840	1.000
GCK	Glucose	PANSS_P	0.005	0.023	rs3757840	1.000
GCK	Glucose	PANSS_N	0.004	0.180	rs3757840	1.000
GCK	Glucose	PANSS_G	0.006	0.025	rs3757840	1.000
APOC3	APOC3 protein	PANSS_N	0.0002	0.002	rs651821	0.715
GCK	GCK protein	PANSS_N	0.00003	0.001	rs3757840	0.689

Notes: Abbreviations: PP.H3, PP.H4, posterior probability of the H3, H4 hypotheses for the Bayesian colocalization analysis; PANSS, Positive and Negative Syndrome Scale; N, Negative scale; G, General psychopathology scale; P, Positive scale.

Table S22. Conventional observational associations between glucose and PANSS percentage reduction in the CAPOC study. Related to Figure 3.

Exposure	PANSS	Beta	SE	CI	P	P.FDR
Glucose	PANSS	-0.086	0.047	-0.179 ~ 0.007	0.070	0.105
	PANSS_P	-0.029	0.038	-0.104 ~ 0.045	0.440	0.440
	PANSS_N	-0.086	0.036	-0.157 ~ -0.014	0.019	0.076
	PANSS_G	-0.054	0.031	-0.115 ~ 0.006	0.079	0.105

Note: Adjusted for age, age^2, gender, center, course of disease, previous drug usage, assigned medication;

False discovery rate (FDR)-corrected P-value < 0.05 was applied as the significant threshold accounting for multiple tests across drug targets and outcomes. The conventional $P<0.05$ was applied as the threshold of suggestive significance.

Abbreviations: PANSS, Positive and Negative Syndrome Scale; N, Negative scale; G, General psychopathology scale; P, Positive scale; CAPOC, Chinese Antipsychotics Pharmacogenomics Consortium.

Table S23. Conventional observational associations between glucose and better antipsychotic efficacy in the CAPOC study. Related to Figure 3.

Exposure	PANSS	OR	SE_logOR	CI	P	P.FDR
Glucose	PANSS	0.998	0.003	0.991 ~ 1.004	0.501	0.501
	PANSS_P	0.997	0.003	0.990 ~ 1.004	0.386	0.501
	PANSS_N	0.995	0.003	0.988 ~ 1.001	0.126	0.501
	PANSS_G	0.997	0.003	0.990 ~ 1.004	0.391	0.501

Note: Adjusted for age, age^2, gender, center, course of disease, previous drug usage, assigned medication;

False discovery rate (FDR)-corrected P-value < 0.05 was applied as the significant threshold accounting for multiple tests across drug targets and outcomes. The conventional $P<0.05$ was applied as the threshold of suggestive significance. Better antipsychotic efficacy: PANSS percentage reduction > median value of PANSS percentage reduction; The standard error (SE) corresponds to the beta coefficient derived from the natural logarithm of the odds ratio (log OR).

Abbreviations: PANSS, Positive and Negative Syndrome Scale; N, Negative scale; G, General psychopathology scale; P, Positive scale; CAPOC, Chinese Antipsychotics Pharmacogenomics Consortium;

Table S24. One-sample Mendelian randomization between glucose and PANSS percentage reduction in the CAPOC study. Related to Figure 3.

Exposure	PANSS	Beta	SE	CI	P	P.FDR
Glucose	PANSS	0.785	0.323	0.152 ~ 1.418	0.015	0.025
	PANSS_P	0.585	0.259	0.077 ~ 1.092	0.024	0.025
	PANSS_N	0.642	0.248	0.156 ~ 1.128	0.010	0.025
	PANSS_G	0.468	0.209	0.058 ~ 0.879	0.025	0.025

Note: Adjusted for age, age^2, gender, center, course of disease, previous drug usage, assigned medication, first five principal components;

False discovery rate (FDR)-corrected P-value < 0.05 was applied as the significant threshold accounting for multiple tests across drug targets and outcomes. The conventional P<0.05 was applied as the threshold of suggestive significance.

Abbreviations: PANSS, Positive and Negative Syndrome Scale; N, Negative scale; G, General psychopathology scale; P, Positive scale; SE, standard error; CAPOC, Chinese Antipsychotics Pharmacogenomics Consortium.

Table S25. One-sample Mendelian randomization between glucose and better antipsychotic efficacy in the CAPOC study. Related to Figure 3.

Exposure	PANSS	OR	SE_logOR	CI	P	P.FDR
Glucose	PANSS	1.054	0.024	1.006 ~ 1.104	0.027	0.054
	PANSS_P	1.015	0.024	0.969 ~ 1.063	0.523	0.523
	PANSS_N	1.060	0.024	1.013 ~ 1.111	0.013	0.052
	PANSS_G	1.048	0.024	1.000 ~ 1.098	0.049	0.065

Note: Adjusted for age, age^2, gender, center, course of disease, previous drug usage, assigned medication, first five principal components;

False discovery rate (FDR)-corrected P-value < 0.05 was applied as the significant threshold accounting for multiple tests across drug targets and outcomes. The conventional P<0.05 was applied as the threshold of suggestive significance.

Better antipsychotic efficacy: PANSS percentage reduction > median value of PANSS percentage reduction; The standard error (SE) corresponds to the beta coefficient derived from the natural logarithm of the odds ratio (log OR).

Abbreviations: PANSS, Positive and Negative Syndrome Scale; N, Negative scale; G, General psychopathology scale; P, Positive scale; SE, standard error; CAPOC, Chinese Antipsychotics Pharmacogenomics Consortium.

Table S26. Associations between antidiabetic drug-target GRSs and PANSS percentage reduction in the CAPOC study. Related to Figure 3.

Trait	Gene	PANSS	Beta	SE	CI_lower	CI_upper	P	P.FDR
Glucose	*GCK*	PANSS	-1.703	0.614	-2.907	-0.499	0.006	0.016
	GCK	PANSS_P	-1.055	0.493	-2.021	-0.089	0.032	0.064
	GCK	PANSS_N	-1.621	0.472	-2.546	-0.696	0.001	0.008
	GCK	PANSS_G	-1.119	0.398	-1.900	-0.339	0.005	0.016
	ABCB11	PANSS	0.200	0.617	-1.009	1.410	0.745	0.851
	ABCB11	PANSS_P	-0.318	0.495	-1.287	0.652	0.521	0.695
	ABCB11	PANSS_N	0.691	0.474	-0.238	1.621	0.145	0.232
	ABCB11	PANSS_G	-0.023	0.400	-0.807	0.761	0.954	0.954

Note: Adjusted for age, age^2, gender, center, course of disease, previous drug usage, assigned medication, first five principal components;

False discovery rate (FDR)-corrected P-value < 0.05 was applied as the significant threshold accounting for multiple tests across drug targets and outcomes. The conventional P<0.05 was applied as the threshold of suggestive significance.

Abbreviations: PANSS, Positive and Negative Syndrome Scale; N, Negative scale; G, General psychopathology scale; P, Positive scale; SE, standard error; GRS, genetic risk score; CAPOC, Chinese Antipsychotics Pharmacogenomics Consortium.

Table S27. Associations between antidiabetic drug-target GRSs and better antipsychotic efficacy in the CAPOC study. Related to Figure 3.

Trait	Gene	PANSS	OR	SE_logOR	CI_lower	CI_upper	P	P.FDR
Glucose	*GCK*	PANSS	0.917	0.045	0.839	1.002	0.056	0.112
	GCK	PANSS_P	0.896	0.045	0.820	0.978	0.014	0.084
	GCK	PANSS_N	0.901	0.045	0.825	0.984	0.021	0.084
	GCK	PANSS_G	0.912	0.045	0.834	0.996	0.041	0.109
	ABCB11	PANSS	0.987	0.045	0.903	1.078	0.772	0.882
	ABCB11	PANSS_P	0.961	0.045	0.880	1.049	0.376	0.501
	ABCB11	PANSS_N	1.003	0.045	0.919	1.095	0.939	0.939
	ABCB11	PANSS_G	0.934	0.045	0.855	1.020	0.129	0.206

Note: Adjusted for age, age^2, gender, center, course of disease, previous drug usage, assigned medication, first five principal components;

Better antipsychotic efficacy: PANSS percentage reduction > median value of PANSS percentage reduction;

The standard error (SE) corresponds to the beta coefficient derived from the natural logarithm of the odds ratio (log OR).

False discovery rate (FDR)-corrected P-value < 0.05 was applied as the significant threshold accounting for multiple tests across drug targets and outcomes. The conventional P<0.05 was applied as the threshold of suggestive significance.

Abbreviations: PANSS, Positive and Negative Syndrome Scale; N, Negative scale; G, General psychopathology scale; P, Positive scale; SE, standard error; GRS, genetic risk score; CAPOC, Chinese Antipsychotics Pharmacogenomics Consortium.

Table S28. Replication analysis for associations between *APOC3* GRS and PANSS percentage reduction in the CAPEC study. Related to Figure 3.

Gene	Trait	PANSS	Beta	SE	CI_lower	CI_upper	P	P.FDR
APOC3	TG	PANSS	0.505	1.399	-2.250	3.261	0.718	0.927
		PANSS_P	-0.309	1.188	-2.649	2.031	0.795	0.927
		PANSS_N	1.186	1.151	-1.081	3.452	0.304	0.927
		PANSS_G	0.230	1.015	-1.769	2.228	0.821	0.927
APOC3	TC	PANSS	0.133	1.450	-2.722	2.989	0.927	0.927
		PANSS_P	-0.754	1.231	-3.177	1.669	0.541	0.927
		PANSS_N	1.578	1.191	-0.768	3.923	0.186	0.927
		PANSS_G	-0.174	1.052	-2.245	1.897	0.869	0.927

Note: Adjusted for age, age^2, gender, center, course of disease, previous drug usage, assigned medication, first five principal components;

False discovery rate (FDR)-corrected P-value < 0.05 was applied as the significant threshold accounting for multiple tests across drug targets and outcomes. The conventional P<0.05 was applied as the threshold of suggestive significance.

Abbreviations: PANSS, Positive and Negative Syndrome Scale; N, Negative scale; G, General psychopathology scale; P, Positive scale; SE, standard error; GRS, genetic risk score; TG, triglyceride; TC, total cholesterol; CAPEC, Chinese Antipsychotics Pharmacogenetics Consortium.

Table S29. Replication analysis for associations between *GCK* GRS and PANSS percentage reduction in the CAPEC study. Related to Figure 3.

Gene	Trait	PANSS	Beta	SE	CI_lower	CI_upper	P	P.FDR
GCK	Glucose	PANSS	-3.200	1.327	-5.813	-0.587	0.017	0.034
		PANSS_P	-2.272	1.131	-4.498	-0.045	0.046	0.061
		PANSS_N	-2.706	1.093	-4.858	-0.554	0.014	0.034
		PANSS_G	-1.732	0.967	-3.636	0.173	0.075	0.075

Note: Adjusted for age, age^2, gender, center, course of disease, previous drug usage, assigned medication, first five principal components; False discovery rate (FDR)-corrected P-value < 0.05 was applied as the significant threshold accounting for multiple tests across drug targets and outcomes. The conventional P<0.05 was applied as the threshold of suggestive significance.

Abbreviations: PANSS, Positive and Negative Syndrome Scale; N, Negative scale; G, General psychopathology scale; P, Positive scale; SE, standard error; GRS, genetic risk score; CAPEC, Chinese Antipsychotics Pharmacogenetics Consortium.

Table S30. Replication analysis for associations between *APOC3* GRSs and better antipsychotic efficacy in the CAPEC study. Related to Figure 3.

Gene	Trait	PANSS	OR	SE_logOR	CI_lower	CI_upper	P	P.FDR
APOC3	TG	PANSS	1.090	0.131	0.844	1.414	0.510	0.808
		PANSS_P	0.959	0.128	0.745	1.233	0.743	0.811
		PANSS_N	1.208	0.133	0.933	1.573	0.155	0.740
		PANSS_G	0.886	0.129	0.687	1.141	0.350	0.808
APOC3	TC	PANSS	1.072	0.135	0.824	1.401	0.606	0.808
		PANSS_P	0.969	0.133	0.746	1.258	0.811	0.811
		PANSS_N	1.200	0.137	0.918	1.578	0.185	0.740
		PANSS_G	0.900	0.133	0.692	1.168	0.430	0.808

Note: Adjusted for age, age^2, gender, center, course of disease, previous drug usage, assigned medication, first five principal components;

Better antipsychotic efficacy: PANSS percentage reduction > median value of PANSS percentage reduction;

The standard error (SE) corresponds to the beta coefficient derived from the natural logarithm of the odds ratio (log OR).

False discovery rate (FDR)-corrected P-value < 0.05 was applied as the significant threshold accounting for multiple tests across drug targets and outcomes. The conventional P<0.05 was applied as the threshold of suggestive significance.

Abbreviations: PANSS, Positive and Negative Syndrome Scale; N, Negative scale; G, General psychopathology scale; P, Positive scale; SE, standard error; GRS, genetic risk score; TG, triglyceride; TC, total cholesterol; CAPEC, Chinese Antipsychotics Pharmacogenetics Consortium.

Table S31. Replication analysis for associations between *GCK* GRS and better antipsychotic efficacy in the CAPEC study. Related to Figure 3.

Gene	Trait	PANSS	OR	SE_logOR	CI_lower	CI_upper	P	P.FDR
GCK	Glucose	PANSS	0.735	0.127	0.570	0.941	0.016	0.032
		PANSS_P	0.760	0.124	0.593	0.967	0.027	0.036
		PANSS_N	0.686	0.130	0.528	0.882	0.004	0.016
		PANSS_G	0.831	0.124	0.649	1.058	0.135	0.135

Note: Adjusted for age, age^2, gender, center, course of disease, previous drug usage, assigned medication, first five principal components; Better antipsychotic efficacy: PANSS percentage reduction > median value of PANSS percentage reduction;

The standard error (SE) corresponds to the beta coefficient derived from the natural logarithm of the odds ratio (log OR).

False discovery rate (FDR)-corrected P-value < 0.05 was applied as the significant threshold accounting for multiple tests across drug targets and outcomes. The conventional P<0.05 was applied as the threshold of suggestive significance.

Abbreviations: PANSS, Positive and Negative Syndrome Scale; N, Negative scale; G, General psychopathology scale; P, Positive scale; SE, standard error; GRS, genetic risk score; CAPEC, Chinese Antipsychotics Pharmacogenetics Consortium.

Table S32. Replication analysis via two-sample MR methods for associations between *APOC3*-lowering TG, *GCK*-lowering glucose on the PANSS percentage reduction. Related to Figure 3.

Exposure	PANSS	Method	nSNP	Beta	SE	CI_Lower	CI_Upper	P	P.FDR
**Main analysis-Inverse variance weighted**									
*APOC3*_TG	PANSS	Inverse variance weighted	13	3.358	2.561	-1.662	8.378	0.1898	0.3254
	PANSS_P	Inverse variance weighted	13	0.642	2.111	-3.496	4.779	0.7612	0.8459
	PANSS_N	Inverse variance weighted	13	6.680	1.968	2.822	10.538	0.0007	0.0021
	PANSS_G	Inverse variance weighted	13	1.239	1.661	-2.017	4.495	0.4556	0.6075
*APOC3*_TC	PANSS	Inverse variance weighted	4	10.108	11.204	-11.853	32.068	0.3670	0.5505
	PANSS_P	Inverse variance weighted	4	2.564	8.987	-15.051	20.179	0.7754	0.8459
	PANSS_N	Inverse variance weighted	4	21.374	8.611	4.497	38.251	0.0131	0.0262
	PANSS_G	Inverse variance weighted	4	0.944	7.931	-14.601	16.488	0.9053	0.9053
*GCK*_Glucose	PANSS	Inverse variance weighted	6	-20.419	5.684	-31.560	-9.279	0.0003	0.0012
	PANSS_P	Inverse variance weighted	6	-12.656	4.560	-21.594	-3.717	0.0055	0.0132
	PANSS_N	Inverse variance weighted	6	-19.481	4.370	-28.045	-10.917	8.26E-06	0.0001
	PANSS_G	Inverse variance weighted	6	-13.630	3.688	-20.858	-6.401	0.0002	0.0012
**Sensitivity analysis - Weighted Median**									
**Exposure**	**PANSS**	**Method**	**nSNP**	**Beta**	**SE**	**CI_Lower**	**CI_Upper**	**P**	**P.FDR**
*APOC3*_TG	PANSS	Weighted median	13	1.901	2.956	-3.892	7.694	0.5201	0.7802
	PANSS_P	Weighted median	13	-0.018	2.433	-4.788	4.752	0.9941	0.9941
	PANSS_N	Weighted median	13	4.889	2.245	0.489	9.289	0.0294	0.0588
	PANSS_G	Weighted median	13	0.576	2.044	-3.431	4.582	0.7783	0.9449
*APOC3*_TC	PANSS	Weighted median	4	9.442	13.376	-16.774	35.658	0.4802	0.7802
	PANSS_P	Weighted median	4	2.720	10.087	-17.050	22.490	0.7874	0.9449
	PANSS_N	Weighted median	4	23.627	9.968	4.090	43.165	0.0178	0.0427
	PANSS_G	Weighted median	4	0.461	8.625	-16.444	17.367	0.9573	0.9941
*GCK*_Glucose	PANSS	Weighted median	6	-21.700	6.770	-34.970	-8.430	0.0013	0.0052
	PANSS_P	Weighted median	6	-15.874	5.388	-26.434	-5.313	0.0032	0.0096
	PANSS_N	Weighted median	6	-21.683	5.448	-32.361	-11.006	0.0001	0.0012
	PANSS_G	Weighted median	6	-15.437	4.433	-24.125	-6.749	0.0005	0.0030

Note: False discovery rate (FDR)-corrected P-value < 0.05 was applied as the significant threshold accounting for multiple tests across drug targets and outcomes. The conventional P<0.05 was applied as the threshold of suggestive significance. Abbreviations: PANSS, Positive and Negative Syndrome Scale; N, Negative scale; G, General psychopathology scale; P, Positive scale; SE, standard error; GRS, genetic risk score; TG, triglyceride; TC, total cholesterol.

Table S33. Test for the replication analysis via two-sample MR methods for associations between *APOC3*-lowering TG, *GCK*-lowering glucose on the PANSS percentage reduction. Related to Figure 3.

Exposure	PANSS	Egger.inter	Egger.SE	P.inter	MR-PRESSO Global test.P	MR-PRESSO N_outlier	*Q*	P.heterogeneity
*APOC3*_TG	PANSS	0.107	0.630	0.868	0.552	0	11.763	0.465
	PANSS_P	-0.143	0.523	0.789	0.728	0	12.673	0.393
	PANSS_N	0.581	0.469	0.241	0.547	0	7.126	0.849
	PANSS_G	-0.078	0.407	0.850	0.608	0	11.659	0.473
*APOC3*_TC	PANSS	-0.739	1.445	0.660	0.431	0	1.471	0.689
	PANSS_P	-0.473	1.159	0.723	0.805	0	0.764	0.858
	PANSS_N	-1.021	1.111	0.455	0.888	0	1.333	0.721
	PANSS_G	-0.889	1.083	0.498	0.762	0	3.572	0.312
*GCK*_Glucose	PANSS	0.137	1.088	0.906	0.837	0	2.556	0.768
	PANSS_P	0.133	0.873	0.886	0.742	0	1.923	0.860
	PANSS_N	0.012	0.837	0.990	0.845	0	2.568	0.766
	PANSS_G	-0.194	0.705	0.796	0.798	0	2.143	0.829

Note: A test for intercepts of MR-Egger (ME) regression was used to indicate the degree of directional horizontal pleiotropy. MR-Egger regression was performed to test for horizontal pleiotropy. Mendelian randomization pleiotropy residual sum and outlier (MR-PRESSO) was applied. SNPs that were detected to be the outliers (P < 0.05) were excluded to minimize pleiotropy bias. Inverse variance weighted Cochrane's *Q* test was performed for the test of heterogeneity.

Abbreviations: PANSS, Positive and Negative Syndrome Scale; N, Negative scale; G, General psychopathology scale; P, Positive scale; SE, standard error; GRS, genetic risk score; TG, triglyceride; TC, total cholesterol.

Table S34. Associations between lipid- and glucose-lowering target pQTL-GRSs and levels of measured lipid and glucose in the CAPOC study. Related to Figure 3.

Gene	Base sample	Trait	Beta	SE	CI_lower	CI_upper	P	P.FDR
APOC3	GNHS-pQTL in blood	TG	-6.010	1.115	-8.195	-3.825	7.65E-08	2.30E-07
APOC3	GNHS-pQTL in blood	TC	-1.821	0.712	-3.217	-0.426	0.011	0.011
GCK	Multi-cohorts-pQTL in CSF	Glucose	-0.748	0.283	-1.303	-0.193	0.008	0.011

Note: Adjusted for age, age^2, gender, center, first five principal components, course of disease, previous drug usage;

False discovery rate (FDR)-corrected P-value < 0.05 was applied as the significant threshold accounting for multiple tests across drug targets, lipids, and glucose. The conventional P<0.05 was applied as the threshold of suggestive significance.

Abbreviations: SE, standard error; pQTL, protein quantitative trait loci; CSF, cerebrospinal fluid; GNHS, Guangzhou Nutrition and Health study; GRS, genetic risk score; CAPOC, Chinese Antipsychotics Pharmacogenomics Consortium.

Table S35. Associations between APOC3 blood-pQTL-GRSs and PANSS percentage reduction in the CAPOC study. Related to Figure 3.

PANSS percentage reduction	Beta	SE	CI_lower	CI_upper	P	P.FDR
PANSS	0.381	0.615	-0.825	1.586	0.536	0.948
PANSS_P	0.032	0.494	-0.936	1.000	0.948	0.948
PANSS_N	0.877	0.471	-0.047	1.802	0.063	0.252
PANSS_G	4.11E-02	0.398	-0.740	0.822	0.918	0.948

Note: Adjusted for age, age^2, gender, center, first five principal components, course of disease, previous drug usage, assigned medication; False discovery rate (FDR)-corrected P-value < 0.05 was applied as the significant threshold accounting for multiple tests across drug targets and outcomes. The conventional P<0.05 was applied as the threshold of suggestive significance.

Abbreviations: PANSS, Positive and Negative Syndrome Scale; N, Negative scale; G, General psychopathology scale; P, Positive scale; SE, standard error; GRS, genetic risk score; pQTL, protein quantitative trait loci; CAPOC, Chinese Antipsychotics Pharmacogenomics Consortium.

Table S36. Associations between APOC3 blood-pQTL-GRSs and better antipsychotic efficacy in the CAPOC study. Related to Figure 3.

PANSS percentage reduction	OR	SE_logOR	OR_lower	OR_upper	P	P.FDR
PANSS	1.069	0.045	0.979	1.167	0.138	0.276
PANSS_P	0.989	0.044	0.906	1.079	0.800	0.800
PANSS_N	1.113	0.045	1.020	1.215	0.016	0.064
PANSS_G	1.013	0.045	0.928	1.106	0.767	0.800

Note: Adjusted for age, age^2, gender, center, first five principal components, course of disease, previous drug usage, assigned medication;

Better antipsychotic efficacy: PANSS percentage reduction > median value of PANSS percentage reduction;

The standard error (SE) corresponds to the beta coefficient derived from the natural logarithm of the odds ratio (log OR).

False discovery rate (FDR)-corrected P-value < 0.05 was applied as the significant threshold accounting for multiple tests across drug targets and outcomes. The conventional P<0.05 was applied as the threshold of suggestive significance.

Abbreviations: PANSS, Positive and Negative Syndrome Scale; N, Negative scale; G, General psychopathology scale; P, Positive scale; SE, standard error; GRS, genetic risk score; pQTL, protein quantitative trait loci; CAPOC, Chinese Antipsychotics Pharmacogenomics Consortium.

Table S37. Associations between GCK CSF-pQTL-GRSs and PANSS percentage reduction in the CAPOC study. Related to Figure 3.

PANSS	Beta	SE	CI	P	P.FDR
PANSS	-0.509	0.616	-1.716 ~ 0.699	0.409	0.579
PANSS_P	-0.274	0.494	-1.243 ~ 0.694	0.579	0.579
PANSS_N	-1.058	0.473	-1.986 ~ -0.130	0.025	0.102
PANSS_G	-0.298	0.400	-1.081 ~ 0.486	0.456	0.579

Note: Adjusted for age, age^2, gender, center, first five principal components, course of disease, previous drug usage, assigned medication;

False discovery rate (FDR)-corrected P-value < 0.05 was applied as the significant threshold accounting for multiple tests across drug targets and outcomes. The conventional P<0.05 was applied as the threshold of suggestive significance.

Abbreviations: PANSS, Positive and Negative Syndrome Scale; N, Negative scale; G, General psychopathology scale; P, Positive scale; SE, standard error; GRS, genetic risk score; pQTL, protein quantitative trait loci; CSF, cerebrospinal fluid; CAPOC, Chinese Antipsychotics Pharmacogenomics Consortium.

Table S38. Associations between GCK CSF-pQTL-GRSs and better antipsychotic efficacy in the CAPOC study. Related to Figure 3.

PANSS	OR	SE_logOR	CI	P	P.FDR
PANSS	0.966	0.045	0.884 ~ 1.055	0.437	0.583
PANSS_P	0.955	0.045	0.874 ~ 1.042	0.300	0.583
PANSS_N	0.941	0.045	0.862 ~ 1.027	0.171	0.583
PANSS_G	1.019	0.045	0.933 ~ 1.113	0.680	0.680

Note: Adjusted for age, age^2, gender, center, first five principal components, course of disease, previous drug usage, assigned medication;

Better antipsychotic efficacy: PANSS percentage reduction > median value of PANSS percentage reduction; The standard error (SE) corresponds to the beta coefficient derived from the natural logarithm of the odds ratio (log OR). False discovery rate (FDR)-corrected P-value < 0.05 was applied as the significant threshold accounting for multiple tests across drug targets and outcomes. The conventional P<0.05 was applied as the threshold of suggestive significance.

Abbreviations: PANSS, Positive and Negative Syndrome Scale; N, Negative scale; G, General psychopathology scale; P, Positive scale; SE, standard error; GRS, genetic risk score; pQTL, protein quantitative trait loci; CSF, cerebrospinal fluid; CAPOC, Chinese Antipsychotics Pharmacogenomics Consortium.

Table S39. Mediation analysis between pQTL-GRS, lipids and glucose, and the antipsychotic efficacy. Related to Figure 3.

Term	OR	SE_logOR*	95%CI_Lower	95%CI_Upper	P
**APOC3 pQTL associated with better antipsychotic efficacy of PANSS_N**					
pQTL-weighted APOC3 score (per 1 unit lower APOC3 protein levels)	1.103	0.044	1.012	1.202	0.026
**APOC3 pQTL associated with better antipsychotic efficacy of PANSS_N, mediated through TG**					
pQTL-weighted APOC3 score (per 1 unit lower APOC3 protein levels)	0.880	0.105	0.716	1.081	0.222
TG-weighted APOC3 score (per 1 unit lower TG through APOC3)	1.299	0.106	1.055	1.598	0.014
**APOC3 pQTL associated with better antipsychotic efficacy of PANSS_N, mediated through TC**					
pQTL-weighted APOC3 score (per 1 unit lower APOC3 protein levels)	1.041	0.066	0.915	1.185	0.540
TC-weighted APOC3 score (per 1 unit lower TC through APOC3)	1.096	0.067	0.962	1.248	0.170

Term	Beta	SE	95%CI_Lower	95%CI_Upper	P
**GCK pQTL associated with decreased percentage reduction of PANSS_N**					
pQTL-weighted GCK score (per 1 unit lower GCK protein levels)	-1.058	0.473	-1.986	-0.130	0.025
**GCK pQTL associated with decreased percentage reduction of PANSS_N, mediated through glucose**					
pQTL-weighted GCK score (per 1 unit lower GCK protein levels)	-0.498	0.513	-1.505	0.508	0.332
Glucose-weighted GCK score (per 1 unit lower glucose through GCK)	-1.426	0.513	-2.431	-0.421	0.005

Note: Adjusted for age, age^2, gender, center, first five principal components, course of disease, previous drug usage, assigned medication;

Better antipsychotic efficacy: PANSS percentage reduction > median value of PANSS percentage reduction; The standard error (SE) corresponds to the beta coefficient derived from the natural logarithm of the odds ratio (log OR).

Abbreviations: PANSS, Positive and Negative Syndrome Scale; N, Negative scale; G, General psychopathology scale; P, Positive scale; SE, standard error; GRS, genetic risk score; pQTL, protein quantitative trait loci.

Table S40. Bulk tissue gene expression (median TPM) for *APOC3* and *GCK*. Related to Figure 3.

Gene	Pancreas	Liver	Adipose - Visceral (Omentum)	Pituitary	Muscle - Skeletal	Brain - Cortex (BA9)
GCK	0.511	0.857	0.425	28.56	0.033	2.125
APOC3	0.351	8122	0.302	0.395	0.035	0.083
**Gene**	**Brain - Caudate (basal ganglia)**	**Brain - Nucleus accumbens (basal ganglia)**	**Brain - Cerebellum**	**Brain - Hypothalamus**	**Small Intestine - Terminal Ileum**	**Artery - Coronary**
GCK	1.248	1.887	6.077	5.484	0.280	1.230
APOC3	0.036	0	0.371	0	112	0.198

Note: Based on GTEx v10 database; TPM, Transcripts Per Million.

Table S41. Associations between *APOC3, GCK* and neural, metabolic-related traits. Related to Figure 3.

**Gene**	**Top SNP**	**EA of lipid- or glucose-lowering**	**T2D_Beta**	**T2D_SE**	**T2D_P**	**2h_OGTT_Beta**	**2h_OGTT_SE**	**2h_OGTT_P**
GCK	rs2908289	G	-0.017	0.008	0.035	-0.068	0.016	3.74E-05
APOC3	rs651821	T	-0.006	0.007	0.375	0.021	0.014	0.129

**Gene**	**Top SNP**	**EA of lipid- or glucose-lowering**	**HbA1C_Beta**	**HbA1C_SE**	**HbA1C_P**	**Insulin_Beta**	**Insulin_SE**	**Insulin_P**
GCK	rs2908289	G	-0.097	0.005	1.91E-74	-0.002	0.006	0.777
APOC3	rs651821	T	0.006	0.005	0.204	-0.002	0.005	0.599

**Gene**	**Top SNP**	**EA of lipid- or glucose-lowering**	**BMI_Beta**	**BMI_SE**	**BMI_P**	**CAD_Beta**	**CAD_SE**	**CAD_P**
GCK	rs2908289	G	0.004	0.005	0.400	-0.026	0.013	0.041
APOC3	rs651821	T	0.012	0.004	0.001	-0.059	0.010	1.21E-08

**Gene**	**Top SNP**	**EA of lipid- or glucose-lowering**	**MetS_Beta**	**MetS_SE**	**MetS_P**	**Hyperlipidemia_Beta**	**Hyperlipidemia_SE**	**Hyperlipidemia_P**
GCK	rs2908289	G	-0.042	0.019	0.027	-0.022	0.008	0.001
APOC3	rs651821	T	-0.367	0.016	6.72E-116	-0.208	0.012	4.37E-86

**Gene**	**Top SNP**	**EA of lipid- or glucose-lowering**	**Sphingomyelin_Beta**	**Sphingomyelin_SE**	**Sphingomyelin_P**	**Beta_Endorphin_Beta**	**Beta_Endorphin_SE**	**Beta_Endorphin_P**
GCK	rs2908289	G	0.014	0.005	0.004	0.007	0.030	0.819
APOC3	rs651821	T	0.039	0.008	1.80E-06	-0.442	0.040	2.24E-28

**Gene**	**Top SNP**	**EA of lipid- or glucose-lowering**	**SCZ_Beta**	**SCZ_SE**	**SCZ_P**
GCK	rs2908289	G	0.143	0.186	0.443
APOC3	rs651821	T	0.328	0.151	0.030

Notes: The effect size referred to the association between lipid- or glucose-lowering allele and the corresponding trait. Abbreviations: T2D, type 2 diabetes; 2h OGTT, 2-Hour Oral Glucose Tolerance Test; HbA1c, Hemoglobin A1c; BMI, body mass index; CAD, coronary artery disease; MetS, metabolic syndrome; SCZ, schizophrenia; EA, effect allele.

Table S42. Associations between *APOC3, GCK* GRSs with laboratory and physical examination measures in the CAPOC study. Related to Figure 3.

GRS	Outcome	Beta	SE	95%CI	P	P.FDR
APOC3_TG	SBP (mmHg)	-0.405	0.237	-0.868 ~ 0.059	0.087	0.316
	DBP (mmHg)	-0.280	0.173	-0.619 ~ 0.059	0.106	0.316
	Pulse (/min)	-0.065	0.222	-0.500 ~ 0.370	0.770	0.866
	ALT (IU/L)	0.815	0.382	0.067 ~ 1.563	0.033	0.316
	AST (IU/L)	0.232	0.301	-0.357 ~ 0.822	0.440	0.625
	BUN (mmol/L)	-0.644	0.518	-1.659 ~ 0.371	0.214	0.480
	CRE (mmol/L)	-0.155	0.483	-1.103 ~ 0.792	0.748	0.866
	PRL (uIU/mL)	-3.260	27.243	-56.657 ~ 50.136	0.905	0.953
	QTc interval (ms)	-1.148	0.650	-2.422 ~ 0.126	0.078	0.316
APOC3_TC	SBP (mmHg)	-0.154	0.238	-0.621 ~ 0.312	0.517	0.698
	DBP (mmHg)	-0.144	0.174	-0.485 ~ 0.197	0.407	0.625
	Pulse (/min)	-0.219	0.223	-0.657 ~ 0.218	0.325	0.585
	ALT (IU/L)	0.950	0.384	0.198 ~ 1.702	0.013	0.316
	AST (IU/L)	0.544	0.302	-0.049 ~ 1.136	0.072	0.316
	BUN (mmol/L)	-0.872	0.521	-1.892 ~ 0.148	0.094	0.316
	CRE (mmol/L)	-0.029	0.486	-0.981 ~ 0.924	0.953	0.953
	PRL (uIU/mL)	-42.384	26.986	-95.277 ~ 10.509	0.117	0.316
	QTc interval (ms)	-0.832	0.652	-2.110 ~ 0.447	0.202	0.480
*GCK*_Glucose	SBP (mmHg)	-0.114	0.236	-0.576 ~ 0.349	0.630	0.810
	DBP (mmHg)	0.015	0.173	-0.323 ~ 0.353	0.930	0.953
	Pulse (/min)	0.265	0.221	-0.168 ~ 0.698	0.231	0.480
	ALT (IU/L)	-0.606	0.381	-1.352 ~ 0.140	0.111	0.316
	AST (IU/L)	-0.316	0.300	-0.904 ~ 0.272	0.292	0.563
	BUN (mmol/L)	-0.813	0.517	-1.827 ~ 0.200	0.116	0.316
	CRE (mmol/L)	-0.177	0.483	-1.123 ~ 0.769	0.714	0.866
	PRL (uIU/mL)	24.903	27.285	-28.575 ~ 78.382	0.362	0.611
	QTc interval (ms)	0.518	0.650	-0.755 ~ 1.791	0.425	0.625

Notes: Adjusted for age, age^2, gender, center, first five principal components, course of disease, previous drug usage.

False discovery rate (FDR)-corrected P-value < 0.05 was applied as the significant threshold accounting for multiple tests across drug targets and outcomes. The conventional P<0.05 was applied as the threshold of suggestive significance.

Abbreviations: SBP, systolic blood pressure; DBP, diastolic blood pressure; BMI, body mass index; QTc, corrected QT interval; ALT, alanine aminotransferase; AST, aspartate aminotransferase; BUN, blood urea nitrogen; CRE, creatinine; PRL, prolactin; HbA1c, Glycated hemoglobin A1c; SE, standard error; CAPOC, Chinese Antipsychotics Pharmacogenomics Consortium.

Table S45. Replication for the subgroup associations between *GCK* GRS and PANSS percentage reduction in the CAPEC study. Related to Figure 4.

First episode or recurrent														
PANSS	First episode (N=94)						Recurrent (N=198)						P.inter	P.inter.FDR
	Beta	SE	CI_lower	CI_upper	P	P.FDR	Beta	SE	CI_lower	CI_upper	P	P.FDR		
PANSS	-5.705	2.675	-11.042	-0.368	0.037	0.074	-1.885	1.528	-4.901	1.131	0.219	0.397	0.194	0.260
PANSS_P	-4.297	2.363	-9.011	0.417	0.073	0.092	-1.141	1.274	-3.655	1.374	0.372	0.397	0.217	0.260
PANSS_N	-5.398	2.294	-9.973	-0.822	0.021	0.074	-1.507	1.226	-3.927	0.913	0.221	0.397	0.127	0.260
PANSS_G	-3.323	1.944	-7.201	0.555	0.092	0.092	-0.946	1.113	-3.144	1.252	0.397	0.397	0.260	0.260

Antipsychotics with stronger or milder metabolic effects														
PANSS	Stronger (N=124)						Milder (N=168)						P.inter	P.inter.FDR
	Beta	SE	CI_lower	CI_upper	P	P.FDR	Beta	SE	CI_lower	CI_upper	P	P.FDR		
PANSS	-3.520	2.081	-7.645	0.605	0.094	0.231	-2.535	1.724	-5.942	0.872	0.144	0.231	0.690	0.992
PANSS_P	-2.239	1.858	-5.921	1.444	0.231	0.231	-2.008	1.465	-4.904	0.888	0.173	0.231	0.744	0.992
PANSS_N	-2.946	1.896	-6.706	0.813	0.123	0.231	-2.494	1.394	-5.248	0.261	0.076	0.231	0.996	0.996
PANSS_G	-2.181	1.648	-5.448	1.087	0.189	0.231	-1.163	1.214	-3.562	1.237	0.340	0.340	0.625	0.992

Baseline diabetes status														
PANSS	With prediabetics or diabetes (N=44)						Without prediabetes or diabetes (N=248)						P.inter	P.inter.FDR
	Beta	SE	CI_lower	CI_upper	P	P.FDR	Beta	SE	CI_lower	CI_upper	P	P.FDR		
PANSS	-7.351	4.058	-15.664	0.963	0.081	0.156	-3.048	1.414	-5.836	-0.261	0.032	0.064	0.401	0.748
PANSS_P	-4.421	3.058	-10.685	1.844	0.159	0.159	-2.359	1.244	-4.812	0.093	0.059	0.079	0.830	0.830
PANSS_N	-4.985	3.084	-11.302	1.332	0.117	0.156	-2.682	1.186	-5.019	-0.345	0.025	0.064	0.561	0.748
PANSS_G	-5.919	3.213	-12.501	0.663	0.076	0.156	-1.401	1.025	-3.422	0.620	0.173	0.173	0.191	0.748

Gender														
PANSS	Male (N=132)						Female (N=160)						P.inter	P.inter.FDR
	Beta	SE	CI_lower	CI_upper	P	P.FDR	Beta	SE	CI_lower	CI_upper	P	P.FDR		
PANSS	-4.506	1.998	-8.468	-0.545	0.026	0.045	-0.955	1.790	-4.494	2.583	0.594	0.792	0.323	0.646
PANSS_P	-3.746	1.643	-7.003	-0.489	0.025	0.045	-0.073	1.559	-3.155	3.010	0.963	0.963	0.165	0.646
PANSS_N	-3.423	1.595	-6.584	-0.261	0.034	0.045	-1.507	1.523	-4.518	1.503	0.324	0.792	0.676	0.901
PANSS_G	-1.698	1.425	-4.522	1.127	0.236	0.236	-1.083	1.359	-3.770	1.604	0.427	0.792	0.952	0.952

Note: Adjusted for age, age^2, gender, center, first five principal components, course of disease, previous drug usage, assigned medication;

Patients with baseline glucose >= 100.8 mg/dL (5.6 mmol/L) were classified into the prediabetes or diabetes group, the remaining patients were in the non-diabetes or prediabetes group;

Individuals with < 2 years of first-episode SCZ course, and < 14 days of antipsychotic exposure, were classified as first-episode, drug-naïve. The remaining patients were categorized as recurrent. Patients prescribed risperidone, olanzapine, quetiapine, or clozapine were grouped as receiving medication with stronger metabolic side effects; Others (prescribing aripiprazole, ziprasidone, or perphenazine) comprised the milder-metabolic-effect group.

False discovery rate (FDR)-corrected P-value < 0.05 was applied as the significant threshold accounting for multiple tests across drug targets and outcomes. The conventional P<0.05 was applied as the threshold of suggestive significance.

Abbreviations: PANSS, Positive and Negative Syndrome Scale; N, Negative scale; G, General psychopathology scale; P, Positive scale; SE, standard error; GRS, genetic risk score; CAPEC, Chinese Antipsychotics Pharmacogenetics Consortium.

Table S46. Replication for the subgroup associations between *GCK* GRS and better antipsychotic efficacy in the CAPEC study. Related to Figure 4.

First episode or recurrent

PANSS	First episode (N=94)						Recurrent (N=198)						P.inter	P.inter.FDR
	OR	SE_logOR	CI_lower	CI_upper	P	P.FDR	OR	SE_logOR	CI_lower	CI_upper	P	P.FDR		
PANSS	0.416	0.308	0.213	0.726	0.004	0.011	0.862	0.154	0.636	1.166	0.336	0.559	0.122	0.244
PANSS_P	0.473	0.277	0.262	0.788	0.007	0.011	0.917	0.148	0.685	1.226	0.559	0.559	0.052	0.208
PANSS_N	0.452	0.297	0.239	0.779	0.008	0.011	0.774	0.152	0.571	1.040	0.092	0.368	0.183	0.244
PANSS_G	0.713	0.243	0.434	1.137	0.164	0.164	0.900	0.152	0.666	1.212	0.487	0.559	0.465	0.465

Antipsychotics with stronger or milder metabolic effects

PANSS	Stronger (N=124)						Milder (N=168)						P.inter	P.inter.FDR
	OR	SE_logOR	CI_lower	CI_upper	P	P.FDR	OR	SE_logOR	CI_lower	CI_upper	P	P.FDR		
PANSS	0.616	0.244	0.374	0.982	0.047	0.188	0.802	0.154	0.589	1.083	0.153	0.204	0.505	0.727
PANSS_P	0.838	0.222	0.537	1.294	0.428	0.428	0.734	0.155	0.537	0.991	0.047	0.094	0.727	0.727
PANSS_N	0.765	0.239	0.473	1.216	0.262	0.349	0.655	0.162	0.471	0.894	0.009	0.036	0.356	0.727
PANSS_G	0.762	0.236	0.475	1.205	0.250	0.349	0.897	0.152	0.664	1.207	0.474	0.474	0.616	0.727

Baseline diabetes status

PANSS	With prediabetics or diabetes (N=44)						Without prediabetes or diabetes (N=248)						P.inter	P.inter.FDR
	OR	SE_logOR	CI_lower	CI_upper	P	P.FDR	OR	SE_logOR	CI_lower	CI_upper	P	P.FDR		
PANSS	0.419	0.604	0.103	1.248	0.150	0.157	0.728	0.138	0.552	0.950	0.021	0.042	0.561	0.649
PANSS_P	0.405	0.569	0.109	1.125	0.112	0.157	0.749	0.137	0.570	0.975	0.034	0.045	0.649	0.649
PANSS_N	0.445	0.572	0.123	1.279	0.157	0.157	0.700	0.140	0.529	0.915	0.010	0.040	0.391	0.649
PANSS_G	0.146	1.023	0.009	0.688	0.060	0.157	0.861	0.134	0.659	1.119	0.266	0.266	0.130	0.520

Gender

PANSS	Male (N=132)						Female (N=160)						P.inter	P.inter.FDR
	OR	SE_logOR	CI_lower	CI_upper	P	P.FDR	OR	SE_logOR	CI_lower	CI_upper	P	P.FDR		
PANSS	0.643	0.217	0.410	0.969	0.042	0.056	0.853	0.177	0.601	1.205	0.368	0.491	0.593	0.791
PANSS_P	0.610	0.212	0.393	0.910	0.020	0.056	0.899	0.173	0.638	1.262	0.537	0.537	0.419	0.791
PANSS_N	0.623	0.221	0.395	0.947	0.032	0.056	0.743	0.180	0.518	1.052	0.098	0.392	0.917	0.917
PANSS_G	0.911	0.197	0.613	1.340	0.635	0.635	0.822	0.171	0.585	1.147	0.251	0.491	0.540	0.791

Note: Adjusted for age, age^2, gender, center, first five principal components, course of disease, previous drug usage, assigned medication;

Patients with baseline glucose >= 100.8 mg/dL (5.6 mmol/L) were classified into the prediabetes or diabetes group, the remaining patients were in the non-diabetes or prediabetes group;

Individuals with < 2 years of first-episode SCZ course, and < 14 days of antipsychotic exposure, were classified as first-episode, drug-naïve. The remaining patients were categorized as recurrent. Patients prescribed risperidone, olanzapine, quetiapine, or clozapine were grouped as receiving medication with stronger metabolic side effects; Others (prescribing aripiprazole, ziprasidone, or perphenazine) comprised the milder-metabolic-effect group.

False discovery rate (FDR)-corrected P-value < 0.05 was applied as the significant threshold accounting for multiple tests across drug targets and outcomes. The conventional P<0.05 was applied as the threshold of suggestive significance. Better antipsychotic efficacy: PANSS percentage reduction > median value of PANSS percentage reduction; The standard error (SE) corresponds to the beta coefficient derived from the natural logarithm of the odds ratio (log OR).

Abbreviations: PANSS, Positive and Negative Syndrome Scale; N, Negative scale; G, General psychopathology scale; P, Positive scale; SE, standard error; GRS, genetic risk score; CAPEC, Chinese Antipsychotics Pharmacogenetics Consortium.

Table S47. Descriptive analysis of glucose and TG by GRSs of *APOC3* and *GCK* in the CAPOC study. Related to Figure 1.

Characteristics (mg/dL)	Statistics	Both lower (N=498)	*APOC3*_TG GRS higher (N=555)	*GCK* GRS higher (N=556)	Both higher (N=502)
Glucose (mg/dL)	Mean	86.6	87.3	86.2	86.4
	Median	85.9	86.9	85.0	85.4
	Q1	77.9	78.4	77.1	77.4
	Q3	93.7	95.5	93.2	93.8
TG (mg/dL)	Mean	108.0	98.7	106.6	96.2
	Median	94.3	85.9	93.9	85.5
	Q1	70.9	62.0	67.3	62.9
	Q3	133.5	118.7	134.8	117.8
**Characteristics (mg/dL)**	**Statistics**	**Both lower (N=428)**	***APOC3*_TC GRS higher (N=625)**	***GCK* GRS higher (N=484)**	**Both higher (N=574)**
Glucose (mg/dL)	Mean	86.3	87.4	86.4	86.2
	Median	85.6	87.2	85.2	85.2
	Q1	78.0	78.4	77.1	77.3
	Q3	93.7	95.5	93.7	93.7
TC (mg/dL)	Mean	161.1	157.2	161.2	158.8
	Median	155.5	154.3	159.1	154.9
	Q1	133.8	135.0	136.0	135.3
	Q3	183.4	175.9	181.0	179.4

Abbreviations: GRS, genetic risk score; TG, triglyceride; TC, total cholesterol; CAPOC, Chinese Antipsychotics Pharmacogenomics Consortium

Table S48. Joint associations between *APOC3, GCK* GRSs and TG, glucose in the CAPOC study. Related to Figure 1.

Trait	Group	Beta	SE	CI_lower	CI_upper	P
***APOC3*_TG * *GCK*_Glucose**						
Glucose	*APOC3* GRS higher	0.972	0.804	-0.605	2.549	0.227
	GCK GRS higher	-0.002	0.805	-1.581	1.577	0.998
	Both higher	0.187	0.824	-1.428	1.803	0.820
TG	*APOC3* GRS higher	-9.213	3.186	-15.460	-2.965	0.004
	GCK GRS higher	-0.830	3.190	-7.085	5.425	0.795
	Both higher	-11.225	3.264	-17.626	-4.823	0.001
***APOC3*_TC * *GCK*_Glucose**						
Glucose	*APOC3* GRS higher	1.240	0.818	-0.365	2.845	0.130
	GCK GRS higher	0.343	0.865	-1.355	2.040	0.692
	Both higher	0.287	0.833	-1.346	1.920	0.730
TC	*APOC3* GRS higher	-4.385	2.067	-8.439	-0.331	0.034
	GCK GRS higher	0.065	2.186	-4.223	4.352	0.976
	Both higher	-1.997	2.103	-6.121	2.127	0.342

Adjusted for age, age^2, gender, center, first five principal components, course of disease, previous drug usage;

Abbreviations: SE, standard error; GRS, genetic risk score; TG, triglyceride; TC, total cholesterol; CAPOC, Chinese Antipsychotics Pharmacogenomics Consortium.

Table S49. Interactive associations between *APOC3, GCK* GRSs and measured glucose, TG in the CAPOC study. Related to Figure 1.

Trait	GRS	Beta	SE	CI_lower	CI_upper	P
***APOC3*_TG * *GCK*_Glucose**						
Glucose	Higher *APOC3* GRS	0.230	0.284	-0.327	0.788	0.417
	Higher *GCK* GRS	-0.634	0.284	-1.190	-0.078	0.026
	Both higher	-0.359	0.281	-0.911	0.193	0.202
TG	Higher *APOC3* GRS	-6.332	1.124	-8.536	-4.129	1.98E-08
	Higher *GCK* GRS	-1.038	1.122	-3.238	1.162	0.355
	Both higher	-0.845	1.113	-3.026	1.337	0.448
***APOC3*_TC * *GCK*_Glucose**						
Glucose	Higher *APOC3* GRS	0.120	0.286	-0.441	0.681	0.674
	Higher *GCK* GRS	-0.655	0.283	-1.211	-0.100	0.021
	Both higher	-0.237	0.291	-0.807	0.333	0.415
TC	Higher *APOC3* GRS	-1.929	0.723	-3.346	-0.511	7.70E-03
	Higher *GCK* GRS	0.398	0.716	-1.005	1.802	0.578
	Both higher	-0.149	0.734	-1.589	1.292	0.840

Adjusted for age, age^2, gender, center, first five principal components, course of disease, previous drug usage;

Abbreviations: SE, standard error; GRS, genetic risk score; TG, triglyceride; TC, total cholesterol; CAPOC, Chinese Antipsychotics Pharmacogenomics Consortium.

Table S50. Joint associations between *APOC3, GCK* GRSs and PANSS percentage reduction in the CAPOC study. Related to Figure 1.

PANSS	Group	Beta	SE	CI_lower	CI_upper	P
***APOC3*_TG * *GCK*_Glucose**						
PANSS	*APOC3* GRS higher	0.570	1.746	-2.854	3.994	0.744
	GCK GRS higher	-3.463	1.748	-6.891	-0.035	0.048
	Both higher	-2.882	1.789	-6.389	0.626	0.107
PANSS_P	*APOC3* GRS higher	0.608	1.401	-2.139	3.355	0.664
	GCK GRS higher	-1.954	1.403	-4.704	0.797	0.164
	Both higher	-2.168	1.435	-4.983	0.647	0.131
PANSS_N	*APOC3* GRS higher	0.868	1.341	-1.762	3.498	0.518
	GCK GRS higher	-3.075	1.343	-5.709	-0.442	0.022
	Both higher	-2.045	1.374	-4.740	0.650	0.137
PANSS_G	*APOC3* GRS higher	0.003	1.133	-2.218	2.224	0.998
	GCK GRS higher	-2.202	1.134	-4.426	0.022	0.052
	Both higher	-2.146	1.160	-4.422	0.129	0.065
***APOC3*_TC * *GCK*_Glucose**						
PANSS	*APOC3* GRS higher	2.090	1.776	-1.394	5.574	0.240
	GCK GRS higher	-2.936	1.879	-6.620	0.748	0.118
	Both higher	-1.662	1.807	-5.206	1.882	0.358
PANSS_P	*APOC3* GRS higher	1.361	1.426	-1.435	4.157	0.340
	GCK GRS higher	-1.870	1.508	-4.826	1.087	0.215
	Both higher	-1.314	1.450	-4.158	1.530	0.365
PANSS_N	*APOC3* GRS higher	1.936	1.364	-0.739	4.610	0.156
	GCK GRS higher	-3.285	1.442	-6.114	-0.457	0.023
	Both higher	-0.725	1.387	-3.445	1.996	0.601
PANSS_G	*APOC3* GRS higher	1.160	1.153	-1.101	3.420	0.314
	GCK GRS higher	-1.570	1.219	-3.960	0.821	0.198
	Both higher	-1.415	1.173	-3.714	0.885	0.228

Note: Adjusted for age, age^2, gender, center, first five principal components, course of disease, previous drug usage, assigned medication;

*Bonferroni correction P-value < 0.05/4=0.0125 was applied as the significant threshold accounting for multiple tests across PANSS subscales. The conventional P<0.05 was applied as the threshold of suggestive significance.

Abbreviations: PANSS, Positive and Negative Syndrome Scale; N, Negative scale; G, General psychopathology scale; P, Positive scale; SE, standard error; GRS, genetic risk score; TG, triglyceride; TC, total cholesterol; CAPOC, Chinese Antipsychotics Pharmacogenomics Consortium.

Table S51. Joint associations between *APOC3, GCK* GRSs and better antipsychotic efficacy in the CAPOC study. Related to Figure 1.

PANSS	Group	OR	SE_logOR	CI_lower	CI_upper	P
***APOC3*_TG * *GCK*_Glucose**						
PANSS	*APOC3* GRS higher	1.145	0.128	0.891	1.473	0.289
	GCK GRS higher	0.848	0.128	0.659	1.090	0.199
	Both higher	0.976	0.131	0.754	1.262	0.850
PANSS_P	*APOC3* GRS higher	1.210	0.127	0.943	1.554	0.134
	GCK GRS higher	0.955	0.127	0.744	1.226	0.717
	Both higher	0.798	0.131	0.617	1.031	0.084
PANSS_N	*APOC3* GRS higher	1.179	0.127	0.919	1.514	0.195
	GCK GRS higher	0.773	0.127	0.602	0.992	0.043
	Both higher	0.993	0.130	0.769	1.281	0.954
PANSS_G	*APOC3* GRS higher	1.019	0.128	0.793	1.310	0.882
	GCK GRS higher	0.781	0.128	0.608	1.004	0.054
	Both higher	0.817	0.131	0.631	1.056	0.123
***APOC3*_TC * *GCK*_Glucose**						
PANSS	*APOC3* GRS higher	1.258	0.130	0.974	1.625	0.079
	GCK GRS higher	0.859	0.138	0.655	1.126	0.271
	Both higher	1.067	0.133	0.823	1.384	0.624
PANSS_P	*APOC3* GRS higher	1.312	0.130	1.018	1.693	0.036
	GCK GRS higher	0.941	0.137	0.719	1.231	0.659
	Both higher	0.923	0.132	0.713	1.195	0.543
PANSS_N	*APOC3* GRS higher	1.338	0.130	1.038	1.725	0.025
	GCK GRS higher	0.788	0.137	0.602	1.030	0.081
	Both higher	1.108	0.131	0.856	1.433	0.437
PANSS_G	*APOC3* GRS higher	1.080	0.130	0.837	1.394	0.553
	GCK GRS higher	0.788	0.138	0.601	1.032	0.084
	Both higher	0.861	0.132	0.664	1.117	0.260

Note: Adjusted for age, age^2, gender, center, first five principal components, course of disease, previous drug usage, assigned medication;

*Bonferroni correction P-value < 0.05/4=0.0125 was applied as the significant threshold accounting for multiple tests across PANSS subscales. The conventional P<0.05 was applied as the threshold of suggestive significance. Better antipsychotic efficacy: PANSS percentage reduction > median value of PANSS percentage reduction; The standard error (SE) corresponds to the beta coefficient derived from the natural logarithm of the odds ratio (log OR).

Abbreviations: PANSS, Positive and Negative Syndrome Scale; N, Negative scale; G, General psychopathology scale; P, Positive scale; SE, standard error; GRS, genetic risk score; TG, triglyceride; TC, total cholesterol; CAPOC, Chinese Antipsychotics Pharmacogenomics Consortium.

Table S52. Interactive associations between *APOC3, GCK* GRSs and PANSS percentage reduction in the CAPOC study. Related to Figure 1.

PANSS	GRS	beta	SE	CI_lower	CI_upper	P
***APOC3*_TG * *GCK*_Glucose**						
PANSS	Higher *APOC3* GRS	0.631	0.618	-0.580	1.842	0.307
	Higher *GCK* GRS	-1.676	0.617	-2.885	-0.467	0.007*
	Both higher	0.233	0.612	-0.967	1.432	0.704
PANSS_P	Higher *APOC3* GRS	0.170	0.496	-0.802	1.143	0.731
	Higher *GCK* GRS	-1.047	0.495	-2.018	-0.076	0.035
	Both higher	0.052	0.491	-0.911	1.015	0.916
PANSS_N	Higher *APOC3* GRS	1.121	0.474	0.192	2.051	0.018
	Higher *GCK* GRS	-1.566	0.473	-2.494	-0.638	0.001*
	Both higher	0.298	0.469	-0.622	1.218	0.525
PANSS_G	Higher *APOC3* GRS	0.241	0.401	-0.545	1.026	0.548
	Higher *GCK* GRS	-1.105	0.400	-1.889	-0.320	0.006*
	Both higher	0.019	0.397	-0.759	0.797	0.962
***APOC3*_TC * *GCK*_Glucose**						
PANSS	Higher *APOC3* GRS	0.356	0.622	-0.863	1.575	0.567
	Higher *GCK* GRS	-1.709	0.615	-2.916	-0.502	0.006*
	Both higher	0.605	0.631	-0.633	1.843	0.338
PANSS_P	Higher *APOC3* GRS	0.058	0.499	-0.921	1.036	0.908
	Higher *GCK* GRS	-1.065	0.494	-2.034	-0.096	0.031
	Both higher	0.316	0.507	-0.678	1.310	0.533
PANSS_N	Higher *APOC3* GRS	0.864	0.477	-0.071	1.799	0.070
	Higher *GCK* GRS	-1.603	0.472	-2.529	-0.677	0.001*
	Both higher	0.647	0.484	-0.303	1.597	0.182
PANSS_G	Higher *APOC3* GRS	-0.021	0.403	-0.812	0.769	0.958
	Higher *GCK* GRS	-1.131	0.399	-1.914	-0.348	0.005*
	Both higher	0.253	0.410	-0.551	1.056	0.537

Note: Adjusted for age, age^2, gender, center, first five principal components, course of disease, previous drug usage, assigned medication;

*Bonferroni correction P-value < 0.05/4=0.0125 was applied as the significant threshold accounting for multiple tests across PANSS subscales. The conventional P<0.05 was applied as the threshold of suggestive significance.

Abbreviations: PANSS, Positive and Negative Syndrome Scale; N, Negative scale; G, General psychopathology scale; P, Positive scale; SE, standard error; GRS, genetic risk score; TG, triglyceride; TC, total cholesterol; CAPOC, Chinese Antipsychotics Pharmacogenomics Consortium.

Table S53. Interactive associations between *APOC3, GCK* GRSs and better antipsychotic efficacy in the CAPOC study. Related to Figure 1.

PANSS	GRS	OR	SE_logOR	CI_lower	CI_upper	P
***APOC3*_TG * *GCK*_Glucose**						
PANSS	Higher *APOC3* GRS	1.079	0.045	0.987	1.179	0.095
	Higher *GCK* GRS	0.922	0.045	0.843	1.008	0.074
	Both higher	0.993	0.045	0.909	1.085	0.884
PANSS_P	Higher *APOC3* GRS	0.992	0.045	0.908	1.083	0.851
	Higher *GCK* GRS	0.898	0.045	0.822	0.981	0.018
	Both higher	0.940	0.045	0.860	1.026	0.166
PANSS_N	Higher *APOC3* GRS	1.149	0.045	1.052	1.255	0.002*
	Higher *GCK* GRS	0.909	0.045	0.831	0.993	0.034
	Both higher	1.015	0.045	0.930	1.109	0.736
PANSS_G	Higher *APOC3* GRS	1.038	0.045	0.949	1.134	0.415
	Higher *GCK* GRS	0.913	0.045	0.835	0.998	0.046
	Both higher	1.012	0.045	0.926	1.105	0.797
***APOC3*_TC * *GCK*_Glucose**						
PANSS	Higher *APOC3* GRS	1.048	0.046	0.959	1.147	0.301
	Higher *GCK* GRS	0.919	0.045	0.840	1.004	0.061
	Both higher	1.023	0.046	0.934	1.121	0.627
PANSS_P	Higher *APOC3* GRS	1.017	0.045	0.930	1.111	0.713
	Higher *GCK* GRS	0.897	0.045	0.821	0.980	0.016
	Both higher	0.970	0.046	0.886	1.062	0.515
PANSS_N	Higher *APOC3* GRS	1.128	0.045	1.032	1.233	0.008*
	Higher *GCK* GRS	0.905	0.045	0.828	0.989	0.027
	Both higher	1.047	0.046	0.956	1.146	0.323
PANSS_G	Higher *APOC3* GRS	1.007	0.046	0.921	1.101	0.877
	Higher *GCK* GRS	0.910	0.045	0.833	0.995	0.039
	Both higher	1.041	0.046	0.951	1.141	0.383

Note: Adjusted for age, age^2, gender, center, first five principal components, course of disease, previous drug usage, assigned medication;

*Bonferroni correction P-value < 0.05/4=0.0125 was applied as the significant threshold accounting for multiple tests across PANSS subscales. The conventional P<0.05 was applied as the threshold of suggestive significance. Better antipsychotic efficacy: PANSS percentage reduction > median value of PANSS percentage reduction; The standard error (SE) corresponds to the beta coefficient derived from the natural logarithm of the odds ratio (log OR).

Abbreviations: PANSS, Positive and Negative Syndrome Scale; N, Negative scale; G, General psychopathology scale; P, Positive scale; SE, standard error; GRS, genetic risk score; TG, triglyceride; TC, total cholesterol; CAPOC, Chinese Antipsychotics Pharmacogenomics Consortium.

Table S54. Multi-omics datasets. Related to STAR Methods.

Study	Abbreviation	Type of data	Sample size	Ancestry	PMID
Chinese Antipsychotics Pharmacogenomics Consortium	CAPOC	Individual-level phenotype and genotype data	2111	East Asians	29503163
Chinese Antipsychotics Pharmacogenetics Consortium	CAPEC	Individual-level phenotype and genotype data	292	East Asians	29503163
Global Lipids Genetics Consortium	GLGC	GWAS summary statistics of lipids	146492	East Asians	36575460
Taiwan Biobank	Taiwan Biobank	GWAS summary statistics of glucose	92615	East Asians	38116116
Anti-Aging Study Cohort, Cardiometabolic Genome Epidemiology Kita-Nagoya Genomic Quad, Cardiometabolic Genome Epidemiology Kita-Nagoya Genomic Omni, Cebu Longitudinal Health and Nutrition Survey, China Health and Nutrition Survey, Korean Association Resource, Multi-Ethnic Study of Atherosclerosis, Nagahama Study, Nutrition and Health of Aging Population in China, "Singapore Prospective Study Program, Illumina1Mduov3", "Singapore Prospective Study Program, Illumina550", "Singapore Prospective Study Program, Illumina610Quad", Taiwan Metabochip Consortium, The Colorectal Cancer Study, Shanghai Men's Health Study	AASC, CAGE-KING-Quad, CAGE-KING-Omni, CLHNS, CHNS, KARE, MESA, Nagahama Study, NHAPC, SP2-Illumina1Mduov3, SP2-Illumina550, SP2-Illumina610Quad, TAICHI, CRC, Shanghai Men's Health Study	GWAS summary statistics of fasting insulin	29792	East Asians	34059833
Biobank of Japan	BBJ	GWAS summary statistics of systolic blood pressure	145505	East Asians	34594039
Biobank of Japan	BBJ	GWAS summary statistics of schizophrenia	177893	East Asians	34594039
Type 2 Diabetes Global Genetics Initative	T2DGGI	GWAS summary statistics of type 2 diabetes	427504	East Asians	38374256
GWAS summary statistics of oral glucose tolerance test-2h from the Shenzhen Baoan Women’s and Children’s Hospital (Shenzhen, China)	GWAS summary statistics of oral glucose tolerance test-2h from the Shenzhen Baoan Women’s and Children’s Hospital (Shenzhen, China)	GWAS summary statistics of oral glucose tolerance test-2h	24931	East Asians	38372780
Taiwan Biobank	Taiwan Biobank	GWAS summary statistics of Hemoglobin A1c levels	92615	East Asians	38116116
Biobank of Japan	BBJ	GWAS summary statistics of coronary artery disease	178726	East Asians	doi.org/10.1101/2021.09.03.21262975
Biobank of Japan	BBJ	GWAS summary statistics of body mass index	158284	East Asians	28892062

GWAS summary statistics of metabolic syndrome among Koreans	GWAS summary statistics of metabolic syndrome among Koreans	GWAS summary statistics of metabolic syndrome	62314	East Asians	39349817
Guangzhou Nutrition and Health study	GNHS	pQTL summary statistics in the blood	2410	East Asians	36797296
Diversity and scale: Genetic architecture of 2068 traits in the VA Million Veteran Program	MVP	GWAS summary statistics of hyperlipidemia	426603	Europeans	39024449
UK Biobank	UKB	GWAS summary statistics of sphingomyelin levels	115006	Europeans	35213538
The Qatar Genome Program Research Consortium	The Qatar Genome Program Research Consortium	GWAS summary statistics of beta-endorphin levels	2935	Greater Middle Eastern	36168886
Six cohorts (Alzheimer's Disease Neuroimaging Initiative, Dominantly Inherited Alzheimer's Network, Knight-ADRC Memory and Aging Project, Ace Alzheimer Center Barcelona, Barcelona-1, and Parkinson's Progression Markers Initiative)	ADNI, DIAN, MAP, FACE, Barcelona-1, PPMI	pQTL summary statistics in the cerebrospinal fluid	3107	Europeans	37333337
The Adult Genotype Tissue Expression Project	GTEx v10	eQTL summary statistics in the brain_cortex	268	Europeans	32913098
The Adult Genotype Tissue Expression Project	GTEx v10	eQTL summary statistics in the brain_hypothalamus	256	Europeans	32913098
The Adult Genotype Tissue Expression Project	GTEx v10	eQTL summary statistics in the brain_cerebellum	264	Europeans	32913098
The Adult Genotype Tissue Expression Project	GTEx v10	eQTL summary statistics in the brain_nucleus_accumbens	284	Europeans	32913098
The Adult Genotype Tissue Expression Project	GTEx v10	eQTL summary statistics in the brain_caudate	298	Europeans	32913098
The Adult Genotype Tissue Expression Project	GTEx v10	eQTL summary statistics in the brain_hippocampus	254	Europeans	32913098
The Adult Genotype Tissue Expression Project	GTEx v10	eQTL summary statistics in the skeletal muscle	816	Europeans	32913098
The Adult Genotype Tissue Expression Project	GTEx v10	eQTL summary statistics in the pituitary	311	Europeans	32913098
The Adult Genotype Tissue Expression Project	GTEx v10	eQTL summary statistics in the visceral adipose	584	Europeans	32913098
The Adult Genotype Tissue Expression Project	GTEx v10	eQTL summary statistics in the liver	261	Europeans	32913098
The Adult Genotype Tissue Expression Project	GTEx v10	eQTL summary statistics in the artery_coronary	268	Europeans	32913098
The Adult Genotype Tissue Expression Project	GTEx v10	eQTL summary statistics in the small intestine - terminal ileum	206	Europeans	32913098
The Adult Genotype Tissue Expression Project	GTEx v10	eQTL summary statistics in the pancreas	362	Europeans	32913098

Table S55. Target genes for antidiabetic and lipid-lowering drugs identified using the DrugBank. Related to Figure 1.

Drug class	ATC code	Name	Gene		
**DRUGS USED IN DIABETES**					
Biguanides	A10BA03	buformin	*ABCB11*		
Sulfonylureas	A10BB01	glibenclamide	*ABCB11*		
Glucagon-like peptide-1 (GLP-1) analogues	A10BJ01	exenatide	*GLP1R*		
	A10BJ02	liraglutide	*GLP1R*		
	A10BJ03	lixisenatide	*GLP1R*		
	A10BJ04	albiglutide	*GLP1R*		
	A10BJ05	dulaglutide	*GLP1R*		
	A10BJ06	semaglutide	*GLP1R*		
Other blood glucose-lowering drugs, excl. insulins	A10BX05	pramlintide	*GLP1R*		
	A10BX16	tirzepatide	*GLP1R*		
	A10BX18	dorzagliatin	*GCK*		
**LIPID MODIFYING AGENTS**					
HMG CoA reductase inhibitors	C10AA01	simvastatin	*HMGCR*		
	C10AA02	lovastatin	*LDLR*	*LPA*	*HMGCR*
	C10AA03	pravastatin	*HMGCR*		
	C10AA04	fluvastatin	*HMGCR*		
	C10AA05	atorvastatin	*HMGCR*		
	C10AA06	cerivastatin	*HMGCR*		
	C10AA07	rosuvastatin	*HMGCR*		
	C10AA08	pitavastatin	*HMGCR*		
Fibrates	C10AB01	clofibrate	*LPL*		
	C10AB02	bezafibrate	*PPARG*		
	C10AB04	gemfibrozil	*LPL*		
Nicotinic acid	C10AD02	nicotinic acid	*HCAR3*	*HCAR2*	
Other lipid modifying agents	C10AX02	probucol	*ABCA1*		
	C10AX12	lomitapide	*MTTP*		
	C10AX13	evolocumab	*PCSK9*		
	C10AX14	alirocumab	*PCSK9*		
	C10AX16	inclisiran	*PCSK9*		
	C10AX17	evinacumab	*ANGPTL3*		
	C10AX18	volanesorsen	*APOC3*		
	C10AX11	mipomersen	*APOB*		
	-	torcetrapib	*CETP*		

Notes: Targets for which the drug acts as an agonist are marked in blue; Targets for which the drug acts as an inhibitor are marked in black.
