# Supplementary material for: Lipid-lowering and glucose-lowering drug targets differentially modulate antipsychotic treatment efficacy in schizophrenia
Source: Cell Rep Med. 2026 Mar 17;7(3):102653. doi: 10.1016/j.xcrm.2026.102653 (PMC13006399; doi:10.1016/j.xcrm.2026.102653)
Supplement: Document S2. Article plus supplemental information [file mmc8.pdf]

Cell Reports Medicine

Article

Lipid-lowering and glucose-lowering drug targets differentially modulate antipsychotic treatment efficacy in schizophrenia

Graphical abstract

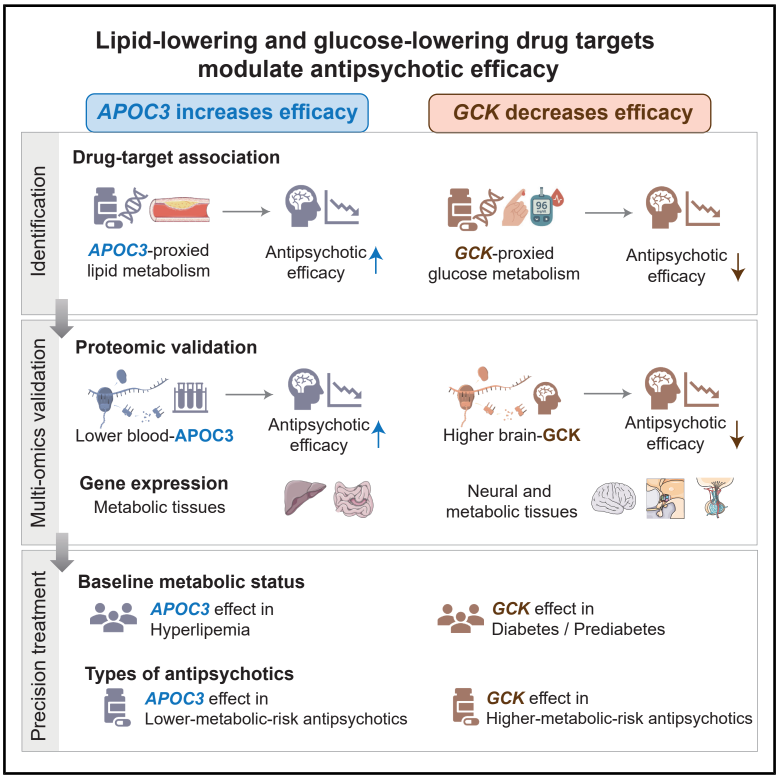

Highlights

- *APOC3*-proxied lipid lowering elevates antipsychotic efficacy of negative symptoms
- *GCK*-proxied glucose lowering reduces positive, negative, general-symptom recovery
- *APOC3/GCK* negative-symptom effects persist in hyperlipidemic/diabetic subgroups
- Genetic proteomics validate blood-APOC3, brain-GCK effects on negative symptoms

Authors

Yunqing Zhu, Rui Yuan, Zhe Lu, ..., Yang Yang, Yaoyao Sun, Weihua Yue

Correspondence

sunyaoyao@bjmu.edu.cn (Y.S.), dryue@bjmu.edu.cn (W.Y.)

In brief

Whether metabolic-modifying agents aggravate schizophrenia progression remains unclear. Zhu et al. identify that *APOC3*-proxied lipid lowering improves schizophrenia negative-symptom recovery, and *GCK*-related glucose lowering worsens antipsychotic efficacy. Causal effects of *APOC3/GCK* on negative symptoms are robust in hyperlipidemic/diabetic subgroups. This study suggests volanesorsen/olezarsen as potential adjunctive candidates, and dorzagliatin warrants prudence in schizophrenia-metabolic comorbidity.

Zhu et al., 2026, Cell Reports Medicine 7, 102653
March 17, 2026 © 2026 The Authors. Published by Elsevier Inc.
https://doi.org/10.1016/j.xcrm.2026.102653

CellPress

Cell Reports Medicine

CellPress
OPEN ACCESS

Article

Lipid-lowering and glucose-lowering drug targets differentially modulate antipsychotic treatment efficacy in schizophrenia

Yunqing Zhu,[1] Rui Yuan,[1] Zhe Lu,[1] Yuyanan Zhang,[1] Zhewei Kang,[1] Xiaoyang Feng,[1] Guorui Zhao,[1] Junyuan Sun,[1] Jing Guo,[1] Tong Yu,[1] Yang Yang,[1] Yaoyao Sun,[1,*] and Weihua Yue[1,2,3,*]

[1]Peking University Sixth Hospital, Peking University Institute of Mental Health, NHC Key Laboratory of Mental Health (Peking University), National Clinical Research Center for Mental Disorders (Peking University Sixth Hospital), Beijing 100191, China

[2]PKU-IDG/McGovern Institute for Brain Research, Peking University, Beijing 100871, China

[3]Lead contact

*Correspondence: sunyaoyao@bjmu.edu.cn (Y.S.), dryue@bjmu.edu.cn (W.Y.)

https://doi.org/10.1016/j.xcrm.2026.102653

SUMMARY

Schizophrenia is frequently comorbid with dyslipidemia and hyperglycemia. However, whether metabolic-modifying agents aggravate schizophrenia progression remains unclear. We perform a drug-target genetic association study in two independent Han Chinese schizophrenia cohorts (N = 2,111/292 for discovery/validation). Leveraging metabolic genome-wide association studies, we generate genetic risk scores (GRSs) for lipid-modifying and hypoglycemic targets. Those with higher *APOC3* (inhibited by volanesorsen/olezarsen) GRS exhibit attenuated triglycerides and improvement in negative symptoms assessed by Positive and Negative Syndrome Scale (PANSS) (β = 1.23, 95% confidence interval [CI]: 0.30–2.16). Higher *GCK* (activated by dorzagliatin) GRS is associated with decreased glucose and less improvement across PANSS total (β = −1.70, 95% CI: −2.91–0.50), positive, negative, general subscales. Causal associations of *GCK* are replicated in independent validation. The effects of *APOC3* and *GCK* on negative symptom recovery are robust in hyperlipidemic/diabetic subgroups. Genetically proxied proteomics analysis provides further functional validation for the identified target-outcome associations. Our findings suggest volanesorsen/olezarsen as potential adjunctive candidates; dorzagliatin warrants prudence in schizophrenia with metabolic disturbance.

INTRODUCTION

Schizophrenia (SCZ) carries a heavy psychiatric burden worldwide, leading to considerable disability and substantial financial costs. Compared to the general population, individuals with SCZ have a markedly reduced life expectancy by approximately 16–20 years, mainly attributed to comorbid cardiometabolic diseases.[1] A global meta-analysis of 198 studies reported a pooled prevalence of metabolic disturbance of 33.4% in SCZ, representing a 1.87-fold higher risk than in the general population.[2] Hypertriglyceridemia and diabetes were associated with 2.73- and 1.99-fold elevated risks, respectively.[3] Several key contributors to this increasing comorbidity included the antipsychotic-induced metabolic side effects, shared genetic susceptibility between SCZ and metabolic indicators,[4] unhealthy diets, and few physical activities.[5] Previous clinical studies reported that lipid-modifying drugs may induce neuropsychiatric adverse effects, including paranoia, irritability, depression, and cognitive impairments.[6,7] Adjuvant metformin might also worsen psychotic symptoms in SCZ.[8] Given concerns that lipid- and glucose-modifying agents may exacerbate psychiatric symptoms, clinicians are less likely to prescribe metabolic medications in those with SCZ (37%) than in non-SCZ populations (85%).[9,10] Thus, it is critical to explore the potential impact of commonly prescribed metabolic-regulating drugs on the progress of psychotic symptoms, which could promote coordinated treatments for individuals co-occurring with SCZ and metabolic disorders.

To date, an increasing number of randomized clinical trials (RCTs) have examined whether metabolic medicines can modify antipsychotic efficacy in SCZ. Most trials of lipid-modifying drugs have centered on statins. The latest meta-analysis involving nine RCTs (N = 533) found that adjunctive statin therapy was associated with improvements in negative symptoms in SCZ.[11] However, the potential influence of other classes of agents on the antipsychotic efficacy remains largely unknown. Moreover, previous clinical trials demonstrated inconsistent evidence for different types of glucose-lowering agents on the efficacy of antipsychotics. A recent multicenter RCT reported that adding semaglutide, a glucagon-like peptide-1 receptor agonist, to quetiapine therapy did not affect psychotic symptom recovery (N = 31).[12] Similarly, a meta-analysis (10 RCTs, N = 453) indicated that current evidence did not support an impact of topiramate on the clinical improvement of SCZ.[13] In contrast, another RCT conducted among 40 patients with SCZ suggested a potential benefit of pioglitazone adjuvant therapy on the reduction rate

© 2026 The Authors. Published by Elsevier Inc.
This is an open access article under the CC BY license (http://creativecommons.org/licenses/by/4.0/).

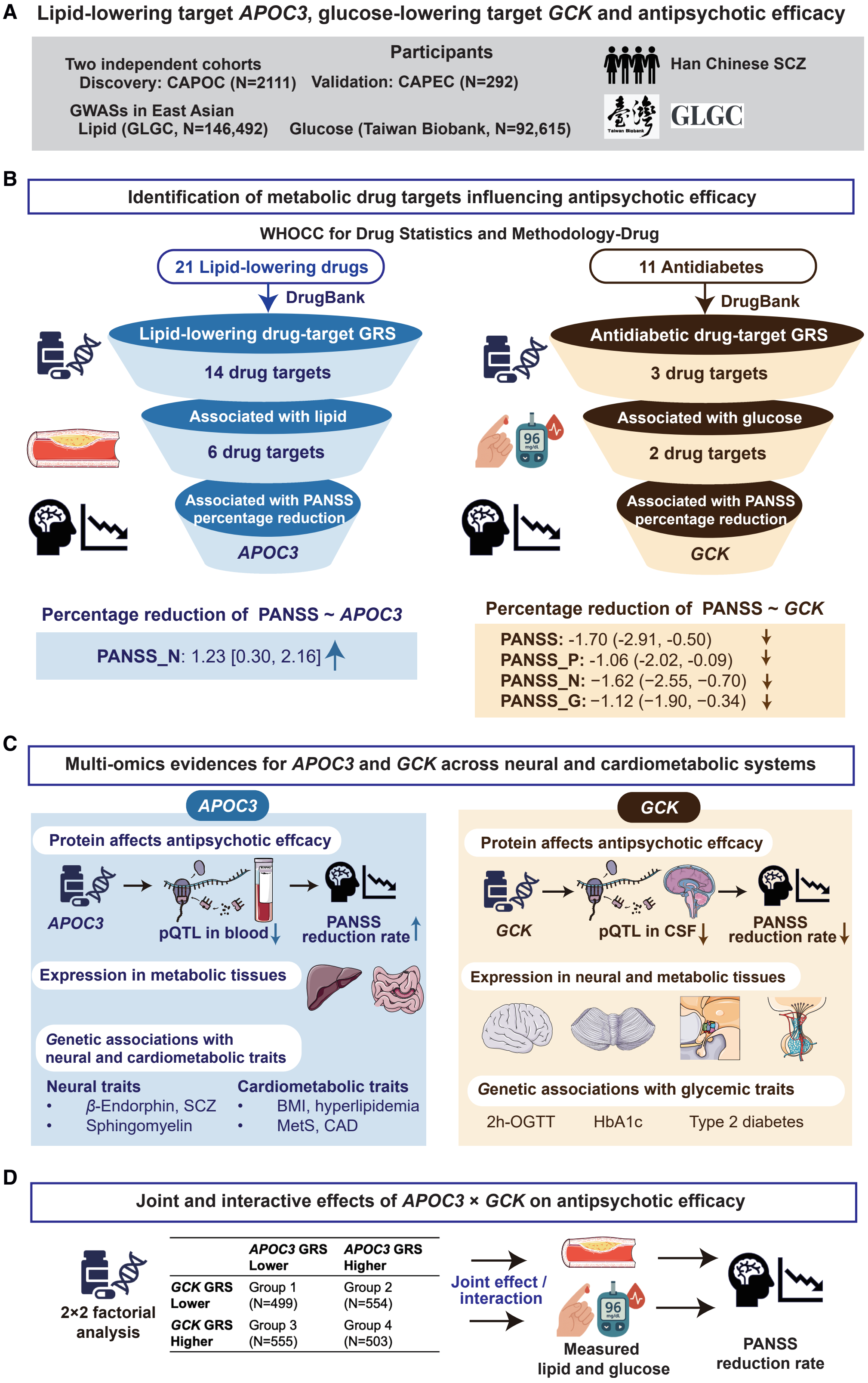

(legend on next page)

of Positive and Negative Syndrome Scale (PANSS) total score and negative scale.[14]

The heterogeneity of these clinical findings suggests that specific glucose-lowering drug targets might differently modulate the antipsychotic efficacy. Similar target-specific dynamics extend to distinct lipid-lowering targets. Comprehensive profiling of the known lipid-modifying and antidiabetic targets will help detect the targets affecting the SCZ symptoms, thereby informing the co-prescribing in psychiatric practice.

Our recent work pinpointed several pleiotropic loci shared between SCZ and cardiometabolic traits among East Asians, motivating the identification of common therapeutic targets through a genetics-based approach. The drug-target Mendelian randomization (MR) analysis applies genetic instruments in each target gene region. This strategy can mimic a randomly assigned drug-intervention study for causal inference, thereby reducing the reverse and confounding bias in observational studies.[15,16] By concentrating on well-defined pharmacologically active targets, this approach offers deeper mechanistic insight into drug action and is more practical and cost-effective than traditional RCTs.[17] None of the previous studies have evaluated the drug-target effects on SCZ symptoms from a genomic aspect. Given the complex metabolic disturbances in patients with SCZ,[18] examining the joint impact of glucose-modulating and lipid-modulating drug targets on antipsychotic efficacy is necessary.

Based on two independent cohorts of participants with SCZ, we conducted a drug-target genetic association study, aimed to (1) estimate the causal effects of lipid lowering and antidiabetic targets on lipid and glucose levels, respectively, as well as their influences on the efficacy of antipsychotic treatments; (2) interpret the identified drug targets affecting antipsychotic efficacy from a multi-omics perspective across brain and cardiometabolic tissues; and (3) evaluate the potential joint and interactive effects of the identified lipid-lowering and antidiabetic targets on lipid and glucose, as well as an antipsychotic efficacy through a 2 $\times$ 2 factorial drug-target design (Figure 1).

RESULTS

Participant characteristics

Our study followed the Strengthening the Reporting of Observational Studies in Epidemiology MR reporting guidelines (Table S1). The present discovery cohort (N = 2,111) was based on the Chinese Antipsychotics Pharmacogenomics Consortium (CAPOC) study, with antipsychotic efficacy assessed at week six. In the CAPOC study, the six-week total PANSS percentage reduction had a median (interquartile range [IQR], %) of 52.4 (29.2, 72.1) (Figure S1). Total cholesterol (TC), triglycerides (TG), and glucose levels (median [IQR], mg/dL) were 155.5 (135.3, 179.8), 88.6 (64.7, 126.7), and 85.8 (77.5, 93.9), respectively. All of the 14 genetic risk scores (GRSs) for lipid-lowering targets and three antidiabetic drug-target GRSs had F statistics larger than 10 (Tables S2, S3, S4, S5, S6, and S7). Participants with higher GRS of lipid-lowering target apolipoprotein C3 (*APOC3*, inhibited by volanesorsen/olezarsen) exhibited reduced concentrations of TC (p for trend = 0.005) and TG (p for trend <0.001). The *APOC3*-GRS accounted for 0.3% and 5.5% of the variances in TC and TG, respectively (Tables 1; Tables S2 and S8). Similarly, the group with higher GRS of glucose-lowering target glucokinase (*GCK*, activated by dorzagliatin) showed decreased glucose (p for trend = 0.017), with the *GCK*-GRS explaining 1.1% of the glucose variance (Tables 1, S2, and S9). No significant associations were observed between drug-target GRSs and other baseline characteristics (p for trend >0.05) (Tables 1 and S10).

Our independent validation was derived from the Chinese Antipsychotics Pharmacogenetics Consortium (CAPEC) study (N = 292). By week eight, the CAPEC participants achieved a median 62.8% decrease in the total PANSS score, with an IQR of 47.0%–75.7% (Figure S1). The detailed characteristics of individuals were displayed in Table S11. The regression diagnostics did not reveal major violations of model assumptions or evidence of model instability for the main analyses (Tables S12 and S13).

Associations between drug-target GRSs with lipids and glucose

Among the 14 lipid-modifying drug-target GRSs, one, four, one, and three GRSs were negatively associated with the measured low-density lipoprotein cholesterol (LDLC), high-density lipoprotein cholesterol (HDLC), TG, and TC, respectively (Benjamini-Hochberg false-discovery-rate-corrected p [P_{FDR}] < 0.05). Two antidiabetic drug-target GRSs were associated with lower glucose levels (P_{FDR} < 0.05). Full results could be found in Tables S8 and S9. Individuals carrying higher GRS of *APOC3*, mimicking exposure to the lipid-modifying drug volanesorsen/olezarsen, exhibited attenuated levels of TG and TC, in turn. The corresponding differences were (β, 95% confidence intervals [95% CI], per standard deviations [SD] increase in GRS) −6.34 (−8.53, −4.15) mg/dL (P_{FDR} <0.001) and −2.02 (−3.42, −0.61) mg/dL (P_{FDR} = 0.025). Those with higher GRS of *GCK*

Figure 1. Study flowchart

(A) Information of study participants.

(B) Identification of lipid-lowering target *APOC3* and antidiabetic target *GCK* influencing antipsychotic efficacy.

The effect size and 95% CI, expressed as beta (95% CI), represent the average percentage reduction in the PANSS for per standard deviation increase in the drug-target GRS.

(C) Proteomic, gene-expression, and genetic-association evidences for *APOC3* and *GCK* across neural and cardiometabolic systems.

(D) A framework of 2 $\times$ 2 factorial analysis estimating joint and interactive effects of *APOC3* and *GCK* on antipsychotic efficacy.

Abbreviations: CAPOC, Chinese Antipsychotics Pharmacogenomics Consortium; CAPEC, Chinese Antipsychotics Pharmacogenetics Consortium; GLGC, Global Lipids Genetics Consortium; GWAS, genome-wide association study; *APOC3*, apolipoprotein C3; *GCK*, glucokinase; GRS, genetic risk score; PANSS, Positive and Negative Syndrome Scale; P, positive scale; N, negative scale; G, general psychopathology scale; pQTL, protein quantitative trait loci; CSF, cerebrospinal fluid; BMI, body mass index; 2h-OGTT, 2-hour oral glucose tolerance test; HbA1c, hemoglobin A1c; T2D, type 2 diabetes; SCZ, schizophrenia.

Table 1. Baseline characteristics of participants in the CAPOC study

		APOC3 GRS		*GCK* GRS	
Baseline characteristics	All	Lower	Higher	Lower	Higher
N (%)	2111	1054 (49.9)	1057 (50.1)	1053 (49.9)	1058 (51.1)
Clinical Characteristics					
Male (%)	1048 (49.6)	512 (48.6)	536 (50.7)	529 (50.2)	519 (49.1)
Female (%)	1063 (50.4)	542 (51.4)	521 (49.3)	524 (49.8)	539 (50.9)
Age (years)	30.0 (24.0, 38.0)	30.0 (24.0, 38.0)	30.0 (23.0, 38.0)	30.0 (24.0, 38.0)	30.0 (23.0, 38.0)
Center in the south (%)	858 (40.6)	417 (39.6)	441 (41.7)	405 (38.5)	453 (42.8)
Middle school or lower (%)	1194 (56.6)	591 (56.1)	603 (57.0)	581 (55.2)	613 (57.9)
Married (%)	692 (32.8)	349 (33.1)	343 (32.5)	333 (31.6)	359 (33.9)
First episode (%)	604 (28.6)	305 (28.9)	299 (28.3)	300 (28.5)	304 (28.7)
Course (years)	4.5 (2.0, 10.0)	4.7 (2.0, 10.0)	4.5 (1.8, 10.0)	5.0 (2.0, 10.0)	4.1 (2.0, 9.4)
Previous medication (%)	1107 (52.4)	538 (51.0)	569 (53.8)	566 (53.8)	541 (51.1)
Assigned medication with stronger metabolic side effects (%)	1056 (50.0)	511 (48.5)	545 (51.6)	517 (49.1)	539 (50.9)
Physical Measurements					
BMI (kg/m^2)	21.7 (19.6, 24.2)	21.8 (19.8, 24.2)	21.6 (19.5, 24.2)	21.6 (19.6, 24.2)	21.8 (19.7, 24.2)
SBP (mmHg)	118 (110, 120)	118 (110, 120)	117 (110, 120)	118 (110, 120)	118 (110, 120)
DBP (mmHg)	76 (70, 80)	76 (70, 80)	76 (70, 80)	76 (70, 80)	75 (70, 80)
Laboratory Measurements					
TC (mg/dL)[a]	155.5 (135.3, 179.8)	158.0 (135.3, 181.0)	154.3 (135.0, 178.3)	154.7 (134.2, 178.7)	156.6 (135.7, 181.0)
TG (mg/dL)[a]	88.6 (64.7, 126.7)	93.9 (68.2, 133.7)	85.9 (62.0, 118.7)	88.6 (64.7, 126.7)	89.5 (64.7, 124.9)
HDLC (mg/dL)	49.9 (42.2, 59.2)	49.5 (42.2, 59.2)	50.3 (42.2, 59.2)	49.5 (42.2, 58.8)	50.3 (42.2, 60.3)
LDLC (mg/dL)	84.7 (67.3, 106.3)	85.8 (68.2, 107.5)	83.5 (66.5, 105.2)	85.1 (66.9, 106.0)	84.7 (68.1, 106.3)
Glucose (mg/dL)[a]	85.8 (77.5, 93.9)	85.2 (77.5, 93.7)	86.5 (77.5, 94.6)	86.5 (78.2, 94.8)	85.2 (77.3, 93.7)

Notes.
[a]Participants with increased *APOC3* GRS, predicting reduced concentrations of TC (p for trend = 0.005) and TG (p for trend <0.001). Similarly, the group with higher GRS of *GCK*, reflecting decreased glucose (p for trend = 0.017). The median and interquartile range (IQR) values were displayed for continuous variables. Abbreviations: BMI, body mass index; SBP, systolic blood pressure; DBP, diastolic blood pressure; LDLC, low-density lipoprotein cholesterol; HDLC, high-density lipoprotein cholesterol; TG, triglyceride; TC, total cholesterol. GRS, genetic risk score; CAPOC, Chinese Antipsychotics Pharmacogenomics Consortium study.

(target of glucose-lowering drug dorzagliatin) demonstrated a reduction in the level of glucose (β [95% CI]: −0.68 [−1.23, −0.12], P_{FDR} = 0.034). Details were shown in Figure 2. Associations between drug-target GRSs and metabolic traits in the CAPEC study were in Table S14.

Associations between drug-target GRSs and the PANSS percentage reduction

The single drug-target genetic association analysis demonstrated that the *APOC3*-proxied lowering TG and TC elevated the antipsychotic efficacy of the negative symptom. In the CAPOC study, genetically predicted higher levels of TG were significantly associated with 0.16% (0.05%, 0.27%) lower reduction rate of the negative scale of PANSS, as well as a lower probability of better antipsychotic efficacy (odds ratio [OR] [95% CI]: 0.98 [0.97, 0.99], P_{FDR} < 0.05) (Tables S15, S16, S17, and S18). An SD increase in the *APOC3* GRS—reflecting lower TG and TC—was associated with 1.23% (0.30%, 2.16%) and 0.96% (0.02%, 1.89%) greater PANSS negative subscale percentage reduction, respectively (nominal p < 0.05) (to convert to per 10 mg/dL increase in TG and TC, multiply by 1.58 and 4.95 correspondingly). *APOC3*-predicted TG lowering was significantly associated with better antipsychotic efficacy (OR [95% CI]: 1.16 [1.06, 1.26], P_{FDR} = 0.036). We did not observe associations between the *APOC3*-proxied lowering of TG and TC and the percentage reduction of total PANSS, as well as the positive and general psychopathology subscales. (Figures 3A, S19, and S20). Colocalization analysis at the *APOC3* locus supported a shared causal single-nucleotide polymorphism (SNP) between TG/TC and PANSS-negative percentage reduction. The top candidate shared variant was rs651821, with an H4 posterior probability (SNP.PP.H4) exceeding 0.6 (Table S21; Figure S2).

In contrast, the glucose reduction predicted by *GCK* diminished the therapeutic efficacy. Genetically predicted increased glucose levels were associated with greater PANSS reduction rates across total (β [95% CI]: 0.78 [0.15,

CellPress
OPEN ACCESS

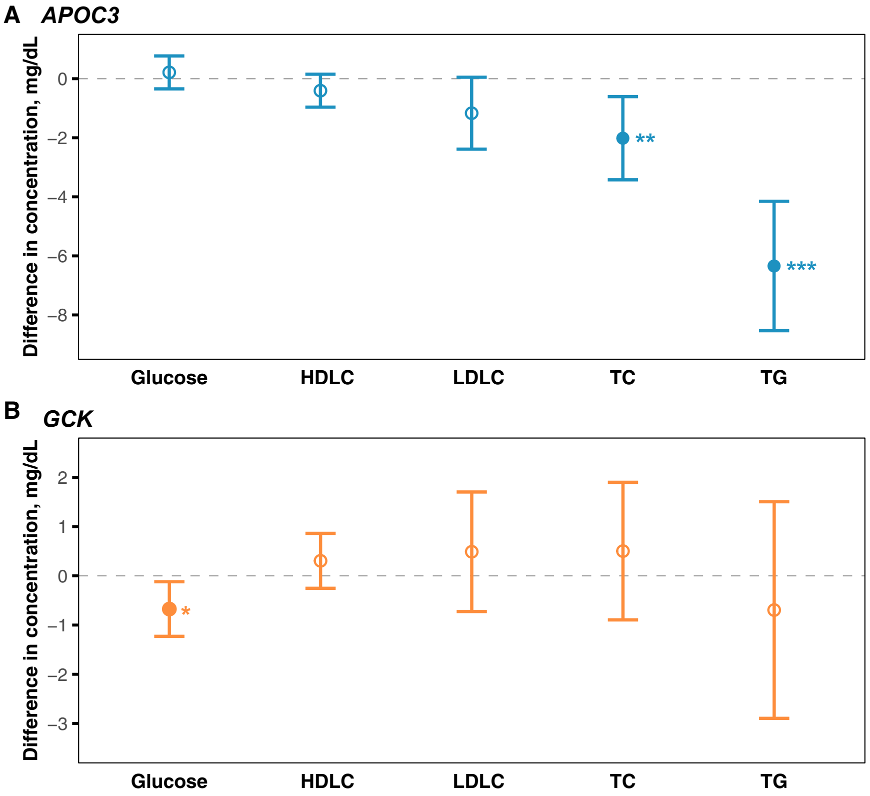

Figure 2. Associations of *APOC3*, *GCK* GRSs with the lipids and glucose

(A) Associations of *APOC3* GRS with the lipids and glucose.

(B) Associations of *GCK* GRS with the lipids and glucose. The beta (95% CI) represents the difference in the plasma lipids and glucose for per standard deviation increase in the drug-target GRS. Error bars indicate the 95% CIs. Nominal p values are provided. *$p < 0.05$, **$p < 0.01$, ***$p < 0.001$.

The corresponding result can be found in Tables S8 and S9.

Abbreviations: *APOC3*, apolipoprotein C3; *GCK*, glucokinase; HDLC, high-density lipoprotein cholesterol; LDLC, low-density lipoprotein cholesterol; TC, total cholesterol; TG, triglyceride; GRS, genetic risk score.

1.42]), positive (β [95% CI]: 0.58 [0.08, 1.09]), negative (β [95% CI]: 0.64 [0.16, 1.13]), and general psychopathology (β [95% CI]: 0.47 [0.06, 0.88]) subscales ($P_{FDR} < 0.05$) (Tables S22, S23, S24, and S25). The *GCK*-proxied attenuated level of glucose had a negative effect on the PANSS percentage reduction. This was reflected in the significantly diminished PANSS total and negative, general psychopathology reduction rates ($P_{FDR} < 0.05$), and a suggestively significant decrease in PANSS-positive percentage reduction (p = 0.032). The corresponding β [95% CI] were −1.70 (−2.91, −0.50), −1.62 (−2.55, −0.70), −1.12 (−1.90, −0.34), and −1.06 (−2.02, −0.09) (to convert to per 10 mg/dL increase in glucose, multiply by 14.71) (Figure 3B; Table S26). The associations were robust in the sensitivity analysis applying the dichotomized outcome of the antipsychotic treatment efficacy (Table S27). Furthermore, rs3757840 was revealed as the top shared SNP in the colocalization analysis at the *GCK* locus between measured glucose and the reduction rates across PANSS total and three subscales (SNP.PP.H4 > 0.6) (Table S21; Figure S2).

The causal effect of *GCK*-predicted glucose was validated in the independent CAPEC cohort. Among the CAPEC participants, *GCK* GRS showed negative effects on the reduction rates of total PANSS and negative subscale ($P_{FDR} < 0.05$). It was also inversely associated with the percentage reduction of the PANSS positive subscale at a nominal significance level (p = 0.046). See the Tables S28, S29, S30, and S31 for details. Two-sample Mendelian randomization analyses further supported these causal associations (Tables S32 and S33).

Multi-omics evidence across neural and cardiometabolic systems supports the identified causal effects of *APOC3* and *GCK*

The causal effects of *APOC3* and *GCK* on the therapeutic outcomes were validated by analyses leveraging downstream molecular data across the circulatory and brain domains. Genetically predicted lowering of APOC3 protein concentrations in the blood was associated with a reduction in the TG (β [95% CI]: −6.01 [−8.20, −3.83]) and TC (β [95% CI]: −1.82 [−3.22, −0.43]) ($P_{FDR} < 0.05$). It also enhanced the probability of greater antipsychotic efficacy (OR [95% CI]: 1.11 [1.02, 1.22]) with suggestive significance (p = 0.016). The entire result was presented in Figure 3C and Tables S34–S36. In contrast, genetically predicted higher cerebrospinal fluid (CSF) GCK levels were associated with lowering glucose (β [95% CI]: −0.75 [−1.30, −0.19], P_{FDR} = 0.011). It was also associated with decreased negative symptom percentage reduction at nominal significance (p = 0.025). The corresponding β [95% CI] was −1.06 [−1.99, −0.13] (Figures 3D and S3; Tables S34, S37, and S38).

The mediation analysis showed that the effect of lowering APOC3 protein on better antipsychotic efficacy for negative symptoms was attributed to attenuated TG (OR [95% CI] of lowering TG conditioning on APOC3-protein quantitative trait loci (pQTL)-GRS: 1.30 [1.06, 1.60]). Little evidence showed the mediation effect of lowering TC (OR [95% CI]: 1.10 [0.96, 1.25]). Besides, the association between higher GCK in CSF and worse antipsychotic efficacy was mediated by decreased glucose (β [95% CI] of lowering glucose conditioning on GCK-pQTL-GRS: −1.43 [−2.43, −0.42]) (Table S39).

Notably, colocalization analysis again pinpointed rs651821 within *APOC3*—the SNP used to construct APOC3-pQTL-GRS—as the top shared variant between circulating APOC3 protein and negative-symptom efficacy (SNP.PP.H4 = 0.715). Likewise, rs3757840 in *GCK*, which was employed in the GCK-pQTL-GRS, emerged as the leading shared signal between the

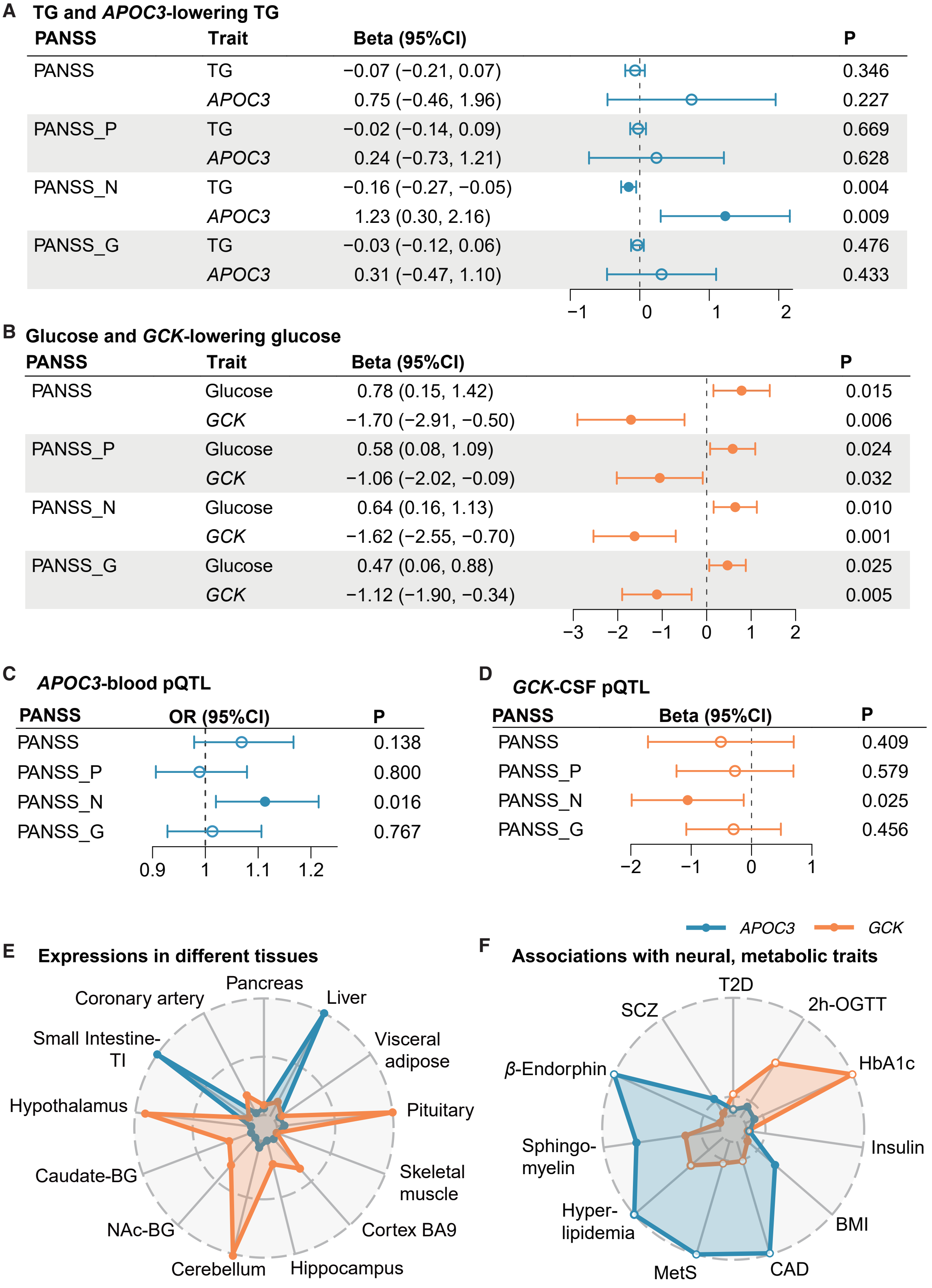

(legend on next page)

CSF GCK protein and percentage reduction of PANSS negative subscale (SNP.PP.H4 = 0.689) (Table S21; Figure S2).

Leveraging the gene expression profiles in different tissues, we captured that *APOC3* exhibited higher levels in the lipid-metabolic systems, involving the liver and small intestine. *GCK*, on the other hand, was predominantly expressed in the brain regions responsible for homeostatic and cognitive functions, including the hypothalamus, pituitary, and cortex. Given GCK's established roles in pancreatic and hepatic glucose metabolism, we compared its expression between these two tissues. We observed that *GCK* exhibited higher expression in the liver (transcripts per million [TPM] = 0.857) than its expression levels in the pancreas (TPM = 0.511) (Figure 3E; Table S40).

Interrogation of GWAS datasets further indicated that *APOC3* was associated with neuropeptide and membrane lipid composition, including β-endorphin and sphingomyelin. It was also associated with the susceptibility to metabolic syndrome (MetS), coronary artery disease, and SCZ (all $p < 0.05$). The *GCK* displayed stronger associations with the glycemic metabolic indicators, involving the 2-hour oral glucose tolerance test, hemoglobin A1c, and type 2 diabetes (all $p < 0.05$). Detailed results could be found in Figures 3F and S41. Based on individuals from the CAPOC study, we observed causal associations between drug-target *APOC3* and the level of aspartate aminotransferase, a typical enzyme reflecting liver function (β [95% CI]: 0.82 [0.07, 1.56]) (Table S42).

Subgroup analysis

The beneficial effect of *APOC3*-predicted TG lowering on negative symptom recovery was robust in participants with hyperlipemia ($n = 635$, β [95% CI]: 2.03 [0.44, 3.62], nominal $p = 0.013$). The detrimental effect of *GCK*-predicted glucose reduction on negative symptom recovery was consistent among those with prediabetes or diabetes ($n = 302$, β [95% CI]: -2.33 [-4.59, -0.07], nominal $p = 0.044$). Significant causal associations of *GCK*-proxied glucose with percentage reductions of PANSS total, positive, negative, and general psychopathology subscales were observed among individuals free of glucose dysfunction ($n = 1{,}809$, all $P_{FDR} < 0.05$) (Figures 4A and 4B; Tables S43 and S44).

When stratified by antipsychotic class, suggestive associations of *GCK*-predicted glucose were observed in patients receiving antipsychotics with stronger metabolic effect ($n = 1{,}056$). These associations spanned PANSS total (β [95% CI]: -1.82 [-3.46, -0.18]), negative (β [95% CI]: -1.38 [-2.68, -0.07]), and general psychopathology scales (β [95% CI]: -1.16 [-2.23, -0.09]). The causal effect of *APOC3*-predicted TG on the negative symptom relief was found in those using drugs with milder effects ($n = 1{,}055$, β [95% CI]: 1.65 [0.36, 2.94]) (all nominal $p < 0.05$, Figures 4C and 4D; Tables S43 and S44).

Among the participants with relapsed SCZ ($n = 1713$), *APOC3*-mediated TG reduction was positively associated with the negative-symptom improvement (β [95% CI]: 1.40 [0.38, 2.41], nominal $p = 0.007$). Glycemic lowering index by the *GCK* aligned with a weaker therapeutic efficacy, including lower reduction rates in total, negative, and general psychopathology PANSS scores ($P_{FDR} < 0.05$). *GCK*-related glucose lowering attenuated the percentage reduction of PANSS negative scales among males ($n = 1048$, $P_{FDR} = 0.008$). In females, it was associated with reduced percentage reduction in the total and general psychopathology scales ($n = 1{,}063$, nominal $p < 0.05$) (Figure S4; Tables S43 and S44). No significant interactive effects between the drug-target GRSs and the subgroup factors were observed (P for interaction >0.05) (Figures 4 and S4; Tables S43 and S44). Subgroup analysis in the CAPEC study was shown in Tables S45 and S46.

Joint and interaction associations between *APOC3*, *GCK*, and antipsychotic efficacy

Compared to the group with relatively lower GRSs of both targets ($n = 498$), participants with both elevated GRSs ($n = 502$) showed a greater reduction in measured TG. The respective β [95% CI] was -11.23 [-17.63, -4.82]. Evaluating the product of the continuous GRSs of *APOC3* and *GCK*, we did not observe the nonadditive interaction effect on the lipids ($p > 0.05$) (Figure S5; Tables S47 and S49).

Relative to individuals with low GRSs for both targets, those with an elevated GRS of *GCK* but an attenuated *APOC3* GRS ($n = 556$)—suggesting lower glycemia and higher lipid levels—experienced poorer antipsychotic efficacy. This was notable for total PANSS and the negative scale of PANSS, with β [95% CI] estimates of -3.46 (-6.89, -0.04) and -3.08 (-5.71, -0.44), showing suggestive significance ($p < 0.05$) (Tables S50 and S51). However, we observed no significant combined effect of the two targets on PANSS percentage reduction and no evidence of interaction ($p > 0.05$) (Figure S6; Tables S52 and S53).

Figure 3. Associations between *APOC3*-lowering TG, *GCK*-lowering glucose, and PANSS percentage reduction and multi-omics evidence
(A) Associations between the GRS of higher TG, *APOC3*-proxied lowering TG, and the percentage reduction of PANSS. (B) Associations between the GRS of higher glucose, *GCK*-proxied lowering glucose, and the percentage reduction of PANSS.
(C and D) Associations between genetically predicted APOC3 protein in the blood and GCK protein in CSF on the reduction rate of PANSS.
The beta (95% CI) represents the average percentage reduction in the PANSS for per standard deviation increase in the drug-target GRS. OR (95% CI) represents the probability of better antipsychotic efficacy (PANSS reduction rate > median value) for a standard deviation increase in the drug-target GRS. Error bars indicate the 95% CIs. Nominal p values are presented. The corresponding result can be found in Tables S35–S38.
(E) Expression levels (transcripts per million) of *APOC3* and *GCK* in different tissues from the GTEx v.10. Full results are in Table S40.
(F) Genetic associations between *APOC3*, *GCK*, and multiple neural, metabolic-related traits. For variants where the effect allele is associated with lower glucose or lipid levels, solid circles indicate positive associations with the traits, and open circles indicate negative associations. Full results are in Table S41.
Abbreviations: *APOC3*, apolipoprotein C3; *GCK*, glucokinase; TG, triglyceride; PANSS, Positive and Negative Syndrome Scale; N, negative scale; G, general psychopathology scale; P, positive scale; CSF, cerebrospinal fluid; pQTL, protein quantitative trait loci; VAT, visceral adipose tissue; BA9, Brodmann area 9; NAc, nucleus accumbens; BG, basal ganglia; T2D, type 2 diabetes; BMI, body mass index; 2h-OGTT, 2-hour oral glucose tolerance test; HbA1c, hemoglobin A1c; CAD, coronary artery disease; MetS, metabolic syndrome; SCZ, schizophrenia; GRS, genetic risk score.

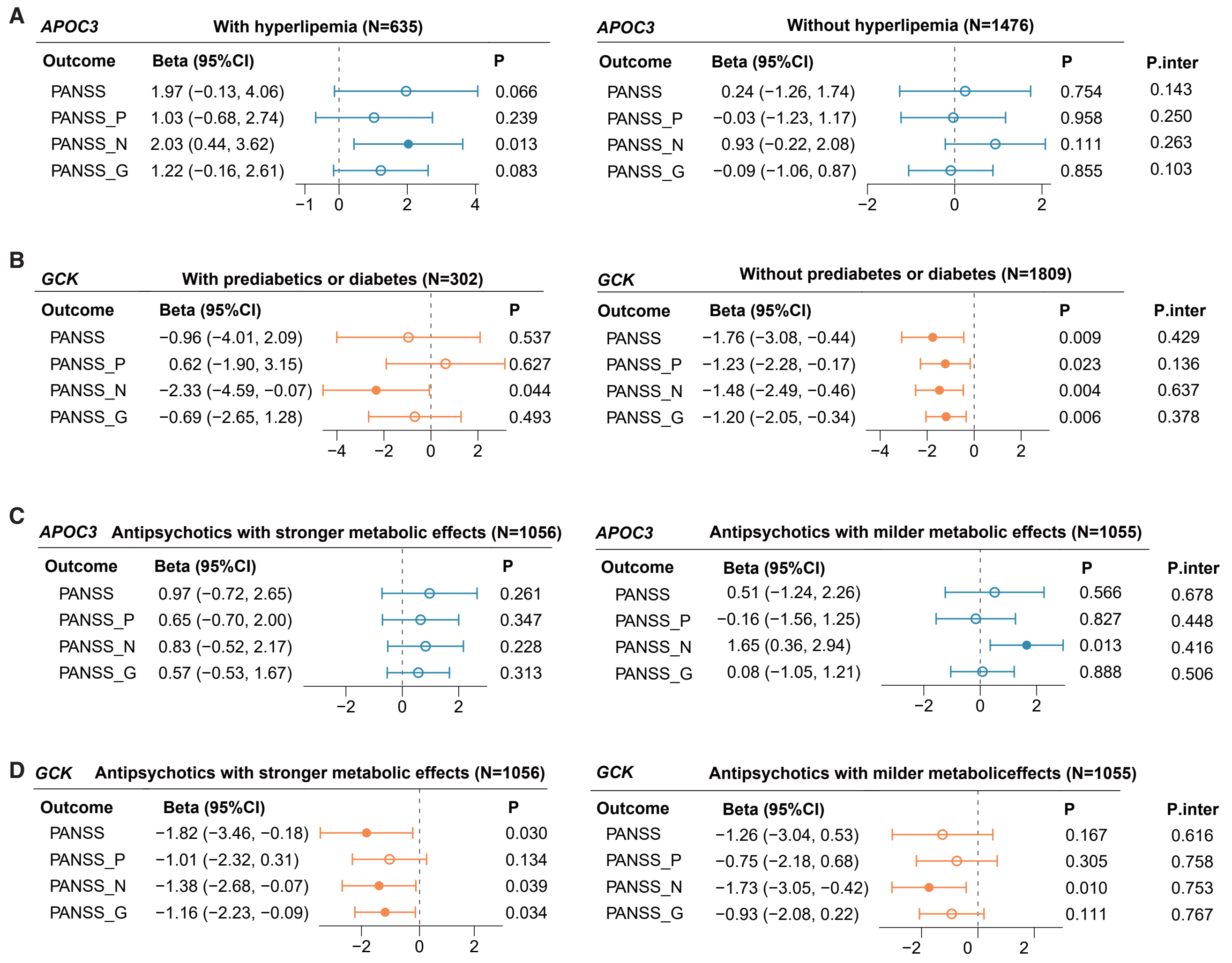

Figure 4. Stratified analysis by the baseline metabolic statuses and the assigned types of antipsychotics

(A and B) Stratified analysis by the baseline metabolic status. Patients with baseline TG ≥ 151 mg/dL (1.70 mmol/L) or baseline HDL ≤ 40 mg/dL (1.04 mmol/L) were categorized in the hyperlipemia group (N = 635); others were in the non-hyperlipemia group (N = 1,476). Patients with baseline glucose ≥ 100.8 mg/dL (5.6 mmol/L) were classified into the prediabetes or diabetes group (N = 302); the remaining patients were in the non-diabetes or prediabetes group (N = 1,809).
(C and D) Stratified analysis by the assigned types of antipsychotics. Patients prescribed risperidone, olanzapine, or quetiapine were grouped as receiving medication with stronger metabolic side effects (N = 1056), while others (prescribed aripiprazole, ziprasidone, perphenazine, or haloperidol) comprised the milder-metabolic-effect group (N = 1055). Full results are in Tables S43 and S44.
The beta (95% CI) represents the average percentage reduction in the PANSS for per standard deviation increase in the drug-target GRS. Error bars indicate the 95% CIs. Nominal p values are presented. P.inter represented the interaction test between the stratified factor and drug-target GRS.
Abbreviations: *APOC3*, apolipoprotein C3; *GCK*, glucokinase; TG, triglyceride; PANSS, Positive and Negative Syndrome Scale; N, negative scale; G, general psychopathology scale; P, positive scale; GRS, genetic risk score.

DISCUSSION

We performed a drug-target genetic association study to assess the independent and combined causal effects of lipid- and glucose-lowering drug targets on antipsychotic treatment efficacy among individuals with SCZ. We identified that *APOC3*-proxied lowering of TG and TC could enhance the improvement of negative symptoms, whereas *GCK* was associated with poor treatment outcomes, through the attenuated glucose concentration. By leveraging multi-omics data in the brain and cardiometabolic systems, we validated the influences of *APOC3* and *GCK* on the efficacy of antipsychotics. The further colocalization analysis strengthened their on-target effects. The *APOC3* and *GCK* jointly reduced TG, without a combined impact on antipsychotic efficacy. Our study provided genetic evidence for the potential benefits and pharmacovigilance considerations of cardiometabolic agents on antipsychotic efficacy.

APOC3 plays a key role in TG metabolism. In the present study, it showed higher expression in digestive tissues, particularly the liver and small intestine. Our findings were similar to previous mechanism studies, which reported that APOC3 could not only inhibit the lipoprotein lipase and block the

hepatic uptake of TG-rich lipoprotein remnants[19] but also modulate chylomicron formation and intestinal secretion of dietary TG.[20] The APOC3 is the primary inhibitory target of volanesorsen and olezarsen, two distinct antisense oligonucleotides targeting elevated TG in chylomicronemia.[21–23] Compared with the healthy controls, *APOC3* expression is downregulated in patients with SCZ, suggesting its diagnostic relevance for the disorder.[24] Notably, among those affected by SCZ, our findings were aligned with previous genetic association studies. Two of the studies reported that the genetic variations of *APOC3* were associated with attenuated serum TG,[25] as well as the risk of MetS.[26]

Focusing on the impact of APOC3 on the progression of SCZ, a recent meta-analysis involving nine RCTs (533 patients with SCZ) indicated that adjunctive statin therapy might benefit the reduction rates of PANSS negative and total scores.[11] This is consistent with our findings demonstrating a protective effect of *APOC3*-proxied lipid lowering. From a peripheral perspective, a previous study (N = 80) showed that the polymorphisms in *APOC3* were related to the olanzapine pharmacokinetic variability.[27] Given the lipophilic nature of olanzapine, *APOC3*-mediated TG transport may influence its pharmacokinetic distribution. Moreover, inhibition of APOC3 in the liver and small intestine, with consequent reductions in remnant lipid particles and microvascular inflammatory injury,[28,29] could improve blood-brain barrier integrity and the central availability of antipsychotic drugs and nutrients.[30,31] In addition, lowering TG in blood helps normalize abnormal inflammatory biomarkers—such as C-reactive protein[32] and circulating cytokines[33]—and reduce oxidative stress.[34] It may also modulate the neurochemical mediators (e.g., leptin),[34] thereby facilitating the central nervous system recovery and functional improvement in SCZ. At the central level, we observed a significant association between *APOC3* and β-endorphin, and higher expressions of *APOC3* in the hypothalamus and cortex. Our findings imply that APOC3 might contribute to neuroregulatory "lipid-opioid-dopamine" networks with potential impact on negative symptom recovery.[35,36] Besides, a previous study indicated that APOC3 might function as a hypothalamic lipid-sensing modulator.[37] The APOC3-inhibition-driven relief of peripheral metabolic stress could lessen the metabolic strain on central energy-reward circuits.[38] This, in turn, could support the remodeling of dopamine- and emotion-related networks relevant to antipsychotic response.[39]

The glucokinase is a hexokinase-family enzyme that facilitates the conversion of glucose to glucose-6-phosphate, the initiating step in most glucose-metabolic pathways. In the present study, *GCK* was expressed more abundantly in hepatic tissue than in the pancreas, paralleling prior *in vivo* evidence. A liver-targeted *GCK* agonist reduced glycemia without raising the circulating insulin,[40] whereas overexpressed hepatic *GCK* accelerated glucose clearance in insulin-null type 1 diabetic mice.[41] These findings underscored an insulin-independent role for glucokinase in liver glucose homeostasis. The glucokinase activator—dorzagliatin—boosts glucose sensitivity and helps maintain glucose homeostasis in individuals with type 2 diabetes.[42]

Our findings indicated that the *GCK*-induced glucose lowering attenuated the antipsychotic efficacy. Consistent with our results, a previous clinical study (64 patients with SCZ, 33 healthy controls) reported that the dysfunction of the glucokinase/glucose-6-phosphate metabolism pathway could lead to cytotoxicity in neurons and cognitive impairment in SCZ.[43] We detected dominant expression of *GCK* within hypothalamic and cortical regions, consistent with its role as a neuronal glucose sensor in the hypothalamus.[44,45] *GCK* gain-of-function variants may both chronically lower peripheral glucose levels and lower the glycemic threshold at which central glucose-sensing neurons initiate counterregulatory responses.[44] This may increase the risk of severe hypoglycaemia[46] and predispose the brain to recurrent neuroglycopenic episodes (brain energy insufficiency).[46,47] These episodes can lead to oxidative and inflammatory stress[46] and neurotoxic injury[48] in hippocampal,[49] prefrontal,[50] and striatal circuits,[51] potentially undermining antipsychotic efficacy.[46,47] In parallel, hypothalamic GCK-positive neurons interface with autonomic, HPA,[52,53] and reward-related networks that regulate mesolimbic dopamine.[54,55] Recurrent low-glucose states may perturb reward dopamine signaling and hinder antipsychotic efficacy.[56] In the peripheral system, GCK functions as a glucose-sensing enzyme in the liver.[40,57] Accordingly, genetically predicted increases in GCK activity enhance hepatic glucose uptake and disposal and predispose individuals to recurrent hypoglycemic episodes. This could contribute to neuroglycopenia and other central disturbances, fostering unfavorable neurobiological conditions for SCZ recovery.[46,47]

From a translational perspective, our findings provide genetic support for APOC3 inhibition (volanesorsen and olezarsen) as a candidate pathway that may beneficially modulate antipsychotic efficacy. The clinical reliability of this finding warrants further consideration. Volanesorsen and olezarsen are administered subcutaneously to lower APOC3 and TG. Both drugs have undergone phase 3 evaluation in familial chylomicronemia syndrome (FCS),[21,23] and olezarsen is approved in America and Europe as a diet adjunct in adults with FCS.[58] Our genetic analyses indicated that *APOC3*-proxied TG lowering did not materially affect common safety indicators, including blood pressure, QTc interval, renal and hepatic indices, and prolactin levels. Such lines of evidence collectively support the clinical safety of these two drugs. However, the potential adverse effects of these drugs (e.g., thrombocytopenia)[21,23] and the pleiotropic effects of APOC3 in the inflammatory and endothelial pathways[28] have not been systematically evaluated in people with SCZ. On the other hand, although these two drugs lower TG predominantly in the liver and have limited brain penetration, APOC3-inhibition-related TG lowering may still influence antipsychotic response through downstream metabolic and neurovascular pathways. It may modify the peripheral distribution of lipophilic antipsychotics[27] and attenuate systemic and cerebrovascular inflammation.[33] Such changes could help preserve the blood-brain barrier integrity,[30] increase central antipsychotic availability, and ultimately facilitate recovery in SCZ. Our observation of *APOC3* expression in the brain and its association with β-endorphin provides supportive evidence that APOC3 may interface with opioid-related neuroregulatory circuits[35,36] and central lipid-sensing pathways,[37] contributing to central nervous system improvement. Future proof-of-concept RCTs in SCZ populations with comorbid hypertriglyceridemia

are required to assess the feasibility, safety, and potential clinical benefit of APOC3-targeted interventions, incorporating detailed monitoring of psychiatric outcomes (particularly negative symptoms) to improve the current prescription guidelines. By contrast, our *GCK*-related glucose-lowering findings call for clinical vigilance, and any future use of dorzagliatin in SCZ populations should pay particular attention to potential adverse effects on antipsychotic response.

The drug-target genetic association strategy, which applies genetic variants within the ±100 kb region of each target, could simulate the on-target effect of the corresponding drugs. This approach can also minimize the risk of inducing horizontal pleiotropy due to the mixed variants from genes nearby.[59] Our colocalization analysis showed that both *APOC3*- and *GCK*-linked antipsychotic efficacy signals shared causal variants with their respective pQTL and with lipids or glucose. The mediation analysis indicated that such effects of these target proteins on SCZ recovery were attributed to the lowering of TG or glucose. Together, these investigations further support predominantly on-target causal effects of *APOC3*- and *GCK*-related lipid and glucose lowering on antipsychotic efficacy. The 2×2 factorial drug-target framework further mimics simultaneous interventions of lipid- and glucose-modifying drugs, allowing us to evaluate their combined effect.[18]

Our stratified analysis implied that, among patients complicated with lipid dysfunction, *APOC3* could be considered as a lipid-lowering target that may enhance negative-symptom improvement. In contrast, *GCK* emerged as a cautionary glucose-modifying target, as it was associated with worse improvement in negative symptoms, among patients with prediabetes or diabetes. Nevertheless, several antipsychotics, such as olanzapine, are known for their common metabolic side effects.[60] Whether glucose-lowering agents should be coadministered to prevent antipsychotic-induced metabolic disturbances remains an active area of investigation.[61,62] Our results raise concerns for using GCK-targeting glucose-lowering drugs as adjunctive treatments, since they may exacerbate psychotic symptoms when co-prescribed with high-metabolic-risk antipsychotics. Besides, the lipid-lowering effect of *APOC3* appeared especially advantageous in patients taking agents with minimal metabolic liabilities.

In summary, based on large-scale longitudinal data of Han Chinese patients with SCZ, we assessed both the individual and combined associations of the metabolism-modifying agents on 6-week antipsychotic treatment efficacy. Validation across two separate cohorts underscored the robustness of our major findings. We revealed that the *APOC3*-proxied lipid-lowering levels might enhance the efficacy of antipsychotic treatments in SCZ, whereas the attenuated glucose related to the *GCK* variants could elevate the risk of worsening therapeutic outcomes. Our findings suggested volanesorsen and olezarsen as potential lipid-modifying adjunctive candidates, while the glucose-lowering drug—dorzagliatin—warrants clinical prudence in managing patients with concurrent psychiatric and metabolic disturbance. The genetic insights from this study pave the way for tailored interventions and therapeutic targets in clinical psychiatry.

Limitations of the study

We acknowledged limitations for further discussion. First, genetic instruments represent lifelong exposure to agents, which differs from short-term randomized pharmacologic trials. In addition, real-world compounds might have off-target effects and yield outcomes that diverge from present findings, which need to be clarified by future clinical studies. And residual confounding cannot be fully excluded. Second, due to limited genome-wide significant pQTLs in East Asians, we selected lipid- and glucose-associated SNPs within target loci; results remained consistent in sensitivity analysis using pQTLs meeting $p < 0.005$. Currently available brain-based pQTL datasets are derived exclusively from Europeans, despite similar allele frequencies across ancestries,[63] East Asian resources are warranted to validate these findings. Because our study was conducted in East Asians, its generalizability to other ancestries remains uncertain. Extensions in diverse ancestries are required to replicate our findings and explore potential ancestry-specific effects. Third, drug-target MR aims to prioritize targets that might influence SCZ progression and inform further clinical investigations.[64,65] Focusing solely on associations surviving multiple testing might overlook some biologically meaningful targets. Given our limited sample size—particularly in the prediabetes/diabetes subgroup—relative to prior drug-target MR studies,[66,67] we also considered nominal results and interpreted results in the context of effect sizes and 95% CI.[68,69]

RESOURCE AVAILABILITY

Lead contact

Further information and requests for resources should be directed to and will be fulfilled by the lead contact, Weihua Yue (dryue@bjmu.edu.cn).

Materials availability

This study did not generate unique reagents.

Data and code availability

- The GWAS and expression quantitative trait loci (eQTL) summary statistics were publicly available. GWAS summary statistics from the Biobank of Japan could be obtained from the corresponding websites (https://pheweb.jp/). GWAS summary statistics for lipids were obtained from the Global Lipids Genetics Consortium (https://csg.sph.umich.edu/willer/public/glgc-lipids2021/results/ancestry_specific/). GWAS summary statistics for type 2 diabetes were from the Type 2 Diabetes Global Genetics Initiative (https://diagram-consortium.org/downloads.html). Other GWAS summary statistics were downloaded from the GWAS Catalog (https://www.ebi.ac.uk/gwas/). The eQTL data from the Genotype Tissue Expression (GTEx) were obtained from the corresponding portals (https://gtexportal.org/home/downloads/). The blood-pQTL summary statistics from the Guangzhou Nutrition and Health study were obtained from the website https://omics.lab.westlake.edu.cn/data/proteins/phenotypes. The CSF-pQTL was downloaded from the https://www.researchsquare.com/article/rs-2814616/v1.
- Constrained by the local law on the management of human genetic resources and the requirements of the research project, the sharing of individual-level data of the Chinese Antipsychotics Pharmacogenomics Consortium study and the Chinese Antipsychotics Pharmacogenetics Consortium was restricted from public access. The data that support the findings of this study are available from the lead contact, upon reasonable request with a proposal. All requests must be approved by the relevant ethics boards and data custodians.

- No custom code is reported in this study. Any additional information required to reanalyze the data reported in this work paper is available from the lead contact upon request.

ACKNOWLEDGMENTS

We want to acknowledge the participants and investigators of the Chinese Antipsychotics Pharmacogenomics Consortium study and Chinese Antipsychotics Pharmacogenetics Consortium, Global Lipids Genetics Consortium, Taiwan Biobank, Biobank of Japan, Type 2 Diabetes Global Genetics Initiative, Guangzhou Nutrition and Health study, Million Veteran Program, UK Biobank, Meta-Analysis of Glucose and Insulin-related Traits Consortium, The Qatar Genome Program Research Consortium, the Adult Genotype Tissue Expression Project, as well as the genome-wide association study conducted by Chen et al., Zhen et al., Park et al., and Cruchaga et al.

This study was supported by the National Key R&D Program of China (2025YFC2511200), National Natural Science Foundation of China (82330042, 82441005, 82301687, 82501828, 82501802), National Key R&D Program of China (2023YFE0119400, 2021YFF1201100), Capital's Funds for Health Improvement and Research (2024-1-4111), Fundamental Research Funds for the Central Universities (Peking University Medicine Fund for world's leading discipline and discipline cluster development, BMU2022DJXK007), Beijing Municipal Health Commission Research Ward Programme (3rd batch), Non-profit Central Research Institute Fund of Chinese Academy of Medical Sciences (2023-PT320-08), Beijing Nova Program (20230484425), Youth Talent Support Program of the China Association for Science and Technology, Beijing Natural Science Foundation (7254462), and Peking University Health Science Center Outstanding Doctoral Student Innovation Fund (BMU2025BSS0011).

AUTHOR CONTRIBUTIONS

W.Y. is the primary investigator. W.Y. and Y.S. designed the study, acquired funding, and contributed to interpretation of data. Y. Zhu and R.Y. analyzed the data. Y. Zhu wrote the first draft of the article. Z.L., Y. Zhang, Z.K., X.F., G.Z., J.S., J.G., T.Y., and Y.Y. were responsible for phenotype cleaning, preparation of the tables and figures, and providing further data interpretation. W.Y. and Y.S. gave interpretation for the study results and the development of the study conclusion. All authors contributed to drafting the work or critically revising it for important intellectual content and made substantial contributions to the concept and design of the study and data acquisition, analysis, and interpretation.

DECLARATION OF INTERESTS

The authors declare no competing interests.

DECLARATION OF GENERATIVE AI AND AI-ASSISTED TECHNOLOGIES IN THE WRITING PROCESS

During the preparation of this work, the authors used ChatGPT in order to improve language and readability. After using this tool/service, the authors reviewed and edited the content as needed and take full responsibility for the content of the publication.

STAR★METHODS

Detailed methods are provided in the online version of this paper and include the following:

- KEY RESOURCES TABLE
- EXPERIMENTAL MODEL AND STUDY PARTICIPANT DETAILS
- METHOD DETAILS
  - Genotyping and quality control
  - Drug-target GRS
  - Outcomes
  - Covariates
- QUANTIFICATION AND STATISTICAL ANALYSIS
  - Single drug-target analysis
  - Stratified analysis and independent validation

SUPPLEMENTAL INFORMATION

Supplemental information can be found online at https://doi.org/10.1016/j.xcrm.2026.102653.

Received: September 17, 2025
Revised: December 28, 2025
Accepted: February 4, 2026
Published: March 17, 2026

REFERENCES

1. Correll, C.U., Solmi, M., Veronese, N., Bortolato, B., Rosson, S., Santonastaso, P., Thapa-Chhetri, N., Fornaro, M., Gallicchio, D., Collantoni, E., et al. (2017). Prevalence, incidence and mortality from cardiovascular disease in patients with pooled and specific severe mental illness: a large-scale meta-analysis of 3,211,768 patients and 113,383,368 controls. World Psychiatry *16*, 163–180. https://doi.org/10.1002/wps.20420.
2. Vancampfort, D., Stubbs, B., Mitchell, A.J., De Hert, M., Wampers, M., Ward, P.B., Rosenbaum, S., and Correll, C.U. (2015). Risk of metabolic syndrome and its components in people with schizophrenia and related psychotic disorders, bipolar disorder and major depressive disorder: a systematic review and meta-analysis. World Psychiatry *14*, 339–347. https://doi.org/10.1002/wps.20252.
3. Vancampfort, D., Wampers, M., Mitchell, A.J., Correll, C.U., De Herdt, A., Probst, M., and De Hert, M. (2013). A meta-analysis of cardio-metabolic abnormalities in drug naïve, first-episode and multi-episode patients with schizophrenia versus general population controls. World Psychiatry *12*, 240–250. https://doi.org/10.1002/wps.20069.
4. Rødevand, L., Rahman, Z., Hindley, G.F.L., Smeland, O.B., Frei, O., Tekin, T.F., Kutrolli, G., Bahrami, S., Hoseth, E.Z., Shadrin, A., et al. (2023). Characterizing the Shared Genetic Underpinnings of Schizophrenia and Cardiovascular Disease Risk Factors. Am. J. Psychiatry *180*, 815–826. https://doi.org/10.1176/appi.ajp.20220660.
5. Firth, J., Siddiqi, N., Koyanagi, A., Siskind, D., Rosenbaum, S., Galletly, C., Allan, S., Caneo, C., Carney, R., Carvalho, A.F., et al. (2019). The Lancet Psychiatry Commission: a blueprint for protecting physical health in people with mental illness. Lancet Psychiatry *6*, 675–712. https://doi.org/10.1016/s2215-0366(19)30132-4.
6. Tuccori, M., Montagnani, S., Mantarro, S., Capogrosso-Sansone, A., Ruggiero, E., Saporiti, A., Antonioli, L., Fornai, M., and Blandizzi, C. (2014). Neuropsychiatric Adverse Events Associated with Statins: Epidemiology, Pathophysiology, Prevention and Management. CNS Drugs *28*, 249–272. https://doi.org/10.1007/s40263-013-0135-1.
7. Cham, S., Koslik, H.J., and Golomb, B.A. (2016). Mood, Personality, and Behavior Changes During Treatment with Statins: A Case Series. Drug Saf. Case Rep. *3*, 1. https://doi.org/10.1007/s40800-015-0024-2.
8. Venkatasubramanian, G., Arasappa, R., Rao, N.P., Behere, R.V., and Gangadhar, B.N. (2010). Adjuvant metformin worsens psychosis in schizophrenia: a case report. Prim. Care Companion J. Clin. Psychiatry *12*, PCC.09l00801. https://doi.org/10.4088/PCC.09l00801yel.
9. Macklin, J., and Morrison, G. (2011). Survey of general practitioners' attitudes to prescribing statins in different patient groups: a web-based survey. Scott. Med. J. *56*, 33–35. https://doi.org/10.1258/smj.2010.010016.
10. Mitchell, A.J., Lord, O., and Malone, D. (2012). Differences in the prescribing of medication for physical disorders in individuals with v. without mental illness: meta-analysis. Br. J. Psychiatry *201*, 435–443. https://doi.org/10.1192/bjp.bp.111.094532.

11. Peng, T.R., Lin, H.H., Lee, M.C., and Chen, S.M. (2024). Statins as an adjuvant therapy for patients with schizophrenia: An up-to-date systematic review and meta-analysis. Gen. Hosp. Psychiatry *89*, 75–83. https://doi.org/10.1016/j.genhosppsych.2024.05.001.

12. Siskind, D., Baker, A., Arnautovska, U., Warren, N., Russell, A., DeMonte, V., Halstead, S., Iyer, R., Korman, N., McKeon, G., et al. (2025). Efficacy and safety of semaglutide versus placebo for people with schizophrenia on clozapine with obesity (COaST): a phase 2, multi-centre, participant and investigator- blinded, randomised controlled trial in Australia. Lancet Psychiatry *12*, 493–503. https://doi.org/10.1016/s2215-0366(25)00129-4.

13. Liang, H., Li, H., Hu, Y., Li, S., Lü, L., and Song, X. (2016). [Effects of Topiramate for atypical antipsychotic-induced body weight gain and metabolic adversities: a systematic review and meta-analysis]. Zhonghua Yixue Zazhi *96*, 216–223. https://doi.org/10.3760/cma.j.issn.0376-2491.2016.03.014.

14. Iranpour, N., Zandifar, A., Farokhnia, M., Goguol, A., Yekehtaz, H., Khodaie-Ardakani, M.R., Salehi, B., Esalatmanesh, S., Zeionoddini, A., Mohammadinejad, P., et al. (2016). The effects of pioglitazone adjuvant therapy on negative symptoms of patients with chronic schizophrenia: a double-blind and placebo-controlled trial. Hum. Psychopharmacol. *31*, 103–112. https://doi.org/10.1002/hup.2517.

15. Davey Smith, G., and Ebrahim, S. (2005). What can mendelian randomisation tell us about modifiable behavioural and environmental exposures? BMJ (Clinical research ed.) *330*, 1076–1079. https://doi.org/10.1136/bmj.330.7499.1076.

16. Katan, M.B. (1986). Apolipoprotein E isoforms, serum cholesterol, and cancer. Lancet (London, England) *1*, 507–508. https://doi.org/10.1016/s0140-6736(86)92972-7.

17. Walker, V.M., Davey Smith, G., Davies, N.M., and Martin, R.M. (2017). Mendelian randomization: a novel approach for the prediction of adverse drug events and drug repurposing opportunities. Int. J. Epidemiol. *46*, 2078–2089. https://doi.org/10.1093/ije/dyx207.

18. Alver, M., Kasela, S., Haring, L., Luitva, L.B., Estonian Biobank Research Team; Möls, M., Fischer, K., Möls, M., and Milani, L. (2024). Genetic predisposition and antipsychotic treatment effect on metabolic syndrome in schizophrenia: a ten-year follow-up study using the Estonian Biobank. Lancet Reg. Health. Eur. *41*, 100914. https://doi.org/10.1016/j.lanepe.2024.100914.

19. Chan, D.C., Chen, M.M., Ooi, E.M.M., and Watts, G.F. (2008). An ABC of apolipoprotein C-III: a clinically useful new cardiovascular risk factor? Int. J. Clin. Pract. *62*, 799–809. https://doi.org/10.1111/j.1742-1241.2007.01678.x.

20. Borén, J., Packard, C.J., and Taskinen, M.R. (2020). The Roles of ApoC-III on the Metabolism of Triglyceride-Rich Lipoproteins in Humans. Front. Endocrinol. *11*, 474. https://doi.org/10.3389/fendo.2020.00474.

21. Witztum, J.L., Gaudet, D., Freedman, S.D., Alexander, V.J., Digenio, A., Williams, K.R., Yang, Q., Hughes, S.G., Geary, R.S., Arca, M., et al. (2019). Volanesorsen and Triglyceride Levels in Familial Chylomicronemia Syndrome. N. Engl. J. Med. *381*, 531–542. https://doi.org/10.1056/NEJMoa1715944.

22. TG and HDL Working Group of the Exome Sequencing Project National Heart Lung and Blood Institute; Crosby, J., Peloso, G.M., Auer, P.L., Crosslin, D.R., Stitziel, N.O., Lange, L.A., Lu, Y., Tang, Z.z., Zhang, H., et al. (2014). Loss-of-function mutations in APOC3, triglycerides, and coronary disease. N. Engl. J. Med. *371*, 22–31. https://doi.org/10.1056/NEJMoa1307095.

23. Stroes, E.S.G., Alexander, V.J., Karwatowska-Prokopczuk, E., Hegele, R.A., Arca, M., Ballantyne, C.M., Soran, H., Prohaska, T.A., Xia, S., Ginsberg, H.N., et al. (2024). Olezarsen, Acute Pancreatitis, and Familial Chylomicronemia Syndrome. N. Engl. J. Med. *390*, 1781–1792. https://doi.org/10.1056/NEJMoa2400201.

24. Rodrigues, J.E., Martinho, A., Santa, C., Madeira, N., Coroa, M., Santos, V., Martins, M.J., Pato, C.N., Macedo, A., and Manadas, B. (2022). Systematic Review and Meta-Analysis of Mass Spectrometry Proteomics Applied to Human Peripheral Fluids to Assess Potential Biomarkers of Schizophrenia. Int. J. Mol. Sci. *23*, 4917. https://doi.org/10.3390/ijms23094917.

25. Smith, R.C., Segman, R.H., Golcer-Dubner, T., Pavlov, V., and Lerer, B. (2008). Allelic variation in ApoC3, ApoA5 and LPL genes and first and second generation antipsychotic effects on serum lipids in patients with schizophrenia. Pharmacogenomics J. *8*, 228–236. https://doi.org/10.1038/sj.tpj.6500474.

26. Boiko, A.S., Mednova, I.A., Kornetova, E.G., Semke, A.V., Bokhan, N.A., Loonen, A.J.M., and Ivanova, S.A. (2019). Apolipoprotein serum levels related to metabolic syndrome in patients with schizophrenia. Heliyon *5*, e02033. https://doi.org/10.1016/j.heliyon.2019.e02033.

27. Zubiaur, P., Soria-Chacartegui, P., Koller, D., Navares-Gómez, M., Ochoa, D., Almenara, S., Saiz-Rodríguez, M., Mejía-Abril, G., Villapalos-García, G., Román, M., et al. (2021). Impact of polymorphisms in transporter and metabolizing enzyme genes on olanzapine pharmacokinetics and safety in healthy volunteers. Biomed. Pharmacother. *133*, 111087. https://doi.org/10.1016/j.biopha.2020.111087.

28. Tao, Y., Xiong, Y., Wang, H., Chu, S., Zhong, R., Wang, J., Wang, G., Ren, X., and Yu, J. (2016). APOC3 induces endothelial dysfunction through TNF-α and JAM-1. Lipids Health Dis. *15*, 153. https://doi.org/10.1186/s12944-016-0326-0.

29. Giammanco, A., Spina, R., Cefalù, A.B., and Averna, M. (2023). APOC-III: a Gatekeeper in Controlling Triglyceride Metabolism. Curr. Atheroscler. Rep. *25*, 67–76. https://doi.org/10.1007/s11883-023-01080-8.

30. Sheikh, M.H., Errede, M., d'Amati, A., Khan, N.Q., Fanti, S., Loiola, R.A., McArthur, S., Purvis, G.S.D., O'Riordan, C.E., Ferorelli, D., et al. (2022). Impact of metabolic disorders on the structural, functional, and immunological integrity of the blood-brain barrier: Therapeutic avenues. FASEB J. *36*, e22107. https://doi.org/10.1096/fj.202101297R.

31. Wang, Q., Zhou, W., and Zhang, J.; Alzheimer's Disease Neuroimaging Initiative (2019). Higher Apolipoprotein C-III Levels in Cerebrospinal Fluid are Associated with Slower Cognitive Decline in Mild Cognitive Impairment. J. Alzheimers Dis. *67*, 961–969. https://doi.org/10.3233/jad-181096.

32. Esposito, K., Marfella, R., Ciotola, M., Di Palo, C., Giugliano, F., Giugliano, G., D'Armiento, M., D'Andrea, F., and Giugliano, D. (2004). Effect of a mediterranean-style diet on endothelial dysfunction and markers of vascular inflammation in the metabolic syndrome: a randomized trial. JAMA *292*, 1440–1446. https://doi.org/10.1001/jama.292.12.1440.

33. Goldsmith, D.R., Massa, N., Miller, B.J., Miller, A.H., and Duncan, E. (2021). The interaction of lipids and inflammatory markers predict negative symptom severity in patients with schizophrenia. NPJ Schizophr. *7*, 50. https://doi.org/10.1038/s41537-021-00179-8.

34. Adamowicz, K., Mazur, A., Mak, M., Samochowiec, J., and Kucharska-Mazur, J. (2020). Metabolic Syndrome and Cognitive Functions in Schizophrenia-Implementation of Dietary Intervention. Front. Psychiatry *11*, 359. https://doi.org/10.3389/fpsyt.2020.00359.

35. Thareja, G., Belkadi, A., Arnold, M., Albagha, O.M.E., Graumann, J., Schmidt, F., Grallert, H., Peters, A., Gieger, C., Consortium, T.Q.G.P.R., and Suhre, K. (2023). Differences and commonalities in the genetic architecture of protein quantitative trait loci in European and Arab populations. Hum. Mol. Genet. *32*, 907–916. https://doi.org/10.1093/hmg/ddac243.

36. Pilozzi, A., Carro, C., and Huang, X. (2020). Roles of β-Endorphin in Stress, Behavior, Neuroinflammation, and Brain Energy Metabolism. Int. J. Mol. Sci. *22*, 338. https://doi.org/10.3390/ijms22010338.

37. Kim, H.K., Shin, M.S., Youn, B.S., Kang, G.M., Gil, S.Y., Lee, C.H., Choi, J.H., Lim, H.S., Yoo, H.J., and Kim, M.S. (2015). Regulation of energy balance by the hypothalamic lipoprotein lipase regulator Angptl3. Diabetes *64*, 1142–1153. https://doi.org/10.2337/db14-0647.

38. Magnan, C., Levin, B.E., and Luquet, S. (2015). Brain lipid sensing and the neural control of energy balance. Mol. Cell. Endocrinol. *418*, 3–8. https://doi.org/10.1016/j.mce.2015.09.019.

CellPress
OPEN ACCESS

39. Baik, J.H. (2021). Dopaminergic Control of the Feeding Circuit. Endocrinol. Metab. *36*, 229–239. https://doi.org/10.3803/EnM.2021.979.

40. Erion, D.M., Lapworth, A., Amor, P.A., Bai, G., Vera, N.B., Clark, R.W., Yan, Q., Zhu, Y., Ross, T.T., Purkal, J., et al. (2014). The hepatoselective glucokinase activator PF-04991532 ameliorates hyperglycemia without causing hepatic steatosis in diabetic rats. PLoS One *9*, e97139. https://doi.org/10.1371/journal.pone.0097139.

41. Morral, N., McEvoy, R., Dong, H., Meseck, M., Altomonte, J., Thung, S., and Woo, S.L.C. (2002). Adenovirus-mediated expression of glucokinase in the liver as an adjuvant treatment for type 1 diabetes. Hum. Gene Ther. *13*, 1561–1570. https://doi.org/10.1089/10430340260201653.

42. Zhu, D., Li, X., Ma, J., Zeng, J., Gan, S., Dong, X., Yang, J., Lin, X., Cai, H., Song, W., et al. (2022). Dorzagliatin in drug-naïve patients with type 2 diabetes: a randomized, double-blind, placebo-controlled phase 3 trial. Nat. Med. *28*, 965–973. https://doi.org/10.1038/s41591-022-01802-6.

43. Luo, X., Dong, J., and Li, T. (2025). The Role of CCL11-CCR3 Induced Mitochondrial Dysfunction and Oxidative Stress in Cognitive Impairment in Early-onset Schizophrenia: Insights from Preclinical Studies. Inflammation *48*, 4533–4548. https://doi.org/10.1007/s10753-025-02344-y.

44. De Backer, I., Hussain, S.S., Bloom, S.R., and Gardiner, J.V. (2016). Insights into the role of neuronal glucokinase. Am. J. Physiol. Endocrinol. Metab. *311*, E42–E55. https://doi.org/10.1152/ajpendo.00034.2016.

45. Roh, E., Song, D.K., and Kim, M.-S. (2016). Emerging role of the brain in the homeostatic regulation of energy and glucose metabolism. Exp. Mol. Med. *48*, e216. https://doi.org/10.1038/emm.2016.4.

46. McCrimmon, R.J. (2021). Consequences of recurrent hypoglycaemia on brain function in diabetes. Diabetologia *64*, 971–977. https://doi.org/10.1007/s00125-020-05369-0.

47. Amiel, S.A. (2021). The consequences of hypoglycaemia. Diabetologia *64*, 963–970. https://doi.org/10.1007/s00125-020-05366-3.

48. Pettersen, J.C., Litchfield, J., Neef, N., Schmidt, S.P., Shirai, N., Walters, K.M., Enerson, B.E., Chatman, L.A., and Pfefferkorn, J.A. (2014). The relationship of glucokinase activator-induced hypoglycemia with arteriopathy, neuronal necrosis, and peripheral neuropathy in nonclinical studies. Toxicol. Pathol. *42*, 696–708. https://doi.org/10.1177/0192623314526006.

49. Won, S.J., Yoo, B.H., Kauppinen, T.M., Choi, B.Y., Kim, J.H., Jang, B.G., Lee, M.W., Sohn, M., Liu, J., Swanson, R.A., and Suh, S.W. (2012). Recurrent/moderate hypoglycemia induces hippocampal dendritic injury, microglial activation, and cognitive impairment in diabetic rats. J. Neuroinflammation *9*, 182. https://doi.org/10.1186/1742-2094-9-182.

50. Languren, G., Montiel, T., Julio-Amilpas, A., and Massieu, L. (2013). Neuronal damage and cognitive impairment associated with hypoglycemia: An integrated view. Neurochem. Int. *63*, 331–343. https://doi.org/10.1016/j.neuint.2013.06.018.

51. De Angelis, L.C., Brigati, G., Polleri, G., Malova, M., Parodi, A., Minghetti, D., Rossi, A., Massirio, P., Traggiai, C., Maghnie, M., and Ramenghi, L.A. (2021). Neonatal Hypoglycemia and Brain Vulnerability. Front. Endocrinol. *12*, 634305. https://doi.org/10.3389/fendo.2021.634305.

52. Verberne, A.J.M., Sabetghadam, A., and Korim, W.S. (2014). Neural pathways that control the glucose counterregulatory response. Front. Neurosci. *8*, 38. https://doi.org/10.3389/fnins.2014.00038.

53. Rao, R. (2015). Hypothalamic-Pituitary-Adrenal Axis Programming after Recurrent Hypoglycemia during Development. J. Clin. Med. *4*, 1729–1740. https://doi.org/10.3390/jcm4091729.

54. Belujon, P., and Grace, A.A. (2015). Regulation of dopamine system responsivity and its adaptive and pathological response to stress. Proc. Biol. Sci. *282*, 20142516. https://doi.org/10.1098/rspb.2014.2516.

55. Koekkoek, L.L., Mul, J.D., and la Fleur, S.E. (2017). Glucose-Sensing in the Reward System. Front. Neurosci. *11*, 716. https://doi.org/10.3389/fnins.2017.00716.

56. Seeman, M.V. (2021). History of the dopamine hypothesis of antipsychotic action. World J. Psychiatry *11*, 355–364. https://doi.org/10.5498/wjp.v11.i7.355.

57. Matschinsky, F.M., and Wilson, D.F. (2019). The Central Role of Glucokinase in Glucose Homeostasis: A Perspective 50 Years After Demonstrating the Presence of the Enzyme in Islets of Langerhans. Front. Physiol. *10*, 148. https://doi.org/10.3389/fphys.2019.00148.

58. Khan, M.S., Chandani, D.K., Siddiqui, E., Khalid, M., and Waafira, A. (2025). Olezarsen: FDA approval and clinical impact in familial chylomicronemia syndrome (FCS). Ann. Med. Surg. *87*, 7846–7850. https://doi.org/10.1097/ms9.0000000000003768.

59. Davies, N.M., Holmes, M.V., and Davey Smith, G. (2018). Reading Mendelian randomisation studies: a guide, glossary, and checklist for clinicians. BMJ *362*, k601. https://doi.org/10.1136/bmj.k601.

60. Burschinski, A., Schneider-Thoma, J., Chiocchia, V., Schestag, K., Wang, D., Siafis, S., Bighelli, I., Wu, H., Hansen, W.P., Priller, J., et al. (2023). Metabolic side effects in persons with schizophrenia during mid- to long-term treatment with antipsychotics: a network meta-analysis of randomized controlled trials. World Psychiatry *22*, 116–128. https://doi.org/10.1002/wps.21036.

61. Praharaj, S.K., Jana, A.K., Goyal, N., and Sinha, V.K. (2011). Metformin for olanzapine-induced weight gain: a systematic review and meta-analysis. Br. J. Clin. Pharmacol. *71*, 377–382. https://doi.org/10.1111/j.1365-2125.2010.03783.x.

62. Luo, C., Wang, X., Huang, H.X., Mao, X.Y., Zhou, H.H., and Liu, Z.Q. (2021). Coadministration of metformin prevents olanzapine-induced metabolic dysfunction and regulates the gut-liver axis in rats. Psychopharmacology (Berl) *238*, 239–248. https://doi.org/10.1007/s00213-020-05677-8.

63. Auton, A., Abecasis, G.R., Altshuler, D.M., Durbin, R.M., Abecasis, G.R., Bentley, D.R., Chakravarti, A., Clark, A.G., Donnelly, P., Eichler, E.E., et al. (2015). A global reference for human genetic variation. Nature *526*, 68–74. https://doi.org/10.1038/nature15393.

64. So, H.C., Chau, C.K.L., Chiu, W.T., Ho, K.S., Lo, C.P., Yim, S.H.Y., and Sham, P.C. (2017). Analysis of genome-wide association data highlights candidates for drug repositioning in psychiatry. Nat. Neurosci. *20*, 1342–1349. https://doi.org/10.1038/nn.4618.

65. Liu, J., Cheng, Y., Li, M., Zhang, Z., Li, T., and Luo, X.J. (2023). Genome-wide Mendelian randomization identifies actionable novel drug targets for psychiatric disorders. Neuropsychopharmacology *48*, 270–280. https://doi.org/10.1038/s41386-022-01456-5.

66. Cupido, A.J., Reeskamp, L.F., Hingorani, A.D., Finan, C., Asselbergs, F.W., Hovingh, G.K., and Schmidt, A.F. (2022). Joint Genetic Inhibition of PCSK9 and CETP and the Association With Coronary Artery Disease: A Factorial Mendelian Randomization Study. JAMA Cardiol. *7*, 955–964. https://doi.org/10.1001/jamacardio.2022.2333.

67. Wang, W., Li, R., Song, Z., Huang, N., Huang, T., Hua, X., and Tang, Y.D. (2025). Joint Associations of APOC3 and LDL-C-Lowering Variants With the Risk of Coronary Heart Disease. JAMA Cardiol. *10*, 463–472. https://doi.org/10.1001/jamacardio.2025.0195.

68. Rothman, K.J. (1990). No adjustments are needed for multiple comparisons. Epidemiology *1*, 43–46.

69. Hooper, R. (2025). To adjust, or not to adjust, for multiple comparisons. J. Clin. Epidemiol. *180*, 111688. https://doi.org/10.1016/j.jclinepi.2025.111688.

70. Kanoni, S., Graham, S.E., Wang, Y., Surakka, I., Ramdas, S., Zhu, X., Clarke, S.L., Bhatti, K.F., Vedantam, S., Winkler, T.W., et al. (2022). Implicating genes, pleiotropy, and sexual dimorphism at blood lipid loci through multi-ancestry meta-analysis. Genome Biol. *23*, 268. https://doi.org/10.1186/s13059-022-02837-1.

71. Chen, C.Y., Chen, T.T., Feng, Y.C.A., Yu, M., Lin, S.C., Longchamps, R.J., Wang, S.H., Hsu, Y.H., Yang, H.I., Kuo, P.H., et al. (2023). Analysis across Taiwan Biobank, Biobank Japan, and UK Biobank identifies hundreds of novel loci for 36 quantitative traits. Cell Genom. *3*, 100436. https://doi.org/10.1016/j.xgen.2023.100436.

72. Sakaue, S., Kanai, M., Tanigawa, Y., Karjalainen, J., Kurki, M., Koshiba, S., Narita, A., Konuma, T., Yamamoto, K., Akiyama, M., et al. (2021). A cross-population atlas of genetic associations for 220 human phenotypes. Nat. Genet. *53*, 1415–1424. https://doi.org/10.1038/s41588-021-00931-x.

73. Kanai, M., Ulirsch, J.C., Karjalainen, J., Kurki, M., Karczewski, K.J., Fauman, E., Wang, Q.S., Jacobs, H., Aguet, F., Ardlie, K.G., et al. (2021). Insights from complex trait fine-mapping across diverse populations. Preprint at medrxiv. https://doi.org/10.1101/2021.09.03.21262975.

74. Akiyama, M., Okada, Y., Kanai, M., Takahashi, A., Momozawa, Y., Ikeda, M., Iwata, N., Ikegawa, S., Hirata, M., Matsuda, K., et al. (2017). Genome-wide association study identifies 112 new loci for body mass index in the Japanese population. Nat. Genet. *49*, 1458–1467. https://doi.org/10.1038/ng.3951.

75. Richardson, T.G., Leyden, G.M., Wang, Q., Bell, J.A., Elsworth, B., Davey Smith, G., and Holmes, M.V. (2022). Characterising metabolomic signatures of lipid-modifying therapies through drug target mendelian randomisation. PLoS Biol. *20*, e3001547. https://doi.org/10.1371/journal.pbio.3001547.

76. Suzuki, K., Hatzikotoulas, K., Southam, L., Taylor, H.J., Yin, X., Lorenz, K.M., Mandla, R., Huerta-Chagoya, A., Melloni, G.E.M., Kanoni, S., et al. (2024). Genetic drivers of heterogeneity in type 2 diabetes pathophysiology. Nature *627*, 347–357. https://doi.org/10.1038/s41586-024-07019-6.

77. Xu, F., Yu, E.Y.W., Cai, X., Yue, L., Jing, L.P., Liang, X., Fu, Y., Miao, Z., Yang, M., Shuai, M., et al. (2023). Genome-wide genotype-serum proteome mapping provides insights into the cross-ancestry differences in cardiometabolic disease susceptibility. Nat. Commun. *14*, 896. https://doi.org/10.1038/s41467-023-36491-3.

78. Verma, A., Huffman, J.E., Rodriguez, A., Conery, M., Liu, M., Ho, Y.L., Kim, Y., Heise, D.A., Guare, L., Panickan, V.A., et al. (2024). Diversity and scale: Genetic architecture of 2068 traits in the VA Million Veteran Program. Science *385*, eadj1182. https://doi.org/10.1126/science.adj1182.

79. Consortium, T.G., Aguet, F., Anand, S., Ardlie, K.G., Gabriel, S., Getz, G.A., Graubert, A., Hadley, K., Handsaker, R.E., Huang, K.H., et al. (2020). The GTEx Consortium atlas of genetic regulatory effects across human tissues. Science *369*, 1318–1330. https://doi.org/10.1126/science.aaz1776.

80. Chen, J., Spracklen, C.N., Marenne, G., Varshney, A., Corbin, L.J., Luan, J., Willems, S.M., Wu, Y., Zhang, X., Horikoshi, M., et al. (2021). The trans-ancestral genomic architecture of glycemic traits. Nat. Genet. *53*, 840–860. https://doi.org/10.1038/s41588-021-00852-9.

81. Zhen, J., Gu, Y., Wang, P., Wang, W., Bian, S., Huang, S., Liang, H., Huang, M., Yu, Y., Chen, Q., et al. (2024). Genome-wide association and Mendelian randomisation analysis among 30,699 Chinese pregnant women identifies novel genetic and molecular risk factors for gestational diabetes and glycaemic traits. Diabetologia *67*, 703–713. https://doi.org/10.1007/s00125-023-06065-5.

82. Park, S., Kim, S., Kim, B., Kim, D.S., Kim, J., Ahn, Y., Kim, H., Song, M., Shim, I., Jung, S.H., et al. (2024). Multivariate genomic analysis of 5 million people elucidates the genetic architecture of shared components of the metabolic syndrome. Nat. Genet. *56*, 2380–2391. https://doi.org/10.1038/s41588-024-01933-1.

83. Cruchaga, C., Western, D., Timsina, J., Wang, L., Wang, C., Yang, C., Ali, M., Beric, A., Gorijala, P., Kohlfeld, P., et al. (2023). Proteogenomic analysis of human cerebrospinal fluid identifies neurologically relevant regulation and informs causal proteins for Alzheimer's disease. Preprint at Res. Sq. https://doi.org/10.21203/rs.3.rs-2814616/v1.

84. Yu, H., Yan, H., Wang, L., Li, J., Tan, L., Deng, W., Chen, Q., Yang, G., Zhang, F., Lu, T., et al. (2018). Five novel loci associated with antipsychotic treatment response in patients with schizophrenia: a genome-wide association study. Lancet Psychiatry *5*, 327–338. https://doi.org/10.1016/s2215-0366(18)30049-x.

85. Purcell, S., Neale, B., Todd-Brown, K., Thomas, L., Ferreira, M.A.R., Bender, D., Maller, J., Sklar, P., de Bakker, P.I.W., Daly, M.J., and Sham, P.C. (2007). PLINK: a tool set for whole-genome association and population-based linkage analyses. Am. J. Hum. Genet. *81*, 559–575. https://doi.org/10.1086/519795.

86. Howie, B.N., Donnelly, P., and Marchini, J. (2009). A flexible and accurate genotype imputation method for the next generation of genome-wide association studies. PLoS Genet. *5*, e1000529. https://doi.org/10.1371/journal.pgen.1000529.

87. Delaneau, O., Marchini, J., and Zagury, J.F. (2011). A linear complexity phasing method for thousands of genomes. Nat. Methods *9*, 179–181. https://doi.org/10.1038/nmeth.1785.

88. Yue, W.H., Wang, H.F., Sun, L.D., Tang, F.L., Liu, Z.H., Zhang, H.X., Li, W.Q., Zhang, Y.L., Zhang, Y., Ma, C.C., et al. (2011). Genome-wide association study identifies a susceptibility locus for schizophrenia in Han Chinese at 11p11.2. Nat. Genet. *43*, 1228–1231. https://doi.org/10.1038/ng.979.

89. Lu, Z., Zhang, Y., Yan, H., Su, Y., Guo, L., Liao, Y., Lu, T., Yu, H., Wang, L., Li, J., et al. (2022). ATAD3B and SKIL polymorphisms associated with antipsychotic-induced QTc interval change in patients with schizophrenia: a genome-wide association study. Transl. Psychiatry *12*, 56. https://doi.org/10.1038/s41398-022-01825-0.

90. Wishart, D.S., Feunang, Y.D., Guo, A.C., Lo, E.J., Marcu, A., Grant, J.R., Sajed, T., Johnson, D., Li, C., Sayeeda, Z., et al. (2018). DrugBank 5.0: a major update to the DrugBank database for 2018. Nucleic Acids Res. *46*, D1074–D1082. https://doi.org/10.1093/nar/gkx1037.

91. Palmer, T.M., Lawlor, D.A., Harbord, R.M., Sheehan, N.A., Tobias, J.H., Timpson, N.J., Davey Smith, G., and Sterne, J.A.C. (2012). Using multiple genetic variants as instrumental variables for modifiable risk factors. Stat. Methods Med. Res. *21*, 223–242. https://doi.org/10.1177/0962280210394459.

92. Burgess, S., and Thompson, S.G. (2021). Mendelian Randomization Methods for Causal Inference Using Genetic Variants, 2th Edition (CRC Press. Taylor & Francis Group).

93. Garfield, V., Salzmann, A., Burgess, S., and Chaturvedi, N. (2023). A Guide for Selection of Genetic Instruments in Mendelian Randomization Studies of Type 2 Diabetes and HbA1c: Toward an Integrated Approach. Diabetes *72*, 175–183. https://doi.org/10.2337/db22-0110.

94. Triozzi, J.L., Hsi, R.S., Wang, G., Akwo, E.A., Wheless, L., Chen, H.C., Tao, R., Ikizler, T.A., Robinson-Cohen, C., and Hung, A.M.; VA Million Veteran Program (2023). Mendelian Randomization Analysis of Genetic Proxies of Thiazide Diuretics and the Reduction of Kidney Stone Risk. JAMA Netw. Open *6*, e2343290. https://doi.org/10.1001/jamanetworkopen.2023.43290.

95. Obermeier, M., Mayr, A., Schennach-Wolff, R., Seemüller, F., Möller, H.J., and Riedel, M. (2010). Should the PANSS be rescaled? Schizophr. Bull. *36*, 455–460. https://doi.org/10.1093/schbul/sbp124.

96. Group, C.M.A.-S.C. (2020). Chinese Expert Consensus on the Management of Metabolic Syndrome in Patients with Schizophrenia (Chinese Journal of Psychiatry).

97. American Diabetes Association Professional Practice Committee (2025). Diagnosis and Classification of Diabetes: Standards of Care in Diabetes-2025. Diabetes Care *48*, S27–S49. https://doi.org/10.2337/dc25-S002.

98. Verbanck, M., Chen, C.Y., Neale, B., and Do, R. (2018). Detection of widespread horizontal pleiotropy in causal relationships inferred from Mendelian randomization between complex traits and diseases. Nat. Genet. *50*, 693–698. https://doi.org/10.1038/s41588-018-0099-7.

99. Burgess, S., Butterworth, A., and Thompson, S.G. (2013). Mendelian randomization analysis with multiple genetic variants using summarized data. Genet. Epidemiol. *37*, 658–665. https://doi.org/10.1002/gepi.21758.

100. Bowden, J., Davey Smith, G., Haycock, P.C., and Burgess, S. (2016). Consistent Estimation in Mendelian Randomization with Some Invalid Instruments Using a Weighted Median Estimator. Genet. Epidemiol. *40*, 304–314. https://doi.org/10.1002/gepi.21965.

CellPress
OPEN ACCESS

101. Bowden, J., Davey Smith, G., and Burgess, S. (2015). Mendelian randomization with invalid instruments: effect estimation and bias detection through Egger regression. Int. J. Epidemiol. *44*, 512–525. https://doi.org/10.1093/ije/dyv080.

102. Hemani, G., Zheng, J., Elsworth, B., Wade, K.H., Haberland, V., Baird, D., Laurin, C., Burgess, S., Bowden, J., Langdon, R., et al. (2018). The MR-Base platform supports systematic causal inference across the human phenome. eLife *7*, e34408–e34429. https://doi.org/10.7554/eLife.34408.

103. Giambartolomei, C., Vukcevic, D., Schadt, E.E., Franke, L., Hingorani, A.D., Wallace, C., and Plagnol, V. (2014). Bayesian test for colocalisation between pairs of genetic association studies using summary statistics. PLoS Genet. *10*, e1004383. https://doi.org/10.1371/journal.pgen.1004383.

104. Wallace, C. (2021). A more accurate method for colocalisation analysis allowing for multiple causal variants. PLoS Genet. *17*, e1009440. https://doi.org/10.1371/journal.pgen.1009440.

105. Breusch, T.S., and Pagan, A.R. (1979). A simple test for heteroscedasticity and random coefficient variation. Econometrica *47*, 1287–1294.

106. White, H. (1980). A heteroskedasticity-consistent covariance matrix estimator and a direct test for heteroskedasticity. Econometrica *48*, 817–838.

107. Cook, R.D. (1977). Detection of Influential Observation in Linear Regression. Technometrics *19*, 15–18. https://doi.org/10.1080/00401706.1977.10489493.

108. Snee, R.D. (1983). Regression Diagnostics: Identifying Influential Data and Sources of Collinearity (Taylor & Francis).

109. van Marken Lichtenbelt, W.D., Vanhommerig, J.W., Smulders, N.M., Drossaerts, J.M.A.F.L., Kemerink, G.J., Bouvy, N.D., Schrauwen, P., and Teule, G.J.J. (2009). Cold-activated brown adipose tissue in healthy men. N. Engl. J. Med. *360*, 1500–1508. https://doi.org/10.1056/NEJMoa0808718.

STAR★METHODS

KEY RESOURCES TABLE

REAGENT or RESOURCE	SOURCE	IDENTIFIER
Deposited data		
GWAS summary statistics of lipids	Global Lipids Genetics Consortium[70]	https://csg.sph.umich.edu/willer/public/glgc-lipids2021/results/ancestry_specific/
GWAS summary statistics of glucose	Taiwan Biobank[71]	http://ftp.ebi.ac.uk/pub/databases/gwas/summary_statistics/GCST90278001-GCST90279000/GCST90278628
GWAS summary statistics of Hemoglobin A1c levels	Taiwan Biobank[71]	http://ftp.ebi.ac.uk/pub/databases/gwas/summary_statistics/GCST90278001-GCST90279000/GCST90278632
GWAS summary statistics of systolic blood pressure	Biobank of Japan[72]	https://pheweb.jp/pheno/SBP
GWAS summary statistics of schizophrenia	Biobank of Japan[72]	https://pheweb.jp/pheno/Schizophrenia
GWAS summary statistics of coronary artery disease	Biobank of Japan[73]	https://pheweb.jp/pheno/CAD
GWAS summary statistics of body mass index	Biobank of Japan[74]	https://pheweb.jp/pheno/BMI
GWAS summary statistics of sphingomyelin levels	UK Biobank[75]	http://ftp.ebi.ac.uk/pub/databases/gwas/summary_statistics/GCST90092001-GCST90093000/GCST90092982
GWAS summary statistics of type 2 diabetes	Type 2 Diabetes Global Genetics Initative[76]	https://diagram-consortium.org/downloads.html
pQTL summary statistics in the blood	Guangzhou Nutrition and Health study[77]	https://omics.lab.westlake.edu.cn/data/proteins/phenotypes
GWAS summary statistics of hyperlipidemia	Million Veteran Program[78]	http://ftp.ebi.ac.uk/pub/databases/gwas/summary_statistics/GCST90475001-GCST90476000/GCST90475718
GWAS summary statistics of beta-endorphin levels	The Qatar Genome Program Research Consortium[35]	http://ftp.ebi.ac.uk/pub/databases/gwas/summary_statistics/GCST90161001-GCST90162000/GCST90161374
eQTL summary statistics	The Adult Genotype Tissue Expression Project v10[79]	https://gtexportal.org/home/aboutAdultGtex
GWAS summary statistics of fasting insulin	Chen et al.[80]	http://ftp.ebi.ac.uk/pub/databases/gwas/summary_statistics/GCST90002001-GCST90003000/GCST90002237
GWAS summary statistics of oral glucose tolerance test-2h	Zhen et al.[81]	http://ftp.ebi.ac.uk/pub/databases/gwas/summary_statistics/GCST90297001-GCST90298000/GCST90297790
GWAS summary statistics of metabolic syndrome	Park et al.[82]	http://ftp.ebi.ac.uk/pub/databases/gwas/summary_statistics/GCST90444001-GCST90445000/GCST90444489
pQTL summary statistics in the cerebrospinal fluid	Cruchaga et al.[83]	https://neurogenomics.wustl.edu/open-science/raw-data/
Chinese Antipsychotics Pharmacogenomics Consortium - Individual-level phenotype and genotype data	Yu et al.[84]	N/A
Chinese Antipsychotics Pharmacogenetics Consortium - Individual-level phenotype and genotype data	Yu et al.[84]	N/A

(*Continued on next page*)

Continued

REAGENT or RESOURCE	SOURCE	IDENTIFIER
Software and algorithms		
R 4.1.3	Open-source	https://cran.r-project.org/
PLINK v1.9	Purcell et al.[85]	https://www.cog-genomics.org/plink2/
IMPUTE v2	Bryan et al.[86]	https://mathgen.stats.ox.ac.uk/impute/impute_v2.html
SHAPEIT v2	Delaneau et al.[87]	https://mathgen.stats.ox.ac.uk/genetics_software/shapeit/shapeit.html

EXPERIMENTAL MODEL AND STUDY PARTICIPANT DETAILS

We applied two non-overlapping, clinically ascertained cohorts of individuals with SCZ. The discovery cohort was based on the CAPOC study, conducted in 2010, across five research centers in China, involving a total of 3030 participants.[84] The eligible patients provided their sociodemographic and clinical questionnaire information, physical measurements, and blood samples upon the baseline assessment. Individuals were assigned to six groups of antipsychotics (aripiprazole, olanzapine, quetiapine, risperidone, ziprasidone, or one of the first-generation antipsychotics [haloperidol or perphenazine]) for six weeks. An independent validation cohort was from the CAPEC study. Patients were enrolled from two study centers in China ($N = 568$) in 2009–2010 and received 8 weeks of treatment with olanzapine, aripiprazole, risperidone, quetiapine, clozapine, ziprasidone, or perphenazine.[84,88]

The CAPOC and CAPEC studies included Han Chinese participants, aged 18 to 45 years, diagnosed with SCZ based on the Diagnostic and Statistical Manual of Mental Disorders - Fourth Edition (DSM-IV) in the acute phase. Those who had severe, unstable physical diseases, such as hypertension, myocardial infarction, or other cardiac diseases; those who had QTc prolongation or a history of congenital QTc prolongation were excluded.

We further excluded participants without qualified genotype data (CAPOC: $n = 474$, CAPEC: $n = 265$), those with missing values or outliers (>3 SD, CAPOC: $n = 442$, CAPEC: $n = 11$), and those who reported using anti-cardiovascular disease drugs (CAPOC: $n = 3$, CAPEC: $n = 3$), leaving 2,111 and 292 participants for the discovery and validation cohorts, respectively (Figure S7). Among the participants from the CAPOC study, the median (IQR) age was 30.0 (24.0–38.0) years, and 1,048 (49.6%) were male. In the CAPEC cohort, the median (IQR) age was 32.0 (23.0–40.0) years, with males accounting for 45.2% of participants. A summary of the multi-omics dataset applied for the present study was shown in Table S54.

The CAPOC study was approved by the ethics committee of the Peking University Sixth Hospital and each participating site. The reference number was 2009-LUNSHEN-23, and the approval date was April 21st, 2009. This study was registered at the Chinese Clinical Trial Registry (ChiCTR-TRC-10000934). The CAPEC study was approved by the ethics committee of each participating site, registered at the Chinese Clinical Trial Registry (ChiCTR-RNC-09000522). For both studies, informed written consent was obtained from all included participants in accordance with the Declaration of Helsinki. All participants were asked to appoint a family member or close friend involved in the informed consent discussion and help the patients with decision-making.

For the publicly available GWAS, expression, and protein quantitative trait loci summary statistics, all contributing studies were conducted in accordance with the ethical standards of the relevant national and institutional committees on human experimentation and the Helsinki Declaration. All study protocols were approved by the institutional ethics review boards at each site, and written or verbal informed consent was obtained from all participants.

METHOD DETAILS

Genotyping and quality control

A total of 900,015 SNPs in the CAPOC study and 487,774 SNPs in the CAPEC study were genotyped using the Illumina Human Omni ZhongHua8 Beadchips (Illumina, San Diego, CA, USA) designed for the Chinese. Genotype quality control was conducted using the following exclusion criteria: (1) duplicated SNPs, (2) call rate <0.05, (3) minor allele frequency (MAF) < 0.05, (4) Hardy-Weinberg P (P_{HW}) < 10^{-6}. The imputation was performed using IMPUTE (Version 2), taking the 1000 Genomes Project-East Asian population (1000G-EAS)[63] as the reference. A total of 11,986,772 SNPs from the CAPOC study and 10,732,149 SNPs from the CAPEC study passed the imputation quality threshold (info score >0.6).[84,89]

Among the 3030 participants of CAPOC study, 474 participants without qualified genotype data were excluded, and 11 out of 303 participants in the CAPEC study were also excluded following the exclusive criteria: (1) call rate <0.05, (2) sex discrepancy, (3) relatedness (proportion identical-by-descent <0.2); (4) heterozygosity (> three standard deviations); (5) principal components outliers. Qualified samples were phased using SHAPEIT (Version 2).

The present study additionally performed the genomic quality control for the imputed SNPs. The SNPs that met any of the criteria were excluded: (1) call rate <0.02, (2) MAF <0.01, (3) $P_{HW} < 10^{-6}$. For the CAPOC study, a total of 4,995,111 SNPs and 2,111 participants were included in the analysis; whereas 2,508,713 SNPs and 292 participants from the CAPEC study were included for the present analysis (Figure S7).

Drug-target GRS

Referring to the World Health Organization Collaborating Center for Drug Statistics Methodology and DrugBank platform,[90] we selected 14 genes encoding pharmacological targets of lipid-lowering drugs and three targets of antidiabetic drugs (Table S55). Drug-target GRSs were constructed by extracting significant genetic associations ($p < 5 \times 10^{-8}$) with lipids and glucose concentrations from the Global Lipids Genetics Consortium (GLGC, $N = 146{,}492$, East Asian)[70] and Taiwan Biobank ($N = 92{,}615$, East Asian).[71] We restricted the independent ($r^2 < 0.1$) common variants (MAF >0.005) within the 100 kb region around the target genes (Table S2). The weighted drug-target GRSs were constructed by summing the number of effect alleles weighted by the effect size at each SNP. The effect sizes were obtained from corresponding GWASs (GLGC for lipid and Taiwan Biobank for glucose) and harmonized to ensure that all SNP effects reflected decreases in the relevant lipids or glucose.[91,92]

In addition, we calculated traditional (genome-wide) GRSs for lipids and glucose. The significant, independent, common genetic associations ($p < 5 \times 10^{-8}$, $r^2 < 0.001$, MAF >0.005) with lipids and glucose were extracted from GLGC and Taiwan Biobank (Tables S3, S4, and S5). The GRSs were calculated by combining all variants for each target, weighed by effect sizes corresponding to higher lipid or glucose levels.[91,92]

To evaluate the strength of these GRSs, we calculated the proportion of variance explained (R^2) by the selected SNPs using the formula: $R^2 = \Sigma\beta^2 \times 2 \times \text{MAF} \times (1 - \text{MAF})$, where β denotes the effect size. We further assessed instrument strength using the F-statistic, computed as $F = [(N - K - 1)/K] \times R^2/(1 - R^2)$, where N was the sample size and K was the number of SNPs included in the GRS.[67,91,92] When allele-frequency information was unavailable, we calculated the F value using the formula: $F = (\beta/\text{standard error})$.[92–94]

Outcomes

Fasting blood biochemistry was measured upon the baseline survey, including glucose and lipid (LDLC, HDLC, TG, and TC, mg/dL). Patients were assessed by trained psychiatrists both at the baseline visit and during a subsequent follow-up (CAPOC at week six; CAPEC at week eight). The severity of psychiatric symptoms was quantified using the PANSS, capturing a comprehensive total score and detailed subscores reflecting positive, negative, and general psychopathology domains. The percentage reduction in the total PANSS was applied as the primary outcome to assess the efficacy of antipsychotic treatments, and was calculated according to the formula below.[95] Given that treatment efficacy varies across different symptom domains, the percentage reduction in the three subscales in PANSS served as the secondary outcomes. We conducted a sensitivity analysis using a binary classification of better antipsychotic efficacy, defined as achieving a PANSS percentage reduction greater than the median value.

$$\textit{PANSS percentage reduction} = (\textit{PANSS endpoint score} - \textit{PANSS baseline score}) / (\textit{PANSS baseline score} - 30) \times 100$$

* When analyzing subscale scores, baseline adjustment of 30 was not applied.

Covariates

The present drug-target genetic association analysis was conditioned on the following covariates: gender, age, age,[2] study centers, the top five genetic principal components (PCs), course of SCZ, and previous medication. The assigned type of antipsychotic drug during follow-up was further adjusted when antipsychotic efficacy served as the outcome.

QUANTIFICATION AND STATISTICAL ANALYSIS

Single drug-target analysis

A one-sample drug-target genetic association analysis was applied to evaluate the causal effects of drug targets on the outcomes. The characteristics were described by the median-dichotomized drug-target GRSs, which predicted relatively lower and higher concentrations of lipid and glucose. Associations between drug-target GRSs and baseline characteristics were tested, in which the linear trends were assessed by treating GRSs as continuous variables.

We investigated the associations between drug-target GRSs and measured lipid or glucose levels using linear regression. The drug-target GRSs significantly associated with lipid or glucose traits - representing genetically proxied lipid-lowering or glucose-lowering effects - were subsequently regressed on the PANSS percentage reduction.

In addition, we assessed the associations between levels of glucose, lipid, and the antipsychotic efficacy using both conventional prospective analysis and one-sample MR analysis. The latter applied a two-stage linear regression for causal estimation: glucose or lipid was regressed on the corresponding traditional GRS. The PANSS percentage reduction was regressed with the traditional GRS-predicted glucose or lipids.

Stratified analysis and independent validation

Stratified analysis

To further examine the effects of identified drug targets on antipsychotic efficacy among participants under specific baseline metabolic conditions, classes of antipsychotics, and specific clinical characteristics, we performed stratified analyses. These analyses were conducted according to: (1) hyperlipemia (baseline TG $\geq$ 151 mg/dL [1.70 mmol/L] or baseline HDL $\leq$ 40mg/dL [1.04 mmol/L]),[96] prediabetes or diabetes status (baseline glucose $\geq$ 100.8 mg/dL [5.6 mmol/L])[97]; (2) antipsychotics with stronger (risperidone, olanzapine, quetiapine, or clozapine) or milder (aripiprazole, ziprasidone, perphenazine, or haloperidol) metabolic side effects; (3) clinical stage of first-episode, drug naive (individuals with <2 years of first-episode SCZ course, and <14 days of antipsychotic exposure) or recurrent patients; (4) gender. We additionally tested for non-additive interaction by including an interaction term between the drug-target GRS and the stratifying variable.

Independent validation

To validate the identified associations between drug targets and antipsychotic efficacy, we applied the CAPEC study, a cohort of Han Chinese participants diagnosed with SCZ disorder independent from the CAPOC, as the replication dataset.

Two-sample Mendelian randomization analysis

We integrated the non-overlapping GWASs of the metabolic indicators (GWAS of lipid from the GLGC, GWAS of glucose from the Taiwan Biobank) and PANSS percentage change (CAPOC study) among East Asians to replicate the causal effects of each drug target using two-sample MR analysis.

Genetic instruments for each drug target were selected using the same criteria applied in our one-sample MR analysis. Mendelian randomization pleiotropy residual sum and outlier (MR-PRESSO)[98] was applied. Outlier SNPs (global test $p < 0.05$) were excluded to minimize pleiotropy bias. For the causal estimation, inverse-variance-weighted (IVW)[99] was applied as the primary analysis, along with the weighted median (WM)[100] method as a sensitivity analysis. The IVW method provided an unbiased causal estimate if there was no horizontal pleiotropy. WM gave valid tests even if the prevalence of invalid SNPs was up to 50%.[100] We assessed directional horizontal pleiotropy using the intercept test from MR-Egger (ME) regression.[101] Cochrane's *Q* test was performed to assess the heterogeneity among SNPs, and a random-effect model in IVW was applied if heterogeneity existed ($p < 0.05$). Two-sample MR Analyses were carried out using R packages "TwoSampleMR" (version 0.5.6),[102] "MR-PRESSO" (version 1.0)[98] in the R environment (version 4.1.3).

Multi-omics expressions

To deepen our insight into the identified associations between lipid- and glucose-modulating targets and antipsychotic efficacy, we performed a broad downstream investigation combining multi-omics datasets across the cardiometabolic and cerebral systems.

Protein-level drug-target GRS analysis

We constructed the drug-target GRSs by applying pQTL data in the blood[77] and CSF.[83] We extracted the significant, genetic associations ($p < 0.005$) with protein concentrations in the blood from the Guangzhou Nutrition and Health study (GNHS, 2410 East Asians),[77] and in the CSF based on 3,107 Europeans,[83] separately. The SNPs were further restricted to independent ($r^2 < 0.1$) common variants (MAF >0.005) within the 100 kb region around the target genes (Tables S6 and S7). To mimic the pharmacological effects of glucose- and lipid-lowering drugs on their target proteins, we adjusted the pQTL-GRS according to the drug–target interaction type. For targets inhibited by their corresponding drugs, the initial pQTL-GRS was multiplied by (−1) to simulate the suppressed effect; for targets stimulated by agonists, we retained the original pQTL-GRS coefficients[66,67] (Figure S3).

Associations between the pQTL-GRSs and measured lipid, glucose, and antipsychotic efficacy were examined using linear or logistic regression models. These pQTL-GRSs enabled validation of whether these targets influence the SCZ treatment efficacy through the protein level changes in the peripheral and central compartments.

Mediation analysis

A mediation analysis was performed to estimate whether the effects of drug-target pQTL-GRSs were mediated by the lowering of lipids or glucose. We conditioned the pQTL-GRS on the GRS of lipids (for lipid-lowering targets) and the GRS of glucose (for glucose-lowering targets). The same genetic variants as those in pQTL-GRS were used, but weighted by their effects on the measured lipid and glucose derived from independent GWAS summary statistics.[70,71]

Colocalization analysis

To strengthen the on-target causal effect, we performed a Bayesian colocalization analysis for each identified target, incorporating the pQTL dataset, GWASs of lipids and glucose, and GWAS of PANSS percentage reduction.[103] The colocalization analysis tested the posterior probability of five hypotheses: H_0: SNP was not associated with either trait. H_1: SNP was only associated with the first trait. H_2: SNP was only associated with the second trait; H_3: Two independent SNPs were separately associated with the two traits; H_4: A shared SNP was associated with both traits. The LD-pruning was performed before the Bayesian test using PLINK 1.9 (window size = 500kb, $r^2 = 0.8$). The posterior probability of H4 (PP.H4) larger than 0.6 was applied to identify causal SNPs, and the SNP with the highest PP.H4 was reported as the lead shared variant within each locus.[103,104] The colocalization analysis was performed using the R package "coloc" (version 5.2.3).[103]

Tissue expression and metabolic/brain associations

By leveraging the expression levels of the identified drug-target genes from the Adult Genotype Tissue Expression Project (GTEx v10),[79] we illustrated the expression profiles for each target across different tissues. We further explored drug-target genetic associations with the laboratory and physiological indicators related to the glucose-lipid pathway and brain domain, along with the related

disorders, based on GWAS summary statistics and the CAPOC study. Using individual-level data from CAPOC, we explored the associations between the identified lipid-lowering, antidiabetic drug-target GRSs with other laboratory and physical measurements, including blood pressure, pulse, QTc interval, liver enzymes, urea nitrogen, creatinine, and prolactin. Linear regression was applied.

Factorial Mendelian randomization

The GRSs that showed significant associations with antipsychotic efficacy in the single drug-target genetic association analysis were carried forward for the factorial MR analysis. Participants were allocated into the 2 × 2 factorial groups based on the median-dichotomized GRSs of each lipid-glucose drug-target pair: (1) genetically predicted higher levels of both lipid and glucose (reference group); (2) genetically predicted lower lipid but higher glucose; (3) genetically predicted higher lipid but lower glucose; (4) genetically predicted lower levels of both lipid and glucose, analogous to a factorial RCT involving two drug classes. Joint associations between the two types of targets on the reduction rates of PANSS were evaluated using linear regression. Non-additive interaction was assessed by including the product term of continuous GRSs of these two targets.

Model diagnostics

For all linear regression models, we assessed for heteroskedasticity using the Breusch–Pagan test[105] and the White test.[106] We additionally calculated heteroskedasticity-consistent (HC3)-robust standard errors via a sandwich variance–covariance estimator, reporting both conventional and HC3-based 95% CIs and p values. For logistic regression models, we focused on HC3-robust standard errors for the log-odds. In both linear and logistic models, we examined influential points using Cook’s distance (>0.5) and studentized deleted residuals (>4),[107–109] refitted models after excluding flagged observations to assess the sensitivity of the estimates. We classified each effect as “stable” if (i) the direction of association was unchanged between the primary and sensitivity models, (ii) the 95% CIs overlapped, and (iii) statistical significance at $\alpha = 0.05$ was concordant across models; otherwise, the effect was labeled “sensitive”. Variance inflation factors (VIFs) were calculated for all covariates to evaluate multicollinearity, and VIF values >10 were considered indicative of potentially problematic multicollinearity.

All analyses were performed in the R environment (version 4.1.3).[102] Considering the multiple tests of drug targets and outcomes, significance was defined as $P_{FDR} < 0.05$, and nominal $p < 0.05$ was considered suggestive. In the factorial MR analysis, we applied the Bonferroni correction for the total PANSS and three subscales, setting the significance threshold at $p < 0.05/4 = 0.0125$. Data were analyzed from August 2024 to December 2025.

Cell Reports Medicine, Volume *7*

Supplemental information

Lipid-lowering and glucose-lowering drug targets differentially modulate antipsychotic treatment efficacy in schizophrenia

Yunqing Zhu, Rui Yuan, Zhe Lu, Yuyanan Zhang, Zhewei Kang, Xiaoyang Feng, Guorui Zhao, Junyuan Sun, Jing Guo, Tong Yu, Yang Yang, Yaoyao Sun, and Weihua Yue

Supplemental Figures

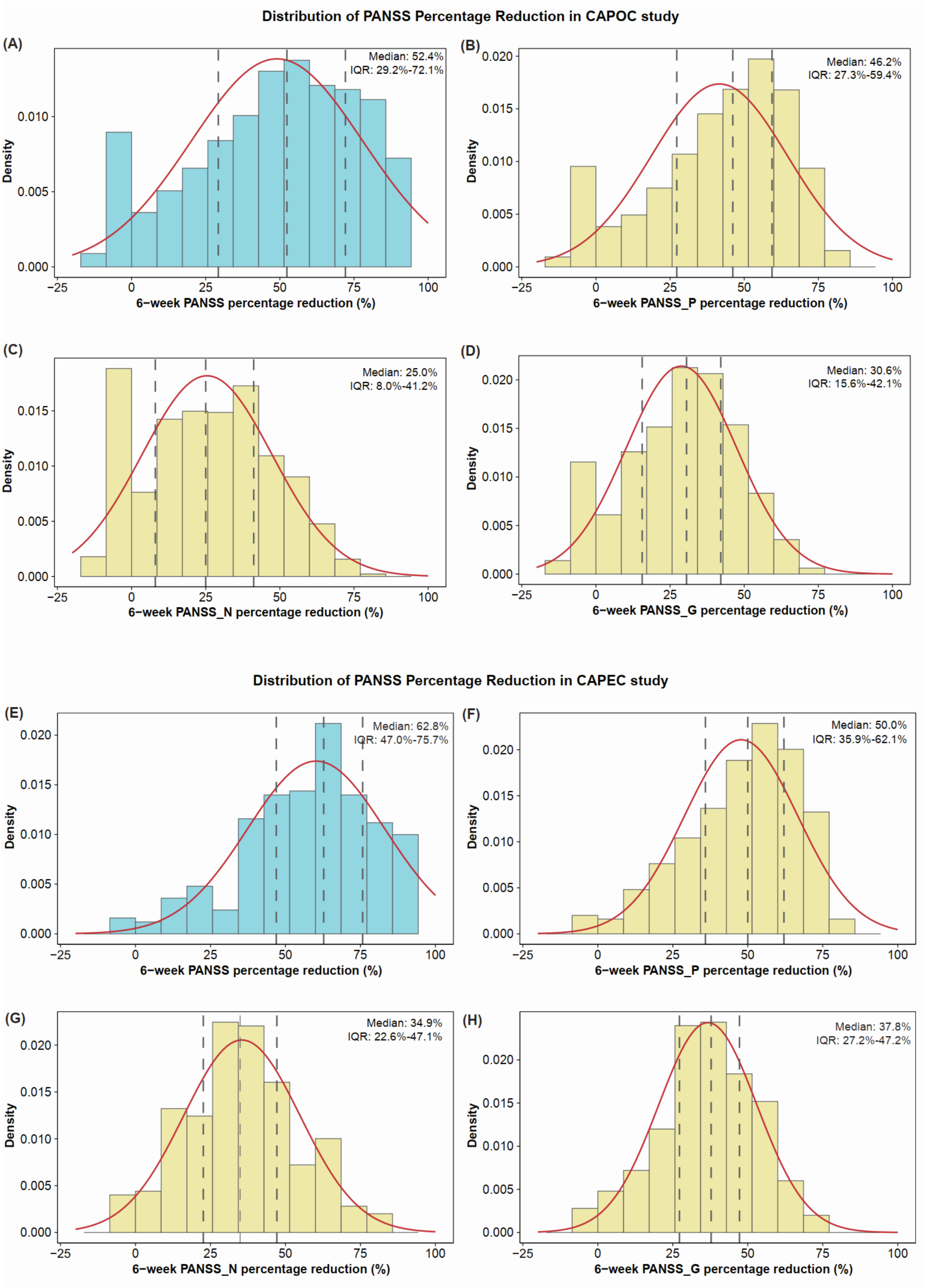

Figure S1. Distribution of PANSS percentage reduction. Related to Table 1.

Panel A-D exhibited the six-week percentage reduction of Positive and Negative Syndrome Scale (PANSS) total score, positive (P), negative (N), and general psychopathology (G) subscales in the Chinese Antipsychotics Pharmacogenomics Consortium (CAPOC) study. **Panel E-H** exhibited the eight-week percentage reduction of PANSS total score, PANSS_P, PANSS_N, and PANSS_G

subscales in the Chinese Antipsychotics Pharmacogenetics Consortium (CAPEC) study. The median and interquartile range (IQR) values were displayed.

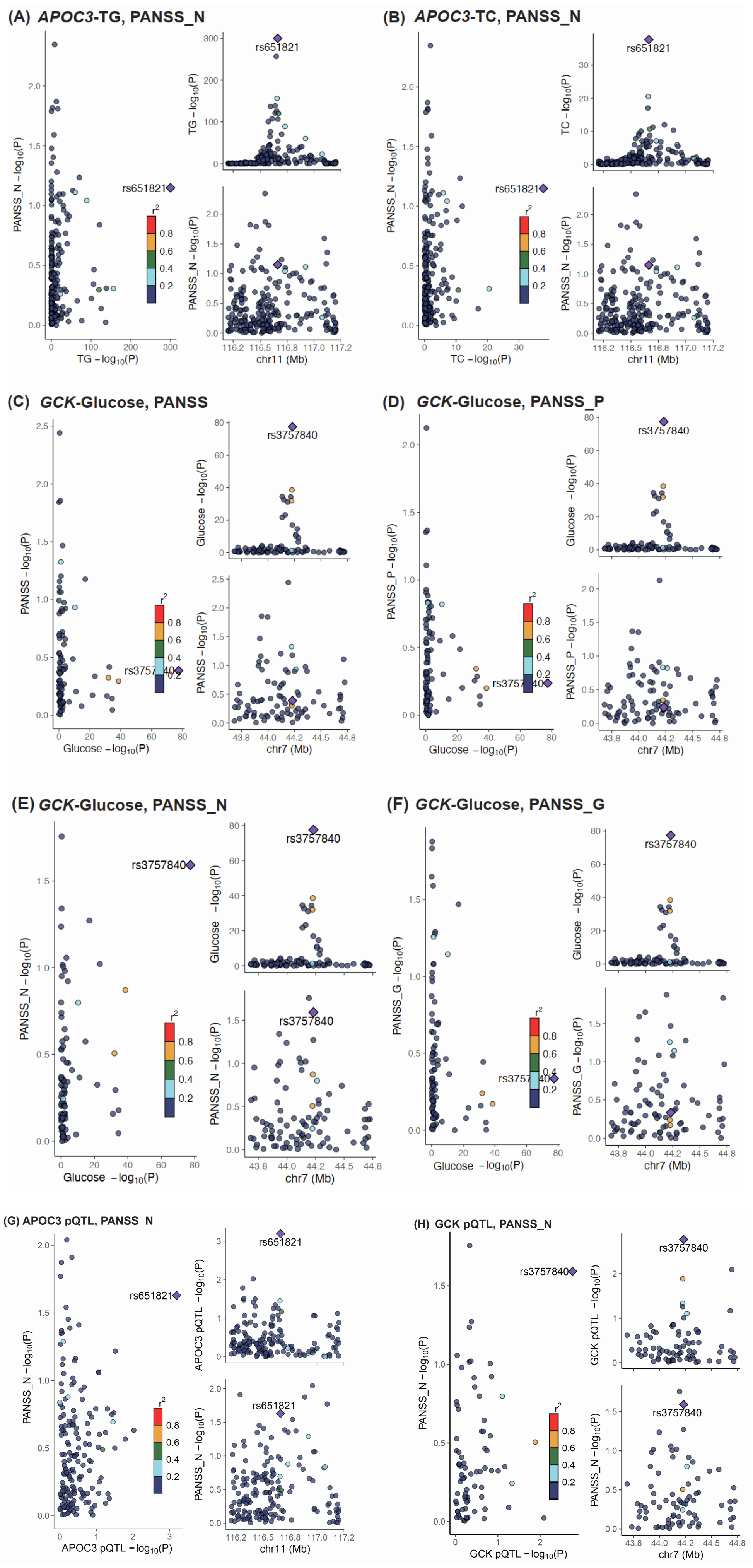

(A) APOC3-TG, PANSS_N
PANSS_N −log10(P)
TG −log10(P)
TG −log10(P)
PANSS_N −log10(P)
chr11 (Mb)
rs651821
r2
0.8
0.6
0.4
0.2
(B) APOC3-TC, PANSS_N
PANSS_N −log10(P)
TC −log10(P)
TC −log10(P)
PANSS_N −log10(P)
chr11 (Mb)
rs651821
(C) GCK-Glucose, PANSS
PANSS −log10(P)
Glucose −log10(P)
Glucose −log10(P)
PANSS −log10(P)
chr7 (Mb)
rs3757840
(D) GCK-Glucose, PANSS_P
PANSS_P −log10(P)
Glucose −log10(P)
Glucose −log10(P)
PANSS_P −log10(P)
chr7 (Mb)
rs3757840
(E) GCK-Glucose, PANSS_N
PANSS_N −log10(P)
Glucose −log10(P)
Glucose −log10(P)
PANSS_N −log10(P)
chr7 (Mb)
rs3757840
(F) GCK-Glucose, PANSS_G
PANSS_G −log10(P)
Glucose −log10(P)
Glucose −log10(P)
PANSS_G −log10(P)
chr7 (Mb)
rs3757840
(G) APOC3 pQTL, PANSS_N
PANSS_N −log10(P)
APOC3 pQTL −log10(P)
APOC3 pQTL −log10(P)
PANSS_N −log10(P)
chr11 (Mb)
rs651821
(H) GCK pQTL, PANSS_N
PANSS_N −log10(P)
GCK pQTL −log10(P)
GCK pQTL −log10(P)
PANSS_N −log10(P)
chr7 (Mb)
rs3757840

Figure S2. Colocalization analysis between GWAS of glucose and lipids, *APOC3* and *GCK* pQTL data, GWAS of PANSS percentage reduction. Related to Figure 3.

Each panel showed a colocalized locus (PP.H4>0.6) identified for the corresponding trait pair. The associations for each trait were shown on the right, and the combined association for pairwise traits was shown on the left. The candidate-shared causal variant identified by pairwise colocalization analysis was shown in the purple diamond. The color of each variant represented its linkage distribution (LD) relationship with the candidate-shared causal variant. All genomic location is based on the reference genome hg19, and LD calculation was based on 1000 Genomes in Phase III of the East Asian population.

(A) *APOC3* locus for TG-PANSS_N percentage reduction; (B) *APOC3* locus for TC-PANSS_N percentage reduction; (C) *GCK* locus for glucose-PANSS percentage reduction; (D) *GCK* locus for glucose-PANSS_P percentage reduction; (E) *GCK* locus for glucose-PANSS_N percentage reduction; (F) *GCK* locus for glucose-PANSS_G percentage reduction; (G) *APOC3* locus for APOC3 protein in blood – better antipsychotic efficacy of PANSS_N; (H) *GCK* locus for GCK protein in CSF- PANSS_N percentage reduction.

Abbreviations: *APOC3*, apolipoprotein C3; *GCK*, glucokinase; TG, triglyceride; TC, total cholesterol; PANSS, Positive and Negative Syndrome Scale; N, Negative scale; G, General psychopathology scale; P, Positive scale; pQTL, protein quantitative trait loci - genetic risk score; CSF, cerebrospinal fluid.

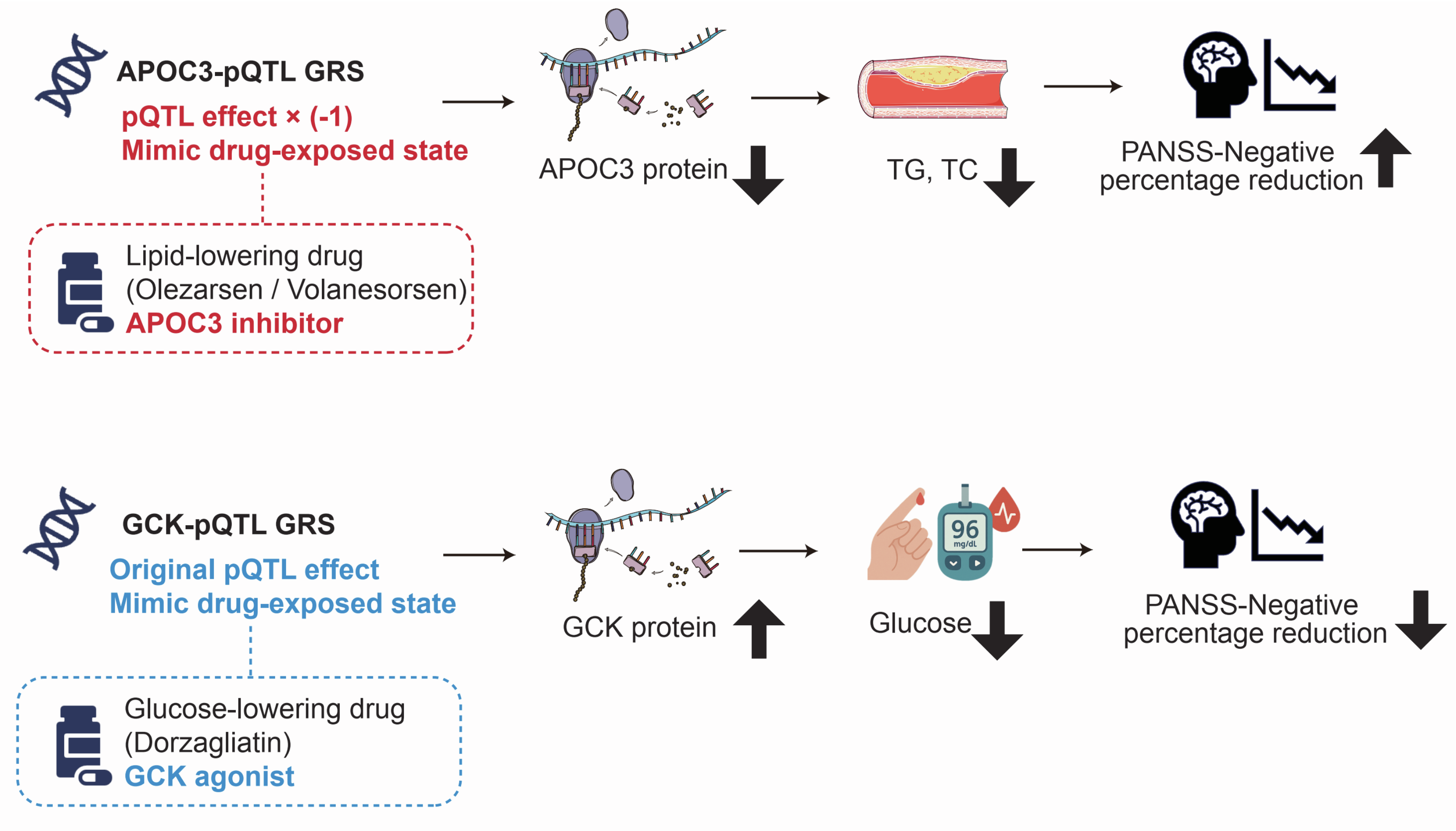

Figure S3. Construction of *APOC3, GCK* pQTL GRSs and their associations with lipid, glucose, and PANSS-negative percentage reduction. Related to Figure 3.

Notes: We calculated the drug-target pQTL-GRSs following published drug-target genetic association studies (PMID: 40105833, 35921096)

Abbreviations: *APOC3*, apolipoprotein C3; *GCK*, glucokinase; GRS, genetic risk score; TG, triglyceride; TC, total cholesterol; PANSS, Positive and Negative Syndrome Scale; N, Negative scale; protein quantitative trait loci (pQTL).

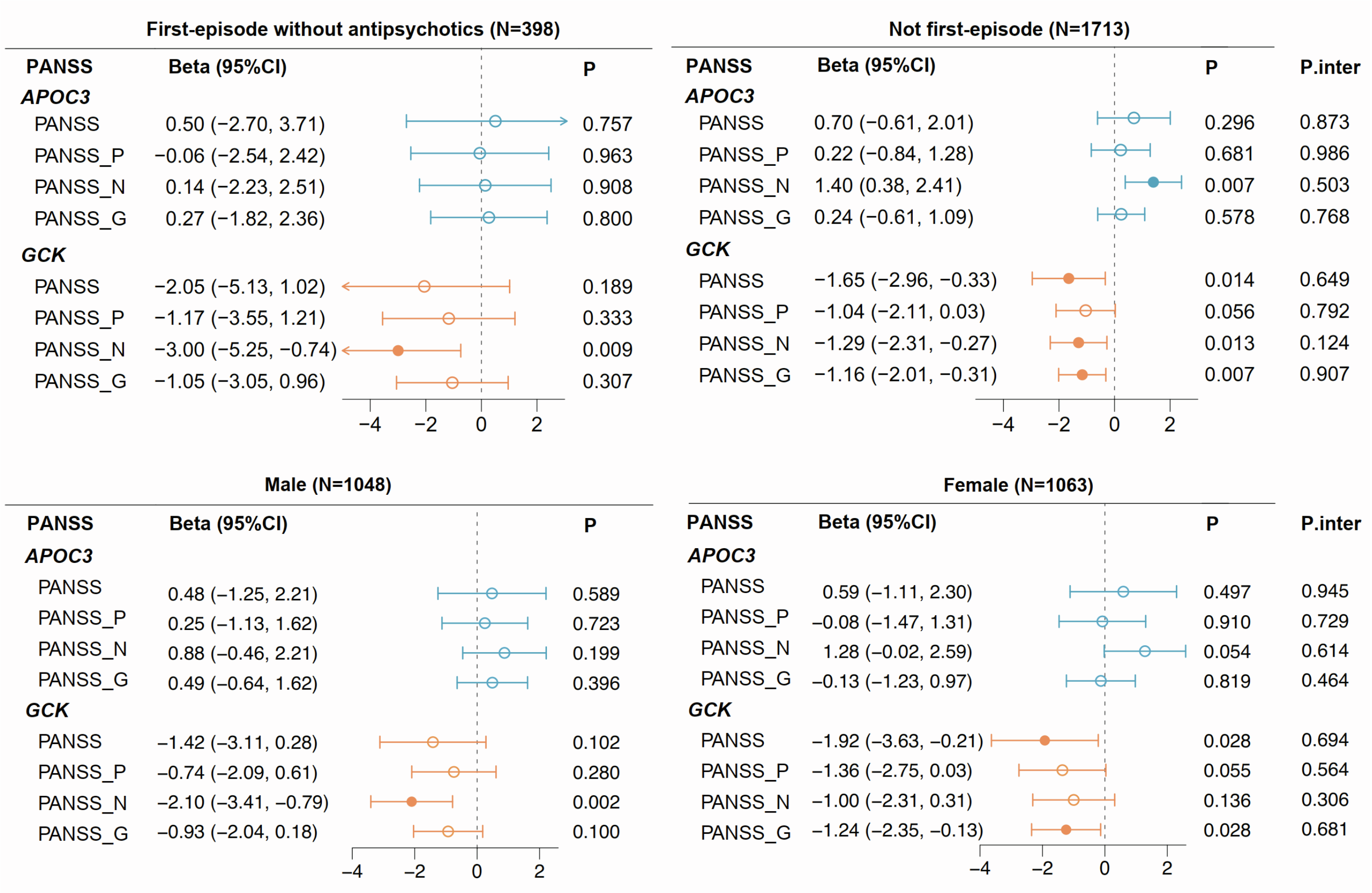

Figure S4. Stratified analysis by the schizophrenia course and gender. Related to Figure 4.

Notes: The beta (95%CIs) represented the average percentage reduction in the PANSS for per standard deviation increase in the drug-target GRS.

The first episode without antipsychotics was defined as individuals with < 2 years of first-episode schizophrenia course, and < 14 days of antipsychotic exposure (N=398), others were classified as the non-first-episode patients (N=1713).

Abbreviations: *APOC3*, apolipoprotein C3; *GCK*, glucokinase; TG, triglyceride; PANSS, Positive and Negative Syndrome Scale; N, Negative scale; G, General psychopathology scale; P, Positive scale; GRS, genetic risk score.

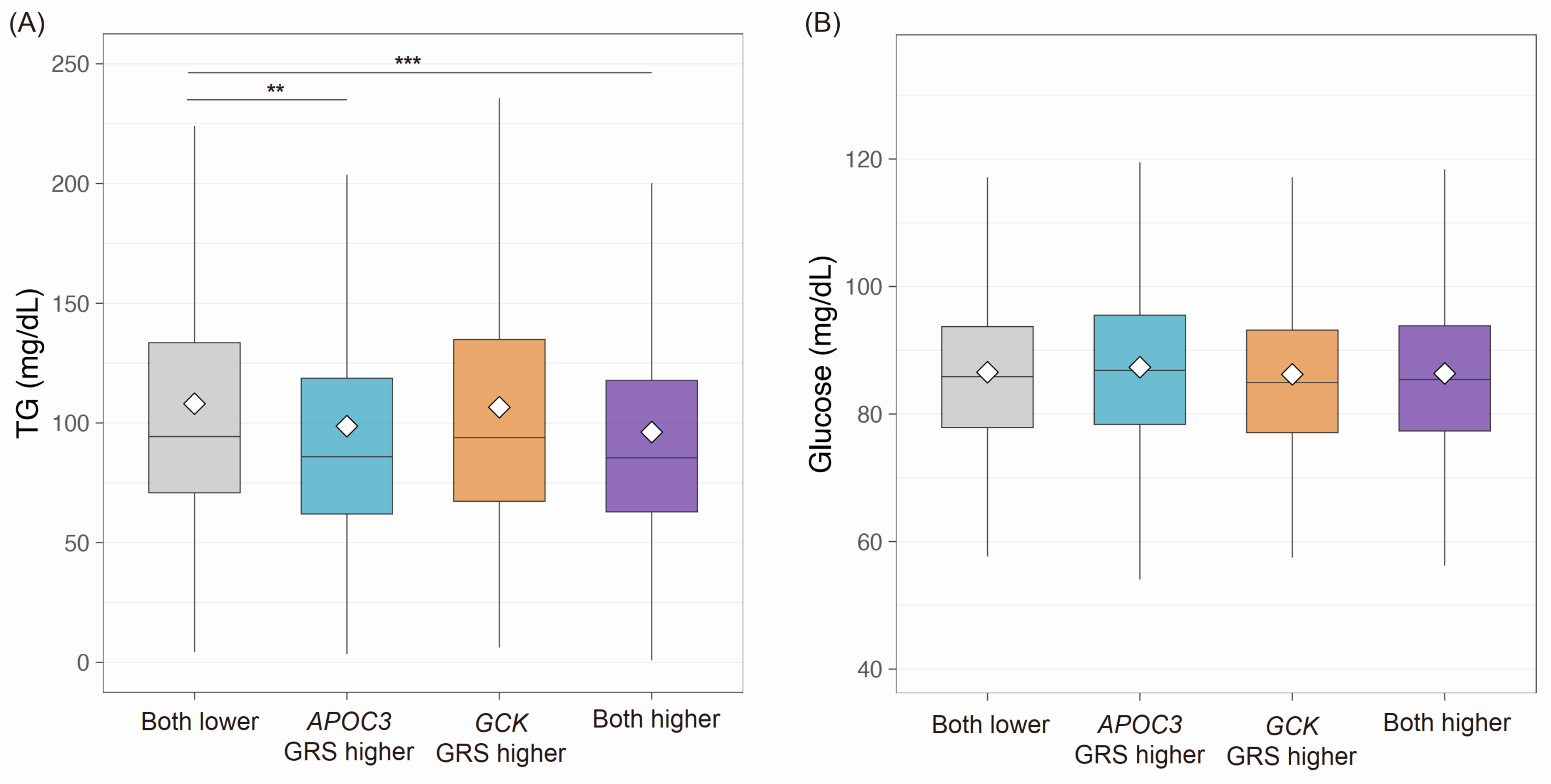

Figure S5. Triglyceride and glucose by 2*2 factorial MR study of *APOC3* and *GCK* GRSs in the CAPOC study. Related to Figure 1.

Notes: Full results could be found in Tables S48-49.* P<0.05, ** P<0.01, *** P<0.001.

Abbreviations: *APOC3*, apolipoprotein C3; *GCK*, glucokinase; TG, triglyceride; GRS, genetic risk score; CAPOC, Chinese Antipsychotics Pharmacogenomics Consortium; MR, mendelian randomization.

PANSS	Group	PANSS percentage reduction	Beta (95%CI)	P.Interaction
PANSS	Both lower	50.0 (31.2, 71.7)	Reference	0.704
	APOC3 GRS higher	51.0 (33.0, 75.0)	0.57 (−2.85, 3.99)	
	***GCK* GRS higher**	**47.4 (25.0, 72.0)**	**−3.46 (−6.89, −0.04)**	
	Both higher	47.7 (27.8, 68.4)	−2.88 (−6.39, 0.63)	
PANSS_P	Both lower	42.3 (27.8, 60.0)	Reference	0.916
	APOC3 GRS higher	43.1 (30.0, 60.9)	0.61 (−2.14, 3.35)	
	GCK GRS higher	40.8 (24.8, 59.3)	−1.95 (−4.70, 0.80)	
	Both higher	40.4 (26.9, 57.7)	−2.17 (−4.98, 0.65)	
PANSS_N	Both lower	26.3 (10.0, 42.7)	Reference	0.525
	APOC3 GRS higher	27.3 (9.1, 42.3)	0.87 (−1.76, 3.50)	
	***GCK* GRS higher**	**23.7 (7.1, 39.5)**	**−3.08 (−5.71, −0.44)**	
	Both higher	24.6 (5.8, 40.7)	−2.04 (−4.74, 0.65)	
PANSS_G	Both lower	29.6 (18.4, 42.0)	Reference	0.962
	APOC3 GRS higher	29.9 (16.4, 44.2)	0.00 (−2.22, 2.22)	
	GCK GRS higher	28.0 (14.2, 41.2)	−2.20 (−4.43, 0.02)	
	Both higher	27.9 (14.8, 41.1)	−2.15 (−4.42, 0.13)	

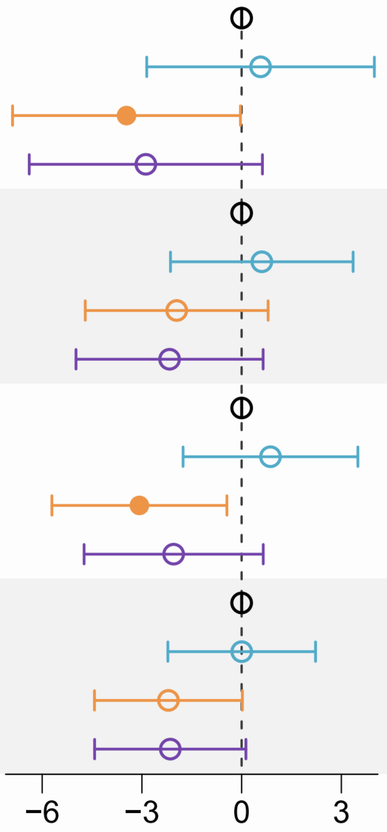

Figure S6. Combined and interactive effects of *APOC3* and *GCK* on the percentage reduction of PANSS. Related to Figure 1.

Notes: The beta (95%CIs) represented the average percentage reduction in the PANSS of the group with higher *APOC3* GRS, higher *GCK* GRS, and both higher GRSs (joint association), versus the reference group – those with both lower GRSs. The statistical significance of interactions was tested through a product term of the continuous *APOC3, GCK* GRSs. Full results could be found in Tables S50-53.

Abbreviations: *APOC3*, apolipoprotein C3; *GCK*, glucokinase; TG, triglyceride; PANSS, Positive and Negative Syndrome Scale; N, Negative scale; G, General psychopathology scale; P, Positive scale; GRS, genetic risk score.

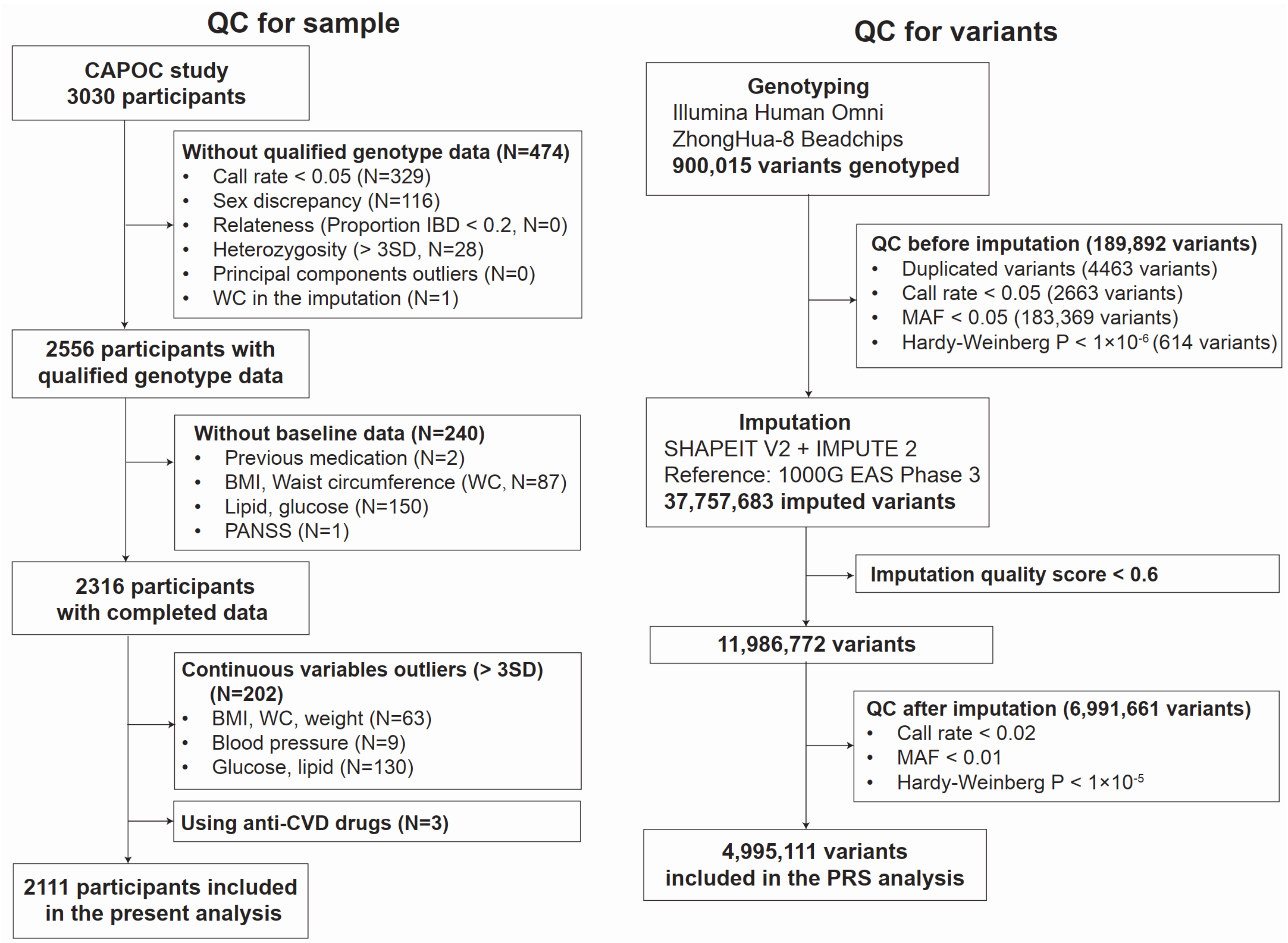

Figure S7. Quality control for participants and variants in the CAPOC study. Related to Table 1.

Notes: Abbreviations: QC, Quality control; CAPOC, Chinese Antipsychotics Pharmacogenomics Consortium; IBD, Identical by Descent; SD, standard deviation; WC, waist circumference; BMI, body mass index; CVD, cardiovascular disease; PANSS, Positive and Negative Syndrome Scale; MAF, minor allele frequency; EAS, East Asian; 1000G EAS Phase 3, 1000 Genomes Project Phase 3 East Asian (EAS) population; PRS, Polygenic Risk Score.

Supplemental Tables

Table S3. Instrument variables applied for TG GRS. Related to Figure 3.

SNP	CHR	BP	P	EA	OA	Beta	SE	EAF
rs1077835	15	58723426	5.01E-36	G	A	0.070	0.005	0.413
rs116111528	6	32313512	2.52E-11	T	C	0.087	0.012	0.0457
rs1260326	2	27730940	1.80E-75	C	T	-0.098	0.005	0.48
rs13246993	7	73022746	3.62E-54	A	G	-0.135	0.008	0.105
rs139154032	11	116678000	1.83E-11	A	G	-0.137	0.019	0.0164
rs1475537	9	92212750	8.93E-10	T	C	0.037	0.006	0.263
rs16990971	20	44601293	3.12E-13	G	A	0.079	0.010	0.0609
rs174559	11	61581656	1.32E-12	A	G	0.041	0.006	0.409
rs2954021	8	126482077	7.97E-42	A	G	0.071	0.005	0.435
rs34828061	8	59382169	1.80E-08	G	A	-0.033	0.006	0.262
rs35570672	8	18272635	8.75E-15	C	T	-0.041	0.005	0.434
rs3752442	4	3446883	1.09E-11	G	A	-0.038	0.005	0.453
rs3755980	4	88042251	4.85E-09	T	C	0.032	0.005	0.364
rs4418728	10	94839724	3.28E-09	G	T	0.039	0.006	0.214
rs4495740	1	63124465	1.53E-34	G	T	-0.082	0.006	0.186
rs4704834	5	156443066	3.51E-18	A	G	-0.053	0.006	0.248
rs483082	19	45416178	4.37E-49	T	G	0.113	0.007	0.166
rs58542926	19	19379549	3.00E-17	T	C	-0.090	0.010	0.067
rs651821	11	116662579	4.95E-525	C	T	0.291	0.006	0.283
rs6706783	2	21191409	1.44E-13	A	T	0.062	0.008	0.105
rs7078456	10	65269479	1.53E-09	C	T	0.034	0.005	0.33
rs7165077	15	58686809	2.89E-18	T	C	-0.050	0.005	0.359
rs74855321	8	19836984	2.40E-92	T	C	-0.177	0.008	0.0986
rs7928320	11	116942753	2.98E-42	G	C	-0.117	0.008	0.103
rs7928320	11	116942753	2.98E-42	G	C	-0.117	0.008	0.103
rs821840	16	56993886	4.51E-10	G	A	-0.044	0.007	0.163
rs9687832	5	55861595	4.44E-08	A	G	0.045	0.008	0.111

Notes: R^2 for SNP: $R^2 = \Sigma\beta^2 \times 2 \times EAF \times (1 - EAF)$, where β denoted the effect size.

F for each drug-target GRS: $F = [(N - K - 1) / K] \times R^2 / (1 - R^2)$, where N was the sample size and K was the number of SNPs included in the GRS.

R^2 of TG GRS = 0.075; F of TG GRS = 439.83.

Abbreviations: CHR, chromosome; BP, base pair; EA, effect allele; OA, other allele; SE, standard error; EAF, effect allele frequency; GRS, genetic risk score; TG, triglyceride.

Table S4. Instrument variables applied for TC GRS. Related to Figure 3.

SNP	CHR	BP	P	EA	OA	Beta	SE	EAF
rs10438978	18	47158186	2.72E-10	T	C	-0.028137	0.00420706	0.277
rs1065853	19	45413233	5.80E-314	T	G	-0.370244	0.00922769	0.0503
rs1077835	15	58723426	4.21E-53	G	A	0.0633784	0.00390075	0.455
rs10846744	12	125312425	1.12E-09	G	C	-0.0291246	0.00451383	0.376
rs10860591	12	100822511	3.83E-14	C	T	0.0341989	0.0042668	0.281
rs10889348	1	63078222	9.17E-20	T	A	-0.0497546	0.00516258	0.163
rs11102967	1	109817245	4.29E-68	C	T	-0.137375	0.00743748	0.0707
rs112784971	8	59406490	8.27E-25	T	C	-0.0462477	0.00424522	0.27
rs11320208	1	234850422	2.84E-20	A	C	-0.0412222	0.00421865	0.27
rs11601507	11	5701074	1.04E-09	A	C	0.0590618	0.00913568	0.084
rs12162136	17	67082266	4.63E-17	G	A	0.0340664	0.00383063	0.408
rs12173764	6	31314012	5.05E-14	T	C	-0.0322456	0.00404242	0.405
rs12233358	22	30432631	3.62E-09	G	C	0.0399809	0.00639682	0.0956
rs12314392	12	110010866	2.49E-09	G	A	0.0270593	0.00428454	0.275
rs12484801	22	44325565	1.50E-11	T	C	-0.0275028	0.00384796	0.417
rs12995972	2	62956520	7.58E-14	A	C	-0.034681	0.00437859	0.261
rs151021730	19	50031075	1.80E-08	A	G	-0.0619466	0.0103872	0.0372
rs17129638	10	113981435	2.05E-11	C	T	-0.0291265	0.00410254	0.295
rs17231506	16	56994528	1.12E-43	T	C	0.0716347	0.00487972	0.181
rs174565	11	61591636	2.05E-20	G	C	-0.0397076	0.0040483	0.4
rs17660635	8	9197250	2.44E-09	G	A	-0.10843	0.0171596	0.0122
rs184643955	16	71452979	1.88E-08	A	G	0.0986131	0.0165573	0.0134
rs2206689	20	39209518	8.44E-13	A	G	0.028864	0.0038091	0.435
rs2263608	10	113944940	4.64E-17	T	A	0.038167	0.00429186	0.265
rs2306986	4	100504575	6.37E-10	C	G	0.0402142	0.00614219	0.203
rs2714875	7	17925975	1.28E-08	A	G	0.0250345	0.00415409	0.294
rs2737265	8	116667634	1.03E-13	G	A	-0.032534	0.00412984	0.286
rs2738464	19	11242307	7.78E-80	G	C	-0.0850829	0.00424537	0.298
rs2740488	9	107661742	1.14E-63	C	A	-0.0765346	0.00428923	0.252
rs2807834	1	220970593	4.39E-14	T	G	-0.0422582	0.00528475	0.153
rs2954021	8	126482077	8.01E-43	A	G	0.0547932	0.00377102	0.44
rs3809868	17	45750596	3.29E-12	G	A	0.0296915	0.00402474	0.35
rs3810308	19	11333596	3.84E-59	C	T	-0.0750637	0.00436982	0.25
rs4344974	20	39818861	6.73E-14	T	C	-0.0401339	0.00505656	0.168
rs4454000	4	69950571	7.30E-10	G	A	-0.0272272	0.00417316	0.313
rs4622073	1	93255073	5.96E-11	A	G	-0.0554232	0.00799476	0.062
rs4690014	4	3436062	1.31E-15	A	G	-0.0331682	0.00391727	0.419
rs532436	9	136149830	4.56E-50	A	G	0.0685843	0.00435182	0.243
rs55686478	8	18250375	1.51E-09	G	A	-0.024068	0.00375991	0.491
rs56130071	7	21598753	1.56E-10	C	G	0.0299187	0.00441349	0.268
rs57176252	10	74662593	2.77E-13	A	C	-0.0356643	0.0046089	0.218
rs5758361	22	41852732	3.93E-10	C	A	0.0276585	0.00417331	0.355
rs57825321	2	21247065	8.47E-78	A	T	-0.114637	0.00579626	0.119
rs61946969	13	32946805	6.48E-15	A	G	-0.0315782	0.00382483	0.415
rs6437108	2	158455135	1.17E-17	T	C	-0.0393568	0.00434274	0.276
rs6453131	5	74644706	7.43E-65	T	G	-0.0677423	0.00376062	0.496

rs651821	11	116662579	1.85E-38	C	T	0.0571283	0.00415872	0.31
rs6663252	1	55630151	2.46E-34	C	T	-0.0743687	0.00574581	0.124
rs6874202	5	156391628	2.23E-38	T	C	-0.062447	0.00455101	0.219
rs6959252	7	25984566	1.18E-09	A	G	0.0259676	0.00403025	0.401
rs7165077	15	58686809	2.34E-19	T	C	-0.0513008	0.00538352	0.359
rs72607108	9	107592641	1.47E-11	G	T	-0.0494518	0.00691596	0.0793
rs7312955	12	123747783	1.68E-08	A	C	0.0254173	0.00425314	0.29
rs7400614	13	114551980	3.36E-11	A	C	0.0304648	0.0043381	0.301
rs75352129	2	20848192	7.85E-28	T	C	0.136357	0.0117723	0.0263
rs77303550	16	72079657	9.24E-67	T	C	-0.0881541	0.0048214	0.228
rs7770628	6	161018174	2.91E-15	C	T	0.0635399	0.00759832	0.118
rs78753419	1	55811671	3.41E-10	T	A	0.0345461	0.00519426	0.155
rs7954039	12	121398654	1.67E-15	A	C	0.0318793	0.00377914	0.492
rs814295	2	27743215	1.40E-33	G	A	-0.0510712	0.00399223	0.321
rs9376090	6	135411228	4.95E-14	C	T	-0.0448819	0.00562452	0.281
rs9399668	6	101161950	7.42E-09	T	C	-0.0257582	0.00420637	0.292
rs9953437	18	47120600	3.98E-49	A	G	0.0594315	0.00380832	0.428

Notes: R^2 for SNP: $R^2 = \Sigma\beta^2 \times 2 \times EAF \times (1 - EAF)$, where β denoted the effect size.

F for each drug-target GRS: $F = [(N - K - 1) / K] \times R^2 / (1 - R^2)$, where N was the sample size and K was the number of SNPs included in the GRS

R^2 of TC GRS = 0.066; F of TC GRS = 164.32

Abbreviations: CHR, chromosome; BP, base pair; EA, effect allele; OA, other allele; SE, standard error; EAF, effect allele frequency; GRS, genetic risk score; TG, triglyceride.

Table S5. Instrument variables applied for glucose GRS. Related to Figure 3.

SNP	chr	bp19	EA	OA	Beta	SE	EAF	P
rs10010131	4	6292915	A	G	-0.045	0.008	0.069	3.55E-08
rs10158845	1	43455283	A	G	-0.026	0.004	0.513	4.68E-09
rs10267664	7	156770498	C	G	-0.031	0.005	0.294	1.51E-10
rs10441113	7	44288665	A	G	-0.041	0.007	0.127	9.12E-10
rs10811658	9	22128600	A	G	-0.042	0.005	0.478	9.55E-21
rs10830963	11	92708710	C	G	-0.084	0.005	0.590	2.63E-77
rs10882083	10	94321973	C	G	0.026	0.005	0.372	1.45E-08
rs12429454	13	28481210	A	G	0.047	0.004	0.471	4.57E-28
rs12443160	15	40656141	T	C	0.024	0.004	0.436	1.66E-08
rs1260326	2	27730940	T	C	-0.059	0.004	0.492	9.12E-46
rs13023591	2	173595699	T	C	0.050	0.004	0.514	7.24E-31
rs1402837	2	169757354	T	C	0.106	0.004	0.404	1.23E-133
rs1406981	6	117269641	T	C	0.027	0.004	0.620	1.95E-10
rs1409333	10	95390919	T	C	-0.028	0.004	0.444	2.40E-11
rs1420566	16	56065760	A	G	0.024	0.004	0.406	3.80E-08
rs16922302	9	672920	C	G	0.059	0.005	0.265	5.25E-35
rs17085675	5	95727664	A	T	0.043	0.004	0.682	1.62E-21
rs17168486	7	14898282	T	C	0.055	0.004	0.479	7.76E-40
rs174554	11	61579463	A	G	0.047	0.004	0.422	1.15E-27
rs1974619	7	15065300	T	C	0.069	0.005	0.675	1.74E-49
rs204926	11	8255106	A	G	-0.038	0.005	0.333	5.75E-16
rs2237896	11	2858440	A	G	-0.045	0.005	0.345	7.08E-22
rs2273800	14	100847707	A	G	0.027	0.004	0.554	6.17E-10
rs231840	11	2724130	T	C	0.032	0.006	0.813	1.32E-08
rs243020	2	60585028	A	G	-0.031	0.005	0.351	1.15E-11
rs2908289	7	44223942	A	G	0.129	0.005	0.196	3.63E-131
rs340515	2	45188370	T	G	-0.097	0.004	0.530	3.16E-104
rs340878	1	214157049	C	G	0.036	0.006	0.814	5.25E-10
rs35674932	10	12261810	A	T	-0.037	0.004	0.449	2.04E-18
rs363404	10	119009631	T	C	-0.025	0.005	0.536	3.24E-08
rs3755934	4	1236083	T	C	-0.039	0.004	0.334	4.90E-18
rs3787186	20	50153344	T	C	0.030	0.005	0.375	7.76E-11
rs3802177	8	118185025	A	G	-0.072	0.004	0.462	2.19E-67
rs419145	1	33179156	A	G	0.030	0.005	0.215	4.90E-09
rs4339696	9	4295880	T	G	-0.037	0.004	0.427	1.10E-18
rs459193	5	55806751	A	G	-0.029	0.004	0.469	5.75E-12
rs519084	13	26821164	A	T	0.026	0.005	0.433	4.79E-09
rs55804588	7	75852586	C	G	-0.041	0.007	0.861	2.63E-10
rs56237081	2	222036237	T	C	0.037	0.006	0.198	4.57E-11
rs56252704	13	33556228	A	G	0.047	0.005	0.209	1.58E-19
rs59299413	7	127843398	C	G	0.040	0.007	0.135	3.47E-09

rs6048206	20	22561382	T	C	0.096	0.006	0.857	1.95E-56
rs61839365	10	26515666	A	G	-0.036	0.005	0.265	4.68E-14
rs6495240	15	77766556	T	C	-0.029	0.004	0.655	3.16E-11
rs67131976	6	20686878	T	C	0.061	0.004	0.342	7.59E-43
rs6780171	3	185503456	A	T	0.029	0.005	0.233	5.01E-09
rs6932473	6	153438573	A	T	-0.025	0.004	0.326	1.20E-08
rs6972160	7	89815373	C	G	-0.036	0.006	0.822	6.31E-11
rs7109575	11	72463435	A	G	-0.066	0.008	0.067	1.00E-14
rs7161785	15	62395224	C	G	-0.054	0.004	0.340	1.38E-34
rs7165887	15	51750846	A	G	-0.027	0.005	0.405	4.07E-09
rs7209518	17	67580175	A	C	0.026	0.005	0.639	2.63E-08
rs883541	17	66449122	A	G	-0.026	0.004	0.525	7.59E-10
rs9263964	6	31186039	T	C	0.025	0.004	0.422	1.74E-09
rs9296285	6	39040469	A	G	0.048	0.005	0.766	5.13E-22
rs9788833	16	292772	T	C	0.027	0.004	0.413	9.33E-10
rs9873341	3	152387723	T	C	0.032	0.005	0.276	1.82E-10

Notes: R^2 for SNP: $R^2 = \Sigma\beta^2 \times 2 \times EAF \times (1 - EAF)$, where β denoted the effect size.

F for each drug-target GRS: $F = [(N - K - 1) / K] \times R^2 / (1 - R^2)$, where N was the sample size and K was the number of SNPs included in the GRS

R^2 of glucose GRS = 0.056; F of glucose GRS = 95.56

Abbreviations: CHR, chromosome; BP, base pair; EA, effect allele; OA, other allele; SE, standard error; EAF, effect allele frequency; GRS, genetic risk score; TG, triglyceride.

Table S6. Instrument variables applied for GRS of APOC3-pQTL in the blood. Related to Figure 3.

SNP	CHR	BP	EA	OA	N	MAF	Beta	SE	P	R^2	F
rs651821	11	116662579	C	T	2410	0.282197	0.106423	0.03115	0.000638	0.00458837	11.0997303

Notes: $R2 = \Sigma\beta^2 \times 2 \times EAF \times (1 - EAF)$, where β denoted the effect size.

$F = [(N - K - 1) / K] \times R^2 / (1 - R^2)$, where N=2410, K = 1

As the TG-lowering drugs (olezarsen and volanesorsen) are inhibitors of APOC3, we multiplied the initial APOC3-pQTL-GRS by (-1) to simulate the drug-induced APOC3 inhibition and the corresponding lipid-lowering effect after treatment.

Abbreviations: SNP, single nucleotide polymorphism; CHR, chromosome; BP, base pair; EA, effect allele; OA, other allele; GRS, genetic risk score; MAF, minor allele frequency.

Table S7. Instrumental variables applied for CSF-pQTL GRSs of GCK. Related to Figure 3.

Gene	SNP	CHR	BP	OA	EA	EAF_1000G EUR	EAF_1000G EAS	EAF_CAPOC	Beta	SE	P	F
GCK	rs3757840	7	44191617	T	G	0.467	0.479	0.464	0.073	0.023	0.0017	10.073724

Notes: As the allele frequency was not available in the CSF-pQTL dataset, F value was calculated by: $F= (beta/SE)^2$

As the antidiabetic drug - dorzagliatin is an agonist of GCK, we retained the original coefficients when calculating GCK-pQTL GRS to mimic the activation of GCK and the glucose-lowering effect of the treatment.

Abbreviations: SNP, single nucleotide polymorphism; CHR, chromosome; BP, base pair; EA, effect allele; OA, other allele; GRS, genetic risk score; CSF, cerebrospinal fluid.

EAF information was based on the 1000 Genomes Project Phase 3; CAPOC, Chinese Antipsychotics Pharmacogenomics Consortium.

Table S8. Associations between lipid-lowering drug-target GRSs, *GCK* GRS, with lipids in the CAPOC study. Related to Figure 2.

Trait	Gene	Beta	SE	CI	P	P.FDR
LDLC	*ABCA1*	0.437	0.618	-0.774 ~ 1.649	0.479	0.561
	PCSK9	-0.607	0.611	-1.805 ~ 0.590	0.320	0.451
	APOB	-1.667	0.620	-2.882 ~ -0.451	0.007	0.031
	LDLR	-1.068	0.623	-2.290 ~ 0.155	0.087	0.190
	LPA	-0.612	0.618	-1.825 ~ 0.600	0.322	0.451
	HMGCR	-1.409	0.620	-2.625 ~ -0.194	0.023	0.081
	APOC3	-1.168	0.621	-2.385 ~ 0.050	0.060	0.150
	GCK	0.490	0.619	-0.724 ~ 1.704	0.429	0.536
HDLC	*PPARG*	-0.373	0.287	-0.935 ~ 0.189	0.193	0.375
	HCAR3	-0.245	0.288	-0.809 ~ 0.320	0.395	0.512
	HCAR2	-0.245	0.288	-0.809 ~ 0.320	0.395	0.512
	ABCA1	-0.853	0.284	-1.410 ~ -0.296	0.003	0.021
	MTTP	-0.319	0.285	-0.878 ~ 0.240	0.264	0.431
	APOC3	-0.406	0.284	-0.963 ~ 0.152	0.153	0.315
	CETP	-1.164	0.285	-1.724 ~ -0.604	4.72E-05	0.001
	APOB	-0.116	0.290	-0.684 ~ 0.453	0.690	0.710
	LDLR	-0.708	0.282	-1.261 ~ -0.155	0.012	0.047
	LPL	-1.095	0.280	-1.644 ~ -0.545	9.72E-05	0.001
	LPA	0.172	0.284	-0.385 ~ 0.729	0.544	0.595
	GCK	0.305	0.285	-0.253 ~ 0.863	0.283	0.431
TG	*ANGPTL3*	-1.925	1.124	-4.129 ~ 0.280	0.087	0.190
	APOC3	-6.343	1.118	-8.534 ~ -4.151	1.58E-08	5.53E-07
	CETP	-2.350	1.133	-4.572 ~ -0.129	0.038	0.121
	APOB	-2.287	1.125	-4.494 ~ -0.081	0.042	0.123
	LPL	-1.220	1.106	-3.390 ~ 0.950	0.270	0.431
	GCK	-0.695	1.123	-2.896 ~ 1.506	0.536	0.595
TC	*ANGPTL3*	-0.002	0.715	-1.404 ~ 1.400	0.998	0.998
	APOB	-2.010	0.715	-3.412 ~ -0.609	0.005	0.025
	APOC3	-2.016	0.718	-3.424 ~ -0.607	0.005	0.025
	CETP	-0.308	0.719	-1.718 ~ 1.102	0.668	0.708
	HMGCR	-1.394	0.715	-2.796 ~ 0.008	0.051	0.137
	LDLR	-2.108	0.716	-3.513 ~ -0.703	0.003	0.021
	LPA	-0.873	0.712	-2.270 ~ 0.523	0.220	0.405
	PCSK9	-0.766	0.704	-2.146 ~ 0.614	0.276	0.431
	GCK	0.503	0.713	-0.895 ~ 1.901	0.481	0.561

Notes: Adjusted for age, age^2, gender, center, first five principal components, course of disease, and previous drug usage.

False discovery rate (FDR)-corrected P-value < 0.05 was applied as the significant threshold accounting for multiple tests across drug targets and outcomes. The conventional P<0.05 was applied as the threshold of suggestive significance.

Abbreviations: LDLC, low-density lipoprotein cholesterol; HDLC, high-density lipoprotein cholesterol; TG, triglyceride; TC, total cholesterol; GRS, genetic risk score; SE, standard error; CAPOC, Chinese Antipsychotics Pharmacogenomics Consortium.

Table S9. Associations between antidiabetic target GRSs, *APOC3* GRS with glucose in the CAPOC study. Related to Figure 2.

Trait	Gene	Beta	SE	CI	P	P.FDR
Glucose	*ABCB11*	-1.296	0.283	-1.851 ~ -0.742	4.80E-06	1.92E-05
	GCK	-0.675	0.283	-1.229 ~ -0.120	0.017	0.034
	GLP1R	-0.282	0.286	-0.842 ~ 0.279	0.325	0.433
	APOC3	0.214	0.284	-0.344 ~ 0.771	0.452	0.452

Notes: Adjusted for age, age^2, gender, center, first five principal components, course of disease, and previous drug usage.

False discovery rate (FDR)-corrected P-value < 0.05 was applied as the significant threshold accounting for multiple tests across drug targets and outcomes. The conventional P<0.05 was applied as the threshold of suggestive significance.

Abbreviations: GRS, genetic risk score; CAPOC, Chinese Antipsychotics Pharmacogenomics Consortium study; SE, standard error.

Table S10. Associations between drug-target GRSs and baseline characteristics. Related to Table 1.

CAPOC study								
**GRS**	**Outcome**	**Model**	**Coff***	**SE**	**CI_low**	**CI_high**	**P**	**P.FDR**
*APOC3*_TC	Gender	logistic	1.021	0.045	0.936	1.114	0.644	0.910
	Age (years)	linear	-0.022	0.021	-0.064	0.020	0.302	0.910
	Center is south	logistic	1.056	0.101	0.867	1.286	0.588	0.910
	First episode	logistic	0.979	0.057	0.875	1.095	0.707	0.910
	Course of SCZ	linear	0.580	1.297	-1.962	3.121	0.655	0.910
	With previous medication	logistic	1.025	0.048	0.932	1.127	0.614	0.910
	Education_lower than middle school	logistic	0.989	0.045	0.905	1.081	0.809	0.910
	Married	logistic	0.967	0.054	0.869	1.075	0.533	0.910
	SBP (mmHg)	linear	-0.154	0.238	-0.621	0.312	0.517	0.910
	DBP (mmHg)	linear	-0.144	0.174	-0.485	0.197	0.407	0.910
	Pulse	linear	-0.219	0.223	-0.657	0.218	0.325	0.910
	BMI (kg/m^2)	linear	-0.022	0.072	-0.164	0.120	0.757	0.910
*APOC3*_TG	Gender	logistic	0.988	0.044	0.906	1.078	0.792	0.910
	Age (years)	linear	-0.018	0.021	-0.060	0.024	0.403	0.910
	Center is south	logistic	1.041	0.097	0.862	1.259	0.675	0.910
	First episode	logistic	0.982	0.056	0.881	1.096	0.749	0.910
	Course of SCZ	linear	-0.564	1.290	-3.092	1.964	0.662	0.910
	With previous medication	logistic	1.081	0.048	0.984	1.188	0.105	0.910
	Education_lower than middle school	logistic	1.009	0.045	0.924	1.103	0.839	0.916
	Married	logistic	0.985	0.055	0.885	1.097	0.786	0.910
	SBP (mmHg)	linear	-0.405	0.237	-0.869	0.059	0.087	0.910
	DBP (mmHg)	linear	-0.280	0.173	-0.619	0.059	0.106	0.910
	Pulse	linear	-0.065	0.222	-0.500	0.370	0.770	0.910
	BMI (kg/m^2)	linear	-0.071	0.072	-0.212	0.070	0.326	0.910
*GCK*_glucose	Gender	logistic	1.045	0.044	0.959	1.139	0.318	0.910
	Age (years)	linear	-0.042	0.021	-0.083	0.000	0.050	0.910
	Center is south	logistic	1.008	0.100	0.828	1.226	0.939	0.939
	First episode	logistic	0.936	0.056	0.838	1.045	0.237	0.910
	Course of SCZ	linear	-1.222	1.285	-3.740	1.296	0.342	0.910

	With previous medication	logistic	0.948	0.048	0.863	1.042	0.270	0.910
	Education_lower than middle school	logistic	1.048	0.045	0.959	1.145	0.301	0.910
	Married	logistic	1.022	0.054	0.919	1.137	0.688	0.910
	SBP (mmHg)	linear	-0.114	0.236	-0.576	0.349	0.630	0.910
	DBP (mmHg)	linear	0.015	0.173	-0.323	0.353	0.930	0.939
	Pulse	linear	0.265	0.221	-0.168	0.698	0.231	0.910
	BMI (kg/m^2)	linear	0.011	0.072	-0.130	0.151	0.884	0.936

CAPEC study

GRS	Outcome	Model	Coff*	SE	CI_low	CI_high	P	P.FDR
*APOC3*_TC	Gender	logistic	0.912	0.136	0.699	1.191	0.500	0.852
	Age (years)	linear	0.047	0.105	-0.159	0.253	0.658	0.852
	Center	logistic	0.833	0.237	0.523	1.327	0.442	0.852
	First episode	logistic	0.818	0.149	0.611	1.094	0.175	0.852
	SBP (mmHg)	linear	-0.371	0.723	-1.787	1.045	0.608	0.852
	DBP (mmHg)	linear	-0.300	0.540	-1.358	0.758	0.579	0.852
	BMI (kg/m^2)	linear	0.396	0.244	-0.083	0.874	0.106	0.852
*APOC3*_TG	Gender	logistic	0.854	0.133	0.659	1.108	0.235	0.852
	Age (years)	linear	0.040	0.102	-0.160	0.240	0.697	0.852
	Center	logistic	0.894	0.234	0.565	1.415	0.633	0.852
	First episode	logistic	0.878	0.143	0.664	1.163	0.365	0.852
	SBP (mmHg)	linear	-0.204	0.701	-1.579	1.170	0.771	0.852
	DBP (mmHg)	linear	-0.220	0.524	-1.247	0.807	0.675	0.852
	BMI (kg/m^2)	linear	0.345	0.237	-0.120	0.810	0.147	0.852
*GCK*_glucose	Gender	logistic	0.858	0.127	0.669	1.099	0.225	0.852
	Age (years)	linear	-0.071	0.098	-0.263	0.120	0.466	0.852
	Center	logistic	0.932	0.232	0.592	1.468	0.760	0.852
	First episode	logistic	0.906	0.136	0.695	1.182	0.466	0.852
	SBP (mmHg)	linear	0.106	0.674	-1.215	1.427	0.875	0.879
	DBP (mmHg)	linear	0.077	0.504	-0.910	1.064	0.879	0.879
	BMI (kg/m^2)	linear	0.222	0.228	-0.226	0.669	0.333	0.852

Note: For logistic regression models, Coff represents the odds ratio (OR) and SE represents the standard error of log(OR); for linear regression models, “Coff” represents the regression coefficient (β) and SE represents the standard error of β.

The models were adjusted for gender, age, study centers, SCZ course, previous drug usage, PC1~5 (excluding any variable from the covariates when it served as the outcome)
Abbreviations: BMI, body mass index; SBP, systolic blood pressure; DBP, diastolic blood pressure; GRS, genetic risk score; CAPOC, Chinese Antipsychotics Pharmacogenomics Consortium study; CAPEC, Chinese Antipsychotics Pharmacogenetics Consortium.

Table S11. Baseline characteristics of participants in the CAPEC study. Related to Table 1

Baseline characteristics	All	*APOC3*_GRS		*GCK*_GRS	
		Lower	Higher	Lower	Higher
N	292	146 (50.0)	146 (50.0)	130 (44.5)	162 (55.5)
Male (%)	132 (45.2)	64 (43.8)	68 (46.6)	58 (44.6)	74 (45.7)
Female (%)	160 (54.8)	82 (56.2)	78 (53.4)	72 (55.4)	88 (54.3)
Age (years)	32.0 (23.0, 40.0)	31.5 (23.2, 39.0)	32.0 (23.0, 41.0)	31.5 (24.0, 39.0)	32.0 (23.0, 41.0)
First episode (%)	94 (32.2)	48 (32.9)	46 (31.5)	39 (30.0)	55 (34.0)
TC (mg/dL)	154.7 (135.3, 182.2)	154.7 (136.1, 185.4)	154.7 (135.3, 177.3)	157.2 (136.3, 184.5)	151.6 (135.3, 181.7)
TG (mg/dL)	93.0 (70.6, 132.9)	97.4 (75.5, 137.7)	89.5 (64.9, 128.9)	98.8 (74.6, 130.9)	89.0 (64.9, 134.6)
HDLC (mg/dL)	46.4 (36.7, 55.7)	44.5 (35.6, 55.2)	47.2 (38.5, 56.7)	45.6 (37.5, 54.9)	46.8 (36.7, 57.1)
LDLC (mg/dL)	90.9 (74.5, 119.5)	94.7 (76.4, 121.3)	88.0 (74.3, 114.3)	94.5 (75.2, 121.4)	90.5 (74.0, 116.7)
Glucose (mg/dL)	83.4 (75.5, 93.6)	82.6 (73.6, 92.4)	84.5 (77.4, 93.7)	83.9 (75.9, 93.7)	83.3 (75.3, 93.3)

Notes: The median and interquartile range (IQR) values were displayed for continuous variables.

Abbreviations: LDLC, low-density lipoprotein cholesterol; HDLC, high-density lipoprotein cholesterol; TG, triglyceride; TC, total cholesterol; GRS, genetic risk score; CAPEC, Chinese Antipsychotics Pharmacogenetics Consortium study.

Table S14. Associations between *GCK, APOC3* GRSs with glucose, lipids in the CAPEC study. Related to Figure 2.

Gene	Trait	Beta	SE	CI	P	P.FDR
GCK	Glucose	0.131	0.956	-1.751 ~ 2.014	0.891	0.891
*APOC3*_TG	TG	-4.131	3.819	-11.651 ~ 3.388	0.280	0.420
*APOC3*_TC	TC	-2.945	2.307	-7.488 ~ 1.598	0.203	0.420

Note: Adjusted for age, age^2, gender, center, first five principal components, course of disease, and previous drug usage.

False discovery rate (FDR)-corrected P-value < 0.05 was applied as the significant threshold accounting for multiple tests across drug targets and outcomes. The conventional P<0.05 was applied as the threshold of suggestive significance.

Abbreviations: GRS, genetic risk score; CAPEC, Chinese Antipsychotics Pharmacogenetics Consortium study; SE, standard error. PANSS, Positive and Negative Syndrome Scale; N, Negative scale; G, General psychopathology scale; P, Positive scale; SE, standard error. LDLC, low-density lipoprotein cholesterol; HDLC, high-density lipoprotein cholesterol; TG, triglyceride; TC, total cholesterol.

Table S15. Conventional observational associations between TG, TC, and PANSS percentage reduction in the CAPOC study. Related to Figure 3.

Exposure	PANSS	Beta	SE	CI	P	P.FDR
TG	PANSS	0.014	0.012	-0.010 ~ 0.037	0.251	0.568
	PANSS_P	0.014	0.010	-0.005 ~ 0.033	0.148	0.568
	PANSS_N	0.012	0.009	-0.006 ~ 0.030	0.187	0.568
	PANSS_G	0.003	0.008	-0.012 ~ 0.018	0.703	0.803
TC	PANSS	-0.007	0.019	-0.044 ~ 0.029	0.692	0.803
	PANSS_P	0.003	0.015	-0.026 ~ 0.033	0.825	0.825
	PANSS_N	0.006	0.014	-0.022 ~ 0.034	0.668	0.803
	PANSS_G	-0.013	0.012	-0.037 ~ 0.011	0.284	0.568

Note: Adjusted for age, age^2, gender, center, course of disease, previous drug usage, and assigned medication.

False discovery rate (FDR)-corrected P-value < 0.05 was applied as the significant threshold accounting for multiple tests across drug targets and outcomes. The conventional P<0.05 was applied as the threshold of suggestive significance.

Abbreviations: PANSS, Positive and Negative Syndrome Scale; N, Negative scale; G, General psychopathology scale; P, Positive scale; SE, standard error. TG, triglyceride; TC, total cholesterol; CAPOC, Chinese Antipsychotics Pharmacogenomics Consortium.

Table S16. Conventional observational associations between lipids and better antipsychotic efficacy in the CAPOC study. Related to Figure 3.

Exposure	PANSS	OR	SE_logOR	CI	P	P.FDR
TG	PANSS	1.001	0.001	1.000 ~ 1.003	0.135	0.419
	PANSS_P	1.001	0.001	0.999 ~ 1.003	0.257	0.514
	PANSS_N	1.000	0.001	0.998 ~ 1.002	0.843	0.994
	PANSS_G	1.000	0.001	0.998 ~ 1.002	0.931	0.994
TC	PANSS	0.998	0.001	0.995 ~ 1.000	0.081	0.419
	PANSS_P	0.998	0.001	0.995 ~ 1.001	0.157	0.419
	PANSS_N	1.001	0.001	0.998 ~ 1.004	0.525	0.840
	PANSS_G	1.000	0.001	0.997 ~ 1.003	0.994	0.994

Note: Adjusted for age, age^2, gender, center, course of disease, previous drug usage, and assigned medication.

Better antipsychotic efficacy: PANSS percentage reduction > median value of PANSS percentage reduction; The standard error (SE) corresponds to the beta coefficient derived from the natural logarithm of the odds ratio (log OR);

False discovery rate (FDR)-corrected P-value < 0.05 was applied as the significant threshold accounting for multiple tests across drug targets and outcomes. The conventional P<0.05 was applied as the threshold of suggestive significance.

Abbreviations: PANSS, Positive and Negative Syndrome Scale; N, Negative scale; G, General psychopathology scale; P, Positive scale; SE, standard error. TG, triglyceride; TC, total cholesterol; CAPOC, Chinese Antipsychotics Pharmacogenomics Consortium.

Table S17. One-sample Mendelian randomization between lipid and PANSS percentage reduction in the CAPOC study. Related to Figure 3.

Exposure	PANSS	Beta	SE	CI	P	P.FDR
TG	PANSS	-0.068	0.072	-0.209 ~ 0.073	0.346	0.635
	PANSS_P	-0.025	0.058	-0.138 ~ 0.089	0.669	0.766
	PANSS_N	-0.160	0.055	-0.268 ~ -0.052	0.004	0.032
	PANSS_G	-0.033	0.047	-0.125 ~ 0.058	0.476	0.635
TC	PANSS	-0.061	0.077	-0.211 ~ 0.090	0.430	0.635
	PANSS_P	-0.009	0.061	-0.129 ~ 0.112	0.890	0.890
	PANSS_N	-0.048	0.059	-0.163 ~ 0.068	0.416	0.635
	PANSS_G	-0.048	0.050	-0.145 ~ 0.050	0.337	0.635

Note: Adjusted for age, age^2, gender, center, course of disease, previous drug usage, assigned medication, first five principal components.

False discovery rate (FDR)-corrected P-value < 0.05 was applied as the significant threshold accounting for multiple tests across drug targets and outcomes. The conventional P<0.05 was applied as the threshold of suggestive significance.

Abbreviations: PANSS, Positive and Negative Syndrome Scale; N, Negative scale; G, General psychopathology scale; P, Positive scale; SE, standard error. TG, triglyceride; TC, total cholesterol; CAPOC, Chinese Antipsychotics Pharmacogenomics Consortium.

Table S18. One-sample Mendelian randomization between lipids and better antipsychotic efficacy in the CAPOC study. Related to Figure 3.

Exposure	PANSS	OR	SE_logOR	CI	P	P.FDR
TG	PANSS	0.993	0.005	0.983 ~ 1.003	0.187	0.499
	PANSS_P	1.001	0.005	0.990 ~ 1.011	0.923	0.923
	PANSS_N	0.979	0.005	0.968 ~ 0.989	3.97E-05	3.18E-04
	PANSS_G	0.999	0.005	0.988 ~ 1.009	0.793	0.906
TC	PANSS	0.990	0.006	0.980 ~ 1.001	0.085	0.340
	PANSS_P	0.995	0.006	0.984 ~ 1.006	0.395	0.632
	PANSS_N	0.996	0.006	0.986 ~ 1.007	0.511	0.681
	PANSS_G	0.995	0.006	0.984 ~ 1.006	0.347	0.632

Note: Adjusted for age, age^2, gender, center, course of disease, previous drug usage, assigned medication, first five principal components.

Better antipsychotic efficacy: PANSS percentage reduction > median value of PANSS percentage reduction; The standard error (SE) corresponds to the beta coefficient derived from the natural logarithm of the odds ratio (log OR).

False discovery rate (FDR)-corrected P-value < 0.05 was applied as the significant threshold accounting for multiple tests across drug targets and outcomes. The conventional P<0.05 was applied as the threshold of suggestive significance.

Abbreviations: PANSS, Positive and Negative Syndrome Scale; N, Negative scale; G, General psychopathology scale; P, Positive scale; TG, triglyceride; TC, total cholesterol; CAPOC, Chinese Antipsychotics Pharmacogenomics Consortium.

Table S19. Associations between lipid-lowering drug-target GRSs and PANSS percentage reduction in the CAPOC study. Related to Figure 3.

Trait	Gene	PANSS	Beta	SE	CI_lower	CI_upper	P	P.FDR
TG	*APOC3*	PANSS	0.745	0.617	-0.464	1.955	0.227	0.908
	APOC3	PANSS_P	0.240	0.495	-0.730	1.211	0.628	0.946
	APOC3	PANSS_N	1.230	0.474	0.301	2.159	0.009	0.324
	APOC3	PANSS_G	0.314	0.400	-0.471	1.099	0.433	0.946
LDLC	*APOB*	PANSS	0.389	0.616	-0.819	1.596	0.528	0.946
	APOB	PANSS_P	0.403	0.494	-0.565	1.371	0.415	0.946
	APOB	PANSS_N	-0.443	0.473	-1.371	0.486	0.350	0.946
	APOB	PANSS_G	0.137	0.399	-0.647	0.920	0.732	0.946
HDLC	*ABCA1*	PANSS	0.242	0.615	-0.963	1.447	0.694	0.946
	ABCA1	PANSS_P	0.202	0.493	-0.765	1.169	0.682	0.946
	ABCA1	PANSS_N	-0.171	0.473	-1.098	0.756	0.718	0.946
	ABCA1	PANSS_G	0.320	0.399	-0.461	1.102	0.422	0.946
	CETP	PANSS	-1.076	0.618	-2.289	0.137	0.082	0.691
	CETP	PANSS_P	-0.825	0.496	-1.798	0.148	0.096	0.691
	CETP	PANSS_N	-0.691	0.476	-1.624	0.242	0.146	0.751
	CETP	PANSS_G	-0.727	0.401	-1.514	0.059	0.070	0.691
	LDLR	PANSS	-0.033	0.610	-1.230	1.164	0.956	0.981
	LDLR	PANSS_P	0.035	0.490	-0.925	0.995	0.942	0.981
	LDLR	PANSS_N	-0.011	0.469	-0.931	0.909	0.981	0.981
	LDLR	PANSS_G	-0.207	0.396	-0.984	0.569	0.601	0.946
	LPL	PANSS	-0.604	0.607	-1.794	0.587	0.320	0.946
	LPL	PANSS_P	-0.762	0.487	-1.717	0.192	0.117	0.702
	LPL	PANSS_N	-0.148	0.467	-1.063	0.767	0.751	0.946
	LPL	PANSS_G	-0.493	0.394	-1.264	0.279	0.211	0.908
TC	*APOB*	PANSS	0.269	0.617	-0.941	1.478	0.663	0.946
	APOB	PANSS_P	0.214	0.495	-0.756	1.184	0.666	0.946
	APOB	PANSS_N	-0.539	0.474	-1.469	0.390	0.256	0.922
	APOB	PANSS_G	0.249	0.400	-0.535	1.033	0.534	0.946
	APOC3	PANSS	0.451	0.622	-0.768	1.671	0.468	0.946
	APOC3	PANSS_P	0.117	0.499	-0.861	1.095	0.815	0.946
	APOC3	PANSS_N	0.955	0.478	0.018	1.891	0.046	0.691
	APOC3	PANSS_G	0.040	0.403	-0.750	0.831	0.920	0.981
	LDLR	PANSS	0.083	0.618	-1.130	1.296	0.893	0.981
	LDLR	PANSS_P	0.137	0.496	-0.836	1.109	0.783	0.946
	LDLR	PANSS_N	0.192	0.475	-0.740	1.125	0.686	0.946
	LDLR	PANSS_G	0.107	0.401	-0.680	0.894	0.790	0.946

Note: Adjusted for age, age^2, gender, center, course of disease, previous drug usage, assigned medication, first five principal components;

False discovery rate (FDR)-corrected P-value < 0.05 was applied as the significant threshold accounting for multiple tests across drug targets and outcomes. The conventional P<0.05 was applied as the threshold of suggestive significance;

Abbreviations: PANSS, Positive and Negative Syndrome Scale; N, Negative scale; G, General psychopathology scale; P, Positive scale; LDLC, low density lipoprotein cholesterol; HDLC, high-density lipoprotein cholesterol; TG, triglyceride; TC, total cholesterol; GRS, genetic risk score; CAPOC, Chinese Antipsychotics Pharmacogenomics Consortium.

Table S20. Associations between lipid-lowering drug-target GRSs and better antipsychotic efficacy in the CAPOC study. Related to Figure 3.

Trait	Gene	PANSS	OR	SE_logOR	CI_lower	CI_upper	P	P.FDR
TG	*APOC3*	PANSS	1.084	0.045	0.992	1.185	0.074	0.459
	APOC3	PANSS_P	0.997	0.045	0.913	1.089	0.952	0.952
	APOC3	PANSS_N	1.156	0.045	1.059	1.263	0.001	0.036
	APOC3	PANSS_G	1.044	0.045	0.955	1.141	0.342	0.675
LDLC	*APOB*	PANSS	1.077	0.045	0.986	1.177	0.102	0.459
	APOB	PANSS_P	1.048	0.045	0.959	1.144	0.300	0.675
	APOB	PANSS_N	1.009	0.045	0.924	1.102	0.840	0.889
	APOB	PANSS_G	1.022	0.045	0.936	1.117	0.627	0.837
HDLC	*ABCA1*	PANSS	1.044	0.045	0.956	1.141	0.338	0.675
	ABCA1	PANSS_P	1.016	0.045	0.931	1.109	0.724	0.869
	ABCA1	PANSS_N	1.009	0.045	0.925	1.102	0.836	0.889
	ABCA1	PANSS_G	1.044	0.045	0.956	1.141	0.334	0.675
	CETP	PANSS	0.947	0.045	0.866	1.035	0.226	0.675
	CETP	PANSS_P	0.920	0.045	0.842	1.005	0.064	0.459
	CETP	PANSS_N	0.926	0.045	0.848	1.012	0.089	0.459
	CETP	PANSS_G	0.927	0.045	0.848	1.013	0.096	0.459
	LDLR	PANSS	0.981	0.045	0.899	1.071	0.674	0.867
	LDLR	PANSS_P	1.022	0.044	0.937	1.115	0.628	0.837
	LDLR	PANSS_N	0.945	0.044	0.866	1.030	0.199	0.675
	LDLR	PANSS_G	0.965	0.045	0.884	1.054	0.430	0.744
	LPL	PANSS	0.969	0.044	0.888	1.057	0.478	0.782
	LPL	PANSS_P	0.912	0.044	0.836	0.995	0.038	0.456
	LPL	PANSS_N	0.958	0.044	0.879	1.045	0.334	0.675
	LPL	PANSS_G	0.960	0.044	0.880	1.047	0.356	0.675
TC	*APOB*	PANSS	1.044	0.045	0.955	1.141	0.345	0.675
	APOB	PANSS_P	1.004	0.045	0.919	1.096	0.932	0.952
	APOB	PANSS_N	0.986	0.045	0.903	1.077	0.755	0.877
	APOB	PANSS_G	1.016	0.045	0.930	1.110	0.721	0.869
	APOC3	PANSS	1.053	0.046	0.963	1.152	0.255	0.675
	APOC3	PANSS_P	1.022	0.045	0.935	1.117	0.627	0.837
	APOC3	PANSS_N	1.134	0.045	1.038	1.240	0.005	0.090
	APOC3	PANSS_G	1.012	0.045	0.926	1.107	0.786	0.884
	LDLR	PANSS	0.978	0.045	0.895	1.069	0.624	0.837
	LDLR	PANSS_P	1.036	0.045	0.948	1.131	0.434	0.744
	LDLR	PANSS_N	0.950	0.045	0.870	1.037	0.254	0.675
	LDLR	PANSS_G	0.975	0.045	0.892	1.065	0.568	0.837

Note: Adjusted for age, age^2, gender, center, course of disease, previous drug usage, assigned medication, first five principal components; Better antipsychotic efficacy: PANSS percentage reduction > median value of PANSS percentage reduction; False discovery rate (FDR)-corrected P-value < 0.05 was applied as the significant threshold accounting for multiple tests across drug targets and outcomes. The conventional P<0.05 was applied as the threshold of suggestive significance. The standard error (SE) corresponds to the beta coefficient derived from the natural logarithm of the odds ratio (log OR).

Abbreviations: PANSS, Positive and Negative Syndrome Scale; N, Negative scale; G, General psychopathology scale; P, Positive scale; LDLC, low density lipoprotein cholesterol; HDLC, high-density lipoprotein cholesterol; TG, triglyceride; TC, total cholesterol; GRS, genetic risk score; CAPOC, Chinese Antipsychotics Pharmacogenomics Consortium;

Table S21. Colocalization analysis between GWASs of glucose and PANSS percentage reduction, and pQTL and PANSS percentage reduction of negative symptoms. Related to Figure 3.

Gene locus	Trait1	Trait2	PP.H3	PP.H4	Causal SNP	SNP.PP.H4
APOC3	TG	PANSS_N	0.013	0.094	rs651821	1.000
APOC3	TC	PANSS_N	0.013	0.094	rs651821	1.000
GCK	Glucose	PANSS	0.007	0.027	rs3757840	1.000
GCK	Glucose	PANSS_P	0.005	0.023	rs3757840	1.000
GCK	Glucose	PANSS_N	0.004	0.180	rs3757840	1.000
GCK	Glucose	PANSS_G	0.006	0.025	rs3757840	1.000
APOC3	APOC3 protein	PANSS_N	0.0002	0.002	rs651821	0.715
GCK	GCK protein	PANSS_N	0.00003	0.001	rs3757840	0.689

Notes: Abbreviations: PP.H3, PP.H4, posterior probability of the H3, H4 hypotheses for the Bayesian colocalization analysis; PANSS, Positive and Negative Syndrome Scale; N, Negative scale; G, General psychopathology scale; P, Positive scale.

Table S22. Conventional observational associations between glucose and PANSS percentage reduction in the CAPOC study. Related to Figure 3.

Exposure	PANSS	Beta	SE	CI	P	P.FDR
Glucose	PANSS	-0.086	0.047	-0.179 ~ 0.007	0.070	0.105
	PANSS_P	-0.029	0.038	-0.104 ~ 0.045	0.440	0.440
	PANSS_N	-0.086	0.036	-0.157 ~ -0.014	0.019	0.076
	PANSS_G	-0.054	0.031	-0.115 ~ 0.006	0.079	0.105

Note: Adjusted for age, age^2, gender, center, course of disease, previous drug usage, assigned medication;

False discovery rate (FDR)-corrected P-value < 0.05 was applied as the significant threshold accounting for multiple tests across drug targets and outcomes. The conventional $P<0.05$ was applied as the threshold of suggestive significance.

Abbreviations: PANSS, Positive and Negative Syndrome Scale; N, Negative scale; G, General psychopathology scale; P, Positive scale; CAPOC, Chinese Antipsychotics Pharmacogenomics Consortium.

Table S23. Conventional observational associations between glucose and better antipsychotic efficacy in the CAPOC study. Related to Figure 3.

Exposure	PANSS	OR	SE_logOR	CI	P	P.FDR
Glucose	PANSS	0.998	0.003	0.991 ~ 1.004	0.501	0.501
	PANSS_P	0.997	0.003	0.990 ~ 1.004	0.386	0.501
	PANSS_N	0.995	0.003	0.988 ~ 1.001	0.126	0.501
	PANSS_G	0.997	0.003	0.990 ~ 1.004	0.391	0.501

Note: Adjusted for age, age^2, gender, center, course of disease, previous drug usage, assigned medication;

False discovery rate (FDR)-corrected P-value < 0.05 was applied as the significant threshold accounting for multiple tests across drug targets and outcomes. The conventional $P<0.05$ was applied as the threshold of suggestive significance. Better antipsychotic efficacy: PANSS percentage reduction > median value of PANSS percentage reduction; The standard error (SE) corresponds to the beta coefficient derived from the natural logarithm of the odds ratio (log OR).

Abbreviations: PANSS, Positive and Negative Syndrome Scale; N, Negative scale; G, General psychopathology scale; P, Positive scale; CAPOC, Chinese Antipsychotics Pharmacogenomics Consortium;

Table S24. One-sample Mendelian randomization between glucose and PANSS percentage reduction in the CAPOC study. Related to Figure 3.

Exposure	PANSS	Beta	SE	CI	P	P.FDR
Glucose	PANSS	0.785	0.323	0.152 ~ 1.418	0.015	0.025
	PANSS_P	0.585	0.259	0.077 ~ 1.092	0.024	0.025
	PANSS_N	0.642	0.248	0.156 ~ 1.128	0.010	0.025
	PANSS_G	0.468	0.209	0.058 ~ 0.879	0.025	0.025

Note: Adjusted for age, age^2, gender, center, course of disease, previous drug usage, assigned medication, first five principal components;

False discovery rate (FDR)-corrected P-value < 0.05 was applied as the significant threshold accounting for multiple tests across drug targets and outcomes. The conventional P<0.05 was applied as the threshold of suggestive significance.

Abbreviations: PANSS, Positive and Negative Syndrome Scale; N, Negative scale; G, General psychopathology scale; P, Positive scale; SE, standard error; CAPOC, Chinese Antipsychotics Pharmacogenomics Consortium.

Table S25. One-sample Mendelian randomization between glucose and better antipsychotic efficacy in the CAPOC study. Related to Figure 3.

Exposure	PANSS	OR	SE_logOR	CI	P	P.FDR
Glucose	PANSS	1.054	0.024	1.006 ~ 1.104	0.027	0.054
	PANSS_P	1.015	0.024	0.969 ~ 1.063	0.523	0.523
	PANSS_N	1.060	0.024	1.013 ~ 1.111	0.013	0.052
	PANSS_G	1.048	0.024	1.000 ~ 1.098	0.049	0.065

Note: Adjusted for age, age^2, gender, center, course of disease, previous drug usage, assigned medication, first five principal components;

False discovery rate (FDR)-corrected P-value < 0.05 was applied as the significant threshold accounting for multiple tests across drug targets and outcomes. The conventional P<0.05 was applied as the threshold of suggestive significance.

Better antipsychotic efficacy: PANSS percentage reduction > median value of PANSS percentage reduction; The standard error (SE) corresponds to the beta coefficient derived from the natural logarithm of the odds ratio (log OR).

Abbreviations: PANSS, Positive and Negative Syndrome Scale; N, Negative scale; G, General psychopathology scale; P, Positive scale; SE, standard error; CAPOC, Chinese Antipsychotics Pharmacogenomics Consortium.

Table S26. Associations between antidiabetic drug-target GRSs and PANSS percentage reduction in the CAPOC study. Related to Figure 3.

Trait	Gene	PANSS	Beta	SE	CI_lower	CI_upper	P	P.FDR
Glucose	*GCK*	PANSS	-1.703	0.614	-2.907	-0.499	0.006	0.016
	GCK	PANSS_P	-1.055	0.493	-2.021	-0.089	0.032	0.064
	GCK	PANSS_N	-1.621	0.472	-2.546	-0.696	0.001	0.008
	GCK	PANSS_G	-1.119	0.398	-1.900	-0.339	0.005	0.016
	ABCB11	PANSS	0.200	0.617	-1.009	1.410	0.745	0.851
	ABCB11	PANSS_P	-0.318	0.495	-1.287	0.652	0.521	0.695
	ABCB11	PANSS_N	0.691	0.474	-0.238	1.621	0.145	0.232
	ABCB11	PANSS_G	-0.023	0.400	-0.807	0.761	0.954	0.954

Note: Adjusted for age, age^2, gender, center, course of disease, previous drug usage, assigned medication, first five principal components;

False discovery rate (FDR)-corrected P-value < 0.05 was applied as the significant threshold accounting for multiple tests across drug targets and outcomes. The conventional P<0.05 was applied as the threshold of suggestive significance.

Abbreviations: PANSS, Positive and Negative Syndrome Scale; N, Negative scale; G, General psychopathology scale; P, Positive scale; SE, standard error; GRS, genetic risk score; CAPOC, Chinese Antipsychotics Pharmacogenomics Consortium.

Table S27. Associations between antidiabetic drug-target GRSs and better antipsychotic efficacy in the CAPOC study. Related to Figure 3.

Trait	Gene	PANSS	OR	SE_logOR	CI_lower	CI_upper	P	P.FDR
Glucose	*GCK*	PANSS	0.917	0.045	0.839	1.002	0.056	0.112
	GCK	PANSS_P	0.896	0.045	0.820	0.978	0.014	0.084
	GCK	PANSS_N	0.901	0.045	0.825	0.984	0.021	0.084
	GCK	PANSS_G	0.912	0.045	0.834	0.996	0.041	0.109
	ABCB11	PANSS	0.987	0.045	0.903	1.078	0.772	0.882
	ABCB11	PANSS_P	0.961	0.045	0.880	1.049	0.376	0.501
	ABCB11	PANSS_N	1.003	0.045	0.919	1.095	0.939	0.939
	ABCB11	PANSS_G	0.934	0.045	0.855	1.020	0.129	0.206

Note: Adjusted for age, age^2, gender, center, course of disease, previous drug usage, assigned medication, first five principal components;

Better antipsychotic efficacy: PANSS percentage reduction > median value of PANSS percentage reduction;

The standard error (SE) corresponds to the beta coefficient derived from the natural logarithm of the odds ratio (log OR).

False discovery rate (FDR)-corrected P-value < 0.05 was applied as the significant threshold accounting for multiple tests across drug targets and outcomes. The conventional P<0.05 was applied as the threshold of suggestive significance.

Abbreviations: PANSS, Positive and Negative Syndrome Scale; N, Negative scale; G, General psychopathology scale; P, Positive scale; SE, standard error; GRS, genetic risk score; CAPOC, Chinese Antipsychotics Pharmacogenomics Consortium.

Table S28. Replication analysis for associations between *APOC3* GRS and PANSS percentage reduction in the CAPEC study. Related to Figure 3.

Gene	Trait	PANSS	Beta	SE	CI_lower	CI_upper	P	P.FDR
APOC3	TG	PANSS	0.505	1.399	-2.250	3.261	0.718	0.927
		PANSS_P	-0.309	1.188	-2.649	2.031	0.795	0.927
		PANSS_N	1.186	1.151	-1.081	3.452	0.304	0.927
		PANSS_G	0.230	1.015	-1.769	2.228	0.821	0.927
APOC3	TC	PANSS	0.133	1.450	-2.722	2.989	0.927	0.927
		PANSS_P	-0.754	1.231	-3.177	1.669	0.541	0.927
		PANSS_N	1.578	1.191	-0.768	3.923	0.186	0.927
		PANSS_G	-0.174	1.052	-2.245	1.897	0.869	0.927

Note: Adjusted for age, age^2, gender, center, course of disease, previous drug usage, assigned medication, first five principal components;

False discovery rate (FDR)-corrected P-value < 0.05 was applied as the significant threshold accounting for multiple tests across drug targets and outcomes. The conventional P<0.05 was applied as the threshold of suggestive significance.

Abbreviations: PANSS, Positive and Negative Syndrome Scale; N, Negative scale; G, General psychopathology scale; P, Positive scale; SE, standard error; GRS, genetic risk score; TG, triglyceride; TC, total cholesterol; CAPEC, Chinese Antipsychotics Pharmacogenetics Consortium.

Table S29. Replication analysis for associations between *GCK* GRS and PANSS percentage reduction in the CAPEC study. Related to Figure 3.

Gene	Trait	PANSS	Beta	SE	CI_lower	CI_upper	P	P.FDR
GCK	Glucose	PANSS	-3.200	1.327	-5.813	-0.587	0.017	0.034
		PANSS_P	-2.272	1.131	-4.498	-0.045	0.046	0.061
		PANSS_N	-2.706	1.093	-4.858	-0.554	0.014	0.034
		PANSS_G	-1.732	0.967	-3.636	0.173	0.075	0.075

Note: Adjusted for age, age^2, gender, center, course of disease, previous drug usage, assigned medication, first five principal components; False discovery rate (FDR)-corrected P-value < 0.05 was applied as the significant threshold accounting for multiple tests across drug targets and outcomes. The conventional P<0.05 was applied as the threshold of suggestive significance.

Abbreviations: PANSS, Positive and Negative Syndrome Scale; N, Negative scale; G, General psychopathology scale; P, Positive scale; SE, standard error; GRS, genetic risk score; CAPEC, Chinese Antipsychotics Pharmacogenetics Consortium.

Table S30. Replication analysis for associations between *APOC3* GRSs and better antipsychotic efficacy in the CAPEC study. Related to Figure 3.

Gene	Trait	PANSS	OR	SE_logOR	CI_lower	CI_upper	P	P.FDR
APOC3	TG	PANSS	1.090	0.131	0.844	1.414	0.510	0.808
		PANSS_P	0.959	0.128	0.745	1.233	0.743	0.811
		PANSS_N	1.208	0.133	0.933	1.573	0.155	0.740
		PANSS_G	0.886	0.129	0.687	1.141	0.350	0.808
APOC3	TC	PANSS	1.072	0.135	0.824	1.401	0.606	0.808
		PANSS_P	0.969	0.133	0.746	1.258	0.811	0.811
		PANSS_N	1.200	0.137	0.918	1.578	0.185	0.740
		PANSS_G	0.900	0.133	0.692	1.168	0.430	0.808

Note: Adjusted for age, age^2, gender, center, course of disease, previous drug usage, assigned medication, first five principal components;

Better antipsychotic efficacy: PANSS percentage reduction > median value of PANSS percentage reduction;

The standard error (SE) corresponds to the beta coefficient derived from the natural logarithm of the odds ratio (log OR).

False discovery rate (FDR)-corrected P-value < 0.05 was applied as the significant threshold accounting for multiple tests across drug targets and outcomes. The conventional P<0.05 was applied as the threshold of suggestive significance.

Abbreviations: PANSS, Positive and Negative Syndrome Scale; N, Negative scale; G, General psychopathology scale; P, Positive scale; SE, standard error; GRS, genetic risk score; TG, triglyceride; TC, total cholesterol; CAPEC, Chinese Antipsychotics Pharmacogenetics Consortium.

Table S31. Replication analysis for associations between *GCK* GRS and better antipsychotic efficacy in the CAPEC study. Related to Figure 3.

Gene	Trait	PANSS	OR	SE_logOR	CI_lower	CI_upper	P	P.FDR
GCK	Glucose	PANSS	0.735	0.127	0.570	0.941	0.016	0.032
		PANSS_P	0.760	0.124	0.593	0.967	0.027	0.036
		PANSS_N	0.686	0.130	0.528	0.882	0.004	0.016
		PANSS_G	0.831	0.124	0.649	1.058	0.135	0.135

Note: Adjusted for age, age^2, gender, center, course of disease, previous drug usage, assigned medication, first five principal components; Better antipsychotic efficacy: PANSS percentage reduction > median value of PANSS percentage reduction;

The standard error (SE) corresponds to the beta coefficient derived from the natural logarithm of the odds ratio (log OR).

False discovery rate (FDR)-corrected P-value < 0.05 was applied as the significant threshold accounting for multiple tests across drug targets and outcomes. The conventional P<0.05 was applied as the threshold of suggestive significance.

Abbreviations: PANSS, Positive and Negative Syndrome Scale; N, Negative scale; G, General psychopathology scale; P, Positive scale; SE, standard error; GRS, genetic risk score; CAPEC, Chinese Antipsychotics Pharmacogenetics Consortium.

Table S32. Replication analysis via two-sample MR methods for associations between *APOC3*-lowering TG, *GCK*-lowering glucose on the PANSS percentage reduction. Related to Figure 3.

Exposure	PANSS	Method	nSNP	Beta	SE	CI_Lower	CI_Upper	P	P.FDR
**Main analysis-Inverse variance weighted**									
*APOC3*_TG	PANSS	Inverse variance weighted	13	3.358	2.561	-1.662	8.378	0.1898	0.3254
	PANSS_P	Inverse variance weighted	13	0.642	2.111	-3.496	4.779	0.7612	0.8459
	PANSS_N	Inverse variance weighted	13	6.680	1.968	2.822	10.538	0.0007	0.0021
	PANSS_G	Inverse variance weighted	13	1.239	1.661	-2.017	4.495	0.4556	0.6075
*APOC3*_TC	PANSS	Inverse variance weighted	4	10.108	11.204	-11.853	32.068	0.3670	0.5505
	PANSS_P	Inverse variance weighted	4	2.564	8.987	-15.051	20.179	0.7754	0.8459
	PANSS_N	Inverse variance weighted	4	21.374	8.611	4.497	38.251	0.0131	0.0262
	PANSS_G	Inverse variance weighted	4	0.944	7.931	-14.601	16.488	0.9053	0.9053
*GCK*_Glucose	PANSS	Inverse variance weighted	6	-20.419	5.684	-31.560	-9.279	0.0003	0.0012
	PANSS_P	Inverse variance weighted	6	-12.656	4.560	-21.594	-3.717	0.0055	0.0132
	PANSS_N	Inverse variance weighted	6	-19.481	4.370	-28.045	-10.917	8.26E-06	0.0001
	PANSS_G	Inverse variance weighted	6	-13.630	3.688	-20.858	-6.401	0.0002	0.0012
**Sensitivity analysis - Weighted Median**									
**Exposure**	**PANSS**	**Method**	**nSNP**	**Beta**	**SE**	**CI_Lower**	**CI_Upper**	**P**	**P.FDR**
*APOC3*_TG	PANSS	Weighted median	13	1.901	2.956	-3.892	7.694	0.5201	0.7802
	PANSS_P	Weighted median	13	-0.018	2.433	-4.788	4.752	0.9941	0.9941
	PANSS_N	Weighted median	13	4.889	2.245	0.489	9.289	0.0294	0.0588
	PANSS_G	Weighted median	13	0.576	2.044	-3.431	4.582	0.7783	0.9449
*APOC3*_TC	PANSS	Weighted median	4	9.442	13.376	-16.774	35.658	0.4802	0.7802
	PANSS_P	Weighted median	4	2.720	10.087	-17.050	22.490	0.7874	0.9449
	PANSS_N	Weighted median	4	23.627	9.968	4.090	43.165	0.0178	0.0427
	PANSS_G	Weighted median	4	0.461	8.625	-16.444	17.367	0.9573	0.9941
*GCK*_Glucose	PANSS	Weighted median	6	-21.700	6.770	-34.970	-8.430	0.0013	0.0052
	PANSS_P	Weighted median	6	-15.874	5.388	-26.434	-5.313	0.0032	0.0096
	PANSS_N	Weighted median	6	-21.683	5.448	-32.361	-11.006	0.0001	0.0012
	PANSS_G	Weighted median	6	-15.437	4.433	-24.125	-6.749	0.0005	0.0030

Note: False discovery rate (FDR)-corrected P-value < 0.05 was applied as the significant threshold accounting for multiple tests across drug targets and outcomes. The conventional P<0.05 was applied as the threshold of suggestive significance. Abbreviations: PANSS, Positive and Negative Syndrome Scale; N, Negative scale; G, General psychopathology scale; P, Positive scale; SE, standard error; GRS, genetic risk score; TG, triglyceride; TC, total cholesterol.

Table S33. Test for the replication analysis via two-sample MR methods for associations between *APOC3*-lowering TG, *GCK*-lowering glucose on the PANSS percentage reduction. Related to Figure 3.

Exposure	PANSS	Egger.inter	Egger.SE	P.inter	MR-PRESSO Global test.P	MR-PRESSO N_outlier	*Q*	P.heterogeneity
*APOC3*_TG	PANSS	0.107	0.630	0.868	0.552	0	11.763	0.465
	PANSS_P	-0.143	0.523	0.789	0.728	0	12.673	0.393
	PANSS_N	0.581	0.469	0.241	0.547	0	7.126	0.849
	PANSS_G	-0.078	0.407	0.850	0.608	0	11.659	0.473
*APOC3*_TC	PANSS	-0.739	1.445	0.660	0.431	0	1.471	0.689
	PANSS_P	-0.473	1.159	0.723	0.805	0	0.764	0.858
	PANSS_N	-1.021	1.111	0.455	0.888	0	1.333	0.721
	PANSS_G	-0.889	1.083	0.498	0.762	0	3.572	0.312
*GCK*_Glucose	PANSS	0.137	1.088	0.906	0.837	0	2.556	0.768
	PANSS_P	0.133	0.873	0.886	0.742	0	1.923	0.860
	PANSS_N	0.012	0.837	0.990	0.845	0	2.568	0.766
	PANSS_G	-0.194	0.705	0.796	0.798	0	2.143	0.829

Note: A test for intercepts of MR-Egger (ME) regression was used to indicate the degree of directional horizontal pleiotropy. MR-Egger regression was performed to test for horizontal pleiotropy. Mendelian randomization pleiotropy residual sum and outlier (MR-PRESSO) was applied. SNPs that were detected to be the outliers (P < 0.05) were excluded to minimize pleiotropy bias. Inverse variance weighted Cochrane's *Q* test was performed for the test of heterogeneity.
Abbreviations: PANSS, Positive and Negative Syndrome Scale; N, Negative scale; G, General psychopathology scale; P, Positive scale; SE, standard error; GRS, genetic risk score; TG, triglyceride; TC, total cholesterol.

Table S34. Associations between lipid- and glucose-lowering target pQTL-GRSs and levels of measured lipid and glucose in the CAPOC study. Related to Figure 3.

Gene	Base sample	Trait	Beta	SE	CI_lower	CI_upper	P	P.FDR
APOC3	GNHS-pQTL in blood	TG	-6.010	1.115	-8.195	-3.825	7.65E-08	2.30E-07
APOC3	GNHS-pQTL in blood	TC	-1.821	0.712	-3.217	-0.426	0.011	0.011
GCK	Multi-cohorts-pQTL in CSF	Glucose	-0.748	0.283	-1.303	-0.193	0.008	0.011

Note: Adjusted for age, age^2, gender, center, first five principal components, course of disease, previous drug usage;

False discovery rate (FDR)-corrected P-value < 0.05 was applied as the significant threshold accounting for multiple tests across drug targets, lipids, and glucose. The conventional P<0.05 was applied as the threshold of suggestive significance.

Abbreviations: SE, standard error; pQTL, protein quantitative trait loci; CSF, cerebrospinal fluid; GNHS, Guangzhou Nutrition and Health study; GRS, genetic risk score; CAPOC, Chinese Antipsychotics Pharmacogenomics Consortium.

Table S35. Associations between APOC3 blood-pQTL-GRSs and PANSS percentage reduction in the CAPOC study. Related to Figure 3.

PANSS percentage reduction	Beta	SE	CI_lower	CI_upper	P	P.FDR
PANSS	0.381	0.615	-0.825	1.586	0.536	0.948
PANSS_P	0.032	0.494	-0.936	1.000	0.948	0.948
PANSS_N	0.877	0.471	-0.047	1.802	0.063	0.252
PANSS_G	4.11E-02	0.398	-0.740	0.822	0.918	0.948

Note: Adjusted for age, age^2, gender, center, first five principal components, course of disease, previous drug usage, assigned medication; False discovery rate (FDR)-corrected P-value < 0.05 was applied as the significant threshold accounting for multiple tests across drug targets and outcomes. The conventional P<0.05 was applied as the threshold of suggestive significance.

Abbreviations: PANSS, Positive and Negative Syndrome Scale; N, Negative scale; G, General psychopathology scale; P, Positive scale; SE, standard error; GRS, genetic risk score; pQTL, protein quantitative trait loci; CAPOC, Chinese Antipsychotics Pharmacogenomics Consortium.

Table S36. Associations between APOC3 blood-pQTL-GRSs and better antipsychotic efficacy in the CAPOC study. Related to Figure 3.

PANSS percentage reduction	OR	SE_logOR	OR_lower	OR_upper	P	P.FDR
PANSS	1.069	0.045	0.979	1.167	0.138	0.276
PANSS_P	0.989	0.044	0.906	1.079	0.800	0.800
PANSS_N	1.113	0.045	1.020	1.215	0.016	0.064
PANSS_G	1.013	0.045	0.928	1.106	0.767	0.800

Note: Adjusted for age, age^2, gender, center, first five principal components, course of disease, previous drug usage, assigned medication;

Better antipsychotic efficacy: PANSS percentage reduction > median value of PANSS percentage reduction;

The standard error (SE) corresponds to the beta coefficient derived from the natural logarithm of the odds ratio (log OR).

False discovery rate (FDR)-corrected P-value < 0.05 was applied as the significant threshold accounting for multiple tests across drug targets and outcomes. The conventional P<0.05 was applied as the threshold of suggestive significance.

Abbreviations: PANSS, Positive and Negative Syndrome Scale; N, Negative scale; G, General psychopathology scale; P, Positive scale; SE, standard error; GRS, genetic risk score; pQTL, protein quantitative trait loci; CAPOC, Chinese Antipsychotics Pharmacogenomics Consortium.

Table S37. Associations between GCK CSF-pQTL-GRSs and PANSS percentage reduction in the CAPOC study. Related to Figure 3.

PANSS	Beta	SE	CI	P	P.FDR
PANSS	-0.509	0.616	-1.716 ~ 0.699	0.409	0.579
PANSS_P	-0.274	0.494	-1.243 ~ 0.694	0.579	0.579
PANSS_N	-1.058	0.473	-1.986 ~ -0.130	0.025	0.102
PANSS_G	-0.298	0.400	-1.081 ~ 0.486	0.456	0.579

Note: Adjusted for age, age^2, gender, center, first five principal components, course of disease, previous drug usage, assigned medication;

False discovery rate (FDR)-corrected P-value < 0.05 was applied as the significant threshold accounting for multiple tests across drug targets and outcomes. The conventional P<0.05 was applied as the threshold of suggestive significance.

Abbreviations: PANSS, Positive and Negative Syndrome Scale; N, Negative scale; G, General psychopathology scale; P, Positive scale; SE, standard error; GRS, genetic risk score; pQTL, protein quantitative trait loci; CSF, cerebrospinal fluid; CAPOC, Chinese Antipsychotics Pharmacogenomics Consortium.

Table S38. Associations between GCK CSF-pQTL-GRSs and better antipsychotic efficacy in the CAPOC study. Related to Figure 3.

PANSS	OR	SE_logOR	CI	P	P.FDR
PANSS	0.966	0.045	0.884 ~ 1.055	0.437	0.583
PANSS_P	0.955	0.045	0.874 ~ 1.042	0.300	0.583
PANSS_N	0.941	0.045	0.862 ~ 1.027	0.171	0.583
PANSS_G	1.019	0.045	0.933 ~ 1.113	0.680	0.680

Note: Adjusted for age, age^2, gender, center, first five principal components, course of disease, previous drug usage, assigned medication;

Better antipsychotic efficacy: PANSS percentage reduction > median value of PANSS percentage reduction; The standard error (SE) corresponds to the beta coefficient derived from the natural logarithm of the odds ratio (log OR). False discovery rate (FDR)-corrected P-value < 0.05 was applied as the significant threshold accounting for multiple tests across drug targets and outcomes. The conventional P<0.05 was applied as the threshold of suggestive significance.

Abbreviations: PANSS, Positive and Negative Syndrome Scale; N, Negative scale; G, General psychopathology scale; P, Positive scale; SE, standard error; GRS, genetic risk score; pQTL, protein quantitative trait loci; CSF, cerebrospinal fluid; CAPOC, Chinese Antipsychotics Pharmacogenomics Consortium.

Table S39. Mediation analysis between pQTL-GRS, lipids and glucose, and the antipsychotic efficacy. Related to Figure 3.

Term	OR	SE_logOR*	95%CI_Lower	95%CI_Upper	P
**APOC3 pQTL associated with better antipsychotic efficacy of PANSS_N**					
pQTL-weighted APOC3 score (per 1 unit lower APOC3 protein levels)	1.103	0.044	1.012	1.202	0.026
**APOC3 pQTL associated with better antipsychotic efficacy of PANSS_N, mediated through TG**					
pQTL-weighted APOC3 score (per 1 unit lower APOC3 protein levels)	0.880	0.105	0.716	1.081	0.222
TG-weighted APOC3 score (per 1 unit lower TG through APOC3)	1.299	0.106	1.055	1.598	0.014
**APOC3 pQTL associated with better antipsychotic efficacy of PANSS_N, mediated through TC**					
pQTL-weighted APOC3 score (per 1 unit lower APOC3 protein levels)	1.041	0.066	0.915	1.185	0.540
TC-weighted APOC3 score (per 1 unit lower TC through APOC3)	1.096	0.067	0.962	1.248	0.170

Term	Beta	SE	95%CI_Lower	95%CI_Upper	P
**GCK pQTL associated with decreased percentage reduction of PANSS_N**					
pQTL-weighted GCK score (per 1 unit lower GCK protein levels)	-1.058	0.473	-1.986	-0.130	0.025
**GCK pQTL associated with decreased percentage reduction of PANSS_N, mediated through glucose**					
pQTL-weighted GCK score (per 1 unit lower GCK protein levels)	-0.498	0.513	-1.505	0.508	0.332
Glucose-weighted GCK score (per 1 unit lower glucose through GCK)	-1.426	0.513	-2.431	-0.421	0.005

Note: Adjusted for age, age^2, gender, center, first five principal components, course of disease, previous drug usage, assigned medication;

Better antipsychotic efficacy: PANSS percentage reduction > median value of PANSS percentage reduction; The standard error (SE) corresponds to the beta coefficient derived from the natural logarithm of the odds ratio (log OR).

Abbreviations: PANSS, Positive and Negative Syndrome Scale; N, Negative scale; G, General psychopathology scale; P, Positive scale; SE, standard error; GRS, genetic risk score; pQTL, protein quantitative trait loci.

Table S40. Bulk tissue gene expression (median TPM) for *APOC3* and *GCK*. Related to Figure 3.

Gene	Pancreas	Liver	Adipose - Visceral (Omentum)	Pituitary	Muscle - Skeletal	Brain - Cortex (BA9)
GCK	0.511	0.857	0.425	28.56	0.033	2.125
APOC3	0.351	8122	0.302	0.395	0.035	0.083
**Gene**	**Brain - Caudate (basal ganglia)**	**Brain - Nucleus accumbens (basal ganglia)**	**Brain - Cerebellum**	**Brain - Hypothalamus**	**Small Intestine - Terminal Ileum**	**Artery - Coronary**
GCK	1.248	1.887	6.077	5.484	0.280	1.230
APOC3	0.036	0	0.371	0	112	0.198

Note: Based on GTEx v10 database; TPM, Transcripts Per Million.

Table S41. Associations between *APOC3, GCK* and neural, metabolic-related traits. Related to Figure 3.

**Gene**	**Top SNP**	**EA of lipid- or glucose-lowering**	**T2D_Beta**	**T2D_SE**	**T2D_P**	**2h_OGTT_ Beta**	**2h_OGTT_SE**	**2h_OGTT_P**
GCK	rs2908289	G	-0.017	0.008	0.035	-0.068	0.016	3.74E-05
APOC3	rs651821	T	-0.006	0.007	0.375	0.021	0.014	0.129
**Gene**	**Top SNP**	**EA of lipid- or glucose-lowering**	**HbA1C_ Beta**	**HbA1C_SE**	**HbA1C_P**	**Insulin_Beta**	**Insulin_SE**	**Insulin_P**
GCK	rs2908289	G	-0.097	0.005	1.91E-74	-0.002	0.006	0.777
APOC3	rs651821	T	0.006	0.005	0.204	-0.002	0.005	0.599
**Gene**	**Top SNP**	**EA of lipid- or glucose-lowering**	**BMI_Beta**	**BMI_SE**	**BMI_P**	**CAD_Beta**	**CAD_SE**	**CAD_P**
GCK	rs2908289	G	0.004	0.005	0.400	-0.026	0.013	0.041
APOC3	rs651821	T	0.012	0.004	0.001	-0.059	0.010	1.21E-08
**Gene**	**Top SNP**	**EA of lipid- or glucose-lowering**	**MetS_Beta**	**MetS_SE**	**MetS_P**	**Hyperlipidemia_ Beta**	**Hyperlipidemia_ SE**	**Hyperlipidemia_P**
GCK	rs2908289	G	-0.042	0.019	0.027	-0.022	0.008	0.001
APOC3	rs651821	T	-0.367	0.016	6.72E-116	-0.208	0.012	4.37E-86
**Gene**	**Top SNP**	**EA of lipid- or glucose-lowering**	**Sphingomyelin_ Beta**	**Sphingomyelin_SE**	**Sphingomyelin_P**	**Beta_ Endorphin_ Beta**	**Beta_ Endorphin_SE**	**Beta_ Endorphin_P**
GCK	rs2908289	G	0.014	0.005	0.004	0.007	0.030	0.819
APOC3	rs651821	T	0.039	0.008	1.80E-06	-0.442	0.040	2.24E-28
**Gene**	**Top SNP**	**EA of lipid- or glucose-lowering**	**SCZ_Beta**	**SCZ_SE**	**SCZ_P**			
GCK	rs2908289	G	0.143	0.186	0.443			
APOC3	rs651821	T	0.328	0.151	0.030			

Notes: The effect size referred to the association between lipid- or glucose-lowering allele and the corresponding trait. Abbreviations: T2D, type 2 diabetes; 2h OGTT, 2-Hour Oral Glucose Tolerance Test; HbA1c, Hemoglobin A1c; BMI, body mass index; CAD, coronary artery disease; MetS, metabolic syndrome; SCZ, schizophrenia; EA, effect allele.

Table S42. Associations between *APOC3, GCK* GRSs with laboratory and physical examination measures in the CAPOC study. Related to Figure 3.

GRS	Outcome	Beta	SE	95%CI	P	P.FDR
APOC3_TG	SBP (mmHg)	-0.405	0.237	-0.868 ~ 0.059	0.087	0.316
	DBP (mmHg)	-0.280	0.173	-0.619 ~ 0.059	0.106	0.316
	Pulse (/min)	-0.065	0.222	-0.500 ~ 0.370	0.770	0.866
	ALT (IU/L)	0.815	0.382	0.067 ~ 1.563	0.033	0.316
	AST (IU/L)	0.232	0.301	-0.357 ~ 0.822	0.440	0.625
	BUN (mmol/L)	-0.644	0.518	-1.659 ~ 0.371	0.214	0.480
	CRE (mmol/L)	-0.155	0.483	-1.103 ~ 0.792	0.748	0.866
	PRL (uIU/mL)	-3.260	27.243	-56.657 ~ 50.136	0.905	0.953
	QTc interval (ms)	-1.148	0.650	-2.422 ~ 0.126	0.078	0.316
APOC3_TC	SBP (mmHg)	-0.154	0.238	-0.621 ~ 0.312	0.517	0.698
	DBP (mmHg)	-0.144	0.174	-0.485 ~ 0.197	0.407	0.625
	Pulse (/min)	-0.219	0.223	-0.657 ~ 0.218	0.325	0.585
	ALT (IU/L)	0.950	0.384	0.198 ~ 1.702	0.013	0.316
	AST (IU/L)	0.544	0.302	-0.049 ~ 1.136	0.072	0.316
	BUN (mmol/L)	-0.872	0.521	-1.892 ~ 0.148	0.094	0.316
	CRE (mmol/L)	-0.029	0.486	-0.981 ~ 0.924	0.953	0.953
	PRL (uIU/mL)	-42.384	26.986	-95.277 ~ 10.509	0.117	0.316
	QTc interval (ms)	-0.832	0.652	-2.110 ~ 0.447	0.202	0.480
*GCK*_Glucose	SBP (mmHg)	-0.114	0.236	-0.576 ~ 0.349	0.630	0.810
	DBP (mmHg)	0.015	0.173	-0.323 ~ 0.353	0.930	0.953
	Pulse (/min)	0.265	0.221	-0.168 ~ 0.698	0.231	0.480
	ALT (IU/L)	-0.606	0.381	-1.352 ~ 0.140	0.111	0.316
	AST (IU/L)	-0.316	0.300	-0.904 ~ 0.272	0.292	0.563
	BUN (mmol/L)	-0.813	0.517	-1.827 ~ 0.200	0.116	0.316
	CRE (mmol/L)	-0.177	0.483	-1.123 ~ 0.769	0.714	0.866
	PRL (uIU/mL)	24.903	27.285	-28.575 ~ 78.382	0.362	0.611
	QTc interval (ms)	0.518	0.650	-0.755 ~ 1.791	0.425	0.625

Notes: Adjusted for age, age^2, gender, center, first five principal components, course of disease, previous drug usage.

False discovery rate (FDR)-corrected P-value < 0.05 was applied as the significant threshold accounting for multiple tests across drug targets and outcomes. The conventional P<0.05 was applied as the threshold of suggestive significance.

Abbreviations: SBP, systolic blood pressure; DBP, diastolic blood pressure; BMI, body mass index; QTc, corrected QT interval; ALT, alanine aminotransferase; AST, aspartate aminotransferase; BUN, blood urea nitrogen; CRE, creatinine; PRL, prolactin; HbA1c, Glycated hemoglobin A1c; SE, standard error; CAPOC, Chinese Antipsychotics Pharmacogenomics Consortium.

Table S45. Replication for the subgroup associations between *GCK* GRS and PANSS percentage reduction in the CAPEC study. Related to Figure 4.

PANSS	First episode or recurrent													
	First episode (N=94)						Recurrent (N=198)						P.inter	P.inter.FDR
	Beta	SE	CI_lower	CI_upper	P	P.FDR	Beta	SE	CI_lower	CI_upper	P	P.FDR		
PANSS	-5.705	2.675	-11.042	-0.368	0.037	0.074	-1.885	1.528	-4.901	1.131	0.219	0.397	0.194	0.260
PANSS_P	-4.297	2.363	-9.011	0.417	0.073	0.092	-1.141	1.274	-3.655	1.374	0.372	0.397	0.217	0.260
PANSS_N	-5.398	2.294	-9.973	-0.822	0.021	0.074	-1.507	1.226	-3.927	0.913	0.221	0.397	0.127	0.260
PANSS_G	-3.323	1.944	-7.201	0.555	0.092	0.092	-0.946	1.113	-3.144	1.252	0.397	0.397	0.260	0.260

PANSS	Antipsychotics with stronger or milder metabolic effects													
	Stronger (N=124)						Milder (N=168)						P.inter	P.inter.FDR
	Beta	SE	CI_lower	CI_upper	P	P.FDR	Beta	SE	CI_lower	CI_upper	P	P.FDR		
PANSS	-3.520	2.081	-7.645	0.605	0.094	0.231	-2.535	1.724	-5.942	0.872	0.144	0.231	0.690	0.992
PANSS_P	-2.239	1.858	-5.921	1.444	0.231	0.231	-2.008	1.465	-4.904	0.888	0.173	0.231	0.744	0.992
PANSS_N	-2.946	1.896	-6.706	0.813	0.123	0.231	-2.494	1.394	-5.248	0.261	0.076	0.231	0.996	0.996
PANSS_G	-2.181	1.648	-5.448	1.087	0.189	0.231	-1.163	1.214	-3.562	1.237	0.340	0.340	0.625	0.992

PANSS	Baseline diabetes status													
	With prediabetics or diabetes (N=44)						Without prediabetes or diabetes (N=248)						P.inter	P.inter.FDR
	Beta	SE	CI_lower	CI_upper	P	P.FDR	Beta	SE	CI_lower	CI_upper	P	P.FDR		
PANSS	-7.351	4.058	-15.664	0.963	0.081	0.156	-3.048	1.414	-5.836	-0.261	0.032	0.064	0.401	0.748
PANSS_P	-4.421	3.058	-10.685	1.844	0.159	0.159	-2.359	1.244	-4.812	0.093	0.059	0.079	0.830	0.830
PANSS_N	-4.985	3.084	-11.302	1.332	0.117	0.156	-2.682	1.186	-5.019	-0.345	0.025	0.064	0.561	0.748
PANSS_G	-5.919	3.213	-12.501	0.663	0.076	0.156	-1.401	1.025	-3.422	0.620	0.173	0.173	0.191	0.748

PANSS	Gender													
	Male (N=132)						Female (N=160)						P.inter	P.inter.FDR
	Beta	SE	CI_lower	CI_upper	P	P.FDR	Beta	SE	CI_lower	CI_upper	P	P.FDR		
PANSS	-4.506	1.998	-8.468	-0.545	0.026	0.045	-0.955	1.790	-4.494	2.583	0.594	0.792	0.323	0.646
PANSS_P	-3.746	1.643	-7.003	-0.489	0.025	0.045	-0.073	1.559	-3.155	3.010	0.963	0.963	0.165	0.646
PANSS_N	-3.423	1.595	-6.584	-0.261	0.034	0.045	-1.507	1.523	-4.518	1.503	0.324	0.792	0.676	0.901
PANSS_G	-1.698	1.425	-4.522	1.127	0.236	0.236	-1.083	1.359	-3.770	1.604	0.427	0.792	0.952	0.952

Note: Adjusted for age, age^2, gender, center, first five principal components, course of disease, previous drug usage, assigned medication;

Patients with baseline glucose >= 100.8 mg/dL (5.6 mmol/L) were classified into the prediabetes or diabetes group, the remaining patients were in the non-diabetes or prediabetes group;

Individuals with < 2 years of first-episode SCZ course, and < 14 days of antipsychotic exposure, were classified as first-episode, drug-naïve. The remaining patients were categorized as recurrent. Patients prescribed risperidone, olanzapine, quetiapine, or clozapine were grouped as receiving medication with stronger metabolic side effects; Others (prescribing aripiprazole, ziprasidone, or perphenazine) comprised the milder-metabolic-effect group.

False discovery rate (FDR)-corrected P-value < 0.05 was applied as the significant threshold accounting for multiple tests across drug targets and outcomes. The conventional P<0.05 was applied as the threshold of suggestive significance.

Abbreviations: PANSS, Positive and Negative Syndrome Scale; N, Negative scale; G, General psychopathology scale; P, Positive scale; SE, standard error; GRS, genetic risk score; CAPEC, Chinese Antipsychotics Pharmacogenetics Consortium.

Table S46. Replication for the subgroup associations between *GCK* GRS and better antipsychotic efficacy in the CAPEC study. Related to Figure 4.

First episode or recurrent														
PANSS	First episode (N=94)						Recurrent (N=198)						P.inter	P.inter.FDR
	OR	SE_logOR	CI_lower	CI_upper	P	P.FDR	OR	SE_logOR	CI_lower	CI_upper	P	P.FDR		
PANSS	0.416	0.308	0.213	0.726	0.004	0.011	0.862	0.154	0.636	1.166	0.336	0.559	0.122	0.244
PANSS_P	0.473	0.277	0.262	0.788	0.007	0.011	0.917	0.148	0.685	1.226	0.559	0.559	0.052	0.208
PANSS_N	0.452	0.297	0.239	0.779	0.008	0.011	0.774	0.152	0.571	1.040	0.092	0.368	0.183	0.244
PANSS_G	0.713	0.243	0.434	1.137	0.164	0.164	0.900	0.152	0.666	1.212	0.487	0.559	0.465	0.465

Antipsychotics with stronger or milder metabolic effects														
PANSS	Stronger (N=124)						Milder (N=168)						P.inter	P.inter.FDR
	OR	SE_logOR	CI_lower	CI_upper	P	P.FDR	OR	SE_logOR	CI_lower	CI_upper	P	P.FDR		
PANSS	0.616	0.244	0.374	0.982	0.047	0.188	0.802	0.154	0.589	1.083	0.153	0.204	0.505	0.727
PANSS_P	0.838	0.222	0.537	1.294	0.428	0.428	0.734	0.155	0.537	0.991	0.047	0.094	0.727	0.727
PANSS_N	0.765	0.239	0.473	1.216	0.262	0.349	0.655	0.162	0.471	0.894	0.009	0.036	0.356	0.727
PANSS_G	0.762	0.236	0.475	1.205	0.250	0.349	0.897	0.152	0.664	1.207	0.474	0.474	0.616	0.727

Baseline diabetes status														
PANSS	With prediabetics or diabetes (N=44)						Without prediabetes or diabetes (N=248)						P.inter	P.inter.FDR
	OR	SE_logOR	CI_lower	CI_upper	P	P.FDR	OR	SE_logOR	CI_lower	CI_upper	P	P.FDR		
PANSS	0.419	0.604	0.103	1.248	0.150	0.157	0.728	0.138	0.552	0.950	0.021	0.042	0.561	0.649
PANSS_P	0.405	0.569	0.109	1.125	0.112	0.157	0.749	0.137	0.570	0.975	0.034	0.045	0.649	0.649
PANSS_N	0.445	0.572	0.123	1.279	0.157	0.157	0.700	0.140	0.529	0.915	0.010	0.040	0.391	0.649
PANSS_G	0.146	1.023	0.009	0.688	0.060	0.157	0.861	0.134	0.659	1.119	0.266	0.266	0.130	0.520

Gender														
PANSS	Male (N=132)						Female (N=160)						P.inter	P.inter.FDR
	OR	SE_logOR	CI_lower	CI_upper	P	P.FDR	OR	SE_logOR	CI_lower	CI_upper	P	P.FDR		
PANSS	0.643	0.217	0.410	0.969	0.042	0.056	0.853	0.177	0.601	1.205	0.368	0.491	0.593	0.791
PANSS_P	0.610	0.212	0.393	0.910	0.020	0.056	0.899	0.173	0.638	1.262	0.537	0.537	0.419	0.791
PANSS_N	0.623	0.221	0.395	0.947	0.032	0.056	0.743	0.180	0.518	1.052	0.098	0.392	0.917	0.917
PANSS_G	0.911	0.197	0.613	1.340	0.635	0.635	0.822	0.171	0.585	1.147	0.251	0.491	0.540	0.791

Note: Adjusted for age, age^2, gender, center, first five principal components, course of disease, previous drug usage, assigned medication;
Patients with baseline glucose >= 100.8 mg/dL (5.6 mmol/L) were classified into the prediabetes or diabetes group, the remaining patients were in the non-diabetes or prediabetes group;
Individuals with < 2 years of first-episode SCZ course, and < 14 days of antipsychotic exposure, were classified as first-episode, drug-naïve. The remaining patients were categorized as recurrent. Patients prescribed risperidone, olanzapine, quetiapine, or clozapine were grouped as receiving medication with stronger metabolic side effects; Others (prescribing aripiprazole, ziprasidone, or perphenazine) comprised the milder-metabolic-effect group.
False discovery rate (FDR)-corrected P-value < 0.05 was applied as the significant threshold accounting for multiple tests across drug targets and outcomes. The conventional P<0.05 was applied as the threshold of suggestive significance. Better antipsychotic efficacy: PANSS percentage reduction > median value of PANSS percentage reduction; The standard error (SE) corresponds to the beta coefficient derived from the natural logarithm of the odds ratio (log OR).
Abbreviations: PANSS, Positive and Negative Syndrome Scale; N, Negative scale; G, General psychopathology scale; P, Positive scale; SE, standard error; GRS, genetic risk score; CAPEC, Chinese Antipsychotics Pharmacogenetics Consortium.

Table S47. Descriptive analysis of glucose and TG by GRSs of *APOC3* and *GCK* in the CAPOC study. Related to Figure 1.

Characteristics (mg/dL)	Statistics	Both lower (N=498)	*APOC3*_TG GRS higher (N=555)	*GCK* GRS higher (N=556)	Both higher (N=502)
Glucose (mg/dL)	Mean	86.6	87.3	86.2	86.4
	Median	85.9	86.9	85.0	85.4
	Q1	77.9	78.4	77.1	77.4
	Q3	93.7	95.5	93.2	93.8
TG (mg/dL)	Mean	108.0	98.7	106.6	96.2
	Median	94.3	85.9	93.9	85.5
	Q1	70.9	62.0	67.3	62.9
	Q3	133.5	118.7	134.8	117.8
**Characteristics (mg/dL)**	**Statistics**	**Both lower (N=428)**	***APOC3*_TC GRS higher (N=625)**	***GCK* GRS higher (N=484)**	**Both higher (N=574)**
Glucose (mg/dL)	Mean	86.3	87.4	86.4	86.2
	Median	85.6	87.2	85.2	85.2
	Q1	78.0	78.4	77.1	77.3
	Q3	93.7	95.5	93.7	93.7
TC (mg/dL)	Mean	161.1	157.2	161.2	158.8
	Median	155.5	154.3	159.1	154.9
	Q1	133.8	135.0	136.0	135.3
	Q3	183.4	175.9	181.0	179.4

Abbreviations: GRS, genetic risk score; TG, triglyceride; TC, total cholesterol; CAPOC, Chinese Antipsychotics Pharmacogenomics Consortium

Table S48. Joint associations between *APOC3, GCK* GRSs and TG, glucose in the CAPOC study. Related to Figure 1.

Trait	Group	Beta	SE	CI_lower	CI_upper	P
***APOC3*_TG * *GCK*_Glucose**						
Glucose	*APOC3* GRS higher	0.972	0.804	-0.605	2.549	0.227
	GCK GRS higher	-0.002	0.805	-1.581	1.577	0.998
	Both higher	0.187	0.824	-1.428	1.803	0.820
TG	*APOC3* GRS higher	-9.213	3.186	-15.460	-2.965	0.004
	GCK GRS higher	-0.830	3.190	-7.085	5.425	0.795
	Both higher	-11.225	3.264	-17.626	-4.823	0.001
***APOC3*_TC * *GCK*_Glucose**						
Glucose	*APOC3* GRS higher	1.240	0.818	-0.365	2.845	0.130
	GCK GRS higher	0.343	0.865	-1.355	2.040	0.692
	Both higher	0.287	0.833	-1.346	1.920	0.730
TC	*APOC3* GRS higher	-4.385	2.067	-8.439	-0.331	0.034
	GCK GRS higher	0.065	2.186	-4.223	4.352	0.976
	Both higher	-1.997	2.103	-6.121	2.127	0.342

Adjusted for age, age^2, gender, center, first five principal components, course of disease, previous drug usage;

Abbreviations: SE, standard error; GRS, genetic risk score; TG, triglyceride; TC, total cholesterol; CAPOC, Chinese Antipsychotics Pharmacogenomics Consortium.

Table S49. Interactive associations between *APOC3, GCK* GRSs and measured glucose, TG in the CAPOC study. Related to Figure 1.

Trait	GRS	Beta	SE	CI_lower	CI_upper	P
***APOC3*_TG * *GCK*_Glucose**						
Glucose	Higher *APOC3* GRS	0.230	0.284	-0.327	0.788	0.417
	Higher *GCK* GRS	-0.634	0.284	-1.190	-0.078	0.026
	Both higher	-0.359	0.281	-0.911	0.193	0.202
TG	Higher *APOC3* GRS	-6.332	1.124	-8.536	-4.129	1.98E-08
	Higher *GCK* GRS	-1.038	1.122	-3.238	1.162	0.355
	Both higher	-0.845	1.113	-3.026	1.337	0.448
***APOC3*_TC * *GCK*_Glucose**						
Glucose	Higher *APOC3* GRS	0.120	0.286	-0.441	0.681	0.674
	Higher *GCK* GRS	-0.655	0.283	-1.211	-0.100	0.021
	Both higher	-0.237	0.291	-0.807	0.333	0.415
TC	Higher *APOC3* GRS	-1.929	0.723	-3.346	-0.511	7.70E-03
	Higher *GCK* GRS	0.398	0.716	-1.005	1.802	0.578
	Both higher	-0.149	0.734	-1.589	1.292	0.840

Adjusted for age, age^2, gender, center, first five principal components, course of disease, previous drug usage;

Abbreviations: SE, standard error; GRS, genetic risk score; TG, triglyceride; TC, total cholesterol; CAPOC, Chinese Antipsychotics Pharmacogenomics Consortium.

Table S50. Joint associations between *APOC3, GCK* GRSs and PANSS percentage reduction in the CAPOC study. Related to Figure 1.

PANSS	Group	Beta	SE	CI_lower	CI_upper	P
***APOC3*_TG * *GCK*_Glucose**						
PANSS	*APOC3* GRS higher	0.570	1.746	-2.854	3.994	0.744
	GCK GRS higher	-3.463	1.748	-6.891	-0.035	0.048
	Both higher	-2.882	1.789	-6.389	0.626	0.107
PANSS_P	*APOC3* GRS higher	0.608	1.401	-2.139	3.355	0.664
	GCK GRS higher	-1.954	1.403	-4.704	0.797	0.164
	Both higher	-2.168	1.435	-4.983	0.647	0.131
PANSS_N	*APOC3* GRS higher	0.868	1.341	-1.762	3.498	0.518
	GCK GRS higher	-3.075	1.343	-5.709	-0.442	0.022
	Both higher	-2.045	1.374	-4.740	0.650	0.137
PANSS_G	*APOC3* GRS higher	0.003	1.133	-2.218	2.224	0.998
	GCK GRS higher	-2.202	1.134	-4.426	0.022	0.052
	Both higher	-2.146	1.160	-4.422	0.129	0.065
***APOC3*_TC * *GCK*_Glucose**						
PANSS	*APOC3* GRS higher	2.090	1.776	-1.394	5.574	0.240
	GCK GRS higher	-2.936	1.879	-6.620	0.748	0.118
	Both higher	-1.662	1.807	-5.206	1.882	0.358
PANSS_P	*APOC3* GRS higher	1.361	1.426	-1.435	4.157	0.340
	GCK GRS higher	-1.870	1.508	-4.826	1.087	0.215
	Both higher	-1.314	1.450	-4.158	1.530	0.365
PANSS_N	*APOC3* GRS higher	1.936	1.364	-0.739	4.610	0.156
	GCK GRS higher	-3.285	1.442	-6.114	-0.457	0.023
	Both higher	-0.725	1.387	-3.445	1.996	0.601
PANSS_G	*APOC3* GRS higher	1.160	1.153	-1.101	3.420	0.314
	GCK GRS higher	-1.570	1.219	-3.960	0.821	0.198
	Both higher	-1.415	1.173	-3.714	0.885	0.228

Note: Adjusted for age, age^2, gender, center, first five principal components, course of disease, previous drug usage, assigned medication;

*Bonferroni correction P-value < 0.05/4=0.0125 was applied as the significant threshold accounting for multiple tests across PANSS subscales. The conventional P<0.05 was applied as the threshold of suggestive significance.

Abbreviations: PANSS, Positive and Negative Syndrome Scale; N, Negative scale; G, General psychopathology scale; P, Positive scale; SE, standard error; GRS, genetic risk score; TG, triglyceride; TC, total cholesterol; CAPOC, Chinese Antipsychotics Pharmacogenomics Consortium.

Table S51. Joint associations between *APOC3, GCK* GRSs and better antipsychotic efficacy in the CAPOC study. Related to Figure 1.

PANSS	Group	OR	SE_logOR	CI_lower	CI_upper	P
***APOC3*_TG * *GCK*_Glucose**						
PANSS	*APOC3* GRS higher	1.145	0.128	0.891	1.473	0.289
	GCK GRS higher	0.848	0.128	0.659	1.090	0.199
	Both higher	0.976	0.131	0.754	1.262	0.850
PANSS_P	*APOC3* GRS higher	1.210	0.127	0.943	1.554	0.134
	GCK GRS higher	0.955	0.127	0.744	1.226	0.717
	Both higher	0.798	0.131	0.617	1.031	0.084
PANSS_N	*APOC3* GRS higher	1.179	0.127	0.919	1.514	0.195
	GCK GRS higher	0.773	0.127	0.602	0.992	0.043
	Both higher	0.993	0.130	0.769	1.281	0.954
PANSS_G	*APOC3* GRS higher	1.019	0.128	0.793	1.310	0.882
	GCK GRS higher	0.781	0.128	0.608	1.004	0.054
	Both higher	0.817	0.131	0.631	1.056	0.123
***APOC3*_TC * *GCK*_Glucose**						
PANSS	*APOC3* GRS higher	1.258	0.130	0.974	1.625	0.079
	GCK GRS higher	0.859	0.138	0.655	1.126	0.271
	Both higher	1.067	0.133	0.823	1.384	0.624
PANSS_P	*APOC3* GRS higher	1.312	0.130	1.018	1.693	0.036
	GCK GRS higher	0.941	0.137	0.719	1.231	0.659
	Both higher	0.923	0.132	0.713	1.195	0.543
PANSS_N	*APOC3* GRS higher	1.338	0.130	1.038	1.725	0.025
	GCK GRS higher	0.788	0.137	0.602	1.030	0.081
	Both higher	1.108	0.131	0.856	1.433	0.437
PANSS_G	*APOC3* GRS higher	1.080	0.130	0.837	1.394	0.553
	GCK GRS higher	0.788	0.138	0.601	1.032	0.084
	Both higher	0.861	0.132	0.664	1.117	0.260

Note: Adjusted for age, age^2, gender, center, first five principal components, course of disease, previous drug usage, assigned medication;

*Bonferroni correction P-value < 0.05/4=0.0125 was applied as the significant threshold accounting for multiple tests across PANSS subscales. The conventional P<0.05 was applied as the threshold of suggestive significance. Better antipsychotic efficacy: PANSS percentage reduction > median value of PANSS percentage reduction; The standard error (SE) corresponds to the beta coefficient derived from the natural logarithm of the odds ratio (log OR).

Abbreviations: PANSS, Positive and Negative Syndrome Scale; N, Negative scale; G, General psychopathology scale; P, Positive scale; SE, standard error; GRS, genetic risk score; TG, triglyceride; TC, total cholesterol; CAPOC, Chinese Antipsychotics Pharmacogenomics Consortium.

Table S52. Interactive associations between *APOC3, GCK* GRSs and PANSS percentage reduction in the CAPOC study. Related to Figure 1.

PANSS	GRS	beta	SE	CI_lower	CI_upper	P
***APOC3*_TG * *GCK*_Glucose**						
PANSS	Higher *APOC3* GRS	0.631	0.618	-0.580	1.842	0.307
	Higher *GCK* GRS	-1.676	0.617	-2.885	-0.467	0.007*
	Both higher	0.233	0.612	-0.967	1.432	0.704
PANSS_P	Higher *APOC3* GRS	0.170	0.496	-0.802	1.143	0.731
	Higher *GCK* GRS	-1.047	0.495	-2.018	-0.076	0.035
	Both higher	0.052	0.491	-0.911	1.015	0.916
PANSS_N	Higher *APOC3* GRS	1.121	0.474	0.192	2.051	0.018
	Higher *GCK* GRS	-1.566	0.473	-2.494	-0.638	0.001*
	Both higher	0.298	0.469	-0.622	1.218	0.525
PANSS_G	Higher *APOC3* GRS	0.241	0.401	-0.545	1.026	0.548
	Higher *GCK* GRS	-1.105	0.400	-1.889	-0.320	0.006*
	Both higher	0.019	0.397	-0.759	0.797	0.962
***APOC3*_TC * *GCK*_Glucose**						
PANSS	Higher *APOC3* GRS	0.356	0.622	-0.863	1.575	0.567
	Higher *GCK* GRS	-1.709	0.615	-2.916	-0.502	0.006*
	Both higher	0.605	0.631	-0.633	1.843	0.338
PANSS_P	Higher *APOC3* GRS	0.058	0.499	-0.921	1.036	0.908
	Higher *GCK* GRS	-1.065	0.494	-2.034	-0.096	0.031
	Both higher	0.316	0.507	-0.678	1.310	0.533
PANSS_N	Higher *APOC3* GRS	0.864	0.477	-0.071	1.799	0.070
	Higher *GCK* GRS	-1.603	0.472	-2.529	-0.677	0.001*
	Both higher	0.647	0.484	-0.303	1.597	0.182
PANSS_G	Higher *APOC3* GRS	-0.021	0.403	-0.812	0.769	0.958
	Higher *GCK* GRS	-1.131	0.399	-1.914	-0.348	0.005*
	Both higher	0.253	0.410	-0.551	1.056	0.537

Note: Adjusted for age, age^2, gender, center, first five principal components, course of disease, previous drug usage, assigned medication;

*Bonferroni correction P-value < 0.05/4=0.0125 was applied as the significant threshold accounting for multiple tests across PANSS subscales. The conventional P<0.05 was applied as the threshold of suggestive significance.

Abbreviations: PANSS, Positive and Negative Syndrome Scale; N, Negative scale; G, General psychopathology scale; P, Positive scale; SE, standard error; GRS, genetic risk score; TG, triglyceride; TC, total cholesterol; CAPOC, Chinese Antipsychotics Pharmacogenomics Consortium.

Table S53. Interactive associations between *APOC3, GCK* GRSs and better antipsychotic efficacy in the CAPOC study. Related to Figure 1.

PANSS	GRS	OR	SE_logOR	CI_lower	CI_upper	P
***APOC3*_TG * *GCK*_Glucose**						
PANSS	Higher *APOC3* GRS	1.079	0.045	0.987	1.179	0.095
	Higher *GCK* GRS	0.922	0.045	0.843	1.008	0.074
	Both higher	0.993	0.045	0.909	1.085	0.884
PANSS_P	Higher *APOC3* GRS	0.992	0.045	0.908	1.083	0.851
	Higher *GCK* GRS	0.898	0.045	0.822	0.981	0.018
	Both higher	0.940	0.045	0.860	1.026	0.166
PANSS_N	Higher *APOC3* GRS	1.149	0.045	1.052	1.255	0.002*
	Higher *GCK* GRS	0.909	0.045	0.831	0.993	0.034
	Both higher	1.015	0.045	0.930	1.109	0.736
PANSS_G	Higher *APOC3* GRS	1.038	0.045	0.949	1.134	0.415
	Higher *GCK* GRS	0.913	0.045	0.835	0.998	0.046
	Both higher	1.012	0.045	0.926	1.105	0.797
***APOC3*_TC * *GCK*_Glucose**						
PANSS	Higher *APOC3* GRS	1.048	0.046	0.959	1.147	0.301
	Higher *GCK* GRS	0.919	0.045	0.840	1.004	0.061
	Both higher	1.023	0.046	0.934	1.121	0.627
PANSS_P	Higher *APOC3* GRS	1.017	0.045	0.930	1.111	0.713
	Higher *GCK* GRS	0.897	0.045	0.821	0.980	0.016
	Both higher	0.970	0.046	0.886	1.062	0.515
PANSS_N	Higher *APOC3* GRS	1.128	0.045	1.032	1.233	0.008*
	Higher *GCK* GRS	0.905	0.045	0.828	0.989	0.027
	Both higher	1.047	0.046	0.956	1.146	0.323
PANSS_G	Higher *APOC3* GRS	1.007	0.046	0.921	1.101	0.877
	Higher *GCK* GRS	0.910	0.045	0.833	0.995	0.039
	Both higher	1.041	0.046	0.951	1.141	0.383

Note: Adjusted for age, age^2, gender, center, first five principal components, course of disease, previous drug usage, assigned medication;

*Bonferroni correction P-value < 0.05/4=0.0125 was applied as the significant threshold accounting for multiple tests across PANSS subscales. The conventional P<0.05 was applied as the threshold of suggestive significance. Better antipsychotic efficacy: PANSS percentage reduction > median value of PANSS percentage reduction; The standard error (SE) corresponds to the beta coefficient derived from the natural logarithm of the odds ratio (log OR).

Abbreviations: PANSS, Positive and Negative Syndrome Scale; N, Negative scale; G, General psychopathology scale; P, Positive scale; SE, standard error; GRS, genetic risk score; TG, triglyceride; TC, total cholesterol; CAPOC, Chinese Antipsychotics Pharmacogenomics Consortium.

Table S54. Multi-omics datasets. Related to STAR Methods.

Study	Abbreviation	Type of data	Sample size	Ancestry	PMID
Chinese Antipsychotics Pharmacogenomics Consortium	CAPOC	Individual-level phenotype and genotype data	2111	East Asians	29503163
Chinese Antipsychotics Pharmacogenetics Consortium	CAPEC	Individual-level phenotype and genotype data	292	East Asians	29503163
Global Lipids Genetics Consortium	GLGC	GWAS summary statistics of lipids	146492	East Asians	36575460
Taiwan Biobank	Taiwan Biobank	GWAS summary statistics of glucose	92615	East Asians	38116116
Anti-Aging Study Cohort, Cardiometabolic Genome Epidemiology Kita-Nagoya Genomic Quad, Cardiometabolic Genome Epidemiology Kita-Nagoya Genomic Omni, Cebu Longitudinal Health and Nutrition Survey, China Health and Nutrition Survey, Korean Association Resource, Multi-Ethnic Study of Atherosclerosis, Nagahama Study, Nutrition and Health of Aging Population in China, "Singapore Prospective Study Program, Illumina1Mduov3", "Singapore Prospective Study Program, Illumina550", "Singapore Prospective Study Program, Illumina610Quad", Taiwan Metabochip Consortium, The Colorectal Cancer Study, Shanghai Men's Health Study	AASC, CAGE-KING-Quad, CAGE-KING-Omni, CLHNS, CHNS, KARE, MESA, Nagahama Study, NHAPC, SP2-Illumina1Mduov3, SP2-Illumina550, SP2-Illumina610Quad, TAICHI, CRC, Shanghai Men's Health Study	GWAS summary statistics of fasting insulin	29792	East Asians	34059833
Biobank of Japan	BBJ	GWAS summary statistics of systolic blood pressure	145505	East Asians	34594039
Biobank of Japan	BBJ	GWAS summary statistics of schizophrenia	177893	East Asians	34594039
Type 2 Diabetes Global Genetics Initative	T2DGGI	GWAS summary statistics of type 2 diabetes	427504	East Asians	38374256
GWAS summary statistics of oral glucose tolerance test-2h from the Shenzhen Baoan Women’s and Children’s Hospital (Shenzhen, China)	GWAS summary statistics of oral glucose tolerance test-2h from the Shenzhen Baoan Women’s and Children’s Hospital (Shenzhen, China)	GWAS summary statistics of oral glucose tolerance test-2h	24931	East Asians	38372780
Taiwan Biobank	Taiwan Biobank	GWAS summary statistics of Hemoglobin A1c levels	92615	East Asians	38116116
Biobank of Japan	BBJ	GWAS summary statistics of coronary artery disease	178726	East Asians	doi.org/10.1101/2021.09.03.21262975
Biobank of Japan	BBJ	GWAS summary statistics of body mass index	158284	East Asians	28892062

GWAS summary statistics of metabolic syndrome among Koreans	GWAS summary statistics of metabolic syndrome among Koreans	GWAS summary statistics of metabolic syndrome	62314	East Asians	39349817
Guangzhou Nutrition and Health study	GNHS	pQTL summary statistics in the blood	2410	East Asians	36797296
Diversity and scale: Genetic architecture of 2068 traits in the VA Million Veteran Program	MVP	GWAS summary statistics of hyperlipidemia	426603	Europeans	39024449
UK Biobank	UKB	GWAS summary statistics of sphingomyelin levels	115006	Europeans	35213538
The Qatar Genome Program Research Consortium	The Qatar Genome Program Research Consortium	GWAS summary statistics of beta-endorphin levels	2935	Greater Middle Eastern	36168886
Six cohorts (Alzheimer's Disease Neuroimaging Initiative, Dominantly Inherited Alzheimer's Network, Knight-ADRC Memory and Aging Project, Ace Alzheimer Center Barcelona, Barcelona-1, and Parkinson's Progression Markers Initiative)	ADNI, DIAN, MAP, FACE, Barcelona-1, PPMI	pQTL summary statistics in the cerebrospinal fluid	3107	Europeans	37333337
The Adult Genotype Tissue Expression Project	GTEx v10	eQTL summary statistics in the brain_cortex	268	Europeans	32913098
The Adult Genotype Tissue Expression Project	GTEx v10	eQTL summary statistics in the brain_hypothalamus	256	Europeans	32913098
The Adult Genotype Tissue Expression Project	GTEx v10	eQTL summary statistics in the brain_cerebellum	264	Europeans	32913098
The Adult Genotype Tissue Expression Project	GTEx v10	eQTL summary statistics in the brain_nucleus_accumbens	284	Europeans	32913098
The Adult Genotype Tissue Expression Project	GTEx v10	eQTL summary statistics in the brain_caudate	298	Europeans	32913098
The Adult Genotype Tissue Expression Project	GTEx v10	eQTL summary statistics in the brain_hippocampus	254	Europeans	32913098
The Adult Genotype Tissue Expression Project	GTEx v10	eQTL summary statistics in the skeletal muscle	816	Europeans	32913098
The Adult Genotype Tissue Expression Project	GTEx v10	eQTL summary statistics in the pituitary	311	Europeans	32913098
The Adult Genotype Tissue Expression Project	GTEx v10	eQTL summary statistics in the visceral adipose	584	Europeans	32913098
The Adult Genotype Tissue Expression Project	GTEx v10	eQTL summary statistics in the liver	261	Europeans	32913098
The Adult Genotype Tissue Expression Project	GTEx v10	eQTL summary statistics in the artery_coronary	268	Europeans	32913098
The Adult Genotype Tissue Expression Project	GTEx v10	eQTL summary statistics in the small intestine - terminal ileum	206	Europeans	32913098
The Adult Genotype Tissue Expression Project	GTEx v10	eQTL summary statistics in the pancreas	362	Europeans	32913098

Table S55. Target genes for antidiabetic and lipid-lowering drugs identified using the DrugBank. Related to Figure 1.

Drug class	ATC code	Name	Gene		
**DRUGS USED IN DIABETES**					
Biguanides	A10BA03	buformin	*ABCB11*		
Sulfonylureas	A10BB01	glibenclamide	*ABCB11*		
Glucagon-like peptide-1 (GLP-1) analogues	A10BJ01	exenatide	*GLP1R*		
	A10BJ02	liraglutide	*GLP1R*		
	A10BJ03	lixisenatide	*GLP1R*		
	A10BJ04	albiglutide	*GLP1R*		
	A10BJ05	dulaglutide	*GLP1R*		
	A10BJ06	semaglutide	*GLP1R*		
Other blood glucose-lowering drugs, excl. insulins	A10BX05	pramlintide	*GLP1R*		
	A10BX16	tirzepatide	*GLP1R*		
	A10BX18	dorzagliatin	*GCK*		
**LIPID MODIFYING AGENTS**					
HMG CoA reductase inhibitors	C10AA01	simvastatin	*HMGCR*		
	C10AA02	lovastatin	*LDLR*	*LPA*	*HMGCR*
	C10AA03	pravastatin	*HMGCR*		
	C10AA04	fluvastatin	*HMGCR*		
	C10AA05	atorvastatin	*HMGCR*		
	C10AA06	cerivastatin	*HMGCR*		
	C10AA07	rosuvastatin	*HMGCR*		
	C10AA08	pitavastatin	*HMGCR*		
Fibrates	C10AB01	clofibrate	*LPL*		
	C10AB02	bezafibrate	*PPARG*		
	C10AB04	gemfibrozil	*LPL*		
Nicotinic acid	C10AD02	nicotinic acid	*HCAR3*	*HCAR2*	
Other lipid modifying agents	C10AX02	probucol	*ABCA1*		
	C10AX12	lomitapide	*MTTP*		
	C10AX13	evolocumab	*PCSK9*		
	C10AX14	alirocumab	*PCSK9*		
	C10AX16	inclisiran	*PCSK9*		
	C10AX17	evinacumab	*ANGPTL3*		
	C10AX18	volanesorsen	*APOC3*		
	C10AX11	mipomersen	*APOB*		
	-	torcetrapib	*CETP*		

Notes: Targets for which the drug acts as an agonist are marked in blue; Targets for which the drug acts as an inhibitor are marked in black.
